# Preserved ratio impaired spirometry, plasma proteomics, and incident heart failure

**DOI:** 10.1016/j.jare.2025.10.009

**Published:** 2025-10-12

**Authors:** Yang Geng, Yi Ding, Yalong Pei, Xiya Zhou, Xujia Lu, Mingcan Xu, Yan Borné, Chaofu Ke

**Affiliations:** a Department of Epidemiology and Biostatistics, School of Public Health, Suzhou Medical College of Soochow University, Suzhou, Jiangsu, China; b Department of Preventive Medicine, College of Clinical Medicine, Suzhou Vocational Health College, Suzhou, Jiangsu, China; c Department of Clinical Sciences Malmö, Lund University, Malmö, Sweden

**Keywords:** Preserved ratio impaired spirometry, Heart failure, Mendelian randomization, Plasma proteomics

## Abstract

**Introduction:**

Epidemiological evidence suggests the association of preserved ratio impaired spirometry (PRISm) at baseline with the risk of heart failure (HF), however, the associations between transitions of PRISm and incident HF remain to be clarified. Furthermore, the causal relationship and underlying biological mechanisms linking PRISm and HF are unclear.

**Objectives:**

This study aimed to investigate the association between the normal-PRISm transition and incident HF, and further evaluate the causal relationship and underlying molecular mechanisms linking PRISm and HF.

**Methods:**

A total of 32,202 individuals with repeated spirometry and 40,047 individuals with proteomic data were included from the UK Biobank. Cox proportional hazards models were employed to estimate the association between the normal-PRISm transition and HF risk. Mendelian randomization (MR) analysis was performed to investigate the causal relationship between PRISm and HF. Mediation analysis was conducted to elucidate the mediation effects of 2,923 plasma proteins.

**Results:**

Compared with consistent normal spirometry, the normal-PRISm transition was associated with an elevated risk of HF (hazard ratio: 1.814, 95 % CI: 1.128, 2.919). The MR suggested a causal link between PRISm and HF (odds ratio: 1.488, 95 % CI: 1.040, 2.130). In addition, 653 proteins demonstrated positive mediation effects, and the overall proteomic score mediated 59.63 % (95% CI: 46.98 %, 77.12 %) of the relationship between PRISm and HF.

**Conclusion:**

Individuals with the normal-PRISm transition were at an elevated risk of HF. The MR results suggested the causal link between PRISm and HF, and a variety of plasma proteins substantially mediated the relationship between PRISm and HF.

## Highlights

• The transition from normal spirometry to preserved ratio impaired spirometry (PRISm) associated with heart failure (HF) risk.
• The Mendelian randomization analysis provided evidence for the causal link between PRISm and HF.
• A wide range of plasma proteins substantially mediated the relation between PRISm and HF.
• This study underscores continuous screening of PRISm for preventing HF, and provides molecular targets for intervention.

## Graphical abstract

**Figure:**
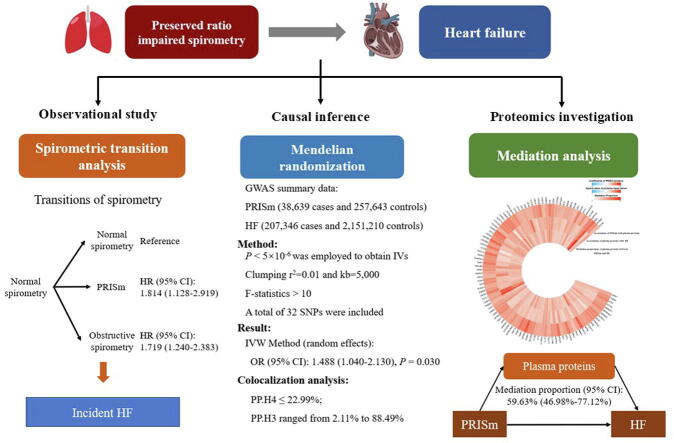

## Introduction

Despite a declining trend in cardiovascular disease (CVD)-related mortality [1,2], the global burden of disease attributed to CVD remains a major concern [3], and the progressions from acute cardiovascular events to chronic diseases, including heart failure (HF), have become increasingly prevalent [1]. HF, affecting approximately 55 million people globally [4], has emerged as a significant global health problem associated with high morbidity and mortality [2]. The total direct medical costs for patients with HF in the United States reached $21 billion and are projected to rise to $53 billion in 2030 [5]. Considering the notable disease burden posed by HF, identification of effective prevention and control measures for HF should constitute an urgent public health and clinical priority.

Preserved ratio impaired spirometry (PRISm), presented in about 10 % of the general population [[6], [7], [8]], is defined as a reduced forced expiratory volume in 1 s (FEV1) of lower than 80 % predicted and a preserved FEV1/forced vital capacity (FVC) ratio of more than or equal to 0.70. Existing studies have unveiled a prospective association between baseline PRISm and subsequent incident HF [9,10]. However, these studies were based on a single spirometry at baseline, which did not capture longitudinal variations in pulmonary function [[11], [12], [13]]. Furthermore, evidence has shown that longitudinal pulmonary function transitions were associated with risks of cardiovascular morbidity and mortality [9,12,14], thereby supporting the potential value of using repeated spirometry measurements in risk stratification. However, the associations between spirometric transitions and the risk of HF remain inadequately understood. To further explore the role of continuous monitoring of PRISm in preventing HF, it is essential to investigate the prospective association of the normal-PRISm transition with the risk of HF.

Mendelian randomization (MR) is an effective tool for causal inference by employing genetic variants that are robustly associated with the exposure as instrumental variables (IVs), thereby minimizing potential confounding and reverse causality [15]. An increasing number of studies have demonstrated that PRISm and HF could trigger shared pathophysiologic injuries, such as systemic inflammation and metabolic dysregulation [[16], [17], [18], [19]], providing biological plausibility for their causal relationship. Although prior epidemiological studies have reported the prospective association between PRISm at baseline and incident HF [9,10], MR evidence addressing the causal link is currently lacking. Furthermore, molecular foundations linking PRISm and incident HF remain unexplored. Plasma proteome, including critical cellular regulators and proteins released from damaged cells, is largely affected by environmental exposures [20]. Plasma proteins are also demonstrated to be important indicators of disease development [21]. Therefore, understanding how PRISm could regulate plasma proteins and how these altered proteins then affect the incidence of HF would help identify potential therapeutic targets for HF.

To fill in these research gaps, we first investigated the prospective association of the normal-PRISm transition with the risk of HF using repeated spirometry data from the UK Biobank. Subsequently, a two-sample MR analysis was employed to examine the causal relationship between PRISm and HF. Last, we conducted a mediation analysis to quantify the mediating roles of the plasma proteins between PRISm and HF.

## Material and methods

### Study population

The UK Biobank cohort recruited approximately 500,000 residents of the United Kingdom (aged 37–73 years) between 2006 and 2010, representing the largest prospective cohort ever established in England [22]. Ethical approval for the UK Biobank study was granted by the Northwest Multi-Centre Research Ethics Committee (REC reference: 21/NW/0157); all participants signed written informed consent. Subsequently, participants completed touch-screen questionnaires and nurse interviews for the baseline survey concerning lifestyle, dietary, environment and reproductive factors, and undertook a variety of physical measurements and collection of biological samples [23]. The UK Biobank tracks participants’ morbidity and mortality via electronic linkage to the hospitalization and death registry [22,23].

**Fig. 1:**
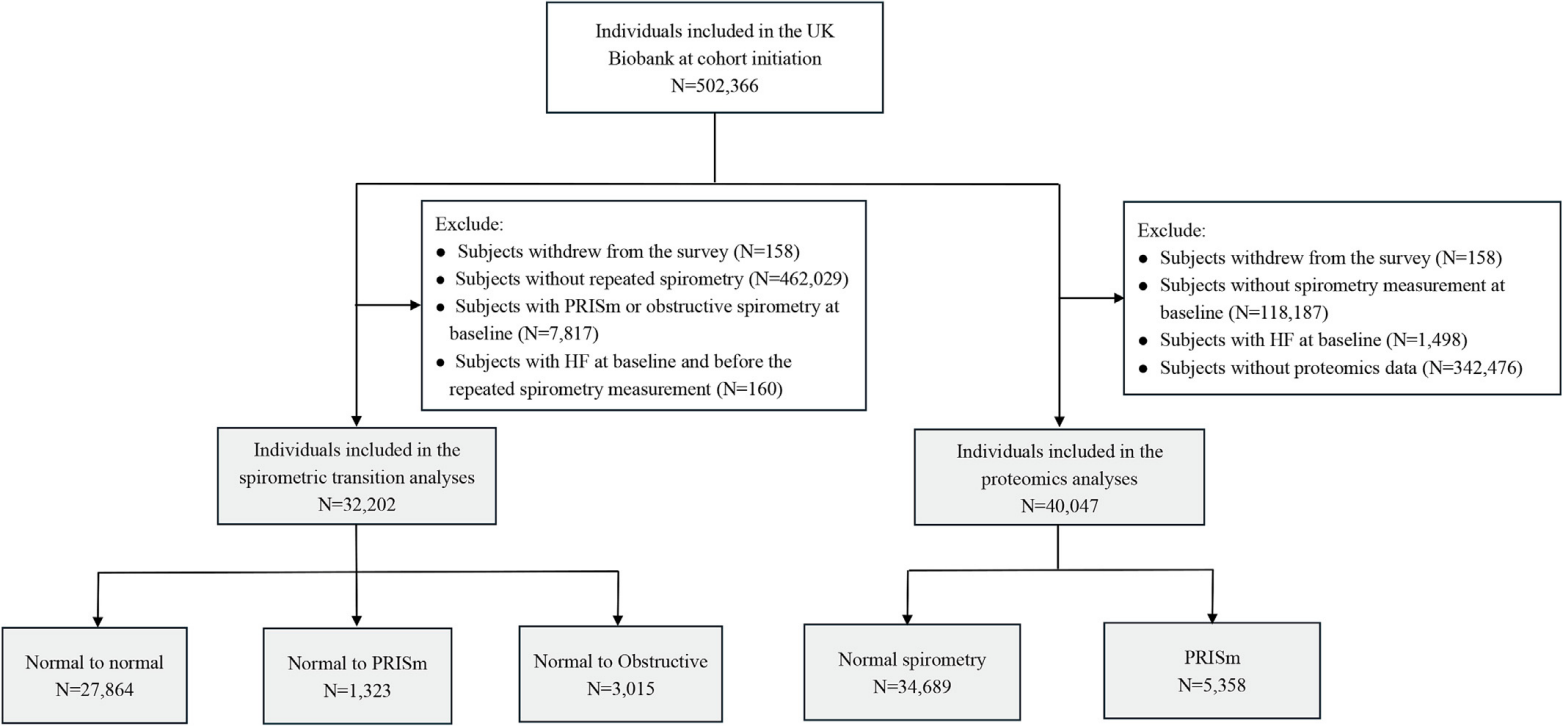
Flow chart of participants selection.

In the current study, for the spirometric transition analysis, exclusion criteria comprised any of the following conditions: (a) withdrew from the survey at baseline, (b) did not have repeated spirometry, (c) had PRISm or obstructive spirometry at baseline, and (d) had HF at baseline or before the repeated spirometry measurement. Ultimately, 32,202 individuals free of HF and with repeated spirometry were included. Furthermore, individuals who had any of the following conditions were excluded for the proteomics analysis: (a) withdrew from the survey at baseline, (b) did not have the spirometry measurement at baseline, (c) had HF at baseline, and (d) without proteomics data. In total, 40,047 participants with valid data on plasma proteins were included. Fig. 1 presents the flow chart of participant selection.

### Assessment of spirometry

At the time of recruitment, trained medical technicians and nurses performed pre-bronchodilator spirometry in the UK Biobank with the use of a Vitalograph Pneumotrac 6800 spirometer [24]. Participants were instructed to undergo two to three blows over approximately 6 min, each measurement was preceded by instrument calibration. A computer was used to compare the initial two blows’ repeatability, and if the previous waves were acceptable (defined as two measurements differing by less than 5 %), the third blow was skipped. The highest measurements recorded were utilized for subsequent analyses. Based on the GLI-2012 (Global Lung Initiative equation-2012), we employed the RSpiro package in R (version 4.3.3) to calculate the predicted values of FEV1 and FVC [25]. FEV1 (%, predicted) and FVC (%, predicted) were derived by dividing the original values by the corresponding predicted values.

Three spirometry groups were defined based on lung function at baseline: PRISm was defined as an FEV1/FVC ratio ≥0.70 and FEV1 <80 % predicted; normal spirometry was defined as having an FEV1/FVC ratio ≥0.70 and FEV1 ≥80 % predicted; obstructive spirometry was described as an FEV1/FVC ratio <0.70 [6]. Participants residing close to an assessment center were re-invited for follow-up measurement. Between 2014 and 2020, approximately 40,000 participants were re-examined for spirometry. Individuals with normal spirometry at baseline were further categorized according to their repeated spirometry measurement, including the normal-normal transition, the normal-PRISm transition and the normal-obstructive transition [6]. Detailed spirometric trajectories are presented in Supplementary Fig. 1.

### Olink proteomics

The UK Biobank Pharma Proteomics Project (UKB-PPP) encompasses a comprehensive investigation into the plasma proteome. Using the Olink™ Explore 3072 Proximity Extension Assay, the UKB-PPP consortium collected blood plasma samples from approximately 54,000 UK Biobank participants and performed proteomic profiling. Olink Explore 3072 incorporates 8 panels, including cardiometabolic, inflammation, neurology, oncology, cardiometabolic Ⅱ, inflammation Ⅱ, neurology Ⅱ, and oncology Ⅱ, covering 2,923 distinct proteins [26]. The quality control processes for the Olink assay in the UK Biobank study ensure the absence of plate effects, batch effects, or abnormalities in protein coefficients of variation. Batch and plate contributed less than 10 % variability in 100 % and 99.5 % of proteins, respectively. In the current study, we utilized proteomics data from the baseline assessment of the UK Biobank study, which can be found in https://biobank.ndph.ox.ac.uk/showcase/label.cgi?id=1839.

### Outcomes

The main outcome of the present study was the occurrence of HF, defined by International Classification of Diseases, Tenth Revision, codes I11.0, I13.0, I13.2 and I50 (category ID 1712 in UK Biobank) [27]. Information on disease outcomes was mainly derived from health records including primary care, hospital admissions, mortality registries, and to a lesser extent, on self-reported data. The follow-up time for the spirometric transition analysis was defined as the period from the date of the repeated spirometry measurement to the diagnosis date of HF, date of death, or the last date of follow-up (December 2022). For the proteomics analysis, the survival time was calculated as the interval between the date of the baseline spirometry measurement and the diagnosis date of HF, date of death, or the last date of follow-up (December 2022), whichever came first. A schematic figure to illustrate the key time points of the cohort analyses is presented in Supplementary Fig. 1.

### Covariates

In this study, covariates encompass sociodemographic factors, lifestyle characteristics, biochemical indicators, coronary heart disease at baseline and stroke at baseline. Detailed descriptions of the covariates are presented in supplementary materials (Supplementary Method 1).

### Statistical analysis

Cox proportional hazards regression models were employed to estimate hazard ratios (HRs) and 95 % confidence intervals (95 % CIs) of the prospective associations between spirometric transitions and the risk of HF. Taking the normal-normal transition as the reference, we ran three distinct models: model 1 was unadjusted; model 2 was adjusted for age and sex; model 3 was adjusted for age, sex, ethnicity, household income, Townsend deprivation index, smoking status, alcohol intake, body mass index (BMI), physical activity, education status, hypertension, high density lipoprotein, triglycerides, glycosylated hemoglobin, glucose-lowering drugs, lipid-lowering drugs, coronary heart disease at baseline and stroke at baseline. We performed the following sensitivity analyses to ensure the robustness of the observed associations: First, to minimize the potential effect of reverse causality, individuals who developed HF within the first two years of follow-up were excluded. Second, we re-conducted the main analyses using a lower limit of normal (LLN) thresholds to define PRISm (FEV1/FVC ≥ LLN and FEV1 < 80 % predicted), normal spirometry (FEV1/FVC ≥ LLN and FEV1 ≥ 80 % predicted) and obstructive spirometry (FEV1/FVC < LLN). Third, we conducted a competing risks analysis, taking all-cause death as a competing event. Fourth, we examined potential interaction effects by incorporating interaction terms between transitions of normal spirometry and the covariates (age, sex, Townsend deprivation index), and further conducted stratified subgroup analyses by age, sex, and Townsend deprivation index.

A two-sample MR was conducted using summary-level genome-wide association study (GWAS) statistics to investigate the causal relationship between PRISm and HF. The GWAS data for individuals with PRISm was obtained from the IEU Open GWAS database (https://gwas.mrcieu.ac.uk/) [28], while the HF GWAS data was derived from the GWAS Catalog (https://www.ebi.ac.uk/gwas/) [29]. The inverse variance weighted (IVW) method was used as the primary analytical approach [30]. Furthermore, we included a series of complementary methods to assess the robustness of our findings, including MR-Egger regression, weighted median, weighted mode and robust adjusted profile score (RAPS). Detailed descriptions of the MR analysis can be found in the supplementary materials (Supplementary Method 2). Subsequently, we conducted several sensitivity analyses to further evaluate the validity of our results. Firstly, Cochran’s Q statistic was performed to detect heterogeneity among IVs. In cases of significant heterogeneity (*P* < 0.05), a random-effects IVW model was applied. Second, the MR pleiotropy residual sum and outlier (MR-PRESSO) analysis was further performed to detect and correct for outliers and moderate pleiotropy. If there were any outlier single nucleotide polymorphisms (SNPs), the MR analysis was performed again after the outliers were removed. Additionally, we conducted a leave-one-out (LOO) analysis to investigate the influence of individual SNPs on IVW results. Moreover, the Steiger directionality tests were conducted to investigate the possibility of reverse causality. At last, we computed the F-statistic for each SNP based on the following formula: *F* = (β/SE)^2^, where β represents the effect size and SE represents the standard error, to assess the strength of each IV [31]. An F-statistic value greater than 10 indicates strong instrument strength [31]. To assess potential overlap bias, we employed a publicly available overlap bias calculator (https://sb452.shinyapps.io/overlap/) [32]. All aforementioned analyses were based on the “TwoSampleMR” and “MR-PRESSO” packages in R (version 4.3.3) [33].

Based on summary GWAS data, colocalization analysis was performed to assess whether PRISm and HF share the same causal variant within a given region [34]. Specifically, we established a ±500 kb window around SNPs identified by our MR instruments to pinpoint pertinent genomic areas. Bayesian colocalization assessed shared genetic variations between traits via posterior probabilities (PP) for five hypotheses. More details of the colocalization analysis were presented in Supplementary Method 3.

Multiple linear regression models and Cox proportional hazards models were utilized to identify the proteins associated with baseline PRISm or incident HF, respectively. All analyses were adjusted as in Model 3. Thereafter, we employed the least absolute shrinkage and selection operator (LASSO) regression to select representative proteomic biomarkers for PRISm. Ten-fold cross-validation was then applied to determine the LASSO penalty parameter ‘lambda’. To enhance the robustness of biomarker selection, we additionally implemented the best subset (BeSS) [35] and minimax concave penalty (MCP) methods [36] and compared their results with those of LASSO. The overall proteomic scores were calculated as the weighted sum of the proteins identified by LASSO, BeSS or MCP. Subsequently, we employed the “mediation” package implemented in the R software to explore the mediation proportions of each plasma protein and the overall proteomic score for the association between PRISm and new-onset HF, and estimated CIs via quasi-Bayesian Monte Carlo method with 2000 simulations [37,38]. For significant protein mediators, Kyoto Encyclopedia of Genes and Genomes (KEGG) enrichment analyses were utilized to investigate the most frequently utilized pathways covered by those proteins with the online software Hiplot (https://hiplot.com.cn/).

All statistical analyses were performed using SAS 9.4 (SAS Institute, Cary, NC, USA) and R software (4.3.3). All statistical tests were two-sided, and *P* value of < 0.05 was considered statistically significant. A Benjamini-Hochberg false discovery rate (*FDR*) of less than 0.05 in protein-related analyses was utilized to correct for multiple testing [39].

## Results

### Study population characteristics

A total of 32,202 participants (17,351 women [53.88 %], 14,851 men [46.12 %]) with baseline normal spirometry were included in the spirometric transition analysis. Those who underwent the normal-PRISm transition were more likely to smoke, have obesity, be less educated, have lower household income, and be deprived according to the Townsend deprivation index (Table 1). For the proteomics analysis, among 40,047 individuals (22,525 women [56.25 %], 17,522 men [43.75 %]) included, 34,689 (86.62 %) and 5,358 (13.38 %) participants had normal spirometry and PRISm at baseline, respectively. Compared to participants with normal spirometry, individuals with PRISm had elevated Townsend deprivation levels, higher BMI, worse smoking status and lower household income (Supplementary Table 1).

**Table 1:** Baseline characteristics of individuals with repeated spirometry.

	Total	Normal-normal	Normal-PRISm	Normal-obstructive
No. of participants	32,202	27,864	1323	3015
Male sex	14,851 (46.12)	12,881 (46.23)	559 (42.25)	1411 (46.80)
Age, y	55 (49–61)	55 (48–60)	54 (48–60)	58 (52–63)
Ethnicity				
White	31,247 (97.03)	27,110 (97.29)	1219 (92.14)	2918 (96.78)
Mixed	152 (0.47)	135 (0.48)	5 (0.38)	12 (0.40)
Asian or Asian British	289 (0.90)	189 (0.68)	74 (5.59)	26 (0.86)
Black or Black British	200 (0.62)	171 (0.61)	16 (1.21)	13 (0.43)
Other	235 (0.73)	188 (0.67)	8 (0.60)	39 (1.29)
Townsend score				
I (least deprived)	8034 (24.95)	7002 (25.13)	292 (22.07)	740 (24.54)
II	8128 (25.24)	7058 (25.33)	320 (24.19)	750 (24.88)
III	8074 (25.07)	7009 (25.15)	326 (24.64)	739 (24.51)
IV	7930 (24.63)	6761 (24.26)	385 (29.10)	784 (26.00)
Smoking status				
Never	20,063 (62.30)	17,461 (62.67)	845 (63.87)	1757 (58.28)
Previous	10,349 (32.14)	8966 (32.18)	355 (26.83)	1028 (34.10)
Current	1727 (5.36)	1381 (4.96)	123 (9.30)	223 (7.40)
Alcohol intake				
Never	1442 (4.48)	1186 (4.26)	89 (6.73)	167 (5.54)
Special occasions only	2607 (8.10)	2236 (8.02)	149 (11.26)	222 (7.36)
One to three times a month	3566 (11.07)	3105 (11.14)	158 (11.94)	303 (10.05)
Once or twice a week	8362 (25.97)	7299 (26.20)	363 (27.44)	700 (23.22)
Three or four times a week	9054 (28.12)	7902 (28.36)	325 (24.57)	827 (27.43)
Daily or almost daily	7161 (22.24)	6128 (21.99)	238 (17.99)	795 (26.37)
Body mass index				
less than 18.5	132 (0.41)	110 (0.39)	5 (0.38)	17 (0.56)
18.5 to 25	12,557 (38.99)	10,780 (38.69)	421 (31.82)	1356 (44.98)
25 to 30	13,881 (43.11)	12,064 (43.30)	576 (43.16)	1246 (41.33)
greater than 30	5620 (17.45)	4898 (17.58)	331 (24.64)	396 (13.13)
Physical activities, min				
0	348 (1.08)	299 (1.07)	17 (1.28)	32 (1.06)
0–600	4530 (14.07)	3879 (13.92)	222 (16.78)	429 (14.23)
600–3000	15,152 (47.05)	13,133 (47.13)	604 (45.65)	1415 (46.93)
greater than 3000	7757 (24.09)	6736 (24.17)	297 (22.45)	724 (24.01)
Education status				
College or University degree	15,139 (47.01)	13,165 (47.25)	574 (43.39)	1400 (46.43)
NVQ or HND or HNC or equivalent	1691 (5.25)	1463 (5.25)	67 (5.06)	161 (5.34)
Other professional qualifications	1499 (4.65)	1296 (4.65)	54 (4.08)	149 (4.94)
A levels/AS levels or equivalent	4311 (13.39)	3746 (13.44)	168 (12.70)	397 (13.17)
Ordinary levels/GCSEs or equivalent / CSEs or equivalent	7532 (23.39)	6501 (23.33)	358 (27.06)	673 (22.32)
None of the above	1946 (6.04)	1623 (5.82)	96 (7.26)	215 (7.44)
Income per year				
greater than £100,000	2405 (7.47)	2171 (7.79)	70 (5.29)	164 (5.44)
£52,000 to £100,000	8504 (26.41)	7465 (26.79)	334 (25.25)	705 (23.38)
£31,000 to £51,999	8990 (27.92)	7792 (27.96)	374 (28.27)	824 (27.33)
£18,000 to £30,999	6381 (19.82)	5408 (19.41)	291 (22.00)	682 (22.62)
less than £18,000	3193 (9.92)	2679 (9.61)	152 (11.49)	362 (12.01)
High density lipoprotein cholesterol				
Low	4423 (13.74)	3803 (13.65)	256 (19.35)	364 (12.07)
High	23,376 (72.59)	20,256 (72.70)	893 (67.50)	2227 (73.86)
Triglycerides				
Low	19,815 (61.53)	17,194 (61.71)	765 (57.82)	1865 (61.56)
High	10,715 (33.27)	9221 (33.09)	492 (37.19)	1002 (33.23)
Hypertension	11,897 (36.94)	10,216 (36.66)	501 (37.87)	1180 (39.14)
Glycosylated hemoglobin	472 (1.47)	392 (1.41)	32 (2.42)	48 (1.59)
Glucose-lowering drugs	507 (1.57)	427 (1.53)	40 (3.02)	40 (1.33)
Lipid-lowering drugs	3553 (11.03)	3022 (10.85)	172 (13.00)	359 (11.91)
FVC, % predicted wave 1	100.44 (92.75–108.86)	100.86 (93.26–109.17)	89.24 (84.69–95.00)	101.54 (93.99–110.32)
FEV1, % predicted wave 1	99.32 (91.59–107.77)	100.03 (92.56–108.29)	86.76 (83.15–92.58)	97.81 (90.00–107.15)
FEV1 to FVC ratio wave 1	0.78 (0.75–0.81)	0.78 (0.76–0.81)	0.78 (0.75–0.81)	0.75 (0.72–0.79)
FVC, % predicted wave 2	92.11 (84.54–100.31)	92.70 (85.67–100.55)	71.07 (66.82–75.17)	92.62 (84.16–102.05)
FEV1, % predicted wave 2	89.65 (81.46–98.04)	91.37 (84.20–99.23)	68.80 (65.34–71.67)	75.57 (65.59–84.62)
FEV1 to FVC ratio wave 2	0.77 (0.74–0.80)	0.78 (0.75–0.81)	0.76 (0.73–0.79)	0.66 (0.59–0.69)
Follow-up time (baseline to repeated spirometry), y	9.18 (7.60–10.29)	9.14 (7.56–10.25)	9.43 (7.93–10.55)	9.48 (7.85–10.52)
Follow-up time (repeated spirometry to outcome), y	4.55 (3.69–6.04)	4.59 (3.72–6.10)	4.30 (3.47–5.70)	4.27 (3.56–5.70)

Data were presented as frequency (%) or median (P_25_-P_75_).

Abbreviations: A levels, Advanced Levels; AS levels, Advanced Subsidiary Levels; CSE, Certificate of Secondary Education; FEV1, forced expiratory volume in one second; FVC, forced vital capacity; GCSE, General Certificate of Secondary Education; HNC, Higher National Certificate; HND, Higher National Diploma; NVQ, National Vocational Qualification; PRISm, preserved ratio impaired spirometry; y, year.

### Associations between transitions of normal spirometry and the risk of HF

At the time point of repeated spirometry, a total of 27,864 (86.53 %) individuals with baseline normal spirometry retained normal spirometry, while 1,323 (4.11 %) participants underwent the transition to PRISm, and 3,015 (9.36 %) individuals developed obstructive spirometry. Over a median follow-up of 4.55 years, incident HF occurred in 210 of 27,864 individuals with the normal-normal transition, 19 of 1,323 individuals with the normal-PRISm transition, and 45 of 3,015 individuals with the normal-obstructive transition, respectively. Compared with the normal-normal transition, the normal-PRISm transition demonstrated a higher risk of HF, with adjusted HR (95 % CI) of 1.814 (1.128–2.919). Detailed results are shown in Table 2.

**Table 2:** Associations of transition trajectories of normal spirometry with incident heart failure.

	N_case_/N_total_	Unadjusted	Adjusted for age and sex	Fully adjusted a
		HR (95 % CI)	HR (95 % CI)	HR (95 % CI)
Normal to Normal	210/27,864	REF	REF	REF
Normal to PRISm	19/1323	2.060 (1.288, 3.294)	2.249 (1.406, 3.598)	1.814 (1.128, 2.919)
Normal to Obstructive	45/3015	2.132 (1.544, 2.942)	1.624 (1.174, 2.245)	1.719 (1.240, 2.383)

Abbreviation: HR, hazard ratio; PRISm, preserved ratio impaired spirometry.

a Adjusted for age, sex, ethnicity, Townsend deprivation index, smoking status, alcohol intake, physical activity, body mass index, household income, education status, hypertension, high density lipoprotein, triglycerides, glycosylated hemoglobin, glucose-lowering drugs, lipid-lowering drugs, coronary heart disease at baseline and stroke at baseline.

We observed similar prospective associations between transitions of normal spirometry and new-onset HF within several sensitivity analyses, including the exclusion of early incident HF cases occurring within the first two years of follow-up (Supplementary Table 2), using LLN definition for FEV1/FVC (Supplementary Table 3) and considering the competing risk (Supplementary Table 4). Moreover, results were generally consistent in stratified subgroup analyses (Supplementary Table 5).

### Causal effect of PRISm on heart failure

In the present study, according to a less stringent selection criteria of IVs (*P* < 5 × 10^-6^), we obtained 32 SNPs from GWAS data of PRISm. All selected IVs had F-statistics exceeding 10, reducing the likelihood of weak IV bias. However, Cochran’s Q test showed potential heterogeneity in the causal link between PRISm and HF (*P* < 0.05). MR-PRESSO analysis identified a significant outlier (rs4921274) among the IVs associated with PRISm. Details of selected SNPs were listed in Supplementary Table 6. After removal of the outlier, the random-effects IVW results showed a consistent causal link between PRISm and HF (odds ratio: 1.488, 95 % CI: 1.040–2.130, *P* = 0.030). The MR-RAPS method (OR: 1.446, 95 % CI: 1.019–2.051, *P* = 0.039) yielded consistent results with the random-effects IVW, whereas the weighted median, weighted mode, and MR-Egger method did not reach statistical significance. Detailed results of the MR analysis can be found in Table 3 and Supplementary Figs. 2–3. In addition, we observed the causal effect of chronic obstructive pulmonary disease on HF in our additional MR analysis (fixed-effects IVW: OR: 1.096, 95 % CI: 1.069–1.124, *P* < 0.001) (Supplementary Table 7).

**Table 3:** MR results for the causal relationship between PRISm and HF.

Exposure	Outcome	MR analysis	Method	nSNP	β	SE	OR (95 % CI)	*P*
PRISm	HF	Raw data	IVW (fixed effects)	32	0.505	0.132	1.657 (1.280–2.145)	<0.001
			IVW (random effects)	32	0.505	0.202	1.657 (1.116–2.459)	0.012
			MR Egger	32	0.457	0.565	1.579 (0.521–4.785)	0.425
			Weighted median	32	0.397	0.210	1.488 (0.986–2.244)	0.058
			Weighted mode	32	0.591	0.352	1.806 (0.905–3.602)	0.104
			RAPS	32	0.419	0.191	1.520 (1.045–2.210)	0.028
PRISm	HF	Outlier removed	IVW (fixed effects)	31	0.398	0.134	1.488 (1.144–1.937)	0.003
			IVW (random effects)	31	0.398	0.183	1.488 (1.040–2.130)	0.030
			MR Egger	31	0.373	0.504	1.451 (0.540–3.901)	0.466
			Weighted median	31	0.365	0.209	1.440 (0.956–2.169)	0.081
			Weighted mode	31	0.615	0.388	1.849 (0.865–3.953)	0.123
			RAPS	31	0.369	0.179	1.446 (1.019–2.051)	0.039

Abbreviation: HF, heart failure; IVW, inverse variance weighted; MR, Mendelian randomization; nSNP, number of single nucleotide polymorphisms; OR, odds ratio; PRISm, preserved ratio impaired spirometry.

Comprehensive sensitivity analyses were undertaken to ensure the reliability of the derived results. Firstly, Cochran’s Q tests indicated potential heterogeneity (*P* < 0.05). After removal of potential outliers, a reanalysis continued to indicate evidence of heterogeneity (Supplementary Table 8 and Fig. 4–5). Subsequently, the MR-Egger intercept exhibited no indication of directional pleiotropy, and the MR-PRESSO tests suggested limited impact of residual pleiotropy in MR analysis results between PRISm and HF (Supplementary Tables 9–10). Moreover, the results of the LOO analysis revealed no influential SNPs driving the overall estimates, thereby affirming the reliability of the results (Supplementary Figs. 6–7). Steiger tests supported a causal direction from PRISm to HF, while no evidence was found for a causal effect of HF on PRISm (Supplementary Table 11). At last, under conservative assumptions, the estimated overlap bias was lower than 0.013 and the type I error rate was at most 0.07, indicating that sample overlap is unlikely to materially affect our estimates.

### Bayesian colocalization analysis for PRISm and HF

Colocalization analysis was conducted to estimate the PP for a shared causal variant. There was limited evidence for a single shared causal variant across all genetic regions centered on the MR instruments, with PP.H4 values up to 22.99 % (Supplementary Table 12). In contrast, the probabilities for distinct causal variants predominated, with PP.H3 ranging from 2.11 % to 88.49 %. Taken together, the colocalization results indicate that PRISm and HF are unlikely to share the same causal variant at the tested genomic regions.

### Mediating effects of plasma proteins on the association between PRISm and incident HF

Supplementary Table 13 shows the associations of baseline PRISm with plasma proteins. After correction for multiple covariates, 1,023 proteins showed positive associations with baseline PRISm (*FDR* < 0.05), with regression coefficients (95 % CIs) ranging from 0.033 (0.020, 0.046) to 0.235 (0.219, 0.251). Furthermore, 76 proteins were negatively associated with PRISm, with regression coefficients (95 % CIs) ranging from −0.165 (−0.181, −0.149) to −0.029 (−0.039 to −0.018). Among them, insulin-like growth factor-binding protein 2 (IGFBP2), N-alpha-acetyltransferase 10 (NAA10) and beta-crystallin B1 (CRYBB1) were the top proteins that showed positive strong associations with PRISm, while proteins such as Golgi reassembly-stacking protein 2 (GORASP2), apolipoprotein A-II (APOA2), disintegrin and metalloproteinase domain-containing protein 12 (ADAM12) were inversely associated with baseline PRISm.

Simultaneously, 859 and 90 proteins were found to be positively and negatively associated with incident HF (*FDR* < 0.05), with HRs (95 % CIs) ranging from 1.062 (1.013, 1.114) to 1.798 (1.710, 1.891) and 0.781 (0.738, 0.828) to 0.928 (0.874–0.986) (Supplementary Table 14), respectively. For instance, fatty acid-binding protein, adipocyte (FABP4), adenosine deaminase (ADA) and exostosin-like 1 (EXTL1) were the top-ranked proteins that were positively associated with incident HF. By contrast, interleukin-5 (IL5), angiopoietin-2 (ANGPT2) and UV excision repair protein RAD23 homolog B (RAD23B) showed significant inverse associations with the risk of HF.

**Fig. 2:**
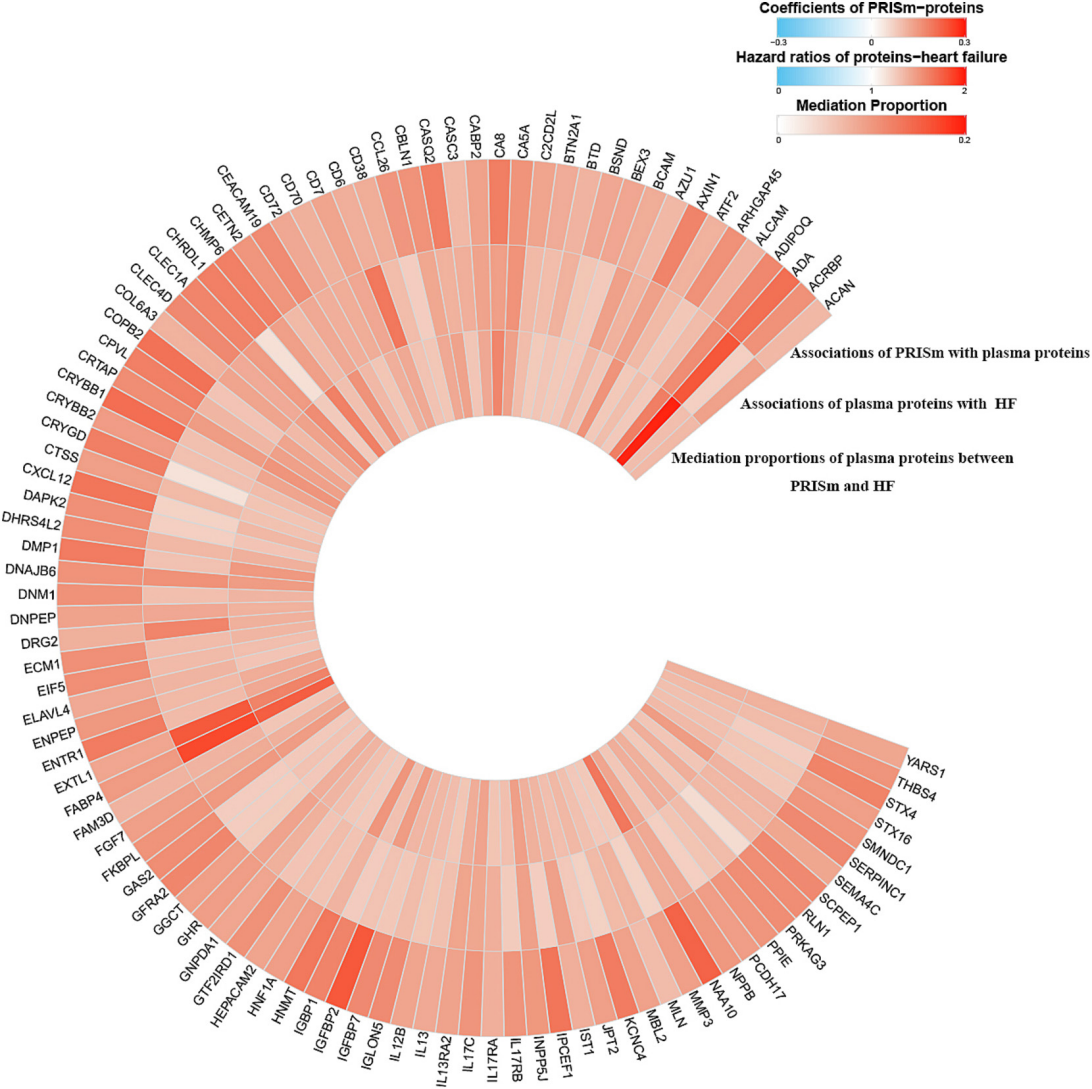
Associations between PRISm and plasma proteins, associations between plasma proteins and the risk of heart failure, and mediation proportions of the top 100 plasma proteins between PRISm and the risk of heart failure. Linear regression models and Cox proportional hazards models were adjusted for age, sex, ethnicity, Townsend deprivation index, household income, smoking status, alcohol intake, physical activity, body mass index, education status, hypertension, high density lipoprotein, triglycerides, glycosylated hemoglobin, glucose-lowering drugs, lipid-lowering drugs, coronary heart disease at baseline and stroke at baseline. *: False discovery rate < 0.05.

A total of 672 plasma proteins were significantly associated with both baseline PRISm and incident HF. 653 mediators demonstrated positive mediation effects, with mediation proportions (95 % CIs) ranging from 0.34 % (0.01 % to 0.94 %) to 18.50 % (14.37 % to 25.12 %) (Supplementary Table 15). Among them, ADA, FABP4, NAA10, charged multivesicular body protein 6 (CHMP6) and adiponectin (ADIPOQ) were identified to be the top 5-ranked mediators (Fig. 2). Moreover, the overall proteomic score demonstrated a considerable mediation proportion (95 % CI) of 59.63 % (46.98 % to 77.12 %) by LASSO. BeSS and MCP methods yielded mediation proportions of 51.96 % (95 % CI: 41.05 % to 68.61 %) and 54.21 % (42.40 % to 70.04 %), respectively. Using KEGG methods, a total of 33 related KEGG pathways were identified at the criteria of *FDR* < 0.05, among which the top 5 enriched pathways were cytokine-cytokine receptor interaction, JAK-STAT signaling pathway, viral protein interaction with cytokine and cytokine receptor, hematopoietic cell lineage and IL-17 signaling pathway. These significant pathways were mainly related to immune and inflammatory responses (cytokine-cytokine receptor interaction and viral protein interaction with cytokine and cytokine receptor) and signal transduction (JAK-STAT signaling pathway and IL-17 signaling pathway) (Supplementary Table 16).

## Discussion

In this study, we found that the normal-PRISm transition was associated with an elevated risk of HF. Furthermore, we provided evidence for the causal relationship between PRISm and HF, and identified protein biomarkers that considerably mediated the relationship between PRISm and HF.

In recent years, several studies have investigated the prospective association between baseline PRISm and the risk of HF [9,10]. For example, using data from the UK Biobank, Zheng [9] showed that PRISm at baseline was associated with an increased risk of HF (HR: 1.88, 95 % CI:1.72–2.05). In addition to baseline PRISm, emerging studies have revealed that the normal-PRISm transition is an independent predictor of subsequent cardiovascular outcomes [9,12,14]. For instance, Wijnant [12] reported that individuals with the normal-PRISm transition were at an increased risk of cardiovascular mortality. To the best of our knowledge, Marott [14] firstly revealed that compared with individuals with consistent normal spirometry, individuals with the normal-PRISm transition, which included 24 incident HF or ischemic heart disease (IHD) cases, were at a higher risk of HF or IHD (HR: 1.91, 95 % CI 1.24–2.95). However, this study recruited a total of 1,160 participants aged 20–40 years and only adjusted for age and sex. Our analysis of 32,202 participants revealed a robust and strong association of the normal-PRISm transition with incident HF among middle-aged and elderly adults. Since PRISm can be measured and monitored during routine spirometry measurements [6,8], obviating the need for any additional tests, our current findings highlight the added value of repeated spirometry in identifying individuals at elevated risk and taking intensive care, thereby facilitating the primary prevention of incident HF to the greatest extent possible.

Epidemiological evidence from both prior studies [9,10] and our current study strongly supports the conclusion that PRISm is an independent risk factor for HF. However, observational studies only confirmed the prospective associations of PRISm and the risk of HF but lacked reliable evidence of causality. Considering that MR analysis is less prone to confounding and reverse causality than observational epidemiologic studies, we conducted the first MR analysis to explore the causality on the association between PRISm and HF. Consistent findings across several sensitivity analyses, including the MR-RAPS, MR-PRESSO, MR-Egger intercept and LOO tests, confirmed the robustness of our findings. However, due to lack of statistical power, the weighted median, weighted mode and MR-Egger method yielded directionally concordant but non-significant estimates. Taken together, this causal link underscores the value of preventive strategies or interventions targeting PRISm to reduce the risk of HF.

Despite the causal relation between PRISm and HF, the underlying biological mechanisms have yet to be uncovered. Our proteomics analysis further identified a range of plasma proteins associated with both baseline PRISm and incident HF, and many of these proteins also played significant mediation roles in the causal relationship between PRISm and HF. These circulating proteins are primarily involved in such pathways as cytokine-cytokine receptor interaction, viral protein interaction with cytokine and cytokine receptor and JAK-STAT pathway. The development of HF depends on great equilibrations among compensatory hypertrophy and cardiomyocyte apoptosis, angiogenesis and fibrosis [40]. The proinflammatory cytokine hypothesis suggested that elevated inflammatory mediators trigger cardiac inflammation, subsequently leading to left ventricular remodeling and dysfunction [41]. Given prior evidence of altered endocrine and cytokine profiles in individuals with PRISm [17], PRISm may promote HF by regulating inflammation-related cytokine expression. Additionally, the JAK-STAT signaling pathway centrally regulates cellular functions and mediates the biological functions of over 50 cytokines and growth factors [42]. Evidence has shown that the JAK-STAT pathway poses an immediate effect on myocardial hypertrophy, and promotes cardiac reshaping via angiogenesis and fibrosis [43]. Given the established associations between PRISm and activation of JAK/STAT pathway related signaling proteins, including the gp130, IL-2, and IL-3, IL-5 GM-CSF families [44], this pathway may mediate the causal link between PRISm and HF. Simultaneously, many top-ranked mediators identified in our proteomics analysis played pivotal roles in these pathways. Prior studies reported that the decreased enzyme activity of the ADA attenuates pathologic consequences of HF by regulating the concentration of adenosine [45,46]. A prospective cohort study also reported the association between FABP4 and the risk of HF, and raised the possibility that FABP4 plays a causal role in the pathogenesis of HF [47]. Given the robust mediating role exhibited by FABP4, we hypothesize that increased FABP4 expression in the PRISm population poses an immediate effect on development of HF. Altogether, our proteomics findings identified several pathways and protein biomarkers that elucidate the causal relationship between PRISm and HF, setting the stage for the targeted preventive and therapeutic strategies in the future.

This present study has notable strengths. The UK Biobank study represents one of the largest prospective cohorts worldwide that enrolled participants aged 37 to 73 years, enabling a large and reliable investigation of the associations between transitions of PRISm and incident HF. Moreover, we firstly investigated the causal relationship between PRISm and HF and are also the first one to explore their underlying proteomic mediators. Other strengths of the present study include a prospective study design and professional spirometry. However, several limitations should also be noted. First, since the UK Biobank cohort and GWAS data consist largely of individuals of the European ancestry, the generalizability of our findings to other populations warrants further validation. Second, the spirometric transition analysis was conducted with spirometries at two time points, and the average follow-up time was limited to about 5 years; consequently, further studies with more frequent spirometric assessments and extended follow-up periods are needed. Third, future MR analyses with access to additional independent exposure or outcome GWAS datasets are needed to validate our findings. Fourth, our proteomic results are exploratory in nature, and validation of these findings in other cohorts and populations is warranted. Last, due to data availability, more than half of the UK Biobank participants were excluded in both the spirometric transition analysis and the proteomics analysis. However, the differences in the baseline characteristics of the included and excluded population were not large from a clinical perspective (Supplementary Table 17), and all potential confounders were accounted for in the multivariable analyses.

## Conclusion

In summary, the present study revealed that individuals with baseline normal spirometry who subsequently developed PRISm exhibited an increased risk of HF, supporting that continuously screening and monitoring PRISm could have far-reaching benefits for the prevention of HF in individuals with normal spirometry. In addition, the MR analysis provided evidence for the causal link between PRISm and HF, and the proteomics analysis further identified a range of plasma proteins that mediated the relationship between PRISm and HF. These findings provide genetic evidence supporting the causal role of PRISm in the development of HF and shed new mechanistic insights into the molecular foundations, thus providing targets for HF intervention in individuals with PRISm.

## Compliance with ethics requirements

All procedures followed were in accordance with the ethical standards of the responsible committee on human experimentation (institutional and national) and with the Helsinki Declaration of 1975, as revised in 2008 (5). Informed consent was obtained from all patients for being included in the study.

The investigation conforms with the principles outlined in the Declaration of Helsinki. The UK Biobank study was approved by the Northwest Multi-Centre Research Ethics Committee (REC reference: 21/NW/0157), and all participants signed informed consent forms. Participants gave informed consent to participate in the study before taking part.

## Financial/nonfinancial disclosures

This work was supported by the National Natural Science Foundation of China (81703316) and A Project Funded by Priority Academic Program Development of Jiangsu Higher Education Institutions (PAPD).

## Declaration of competing interest

The authors declare that they have no known competing financial interests or personal relationships that could have appeared to influence the work reported in this paper.
