# Supplementary material for: Preserved ratio impaired spirometry, plasma proteomics, and incident heart failure
Source: J Adv Res. 2025 Oct 12;85:715–23. doi: 10.1016/j.jare.2025.10.009 (PMC13316358; doi:10.1016/j.jare.2025.10.009)
Supplement: Supplementary Data 1 [file mmc1.pdf]

Supplementary materials

Preserved ratio impaired spirometry, plasma proteomics, and incident heart failure

Supplementary materials

Supplementary Figure 1 Schematic diagram of the cohort study design and key time points

Supplementary Figure 2 Scatter plot for the potential impacts of SNPs on both PRISm and HF

Supplementary Figure 3 Scatter plot for the potential impacts of SNPs on both PRISm and HF with outliers removed

Supplementary Figure 4 Funnel plot for the causal relationship between PRISm and HF

Supplementary Figure 5 Funnel plot for the causal relationship between PRISm and HF with outliers removed

Supplementary Figure 6 Forest plots of the leave-one-out analysis for the causal relationship between PRISm and HF

Supplementary Figure 7 Forest plots of the leave-one-out analysis for the causal relationship between PRISm and HF with outliers removed

Supplementary Method 1 Assessments for covariates

Supplementary Method 2 Two-sample Mendelian randomization

Supplementary Method 3 Colocalization analysis

Supplementary Table 1 Baseline characteristics of individuals with valid data on plasma proteins

Supplementary Table 2 Associations of transition trajectories of normal spirometry with incident HF after excluding morbidity cases that occurred within two years of follow up

Supplementary Table 3 Associations of transition trajectories of normal spirometry with incident HF using LLN definition for FEV1/FVC

Supplementary Table 4 Associations of transition trajectories of normal spirometry with incident HF (Fine-Gray model)

Supplementary Table 5 Subgroup analyses of associations between transitions of normal spirometry and incident HF

Supplementary Table 6 SNPs used as instruments and their association with the exposure and outcome

Supplementary Table 7 MR results for the causal relationship between COPD and HF

Supplementary Table 8 Measures of heterogeneity with Cochran's Q test

Supplementary Table 9 Examination of horizontal pleiotropy effects with MR-Egger regression tests

Supplementary Table 10 MR-PRESSO analysis for the causal relationship between PRISm and HF

Supplementary Table 11 Steiger directionality test for the causal relationship between PRISm and HF

Supplementary Table 12 Colocalization analysis of the association between PRISm and HF

Supplementary Table 13 Associations of PRISm with plasma proteins

Supplementary Table 14 Associations of plasma proteins with the risk of HF

Supplementary Table 15 Mediation effects of plasma proteins associated with both PRISm and the risk of HF

Supplementary Table 16 KEGG pathways enriched by the significant mediators between PRISm and HF

Supplementary Table 17 Baseline characteristics of the included and excluded population

Supplementary Table 18 SNPs excluded due to potential associations with confounders

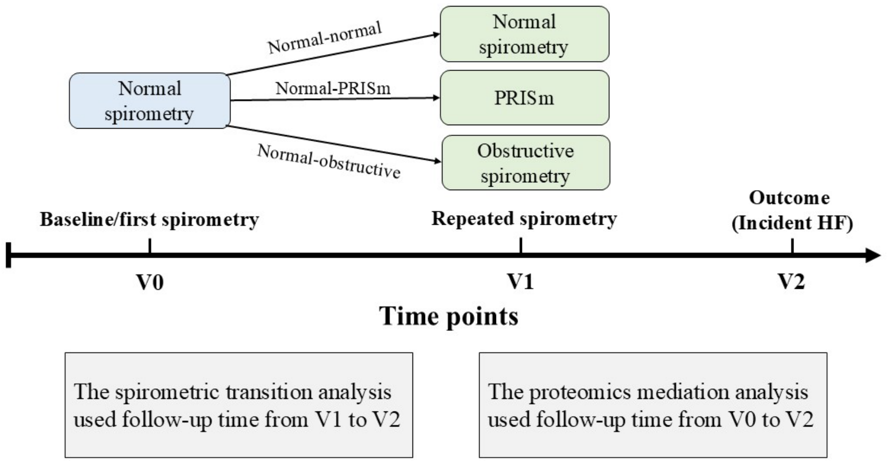

Supplementary Figure 1 Schematic diagram of the cohort study design and key time points

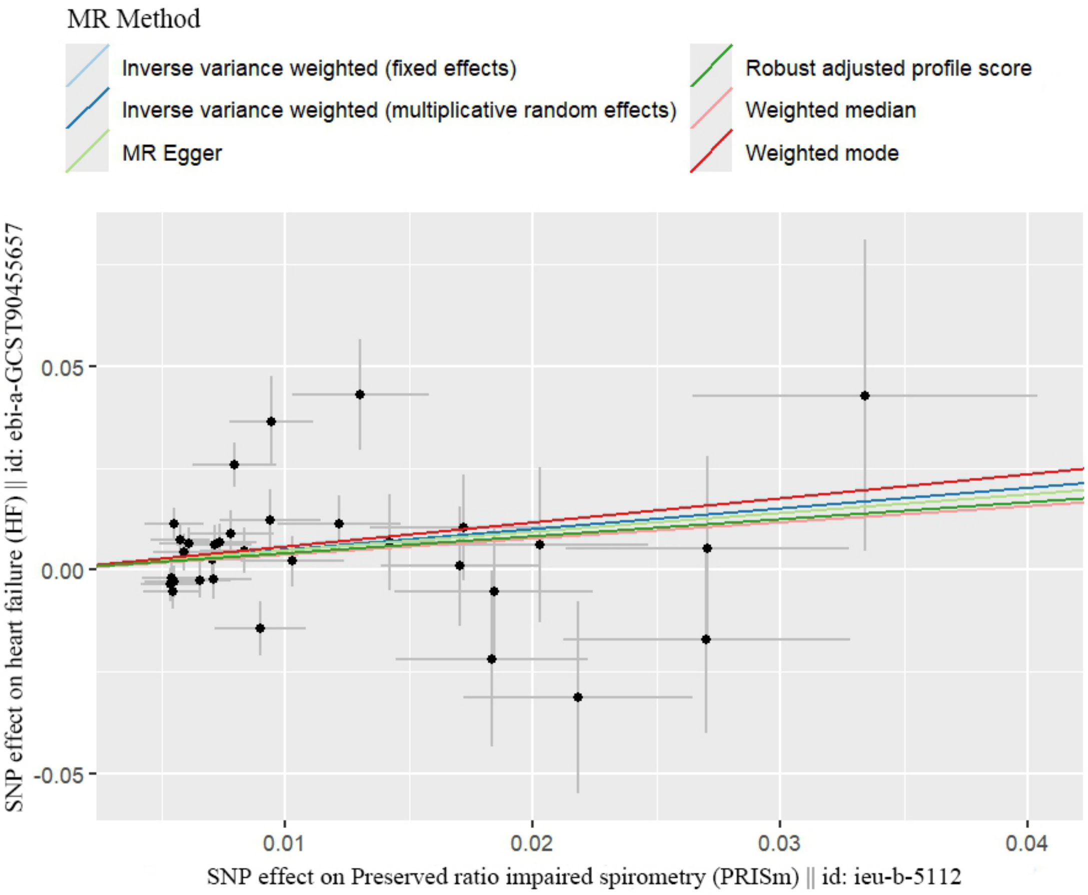

Supplementary Figure 2 Scatter plot for the potential impacts of SNPs on both PRISm and HF

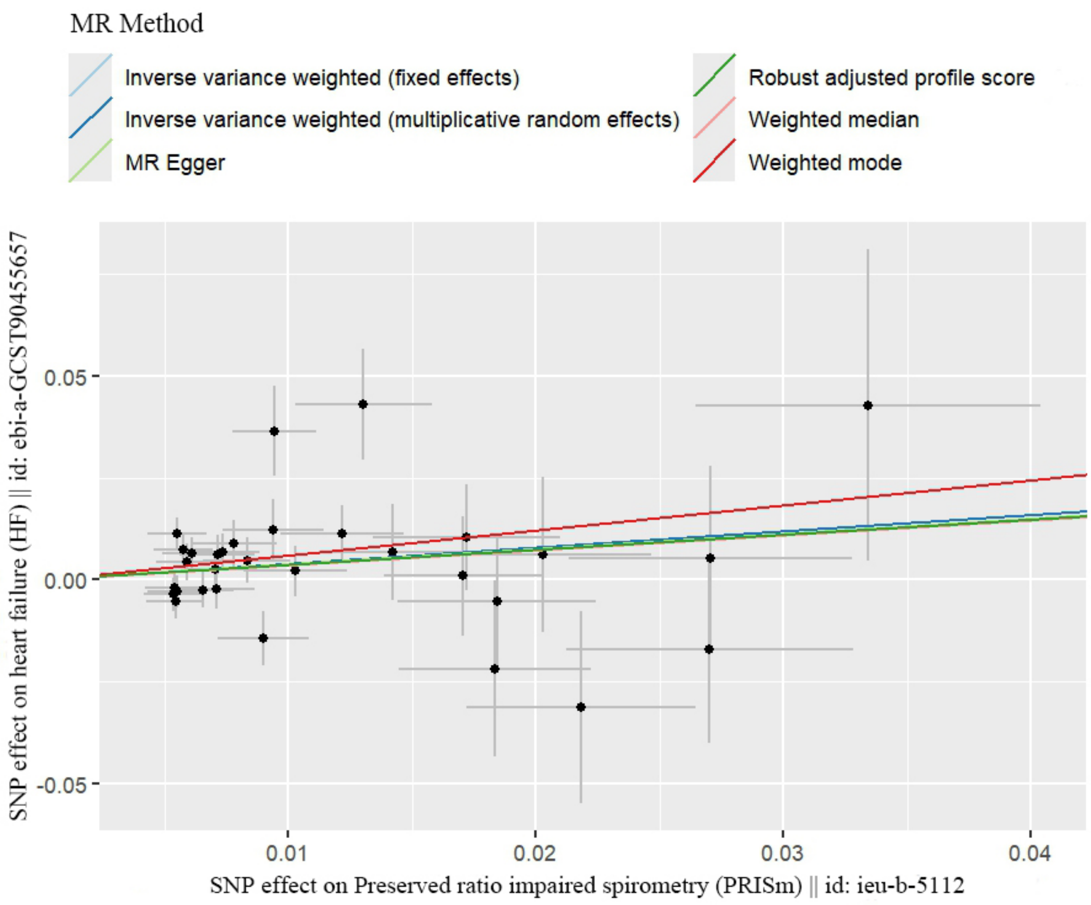

Supplementary Figure 3 Scatter plot for the potential impacts of SNPs on both PRISm and HF with outliers removed

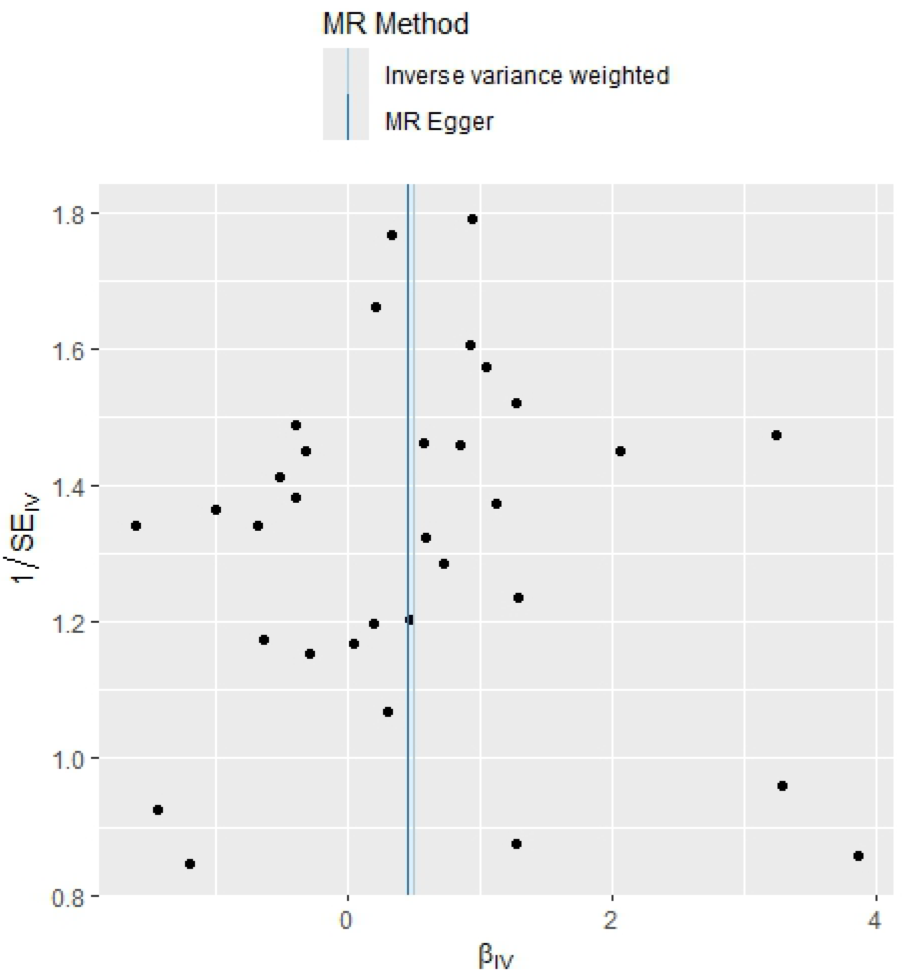

Supplementary Figure 4 Funnel plot for the causal relationship between PRISm and HF

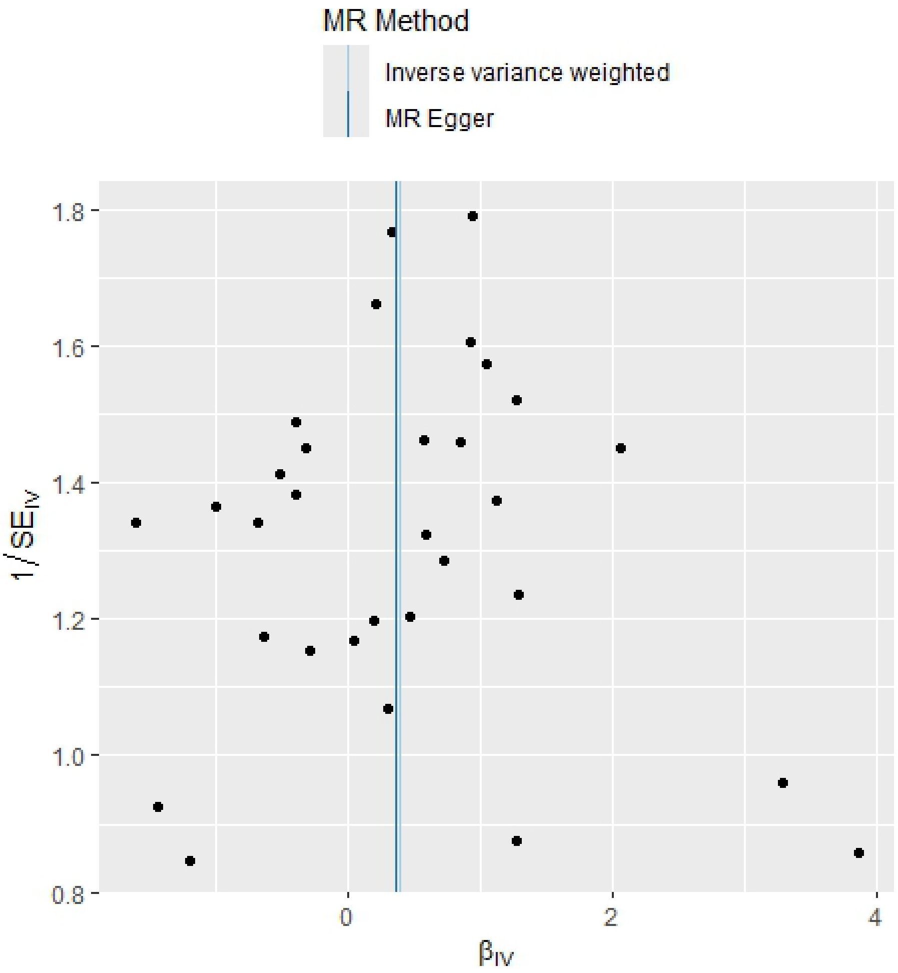

Supplementary Figure 5 Funnel plot for the causal relationship between PRISm and HF with outliers removed

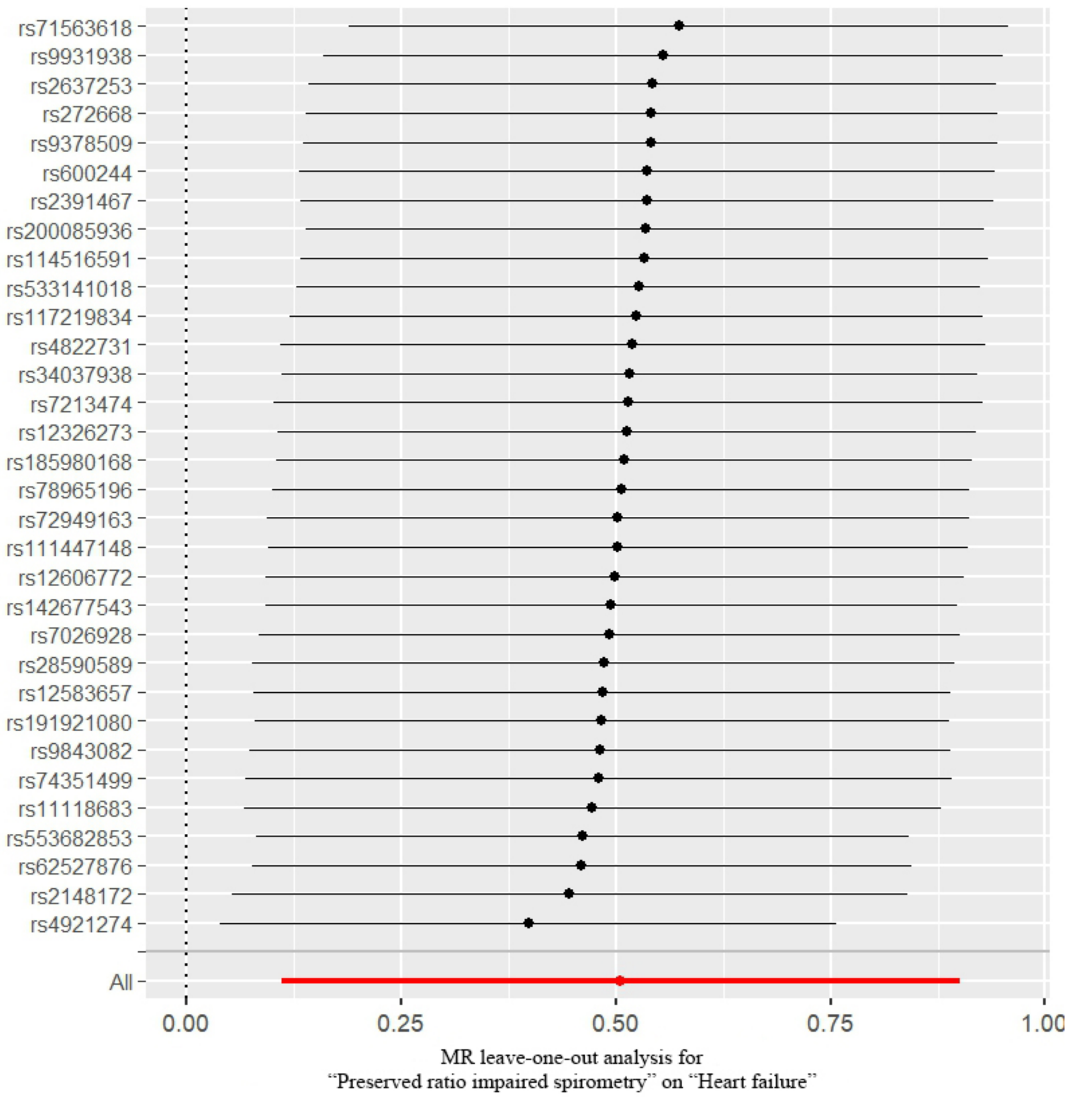

Supplementary Figure 6 Forest plots of the leave-one-out analysis for the causal relationship between PRISm and HF

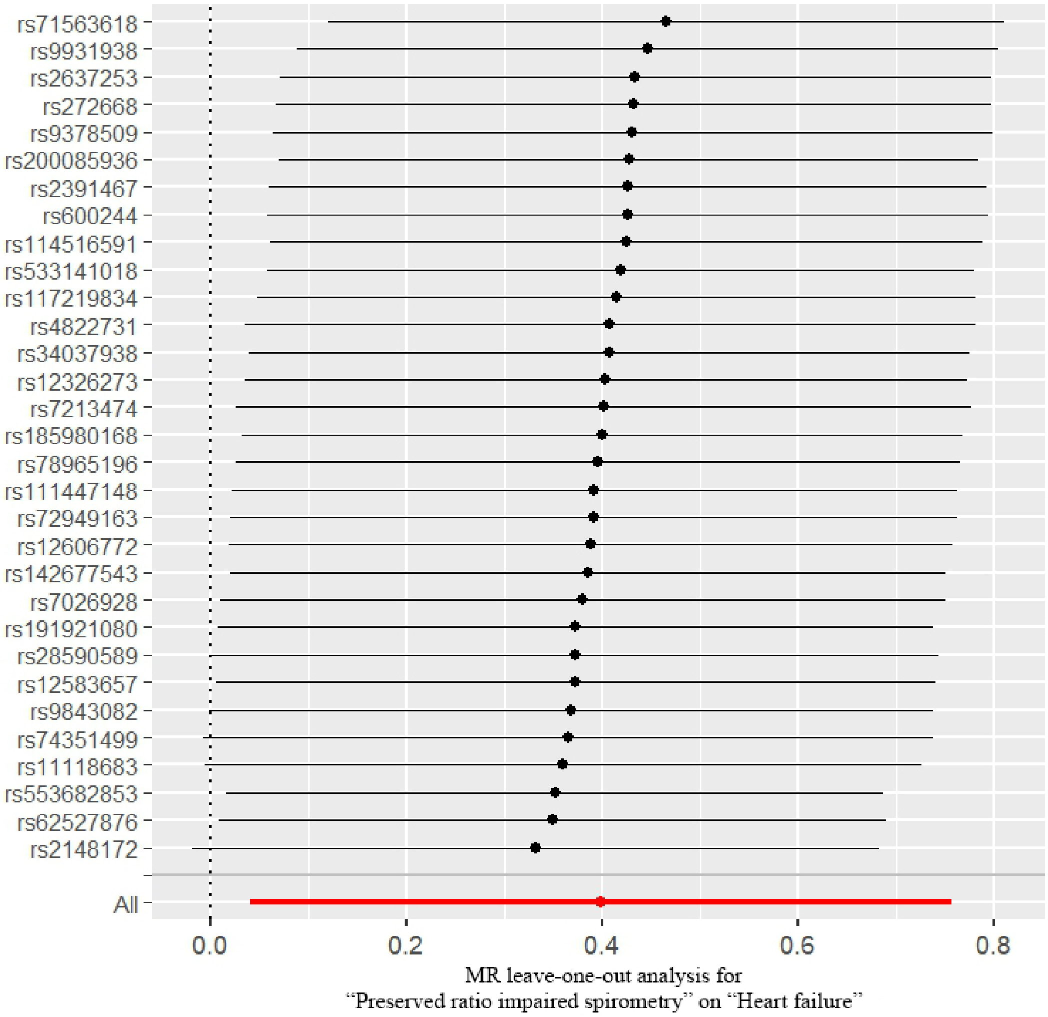

Supplementary Figure 7 Forest plots of the leave-one-out analysis for the causal relationship between PRISm and HF with outliers removed

Supplementary methods

Supplementary Method 1 Assessments for covariates

Sociodemographic covariates included age (continuous), sex (females/males), ethnicity (categorized as White, mixed, Asian/Asian British, Black/Black British, and others), Townsend deprivation index, household income and education status. Townsend deprivation index was derived from participants' postcodes and assigned a score based on socio-economic status through the quartiles of the indices [1]. We categorized household income into five groups from high to low (greater than £100,000, £52,000 to £100,000, £31,000 to £51,999, £18,000 to £30,999, less than £18,000). Education status was classified as College or University degree, National Vocational Qualification or Higher National Diploma or Higher National Certificate or equivalent, Other professional qualifications, Advanced levels/Advanced Subsidiary levels or equivalent, Ordinary levels/ General Certificates of Secondary Education or equivalent/ Certificate of Secondary Education or equivalent and None of the above. Lifestyle covariates encompassed body mass index (BMI), smoking status (never smoked, ever smoked, or currently smoking), alcohol intake (classified as never, special occasions only, one to three times a month, once or twice a week, three or four times a week, and daily or almost daily) and physical activity. Body mass index was calculated as weight (kg) divided by height squared (m^2) based on the recommendations of the World Health Organization (WHO): underweight (<18.5 kg/m^2), normal weight (18.5 to 25 kg/m^2), overweight (25 to 30 kg/m^2) and obesity (≥ 30 kg/m^2). Physical activity was categorized into four groups according to the total Metabolic Equivalent Task (MET) minutes for

all activities over the previous week [2]: no (MET=0), low (MET <600 min/week), moderate (600-3,000 MET) and high (≥3,000 MET). Hypertension was determined based on the combination of a self-reported history of hypertension, clinical blood pressure measurements (systolic blood pressure [SBP] ≥140 mm Hg or diastolic blood pressure [DBP] ≥90 mm Hg) or self-reported medication records. Biochemical indexes, including glycosylated hemoglobin (HbA1c), high density lipoprotein (HDL) and triglycerides were defined as secondary classified variables (normal or abnormal). We employed common clinical criteria to classify these biochemical indexes, which were 48 mmol/mol for HbA1c [3], 1.3 mmol/L (female) /1.0 mmol/L (male) for HDL [4] and 1.7 mmol/L for triglycerides [4]. Given the potential bias of medications, the current status of taking glucose-lowering drugs or lipid-lowering drugs was also included as covariates. Other covariates included coronary heart disease at baseline and stroke at baseline. We created a separate response category for participants with missing values and/or selection of unknown and/or preferring not to answer. Details of the above variables can be found on http://biobank.ndph.ox.ac.uk/showcase.

Supplementary Method 2 Two-sample Mendelian randomization

2.1 Data sources

The summary-level genome-wide association study (GWAS) data for individuals with PRISm were sourced from the IEU Open GWAS dataset (https://gwas.mrcieu.ac.uk/), which involved a study of 296,282 UK Biobank participants, comprising 38,639 cases and 257,643 controls (Study ID: ieu-b-5112) [5]. In addition, GWAS summary statistics for HF were retrieved from the GWAS Catalog (https://www.ebi.ac.uk/gwas/). This

dataset was obtained through a large-scale GWAS of 2,358,556 participants, predominantly of European ancestry (207,346 cases and 2,151,210 controls), and 40,637,482 single nucleotide polymorphisms (SNPs), based on nonoverlapping analyses of eight separate cohorts/consortia (HERMES, VA MVP, FinnGen, Mount Sinai BioMe, PMBB, eMERGE, Geisinger DiscovEHR and the Global Biobank Meta-analysis Initiative) (Study ID: ebi-a-GCST90455657) [6]. Since the Mendelian randomization (MR) study only utilized publicly available summary-level data, no additional ethical authorizations were required.

2.2 Instrumental variable (IV) selection

To ensure effective IVs, we first set a genome-wide significance threshold of $P < 5\times10^{-8}$ to identify SNPs that fulfill the three main assumptions of the MR analysis: (1) the IVs are robustly associated with PRISm; (2) the IVs influence risk of HF only through their effect on PRISm; and (3) the IVs are independent of potential confounders. Given the limited sample sizes in the GWAS of PRISm, the number of IVs reaching the conventional significance threshold of $P < 5\times10^{-8}$ was insufficient. To enhance the statistical power of the MR analysis and to minimize the risk of overlooking potential true associations, a less stringent threshold of 5×10^{-6} was employed to obtain more IVs that fulfilled the requirements and to preserve adequate statistical power [7]. Simultaneously, the linkage disequilibrium (LD) between SNPs was eliminated through clustering (LD clumping r^2=0.01 and kb=5,000) [8]. We excluded SNPs with incongruent alleles or intermediate allele frequencies to harmonize these exposure and outcome datasets. Considering the relatively relaxed threshold, the F-statistics for IVs

were calculated using the following formula: $F = (\beta/SE)^2$, where β represents the effect size and SE represents the standard error [9]. Selected IVs with F-statistics exceeding 10 indicate a lower probability of potential weak instrument bias [9]. All screened SNPs were utilized as IVs for subsequent MR analysis. Furthermore, to bolster the validity of our instrument selection, we excluded SNPs related to confounders ($P < 5\times10^{-8}$) using the PhenoScanner V2 (http://www.phenoscanner.medschl.cam.ac.uk/) tool, specifically confounders related to body composition, blood pressure, lipid metabolism, and systemic inflammation. Consequently, detailed information about the removed SNPs in this MR analysis was provided in **Supplementary Table 18**.

2.3 Statistical analysis

The inverse variance weighted (IVW) method was regarded as the main analysis [10]. Significant associations were identified with an IVW P value < 0.05 and consistent β-values across complementary methods. The IVW method combines the effects of multiple IVs by weighting them according to their inverse variance, thereby reducing the influence of less precise estimates and providing a more accurate overall causal estimate [10]. This method is particularly effective for estimating the unbiased causal effect when all IVs are valid and horizontal pleiotropy is absent. In the present study, both fixed and random effects IVW models are available. When significant heterogeneity is detected by the Cochran's Q statistics ($P < 0.05$), a random-effects IVW model is used. In contrast, the MR Egger regression allows for the presence of directional horizontal pleiotropy in IVs included in the analysis, although this method generally has lower statistical power [11]. The weighted median approach yields a

consistent causal estimation when only half of the instruments used in the MR analysis are valid [12]. The weighted mode method produces a reliable causal relation if the largest cluster of instruments with similar effect estimates consists of valid IVs, thus offering robustness against violations in a subset of instruments [13]. Moreover, the robust adjusted profile score (RAPS) method accommodates many weak instruments and idiosyncratic pleiotropy, thereby providing more reliable causal estimates in settings where conventional approaches may be biased [14]. The MR pleiotropy residual sum and outlier (MR-PRESSO) analysis identifies and attempts to correct for pleiotropy by excluding significant outliers. However, the MR-PRESSO outlier test necessitates that at least 50% of the genetic variants are valid instruments and is dependent on the InSIDE assumption [15]. As PRISm is considered a preclinical stage of chronic obstructive pulmonary disease (COPD) [16-18], we conducted an additional MR analysis to investigate the causal effect of COPD on HF. Summary-level COPD GWAS data were obtained from the IEU Open GWAS repository (https://gwas.mrcieu.ac.uk/), comprising 13,530 cases and 454,945 controls (Study ID: ebi-a-GCST90018807) [19]. All aforementioned results were evaluated by odds ratios (ORs) with 95% CIs.

Supplementary Method 3 Colocalization analysis

The colocalization analysis was performed to determine whether the two phenotypes share a common causal variant within a specific genomic region. For each IV identified in the MR analysis, regions that had 500 kb upstream and downstream were selected for colocalization. Five mutually exclusive hypotheses were tested: (1) no causal

genetic variant for either trait (H0); (2) there is one causal variant for PRISm only (H1); (3) there is one causal variant for HF only (H2); (4) there are two distinct causal genetic variants, one for each trait (H3); and (5) there is a shared causal genetic variant for both traits (H4). Posterior probabilities (PP) were calculated to quantify support for each hypothesis, which were expressed as $PP.H_0$, $PP.H_1$, $PP.H_2$, $PP.H_3$, and $PP.H_4$ [20]. The average PP.H4 value across all regions was regarded as the overall colocalization estimate. We set the prior probabilities at 1×10^{-4} for association with trait 1 only (p1), 1×10^{-4} for association with trait 2 only (p2), and 1×10^{-5} for association with both traits (p3). A PP.H4 level greater than 75% was considered suggestive of evidence for a shared causal variant for both traits. All PPs were calculated based on the "coloc" package in R version 4.3.3.

References

1. Jarman B, Townsend P, Carstairs V. Deprivation indices. *BMJ*. 1991;303(6801):523.
2. Group TI. IPAQ scoring protocol - International Physical Activity Questionnaire [Available from: https://sites.google.com/site/theipaq/scoring-protocol.
3. Diagnosis and classification of diabetes mellitus. *Diabetes Care*. 2011;34 Suppl 1(Suppl 1):S62-9.
4. Alberti KG, Eckel RH, Grundy SM, Zimmet PZ, Cleeman JI, Donato KA, et al. Harmonizing the metabolic syndrome: a joint interim statement of the International Diabetes Federation Task Force on Epidemiology and Prevention; National Heart, Lung, and Blood Institute; American Heart Association; World Heart Federation; International Atherosclerosis Society; and International Association for the Study of Obesity. *Circulation*. 2009;120(16):1640-5.
5. Higbee DH, Lirio A, Hamilton F, Granell R, Wyss AB, London SJ, et al. Genome-wide association study of preserved ratio impaired spirometry (PRISm). *Eur Respir J*. 2024;63(1):2300337.
6. Lee DSM, Cardone KM, Zhang DY, Tsao NL, Abramowitz S, Sharma P, et al. Common-variant and rare-variant genetic architecture of heart failure across the allele-frequency spectrum. *Nat Genet*. 2025;57(4):829-38.
7. Cao J, Wang Z, Zhu M, Huang Y, Jin Z, Xiong Z. Low-density lipoprotein cholesterol and risk of hepatocellular carcinoma: a Mendelian randomization

and mediation analysis. *Lipids Health Dis*. 2023;22(1):110.

8. Clarke L, Zheng-Bradley X, Smith R, Kulesha E, Xiao C, Toneva I, et al. The 1000 Genomes Project: data management and community access. *Nat Methods*. 2012;9(5):459-62.

9. Burgess S, Thompson SG. Avoiding bias from weak instruments in Mendelian randomization studies. *Int J Epidemiol*. 2011;40(3):755-64.

10. Zuber V, Colijn JM, Klaver C, Burgess S. Selecting likely causal risk factors from high-throughput experiments using multivariable Mendelian randomization. *Nat Commun*. 2020;11(1):29.

11. Bowden J, Davey Smith G, Burgess S. Mendelian randomization with invalid instruments: effect estimation and bias detection through Egger regression. *Int J Epidemiol*. 2015;44(2):512-25.

12. Bowden J, Davey Smith G, Haycock PC, Burgess S. Consistent Estimation in Mendelian Randomization with Some Invalid Instruments Using a Weighted Median Estimator. *Genet Epidemiol*. 2016;40(4):304-14.

13. Hartwig FP, Davey Smith G, Bowden J. Robust inference in summary data Mendelian randomization via the zero modal pleiotropy assumption. *Int J Epidemiol*. 2017;46(6):1985-98.

14. Zhao Q, Wang J, Hemani G, Bowden J, Small DS. Statistical inference in two-sample summary-data Mendelian randomization using robust adjusted profile score. *Annals of Statistics*. 2020;48(3):1742-69.

15. Verbanck M, Chen CY, Neale B, Do R. Detection of widespread horizontal

pleiotropy in causal relationships inferred from Mendelian randomization between complex traits and diseases. *Nat Genet*. 2018;50(5):693-8.

16. Wan ES, Fortis S, Regan EA, Hokanson J, Han MK, Casaburi R, et al. Longitudinal Phenotypes and Mortality in Preserved Ratio Impaired Spirometry in the COPDGene Study. *Am J Respir Crit Care Med*. 2018;198(11):1397-405.

17. Wijnant SRA, De Roos E, Kavousi M, Stricker BH, Terzikhan N, Lahousse L, Brusselle GG. Trajectory and mortality of preserved ratio impaired spirometry: the Rotterdam Study. *Eur Respir J*. 2020;55(1):1901217.

18. Wan ES, Hokanson JE, Regan EA, Young KA, Make BJ, DeMeo DL, et al. Significant Spirometric Transitions and Preserved Ratio Impaired Spirometry Among Ever Smokers. *Chest*. 2022;161(3):651-61.

19. Sakaue S, Kanai M, Tanigawa Y, Karjalainen J, Kurki M, Koshiba S, et al. A cross-population atlas of genetic associations for 220 human phenotypes. *Nat Genet*. 2021;53(10):1415-24.

20. Giambartolomei C, Vukcevic D, Schadt EE, Franke L, Hingorani AD, Wallace C, Plagnol V. Bayesian test for colocalisation between pairs of genetic association studies using summary statistics. *PLoS Genet*. 2014;10(5):e1004383.

Supplementary Table 1 Baseline characteristics of individuals with valid data on plasma proteins

	Total	Normal	PRISm
No. of participants	40,047	34,689	5358
Male sex	17,522 (43.75)	15,149 (43.67)	2403 (44.85)
Age, y	57 (49-63)	57 (49-63)	58 (50-63)
Ethnicity			
White	37,327 (93.21)	32,693 (94.25)	4634 (86.49)
Mixed	277 (0.69)	248 (0.71)	29 (0.54)
Asian or Asian British	742 (1.85)	466 (1.34)	276 (5.15)
Black or Black British	920 (2.30)	662 (1.91)	258 (4.82)
Other	590 (1.47)	463 (1.33)	127 (2.37)
Townsend score			
I (least deprived)	9770 (24.40)	8659 (24.96)	1111 (20.74)
II	9758 (24.37)	8662 (24.97)	1096 (20.46)
III	9886 (24.69)	8604 (24.80)	1282 (23.93)
IV	10,582 (26.42)	8719 (25.13)	1863 (34.77)
Smoking status			
Never	22,915 (57.22)	20,047 (57.79)	2868 (53.53)
Previous	13,499 (33.71)	11,691 (33.70)	1808 (33.74)
Current	3467 (8.66)	2822 (8.14)	645 (12.04)
Alcohol intake			
Never	3259 (8.14)	2588 (7.46)	671 (12.52)
Special occasions only	4613 (11.52)	3836 (11.06)	777 (14.50)
One to three times a month	4525 (11.30)	3900 (11.24)	625 (11.66)
Once or twice a week	10,551 (26.35)	9251 (26.67)	1300 (24.26)
Three or four times a week	9152 (22.85)	8121 (23.41)	1031 (19.24)
Daily or almost daily	7864 (19.64)	6938 (20.00)	926 (17.28)
Body mass index			
less than 18.5	169 (0.42)	143 (0.41)	26 (0.49)
18.5 to 25	12,767 (31.88)	11,551 (33.30)	1216 (22.70)
25 to 30	17,238 (43.04)	15,152 (43.68)	2086 (38.93)
greater than 30	9826 (24.54)	7812 (22.52)	2014 (37.59)
Physical activities, min			
0	667 (1.67)	515 (1.48)	152 (2.84)
0-600	5480 (13.68)	4596 (13.25)	884 (16.50)
600-3000	16,360 (40.85)	14,463 (41.69)	1897 (35.41)
greater than 3000	9897 (24.71)	8722 (25.14)	1175 (21.93)
Education status			
College or University degree	13,472 (33.64)	11,961 (34.48)	1511 (28.20)
NVQ or HND or HNC or equivalent	2579 (6.44)	2175 (6.27)	404 (7.54)
Other professional qualifications	2100 (5.24)	1808 (5.21)	292 (5.45)
A levels/AS levels or equivalent	4619 (11.53)	4065 (11.72)	554 (10.34)
Ordinary levels/GCSEs or equivalent / CSEs or equivalent	10,664 (26.63)	9279 (26.75)	1385 (25.85)
None of the above	6193 (15.46)	5071 (14.62)	1122 (20.94)
Income per year			
greater than £100,000	1913 (4.78)	1740 (5.02)	173 (3.23)
£52,000 to £100,000	7087 (17.70)	6345 (18.29)	742 (13.85)
£31,000 to £51,999	9052 (22.60)	8001 (23.06)	1051 (19.62)
£18,000 to £30,999	8865 (22.14)	7666 (22.10)	1199 (22.38)
less than £18,000	7392 (18.46)	6142 (17.71)	1250 (23.33)
High density lipoprotein cholesterol			
Low	7093 (17.71)	5798 (16.71)	1295 (24.17)
High	27,876 (69.61)	24,510 (70.66)	3366 (62.82)
Triglycerides			
Low	23,059 (57.58)	20,379 (58.75)	2680 (50.02)
High	15,191 (37.93)	12,779 (36.84)	2412 (45.02)
Hypertension	17,353 (43.33)	14,709 (42.40)	2644 (49.35)
Glycosylated hemoglobin	1368 (3.42)	949 (2.74)	419 (7.82)
Glucose-lowering drugs	1433 (3.58)	1027 (2.96)	406 (7.58)
Lipid-lowering drugs	6882 (17.18)	5544 (15.98)	1338 (24.97)
FVC, % predicted	97.03 (87.61-106.31)	99.30 (91.71-107.82)	75.54 (69.83-80.21)
FEV1, % predicted	95.99 (86.45-105.36)	98.28 (90.68-106.92)	73.88 (68.59-77.41)
FEV1 to FVC ratio	0.78 (0.75-0.81)	0.78 (0.75-0.81)	0.76 (0.73-0.80)
Follow-up time, y	13.74 (13.04-14.48)	13.76 (13.07-14.49)	13.59 (12.81-14.40)

Data were presented as frequency (%) or median (P_{25}-P_{75}).

Abbreviations: A levels, Advanced Levels; AS levels, Advanced Subsidiary Levels; CSE, Certificate of Secondary Education; FEV1, forced expiratory volume in one second; FVC, forced vital capacity; GCSE, General Certificate of Secondary Education; HNC, Higher National Certificate; HND, Higher National Diploma; NVQ, National Vocational Qualification; PRISm, preserved ratio impaired spirometry; y, year.

Supplementary Table 2 Associations of transition trajectories of normal spirometry with incident HF after excluding morbidity cases that occurred within two years of follow up

	N_{case}/N_{total}	Unadjusted	Adjusted for age and sex	Fully adjusted [a]
		HR (95% CI)	HR (95% CI)	HR (95% CI)
Normal to Normal	136/27,790	REF	REF	REF
Normal to PRISm	14/1318	2.443 (1.409, 4.235)	2.742 (1.581, 4.756)	2.215 (1.267, 3.874)
Normal to Obstructive	28/2998	2.130 (1.418, 3.200)	1.619 (1.075, 2.439)	1.673 (1.107, 2.530)

Abbreviation: HF, heart failure; PRISm, preserved ratio impaired spirometry.

[a] Adjusted for age, sex, ethnicity, Townsend deprivation index, smoking status, alcohol intake, physical activity, body mass index, household income, education status, hypertension, high density lipoprotein, triglycerides, glycosylated hemoglobin, glucose-lowering drugs, lipid-lowering drugs, coronary heart disease at baseline and stroke at baseline.

Supplementary Table 3 Associations of transition trajectories of normal spirometry with incident HF using LLN definition for FEV1/FVC

	N_{case}/N_{total}	Unadjusted	Adjusted for age and sex	Fully adjusted [a]
		HR (95% CI)	HR (95% CI)	HR (95% CI)
Normal to Normal	236/31,147	REF	REF	REF
Normal to PRISm	35/1997	2.544 (1.783, 3.628)	2.475 (1.735, 3.532)	2.087 (1.454, 2.996)
Normal to Obstructive	34/2038	2.373 (1.656, 3.400)	2.366 (1.650, 3.393)	2.649 (1.841, 3.810)

Abbreviation: HF, heart failure; FEV1, forced expiratory volume in one second; FVC, forced vital capacity; PRISm, preserved ratio impaired spirometry.

[a] Adjusted for age, sex, ethnicity, Townsend deprivation index, smoking status, alcohol intake, physical activity, body mass index, household income, education status, hypertension, high density lipoprotein, triglycerides, glycosylated hemoglobin, glucose-lowering drugs, lipid-lowering drugs, coronary heart disease at baseline and stroke at baseline.

Supplementary Table 4 Associations of transition trajectories of normal spirometry with incident HF (Fine-Gray model [a])

	N_{case}/N_{total}	Unadjusted	Adjusted for age and sex	Fully adjusted [b]
		SHR (95% CI)	SHR (95% CI)	SHR (95% CI)
Normal to Normal	210/27,864	REF	REF	REF
Normal to PRISm	19/1323	2.036 (1.274, 3.255)	2.212 (1.387, 3.528)	1.794 (1.134, 2.837)
Normal to Obstructive	45/3015	2.118 (1.535, 2.923)	1.609 (1.162, 2.229)	1.703 (1.228, 2.362)

Abbreviation: HF, heart failure; PRISm, preserved ratio impaired spirometry; SHR, sub-distribution hazard ratio.

[a] Fine-Gray models considered all-cause death as a competing risk.

[b] Adjusted for age, sex, ethnicity, Townsend deprivation index, smoking status, alcohol intake, physical activity, body mass index, household income, education status, hypertension, high density lipoprotein, triglycerides, glycosylated hemoglobin, glucose-lowering drugs, lipid-lowering drugs, coronary heart disease at baseline and stroke at baseline.

Supplementary Table 5 Subgroup analyses of associations between transitions of normal spirometry and incident HF

	Normal-normal	Normal-PRISm		Normal-obstructive	
		HR (95% CI) [a]	*P* for interaction	HR (95% CI) [a]	*P* for interaction
Age			0.1743		0.9644
<60	REF	0.964 (0.349-2.663)		1.703 (0.899-3.224)	
≥60	REF	2.320 (1.347-3.998)		1.728 (1.179-2.532)	
Sex			0.7087		0.6115
Male	REF	2.052 (1.149-3.665)		1.702 (1.126-2.571)	
Female	REF	1.626 (0.697-3.795)		1.803 (1.042-3.119)	
Townsend deprivation index			0.5057		0.1228
Above median	REF	1.617 (0.739-3.537)		1.261 (0.740-2.149)	
Others	REF	1.923 (1.045-3.539)		2.240 (1.471-3.412)	

Abbreviations: HF, heart failure; HR, hazard ratio; PRISm, preserved ratio impaired spirometry.

[a] Adjusted for age, sex, ethnicity, Townsend deprivation index, smoking status, alcohol intake, physical activity, body mass index, household income, education status, hypertension, high density lipoprotein, triglycerides, glycosylated hemoglobin, glucose-lowering drugs, lipid-lowering drugs, coronary heart disease at baseline and stroke at baseline.

Supplementary Table 6 SNPs used as instruments and their association with the exposure and outcome

SNP ID	EA	OA	EAF	Exposure				Outcome		
				β	SE	*P* value	*F* statistic	β	SE	*P* value
rs11118683	T	C	0.4288	-0.00578	0.0012	1.40×10^{-06}	23.22165	-0.0074	0.0038	0.05028
rs111447148	A	G	0.0273	0.017205	0.003763	4.80×10^{-06}	20.90683	0.0103	0.013	0.4284
rs114516591	G	C	0.014	0.027034	0.005786	3.00×10^{-06}	21.83271	-0.0172	0.023	0.4537
rs117219834	G	A	0.0196	0.018438	0.004015	4.40×10^{-06}	21.08368	-0.0053	0.016	0.7431
rs12326273	C	T	0.0169	-0.02705	0.005705	2.10×10^{-06}	22.49348	-0.0054	0.0226	0.8112
rs12583657	A	G	0.1282	0.007831	0.001705	4.40×10^{-06}	21.08829	0.0088	0.0057	0.1244
rs12606772	C	T	0.6141	-0.00591	0.001261	2.80×10^{-06}	21.97544	-0.0043	0.0046	0.3483
rs142677543	A	G	0.0064	-0.03342	0.006979	1.70×10^{-06}	22.9376	-0.0428	0.0382	0.2624
rs185980168	A	G	0.0256	0.020284	0.004387	3.80×10^{-06}	21.37558	0.0061	0.019	0.7463
rs191921080	A	G	0.0798	0.009391	0.002051	4.70×10^{-06}	20.95784	0.0121	0.0076	0.1127
rs200085936	C	G	0.0176	0.02185	0.004636	2.40×10^{-06}	22.21896	-0.0312	0.0236	0.1867
rs2148172	C	A	0.6262	-0.00551	0.001201	4.40×10^{-06}	21.0758	-0.0114	0.0038	0.002509
rs2391467	G	A	0.5063	-0.0054	0.001178	4.60×10^{-06}	21.00397	0.0021	0.0039	0.5813
rs2637253	T	G	0.457	0.00536	0.001174	4.90×10^{-06}	20.85915	-0.0036	0.004	0.3645
rs272668	G	A	0.4233	-0.00551	0.001176	2.80×10^{-06}	21.94286	0.0028	0.0039	0.4691
rs28590589	C	T	0.1909	-0.00738	0.001452	3.70×10^{-07}	25.86348	-0.0068	0.0046	0.1429
rs34037938	C	A	0.0279	-0.01705	0.003191	9.20×10^{-08}	28.54439	-9.00E-04	0.0146	0.9501
rs4822731	G	C	0.1468	-0.01029	0.002108	1.10×10^{-06}	23.83303	-0.0022	0.0062	0.7166
rs4921274 [a]	T	C	0.8323	-0.00796	0.001699	2.80×10^{-06}	21.95819	-0.0258	0.0054	1.54E-06
rs533141018	T	C	0.021	0.018372	0.003878	2.20×10^{-06}	22.44548	-0.0218	0.0217	0.3148
rs553682853	A	G	0.1313	0.009447	0.001705	3.00×10^{-08}	30.70295	0.0365	0.011	0.000889
rs600244	T	G	0.7685	0.007102	0.001556	5.00×10^{-06}	20.84472	-0.0022	0.0049	0.6576
rs62527876	C	T	0.0309	-0.01304	0.002769	2.50×10^{-06}	22.17807	-0.0429	0.0136	0.001622
rs7026928	A	G	0.1732	0.007152	0.001517	2.40×10^{-06}	22.2222	0.0061	0.0049	0.2161
rs71563618	G	A	0.1071	0.008986	0.001842	1.10×10^{-06}	23.79705	-0.0144	0.0067	0.03212
rs7213474	C	T	0.6436	-0.00707	0.001244	1.30×10^{-08}	32.28545	-0.0024	0.004	0.5517
rs72949163	T	C	0.1346	-0.00834	0.001757	2.10×10^{-06}	22.52032	-0.0048	0.0057	0.4019
rs74351499	G	C	0.1264	0.012183	0.0025	1.10×10^{-06}	23.7513	0.0114	0.0068	0.09649
rs78965196	A	G	0.0347	-0.01421	0.002952	1.50×10^{-06}	23.17505	-0.0068	0.0118	0.5632
rs9378509	C	T	0.6218	-0.00655	0.001234	1.10×10^{-07}	28.16152	0.0025	0.0044	0.5713
rs9843082	G	T	0.6167	0.006135	0.001216	4.50×10^{-07}	25.47419	0.0064	0.0039	0.09709
rs9931938	C	T	0.3793	0.005454	0.001178	3.60×10^{-06}	21.44893	-0.0054	0.004	0.1819

Abbreviations: EA, effect allele; EAF, effect allele frequency; MR, Mendelian randomization; OA, other allele; SE, standard error; β, effect estimate;

[a] rs4921274 was detected as a significant outlier by the MR-pleiotropy residual sum and outlier analysis.

Supplementary Table 7 MR results for the causal relationship between COPD and HF

Exposure	Outcome	Method	nSNP	β	SE	OR (95% CI)	*P*
COPD	HF	IVW (fixed effects)	11	0.092	0.013	1.096 (1.069-1.124)	<0.001
		MR Egger	11	0.079	0.036	1.082 (1.008-1.162)	0.057
		Weighted median	11	0.121	0.019	1.129 (1.088-1.171)	<0.001
		Weighted mode	11	0.123	0.028	1.131 (1.070-1.195)	0.001
		RAPS	11	0.094	0.015	1.099 (1.067-1.131)	<0.001

Abbreviation: COPD, chronic obstructive pulmonary disease; HF, heart failure; IVW, inverse variance weighted; MR, Mendelian randomization; nSNP, number of single nucleotide polymorphisms; OR, odds ratio; PRISm, preserved ratio impaired spirometry.

Supplementary Table 8 Measures of heterogeneity with Cochran's Q test

Exposure	Outcome	MR analysis	Method	Q	Q_df	Q_pval
PRISm	HF	Raw data	MR Egger	72.490	30	2.235×10^{-05}
			IVW	72.510	31	3.556×10^{-05}
PRISm	HF	Outlier removed	MR Egger	55.597	29	0.002
			IVW	55.603	30	0.003

Abbreviation: HF, heart failure; IVW, inverse variance weighted; MR, Mendelian randomization; PRISm, preserved ratio impaired spirometry; Q_df, degrees of freedom associated with the Cochran Q test of heterogeneity; Q_pval, *P* value of Q test for heterogeneity.

Supplementary Table 9 Examination of horizontal pleiotropy effects with MR-Egger regression tests

Exposure	Outcome	MR analysis	egger_intercept	SE	*P*
PRISm	HF	Raw data	0.00039	0.00433	0.92824
		Outlier removed	0.00021	0.00386	0.95768

Abbreviation: HF, heart failure; MR, Mendelian randomization; PRISm, preserved ratio impaired spirometry; SE, standard error.

Supplementary Table 10 MR-PRESSO analysis for the causal relationship between PRISm and HF

Exposure	Outcome	MR analysis	Causal Estimate	T value	*P*-value	RSSobs	Global test *P* value	Distortion *P* value
PRISm	HF	Raw data	0.50492	2.50544	0.01769	76.90978	<0.001	0.379
		Outlier removed	0.39764	2.17434	0.03770			

Abbreviation: HF, heart failure; MR, Mendelian randomization; MR-PRESSO, MR pleiotropy residual sum and outlier; PRISm, preserved ratio impaired spirometry; RSSobs, observed residual sum of squares; SE, standard error.

Supplementary Table 11 Steiger directionality test for the causal relationship between PRISm and HF

Exposure	Outcome	MR analysis	R^2_exposure	R^2_outcome	Correct causal direction	*P* value
PRISm	HF	Raw data	0.002511	5.66×10^{-5}	TRUE	2.08×10^{-3}
PRISm	HF	Outlier removed	0.002437	4.67×10^{-5}	TRUE	4.72×10^{-3}

Abbreviation: HF, heart failure; MR, Mendelian randomization; PRISm, preserved ratio impaired spirometry.

Supplementary Table 12 Colocalization analysis of the association between PRISm and HF

SNP_id	Chromosome	Position	SNPs	PP.H0	PP.H1	PP.H2	PP.H3	PP.H4
rs4921274	5	158491655	6700	0.003915	0.001079	0.599945	0.165155	0.229906
rs553682853	17	44759766	6184	2.22E-07	0.000145	0.001358	0.884894	0.113603
rs2148172	13	71716891	7599	0.083094	0.032941	0.622517	0.246772	0.014676
rs71563618	7	114837804	5672	0.370131	0.291115	0.181733	0.142922	0.014099
rs11118683	1	221473248	7059	0.407172	0.368959	0.114311	0.103577	0.00598
rs9843082	3	132684372	7344	0.219327	0.281556	0.216283	0.277643	0.005191
rs74351499	4	19096154	8253	0.095407	0.494753	0.065476	0.339535	0.004828
rs2637253	10	78311287	6392	0.271095	0.38961	0.137361	0.197407	0.004527
rs34037938	19	32281390	6721	0.199179	0.512016	0.079669	0.204796	0.00434
rs191921080	2	157150418	6078	0.555782	0.24003	0.139705	0.060331	0.004152
rs111447148	14	78228485	7889	0.166355	0.10583	0.442456	0.281474	0.003885
rs185980168	4	17110169	7182	0.38289	0.084301	0.433612	0.095465	0.003732
rs78965196	2	155532242	7389	0.503525	0.17808	0.2329	0.082366	0.003129
rs533141018	16	2661860	6833	0.213303	0.115303	0.433815	0.234499	0.003079
rs4822731	22	26929173	8347	0.50197	0.329529	0.099901	0.065579	0.003021
rs272668	7	24131280	8242	0.546204	0.142995	0.243959	0.063865	0.002978
rs12606772	18	35266566	6226	0.552557	0.261378	0.12468	0.058975	0.002411
rs200085936	7	143956006	5021	0.001782	0.000441	0.798314	0.197329	0.002134
rs7026928	9	19881825	7730	0.346194	0.066187	0.49161	0.093986	0.002023
rs600244	11	128645057	7194	0.711161	0.081025	0.184938	0.021069	0.001806
rs9378509	6	8009268	7774	0.055827	0.728294	0.015249	0.198933	0.001697
rs2391467	1	94850443	6790	0.598149	0.186133	0.16323	0.050792	0.001695
rs7213474	17	79962718	8217	0.048351	0.349827	0.07288	0.527301	0.001641
rs117219834	14	57818928	7163	0.639174	0.077867	0.250772	0.030549	0.001638
rs114516591	1	155946015	4645	0.00607	0.001831	0.760919	0.229547	0.001634
rs12583657	13	25317575	8716	0.661421	0.063518	0.24956	0.023964	0.001536
rs72949163	11	81234533	8606	0.660103	0.110543	0.195218	0.03269	0.001446
rs12326273	18	75395383	9957	0.041102	0.012736	0.721382	0.223532	0.001248
rs9931938	16	87272970	11073	0.086074	0.009344	0.814956	0.088473	0.001153
rs142677543	4	81786127	7131	0.006415	0.007524	0.453413	0.53185	0.000798
rs28590589	18	57650243	8507	1.8E-08	2.62E-08	0.406322	0.59291	0.000768
rs62527876	8	125447641	7135	0.000117	2.11E-05	0.846289	0.152998	0.000576

Supplementary Table 13 Associations of PRISm with plasma proteins

	Protein's description used in the UK Biobank	Association of PRISm with plasma proteins				
		Coefficient	Lower	Upper	*P*	*FDR*
ABCA2	ATP-binding cassette sub-family A member 2	0.075	0.058	0.091	0.00000506	0.0000298
ABHD14B	Protein ABHD14B	-0.069	-0.084	-0.054	0.00000592	0.0000342
ABL1	Tyrosine-protein kinase ABL1	0.085	0.071	0.100	2.91E-09	2.91E-08
ABO	Histo-blood group ABO system transferase	0.061	0.045	0.078	0.000231579	0.000995448
ACADM	Medium-chain specific acyl-CoA dehydrogenase, mitochondrial	0.116	0.101	0.131	4.44E-14	8.15E-13
ACAN	Aggrecan core protein	0.100	0.085	0.115	2.99E-11	3.93E-10
ACE	Angiotensin-converting enzyme	0.088	0.073	0.102	5.91E-10	6.62E-09
ACHE	Acetylcholinesterase	0.128	0.112	0.143	7.2E-16	1.62E-14
ACP5	Tartrate-resistant acid phosphatase type 5	0.099	0.083	0.116	7.92E-10	8.73E-09
ACRBP	Acrosin-binding protein	0.157	0.142	0.172	3.22E-25	2.05E-23
ACRV1	Acrosomal protein SP-10	0.057	0.041	0.073	0.000349202	0.001449883
ACY1	Aminoacylase-1	0.079	0.065	0.094	8.38E-08	0.000000648
ACY3	N-acyl-aromatic-L-amino acid amidohydrolase	-0.121	-0.136	-0.107	2.03E-16	4.93E-15
ACYP1	Acylphosphatase-1	0.075	0.060	0.089	0.000000251	0.00000185
ADA	Adenosine deaminase	0.202	0.189	0.216	9.32E-50	1.36E-46
ADA2	Adenosine deaminase 2	0.051	0.035	0.068	0.001519548	0.005514073
ADAM12	Disintegrin and metalloproteinase domain-containing protein 12	-0.127	-0.143	-0.111	8.16E-16	1.79E-14
ADAM22	Disintegrin and metalloproteinase domain-containing protein 22	0.059	0.043	0.075	0.000259141	0.001107411
ADAM23	Disintegrin and metalloproteinase domain-containing protein 23	0.118	0.103	0.133	9.03E-15	1.79E-13
ADAM9	Disintegrin and metalloproteinase domain-containing protein 9	0.106	0.091	0.122	2.45E-11	3.26E-10
ADAMTS1	A disintegrin and metalloproteinase with thrombospondin motifs 1	0.067	0.050	0.083	0.0000539	0.000264935
ADAMTS16	A disintegrin and metalloproteinase with thrombospondin motifs 16	0.078	0.062	0.094	0.00000146	0.00000939
ADAMTSL2	ADAMTS-like protein 2	0.044	0.027	0.061	0.009391128	0.028010475
ADAMTSL4	ADAMTS-like protein 4	0.041	0.025	0.058	0.013200865	0.037251916
ADCYAP1R1	Pituitary adenylate cyclase-activating polypeptide type I receptor	0.055	0.040	0.070	0.000239835	0.001027913
ADD1	Alpha-adducin	0.043	0.028	0.059	0.005669123	0.018051032
ADGRB3	Adhesion G protein-coupled receptor B3	0.056	0.040	0.072	0.000424652	0.001743341
ADGRD1	Adhesion G-protein coupled receptor D1	0.054	0.039	0.068	0.000193747	0.000840241
ADGRE2	Adhesion G protein-coupled receptor E2	0.059	0.044	0.074	0.0000672	0.000321837
ADGRF5	Adhesion G protein-coupled receptor F5	0.069	0.052	0.085	0.000033	0.000168094
ADH1B	All-trans-retinol dehydrogenase	0.079	0.064	0.095	0.00000042	0.00000296
ADH4	All-trans-retinol dehydrogenase [NAD(+)] ADH4	0.044	0.028	0.061	0.007769027	0.023778918
ADIPOQ	Adiponectin	0.166	0.150	0.182	1.02E-25	7.09E-24
ADM	Pro-adrenomedullin	0.049	0.033	0.065	0.002742315	0.009497378
ADRA2A	Alpha-2A adrenergic receptor	0.111	0.095	0.126	9.34E-13	1.44E-11
AFAP1	Actin filament-associated protein 1	0.089	0.075	0.104	1.16E-09	1.26E-08
AFM	Afamin	0.076	0.060	0.092	0.00000182	0.0000114
AGER	Advanced glycosylation end product-specific receptor	0.082	0.066	0.099	0.000000616	0.00000424
AGR2	Anterior gradient protein 2 homolog	0.063	0.046	0.079	0.000142573	0.000636247
AGRN	Agrin	0.040	0.023	0.056	0.017331861	0.046778419
AGT	Angiotensinogen	0.041	0.024	0.058	0.014600882	0.040684821
AHNAK	Neuroblast differentiation-associated protein AHNAK	0.065	0.049	0.081	0.0000578	0.000282579
AHNAK2	Protein AHNAK2	0.059	0.043	0.075	0.00027066	0.001151584
AHSP	Alpha-hemoglobin-stabilizing protein	-0.064	-0.079	-0.049	0.0000185	0.0000985
AIDA	Axin interactor, dorsalization-associated protein	0.068	0.052	0.085	0.0000391	0.000195928
AIF1L	Allograft inflammatory factor 1-like	0.088	0.072	0.105	7.19E-08	0.000000568
AKR1C4	Aldo-keto reductase family 1 member C4	0.087	0.071	0.103	3.78E-08	0.000000318
AKR7L	Aflatoxin B1 aldehyde reductase member 4	0.084	0.067	0.100	0.000000309	0.00000224
AKT1S1	Proline-rich AKT1 substrate 1	-0.111	-0.126	-0.096	7.62E-14	1.36E-12
ALCAM	CD166 antigen	0.109	0.094	0.123	1.55E-13	2.64E-12
ALDH5A1	Succinate-semialdehyde dehydrogenase, mitochondrial	0.048	0.031	0.065	0.00365343	0.012246532
ALPI	Intestinal-type alkaline phosphatase	0.041	0.025	0.058	0.013844847	0.03894946
AMIGO2	Amphoterin-induced protein 2	0.104	0.088	0.120	1.28E-10	1.55E-09
AMY1A_AMY1B_A	Alpha-amylase 1A_Alpha-amylase 1B_Alpha-amylase 1C	0.098	0.082	0.114	4.98E-10	5.67E-09
ANGPT1	Angiopoietin-1	0.042	0.026	0.059	0.00988618	0.029042518
ANGPT2	Angiopoietin-2	-0.043	-0.058	-0.027	0.006427734	0.020180738
ANGPTL1	Angiopoietin-related protein 1	0.047	0.030	0.063	0.00476519	0.015459102
ANGPTL2	Angiopoietin-related protein 2	0.040	0.024	0.057	0.014318183	0.040049807
ANKMY2	Ankyrin repeat and MYND domain-containing protein 2	0.040	0.024	0.056	0.013683356	0.038532224
ANP32C	Acidic leucine-rich nuclear phosphoprotein 32 family member C	0.088	0.071	0.105	0.00000014	0.00000105
ANPEP	Aminopeptidase N	0.041	0.025	0.058	0.011023812	0.031903566
ANXA1	Annexin A1	0.089	0.074	0.104	5.06E-09	4.83E-08
ANXA11	Annexin A11	0.050	0.035	0.066	0.00135867	0.005014386
ANXA3	Annexin A3	0.119	0.103	0.135	1.01E-13	1.78E-12
ANXA5	Annexin A5	0.078	0.062	0.093	0.000000759	0.00000513
AOC1	Amiloride-sensitive amine oxidase [copper-containing]	0.057	0.041	0.073	0.000446042	0.001818384
AOC3	Membrane primary amine oxidase	0.079	0.063	0.096	0.000000734	0.00000497
AP2B1	AP-2 complex subunit beta	0.139	0.123	0.155	4.69E-18	1.39E-16
AP3B1	AP-3 complex subunit beta-1	0.107	0.091	0.123	3.57E-11	4.61E-10
APOA2	Apolipoprotein A-II	-0.142	-0.157	-0.127	5.42E-22	2.26E-20
APOC1	Apolipoprotein C-I	0.084	0.068	0.099	7.04E-08	0.000000559
APOE	Apolipoprotein E	0.051	0.035	0.067	0.001446634	0.005292253
APOH	Beta-2-glycoprotein 1	0.077	0.061	0.093	0.000000828	0.00000558
APOL1	Apolipoprotein L1	0.121	0.108	0.135	1.24E-19	4.12E-18
APPL2	DCC-interacting protein 13-beta	0.073	0.057	0.089	0.00000617	0.0000354
APRT	Adenine phosphoribosyltransferase	0.062	0.046	0.078	0.000138883	0.000624546
ARHGAP1	Rho GTPase-activating protein 1	0.039	0.023	0.055	0.017579795	0.047307244
ARHGAP45	Rho GTPase-activating protein 45	0.157	0.142	0.172	1.68E-26	1.45E-24
ARHGAP5	Rho GTPase-activating protein 5	0.076	0.060	0.091	0.000000955	0.00000636
ARHGEF1	Rho guanine nucleotide exchange factor 1	0.053	0.037	0.070	0.000998953	0.00376281
ARHGEF10	Rho guanine nucleotide exchange factor 10	0.099	0.082	0.115	1.39E-09	1.49E-08
ARHGEF12	Rho guanine nucleotide exchange factor 12	0.114	0.098	0.130	8.7E-13	1.36E-11
ARHGEF5	Rho guanine nucleotide exchange factor 5	0.093	0.078	0.108	5.25E-10	5.92E-09
ARID3A	AT-rich interactive domain-containing protein 3A	0.081	0.065	0.098	0.000000629	0.00000431
ARID4B	AT-rich interactive domain-containing protein 4B	0.088	0.072	0.104	0.000000032	0.000000271
ARL13B	ADP-ribosylation factor-like protein 13B	0.097	0.081	0.113	2.97E-09	2.95E-08
ARMCX2	Armadillo repeat-containing X-linked protein 2	0.151	0.135	0.167	3.42E-21	1.31E-19
ARNT	Aryl hydrocarbon receptor nuclear translocator	0.136	0.120	0.152	4.61E-17	1.18E-15
ARNTL	Aryl hydrocarbon receptor nuclear translocator-like protein 1	0.137	0.121	0.152	3.98E-18	1.2E-16
ARSA	Arylsulfatase A	0.046	0.030	0.061	0.002905327	0.009979167
ART3	Ecto-ADP-ribosyltransferase 3	0.051	0.035	0.067	0.001423109	0.005232389
ART5	Ecto-ADP-ribosyltransferase 5	0.043	0.027	0.060	0.008702531	0.026175956
ARTN	Artemin	0.052	0.037	0.067	0.000584907	0.002313509
ASAH2	Neutral ceramidase	0.149	0.133	0.164	3.22E-21	1.25E-19
ASGR1	Asialoglycoprotein receptor 1	0.054	0.038	0.071	0.000902972	0.003441182
ASGR2	Asialoglycoprotein receptor 2	0.083	0.067	0.100	0.000000339	0.00000244
ASPN	Asporin	0.166	0.151	0.182	1.55E-26	1.42E-24
ASPSCR1	Tether containing UBX domain for GLUT4	0.098	0.082	0.115	1.78E-09	1.87E-08
ASRGL1	Isoaspartyl peptidase/L-asparaginase	0.047	0.032	0.062	0.001779598	0.006363255
ASS1	Argininosuccinate synthase	0.041	0.024	0.057	0.013259053	0.037373396
ATF2	Cyclic AMP-dependent transcription factor ATF-2	0.112	0.097	0.127	6.76E-14	1.23E-12
ATF4	Cyclic AMP-dependent transcription factor ATF-4	0.162	0.146	0.178	9.43E-24	5.1E-22
ATG16L1	Autophagy-related protein 16-1	0.104	0.089	0.118	1.24E-12	1.9E-11
ATP1B1	Sodium/potassium-transporting ATPase subunit beta-1	0.068	0.052	0.085	0.0000346	0.000174892

ATP1B2	Sodium/potassium-transporting ATPase subunit beta-2	0.073	0.057	0.089	0.00000578	0.0000335
ATP1B3	Sodium/potassium-transporting ATPase subunit beta-3	0.065	0.049	0.081	0.000051	0.000251974
ATP1B4	Protein ATP1B4	0.143	0.128	0.159	4.62E-20	1.59E-18
ATP5PO	ATP synthase subunit O, mitochondrial	-0.043	-0.059	-0.027	0.007469026	0.022932735
ATP6AP2	Renin receptor	0.057	0.040	0.073	0.00063309	0.002480594
ATP6V1G1	V-type proton ATPase subunit G 1	0.071	0.056	0.087	0.00000498	0.0000294
ATRAID	All-trans retinoic acid-induced differentiation factor	0.093	0.077	0.109	4.9E-09	4.69E-08
ATRN	Attractin, Isoform 2	0.095	0.079	0.111	4.82E-09	4.63E-08
ATXN10	Ataxin-10	0.039	0.023	0.055	0.016214919	0.044171677
ATXN2	Ataxin-2	0.081	0.064	0.097	0.000000948	0.00000632
ATXN3	Ataxin-3	0.073	0.057	0.089	0.00000376	0.0000228
AXIN1	Axin-1	0.175	0.160	0.190	1.69E-30	2.15E-28
AXL	Tyrosine-protein kinase receptor UFO	0.072	0.056	0.088	0.00000462	0.0000274
AZU1	Azurocidin	0.101	0.085	0.116	5.07E-11	6.47E-10
B3GAT3	Galactosylgalactosylxylosylprotein 3-beta-glucuronosyltransferase 3	-0.092	-0.108	-0.075	1.96E-08	0.000000173
B3GNT7	UDP-GlcNAc:betaGal beta-1,3-N-acetylglucosaminyltransferase 7	0.033	0.020	0.046	0.012273186	0.035033714
B4GALT1	Beta-1,4-galactosyltransferase 1	0.096	0.080	0.111	4.82E-10	5.5E-09
B4GAT1	Beta-1,4-glucuronyltransferase 1	0.076	0.059	0.092	0.00000391	0.0000235
BABAM1	BRISC and BRCA1-A complex member 1	0.055	0.039	0.072	0.000821983	0.003153093
BACH1	Transcription regulator protein BACH1	0.062	0.046	0.078	0.000127195	0.00057642
BAG3	BAG family molecular chaperone regulator 3	0.041	0.025	0.057	0.011695064	0.033547275
BAG4	BAG family molecular chaperone regulator 4	0.084	0.068	0.099	7.79E-08	0.000000609
BAIAP2	Brain-specific angiogenesis inhibitor 1-associated protein 2	-0.052	-0.069	-0.036	0.001516072	0.005514073
BAMBI	BMP and activin membrane-bound inhibitor homolog	0.114	0.099	0.129	2.88E-14	5.34E-13
BANK1	B-cell scaffold protein with ankyrin repeats	0.051	0.035	0.067	0.001420751	0.005230296
BAP18	Chromatin complexes subunit BAP18	0.050	0.033	0.066	0.002469754	0.008614667
BATF	Basic leucine zipper transcriptional factor ATF-like	0.055	0.038	0.072	0.000977909	0.003693058
BCAM	Basal cell adhesion molecule	0.117	0.102	0.132	1.59E-14	3.09E-13
BCAN	Brevican core protein	0.094	0.078	0.110	2.69E-09	2.72E-08
BCAT1	Branched-chain-amino-acid aminotransferase, cytosolic	0.080	0.064	0.095	0.000000256	0.00000187
BCL2	Apoptosis regulator Bcl-2	0.049	0.033	0.066	0.002915545	0.010002511
BCL2L1	Bcl-2-like protein 1	0.053	0.039	0.067	0.000171823	0.000755248
BCL2L11	Bcl-2-like protein 11, Isoform BimL	0.100	0.084	0.115	1.39E-10	1.66E-09
BCL7B	B-cell CLL/lymphoma 7 protein family member B	0.102	0.087	0.116	1.63E-12	2.46E-11
BCR	Breakpoint cluster region protein	0.064	0.047	0.080	0.000102901	0.000478186
BDNF	Brain-derived neurotrophic factor	0.042	0.028	0.056	0.002841471	0.009794949
BEX3	Protein BEX3	0.128	0.114	0.143	8.68E-19	2.82E-17
BHMT2	S-methylmethionine--homocysteine S-methyltransferase BHMT2	0.073	0.056	0.090	0.0000126	0.0000687
BID	BH3-interacting domain death agonist	0.132	0.120	0.145	4.76E-26	3.48E-24
BIN2	Bridging integrator 2	0.076	0.060	0.092	0.00000126	0.00000818
BLOC1S2	Biogenesis of lysosome-related organelles complex 1 subunit 2	0.063	0.047	0.080	0.0000922	0.00043243
BLOC1S3	Biogenesis of lysosome-related organelles complex 1 subunit 3	0.070	0.053	0.086	0.0000227	0.000118043
BMP10	Bone morphogenetic protein 10	0.043	0.026	0.059	0.009585856	0.028434551
BMP6	Bone morphogenetic protein 6	0.051	0.035	0.068	0.001587423	0.005721379
BMPER	BMP-binding endothelial regulator protein	0.072	0.056	0.088	0.00000863	0.0000485
BNIP2	BCL2/adenovirus E1B 19 kDa protein-interacting protein 2	0.043	0.026	0.059	0.010166023	0.029715286
BOLA1	BolA-like protein 1	0.059	0.042	0.075	0.000433149	0.001770761
BOLA2_BOLA2B	BolA-like protein 2	0.047	0.030	0.064	0.004901793	0.015831979
BPIFB1	BPI fold-containing family B member 1	0.048	0.032	0.065	0.004117246	0.013583194
BPIFB2	BPI fold-containing family B member 2	0.071	0.056	0.086	0.00000121	0.00000789
BRAP	BRCA1-associated protein	0.080	0.065	0.096	0.000000384	0.00000273
BRD3	Bromodomain-containing protein 3	0.041	0.024	0.057	0.014339527	0.040071164
BRK1	Protein BRICK1	0.072	0.056	0.089	0.00000909	0.0000508
BSND	Barttin	0.124	0.109	0.140	6.98E-16	1.59E-14
BTD	Biotinidase	0.108	0.094	0.123	7.3E-14	1.31E-12
BTLA	B- and T-lymphocyte attenuator	0.114	0.097	0.130	3.62E-12	5.28E-11
BTN2A1	Butyrophilin subfamily 2 member A1	0.119	0.103	0.135	5.56E-14	1.02E-12
BTN3A2	Butyrophilin subfamily 3 member A2	0.040	0.023	0.057	0.017008364	0.046032823
BTNL10	Butyrophilin-like protein 10	0.060	0.043	0.077	0.00028593	0.001209512
C19orf12	Protein C19orf12	0.042	0.026	0.059	0.010155964	0.029715286
C1GALT1C1	C1GALT1-specific chaperone 1	0.085	0.069	0.102	0.000000268	0.00000195
C1QA	Complement C1q subcomponent subunit A	0.049	0.033	0.065	0.00212723	0.007500475
C1QBP	Complement component 1 Q subcomponent-binding protein, mitochondrial	0.079	0.062	0.096	0.00000187	0.0000117
C1QL2	Complement C1q-like protein 2	0.059	0.042	0.075	0.000350775	0.001454349
C1QTNF1	Complement C1q tumor necrosis factor-related protein 1	0.042	0.026	0.058	0.00868497	0.026171307
C1QTNF6	Complement C1q tumor necrosis factor-related protein 6	0.076	0.061	0.092	0.00000113	0.00000737
C1R	Complement C1r subcomponent	0.080	0.065	0.096	0.000000378	0.00000269
C1RL	Complement C1r subcomponent-like protein	0.093	0.077	0.110	1.28E-08	0.000000116
C1S	Complement C1s subcomponent	0.048	0.031	0.064	0.00344905	0.011682008
C2CD2L	Phospholipid transfer protein C2CD2L	0.129	0.115	0.143	4.3E-20	1.5E-18
C2orf69	UPF0565 protein C2orf69	0.043	0.026	0.059	0.010200159	0.029785279
C7	Complement component C7	0.106	0.090	0.122	8.51E-11	1.05E-09
C9orf40	Uncharacterized protein C9orf40	0.050	0.033	0.066	0.003447849	0.011682008
CA1	Carbonic anhydrase 1	0.041	0.024	0.057	0.014697898	0.04091615
CA13	Carbonic anhydrase 13	0.066	0.050	0.082	0.0000374	0.000187831
CA5A	Carbonic anhydrase 5A, mitochondrial	0.152	0.137	0.167	6.48E-24	3.57E-22
CA7	Carbonic anhydrase 7	0.050	0.034	0.066	0.001520473	0.005514073
CA8	Carbonic anhydrase-related protein	0.183	0.168	0.197	2.73E-35	6.64E-33
CABP2	Calcium-binding protein 2	0.133	0.119	0.148	2.09E-20	7.35E-19
CACNA1C	Voltage-dependent L-type calcium channel subunit alpha-1C	0.076	0.061	0.092	0.00000103	0.00000681
CACYBP	Calcyclin-binding protein	0.068	0.051	0.084	0.0000474	0.000234746
CADPS	Calcium-dependent secretion activator 1	0.056	0.039	0.072	0.000628942	0.002470965
CALB1	Calbindin	0.060	0.044	0.076	0.000231524	0.000995448
CALB2	Calretinin	0.044	0.028	0.060	0.007005023	0.021782642
CALCB	Calcitonin gene-related peptide 2	0.121	0.107	0.135	8.49E-18	2.39E-16
CALCOCO2	Calcium-binding and coiled-coil domain-containing protein 2	0.078	0.062	0.095	0.00000232	0.0000144
CALY	Neuron-specific vesicular protein calcyon	0.060	0.043	0.076	0.000309102	0.001294418
CAMKK1	Calcium/calmodulin-dependent protein kinase kinase 1	0.042	0.026	0.059	0.009698948	0.028636389
CANT1	Soluble calcium-activated nucleotidase 1	0.090	0.074	0.105	1.33E-08	0.00000012
CAPG	Macrophage-capping protein	0.072	0.056	0.088	0.00000899	0.0000504
CAPN3	Calpain-3	-0.062	-0.079	-0.045	0.000186879	0.00081408
CAPS	Calcyphosin	0.130	0.114	0.145	3.72E-17	9.71E-16
CASC3	Protein CASC3	0.100	0.085	0.116	2.61E-10	3.07E-09
CASP1	Caspase-1	0.044	0.029	0.059	0.003079337	0.010502802
CASP10	Caspase-10	-0.047	-0.062	-0.033	0.000919954	0.003496784
CASP3	Caspase-3	0.118	0.103	0.134	6.81E-14	1.23E-12
CASP7	Caspase-7	-0.069	-0.085	-0.053	0.00000902	0.0000505
CASQ2	Calsequestrin-2	0.182	0.167	0.197	2.11E-32	3.25E-30
CAT	Catalase	0.078	0.062	0.095	0.00000255	0.0000157
CBLIF	Cobalamin binding intrinsic factor	0.069	0.053	0.086	0.0000212	0.000110894
CBLN1	Cerebellin-1	0.154	0.139	0.168	1.4E-26	1.32E-24
CBLN4	Cerebellin-4	0.039	0.023	0.055	0.015684987	0.043008647
CBX2	Chromobox protein homolog 2	0.083	0.067	0.100	0.000000465	0.00000326
CC2D1A	Coiled-coil and C2 domain-containing protein 1A	0.084	0.068	0.100	0.000000133	0.000000999
CCDC80	Coiled-coil domain-containing protein 80	0.063	0.047	0.079	0.000103337	0.000478693
CCER2	Coiled-coil domain-containing glutamate-rich protein 2	0.129	0.113	0.144	7.38E-17	1.86E-15
CCL13	C-C motif chemokine 13	0.058	0.041	0.074	0.000413926	0.001701695

CCL14	C-C motif chemokine 14	0.104	0.088	0.120	5.11E-11	6.49E-10
CCL15	C-C motif chemokine 15	0.088	0.072	0.103	1.42E-08	0.000000127
CCL16	C-C motif chemokine 16	0.127	0.109	0.145	1.45E-12	2.19E-11
CCL18	C-C motif chemokine 18	0.100	0.084	0.115	1.32E-10	1.59E-09
CCL2	C-C motif chemokine 2	0.075	0.059	0.092	0.00000448	0.0000266
CCL20	C-C motif chemokine 20	0.040	0.024	0.057	0.013974043	0.039237395
CCL21	C-C motif chemokine 21	0.120	0.104	0.135	1.92E-14	3.63E-13
CCL22	C-C motif chemokine 22	0.119	0.103	0.135	3.42E-13	5.68E-12
CCL24	C-C motif chemokine 24	0.142	0.126	0.159	1.38E-18	4.37E-17
CCL26	C-C motif chemokine 26	0.151	0.135	0.166	5.95E-22	2.45E-20
CCL27	C-C motif chemokine 27	0.065	0.052	0.078	0.000000681	0.00000465
CCL3	C-C motif chemokine 3	0.054	0.038	0.070	0.000773446	0.002989869
CCL5	C-C motif chemokine 5	0.063	0.047	0.080	0.000125976	0.000571781
CCL7	C-C motif chemokine 7	0.121	0.105	0.136	7.87E-15	1.59E-13
CCN1	CCN family member 1	0.065	0.049	0.081	0.0000648	0.000310783
CCN4	CCN family member 4	0.054	0.037	0.070	0.001141174	0.00426554
CCND2	G1/S-specific cyclin-D2	-0.047	-0.062	-0.031	0.002236724	0.007829873
CD109	CD109 antigen	0.081	0.065	0.098	0.000000513	0.00000356
CD14	Monocyte differentiation antigen CD14	0.042	0.027	0.056	0.005183266	0.01663083
CD160	CD160 antigen	0.054	0.038	0.070	0.000798916	0.003080779
CD163	Scavenger receptor cysteine-rich type 1 protein M130	-0.042	-0.059	-0.026	0.009692511	0.028636389
CD164	Sialomucin core protein 24	0.047	0.031	0.063	0.003918248	0.013014817
CD177	CD177 antigen	0.047	0.030	0.063	0.00484951	0.01568044
CD1C	T-cell surface glycoprotein CD1c	0.078	0.062	0.095	0.00000244	0.0000151
CD2	T-cell surface antigen CD2	0.091	0.075	0.106	2.31E-09	2.38E-08
CD200	OX-2 membrane glycoprotein	0.040	0.024	0.057	0.015241012	0.042107257
CD209	CD209 antigen	0.065	0.048	0.081	0.0000782	0.000372486
CD226	CD226 antigen	0.058	0.042	0.074	0.000347348	0.001444237
CD27	CD27 antigen	0.075	0.060	0.091	0.00000143	0.00000918
CD2AP	CD2-associated protein	-0.042	-0.058	-0.026	0.007396723	0.02275855
CD300LG	CMRF35-like molecule 9	0.044	0.028	0.061	0.007704344	0.023605658
CD302	CD302 antigen	0.057	0.041	0.073	0.000397742	0.001639773
CD34	Hematopoietic progenitor cell antigen CD34	-0.037	-0.053	-0.021	0.018381908	0.049024013
CD38	ADP-ribosyl cyclase/cyclic ADP-ribose hydrolase 1	0.119	0.105	0.133	1.26E-16	3.15E-15
CD3D	T-cell surface glycoprotein CD3 delta chain	0.091	0.075	0.107	8.83E-09	8.14E-08
CD3E	T-cell surface glycoprotein CD3 epsilon chain	0.074	0.058	0.090	0.00000565	0.0000329
CD4	T-cell surface glycoprotein CD4	0.083	0.068	0.099	6.99E-08	0.000000557
CD40	Tumor necrosis factor receptor superfamily member 5	-0.044	-0.060	-0.029	0.004013765	0.013301854
CD40LG	CD40 ligand	0.043	0.028	0.059	0.005582047	0.017851558
CD48	CD48 antigen	0.049	0.033	0.064	0.001441221	0.005279059
CD59	CD59 glycoprotein	0.058	0.042	0.074	0.000289377	0.001222325
CD6	T-cell differentiation antigen CD6	0.113	0.100	0.126	1.01E-17	2.77E-16
CD7	T-cell antigen CD7	0.128	0.114	0.143	1.99E-18	6.18E-17
CD70	CD70 antigen	0.112	0.096	0.128	1.43E-12	2.18E-11
CD72	B-cell differentiation antigen CD72	0.124	0.109	0.139	7.22E-16	1.62E-14
CD74	HLA class II histocompatibility antigen gamma chain	0.044	0.028	0.061	0.007818412	0.023905039
CD79B	B-cell antigen receptor complex-associated protein beta chain	0.096	0.080	0.112	3.68E-09	3.61E-08
CD80	T-lymphocyte activation antigen CD80	-0.046	-0.062	-0.030	0.004163598	0.013705178
CD82	CD82 antigen	0.049	0.033	0.065	0.002360979	0.008254953
CD84	SLAM family member 5	0.044	0.027	0.060	0.007593838	0.023291489
CD93	Complement component C1q receptor	0.075	0.059	0.091	0.00000419	0.0000251
CD99	CD99 antigen	0.106	0.089	0.123	1.47E-10	1.75E-09
CD99L2	CD99 antigen-like protein 2	0.047	0.031	0.063	0.003873491	0.01289546
CDAN1	Codanin-1	0.040	0.024	0.057	0.015395537	0.042413907
CDC123	Cell division cycle protein 123 homolog	0.077	0.063	0.090	1.21E-08	0.00000011
CDC25A	M-phase inducer phosphatase 1	-0.056	-0.072	-0.041	0.00029782	0.00125256
CDC26	Anaphase-promoting complex subunit CDC26	0.099	0.083	0.115	1.54E-09	1.64E-08
CDC27	Cell division cycle protein 27 homolog	0.069	0.052	0.086	0.0000415	0.000207385
CDH1	Cadherin-1	0.041	0.026	0.055	0.005069085	0.016300261
CDH17	Cadherin-17	0.064	0.048	0.080	0.0000473	0.000234582
CDH2	Cadherin-2	0.041	0.025	0.057	0.010822168	0.031444529
CDH23	Cadherin-23	0.044	0.027	0.061	0.009836976	0.028956174
CDH3	Cadherin-3	0.119	0.102	0.135	4.62E-13	7.51E-12
CDH4	Cadherin-4	0.075	0.059	0.091	0.0000018	0.0000113
CDH5	Cadherin-5	0.101	0.086	0.116	1.54E-11	2.12E-10
CDHR5	Cadherin-related family member 5	0.059	0.043	0.076	0.000346951	0.001444237
CDK1	Cyclin-dependent kinase 1	0.056	0.040	0.072	0.000528519	0.002113353
CDKN1A	Cyclin-dependent kinase inhibitor 1	0.096	0.081	0.112	1.01E-09	1.11E-08
CDKN2D	Cyclin-dependent kinase 4 inhibitor D	0.056	0.040	0.072	0.000586168	0.002315363
CDNF	Cerebral dopamine neurotrophic factor	0.060	0.044	0.077	0.000271488	0.001153431
CEACAM1	Carcinoembryonic antigen-related cell adhesion molecule 1	0.122	0.107	0.137	2.93E-16	6.95E-15
CEACAM16	Carcinoembryonic antigen-related cell adhesion molecule 16	0.105	0.090	0.121	3.57E-12	5.24E-11
CEACAM18	Carcinoembryonic antigen-related cell adhesion molecule 18	0.041	0.024	0.057	0.012958949	0.036633469
CEACAM19	Carcinoembryonic antigen-related cell adhesion molecule 19	0.164	0.152	0.176	1.56E-40	5.68E-38
CEACAM20	Carcinoembryonic antigen-related cell adhesion molecule 20	0.056	0.040	0.073	0.00049909	0.002006658
CEACAM8	Carcinoembryonic antigen-related cell adhesion molecule 8	0.089	0.074	0.105	1.17E-08	0.000000107
CEBPA	CCAAT/enhancer-binding protein alpha	0.056	0.040	0.072	0.000476272	0.001920196
CELA3A	Chymotrypsin-like elastase family member 3A	0.078	0.062	0.094	0.000000889	0.00000597
CELSR2	Cadherin EGF LAG seven-pass G-type receptor 2	0.096	0.080	0.113	4.22E-09	4.11E-08
CEMIP2	Cell surface hyaluronidase	0.068	0.052	0.084	0.0000244	0.000126224
CEND1	Cell cycle exit and neuronal differentiation protein 1	0.104	0.088	0.121	2.83E-10	3.31E-09
CEP112	Centrosomal protein of 112 kDa	0.039	0.023	0.055	0.016151458	0.044039842
CEP20	Centrosomal protein 20	0.093	0.077	0.108	3.28E-09	3.25E-08
CEP350	Centrosome-associated protein 350	0.073	0.056	0.089	0.0000119	0.0000652
CERT	Ceramide transfer protein	0.067	0.051	0.083	0.0000177	0.0000944
CES3	Carboxylesterase 3	0.142	0.127	0.157	6.82E-21	2.55E-19
CETN2	Centrin-2	0.150	0.134	0.166	9.58E-21	3.46E-19
CFB	Complement factor B	-0.037	-0.053	-0.021	0.017537867	0.047247176
CFC1	Cryptic protein	0.088	0.073	0.103	2.49E-09	2.54E-08
CFHR5	Complement factor H-related protein 5	0.048	0.031	0.064	0.004058045	0.013433369
CGA	Glycoprotein hormones alpha chain	0.042	0.026	0.059	0.011010116	0.03189551
CGB3_CGB5_CGB8	Choriogonadotropin subunit beta 3	0.072	0.056	0.089	0.0000106	0.0000591
CGN	Cingulin	0.043	0.027	0.060	0.008736491	0.026245387
CGREF1	Cell growth regulator with EF hand domain protein 1	-0.064	-0.081	-0.048	0.000120858	0.000551981
CHAC2	Glutathione-specific gamma-glutamylcyclotransferase 2	0.080	0.064	0.095	0.000000379	0.00000269
CHAD	Chondroadherin	0.058	0.042	0.074	0.000290965	0.001227258
CHEK2	Serine/threonine-protein kinase Chk2	-0.081	-0.096	-0.065	0.00000021	0.00000156
CHGA	Chromogranin-A	0.093	0.078	0.109	1.51E-09	1.61E-08
CHL1	Neural cell adhesion molecule L1-like protein	0.040	0.023	0.057	0.017062052	0.04613541
CHMP6	Charged multivesicular body protein 6	0.181	0.166	0.195	3.01E-35	6.76E-33
CHP1	Calcineurin B homologous protein 1	0.057	0.041	0.073	0.00052057	0.00208728
CHRDL1	Chordin-like protein 1	0.183	0.168	0.197	2.5E-37	6.65E-35
CHRDL2	Chordin-like protein 2	0.047	0.031	0.064	0.004531462	0.014790713
CHRM1	Muscarinic acetylcholine receptor M1	0.080	0.064	0.095	0.00000035	0.0000025
CIAPIN1	Anamorsin	0.063	0.047	0.080	0.000146419	0.000649986
CILP	Cartilage intermediate layer protein 1	0.069	0.053	0.086	0.0000303	0.000155293
CIT	Citron Rho-interacting kinase	-0.045	-0.061	-0.029	0.004260575	0.014008617

CKAP4	Cytoskeleton-associated protein 4	0.089	0.073	0.105	2.15E-08	0.000000188
CLC	Galectin-10	0.096	0.080	0.112	2.19E-09	2.27E-08
CLEC11A	C-type lectin domain family 11 member A	0.046	0.029	0.063	0.006697225	0.020914517
CLEC12A	C-type lectin domain family 12 member A	0.057	0.042	0.072	0.00022357	0.000963857
CLEC14A	C-type lectin domain family 14 member A	0.077	0.061	0.093	0.00000166	0.0000105
CLEC1A	C-type lectin domain family 1 member A	0.172	0.159	0.185	8.04E-42	3.91E-39
CLEC2L	C-type lectin domain family 2 member L	0.062	0.046	0.078	0.000140099	0.000628081
CLEC3B	Tetranectin	0.039	0.023	0.054	0.012571218	0.035710079
CLEC4A	C-type lectin domain family 4 member A	0.052	0.036	0.069	0.001457627	0.005325805
CLEC4D	C-type lectin domain family 4 member D	0.171	0.156	0.185	7.2E-31	9.57E-29
CLEC6A	C-type lectin domain family 6 member A	0.158	0.142	0.173	1.03E-24	6.3E-23
CLEC7A	C-type lectin domain family 7 member A	0.116	0.102	0.131	4.46E-16	1.04E-14
CLGN	Calmegin	0.053	0.036	0.069	0.001240812	0.004614369
CLIC5	Chloride intracellular channel protein 5	-0.038	-0.053	-0.023	0.012921347	0.036562533
CLMP	CXADR-like membrane protein	-0.051	-0.066	-0.036	0.00073148	0.002835698
CLPP	ATP-dependent Clp protease proteolytic subunit, mitochondrial	0.064	0.048	0.080	0.0000626	0.000301873
CLPS	Colipase	0.055	0.039	0.072	0.000834692	0.003197648
CLU	Clusterin	0.058	0.042	0.075	0.000378193	0.001563589
CLUL1	Clusterin-like protein 1	0.157	0.141	0.173	1.32E-23	6.91E-22
CNST	Consortin	0.055	0.039	0.072	0.000817024	0.003138188
CNTF	Ciliary neurotrophic factor	0.069	0.052	0.085	0.000034	0.000172885
CNTN1	Contactin-1	0.079	0.063	0.095	0.000000405	0.00000287
CNTN4	Contactin-4	0.114	0.098	0.130	2.62E-12	3.87E-11
CNTN5	Contactin-5	0.106	0.090	0.122	7.03E-11	8.71E-10
CNTNAP4	Contactin-associated protein-like 4	0.082	0.067	0.096	2.99E-08	0.000000255
COCH	Cochlin	0.084	0.068	0.099	0.000000053	0.000000431
COL1A1	Collagen alpha-1(I) chain	0.129	0.115	0.143	8.56E-20	2.88E-18
COL28A1	Collagen alpha-1(XXVIII) chain	0.082	0.066	0.098	0.000000259	0.00000189
COL4A4	Collagen alpha-4(IV) chain	0.088	0.073	0.103	1.05E-08	9.67E-08
COL6A3	Collagen alpha-3(VI) chain	0.111	0.097	0.126	7.23E-15	1.47E-13
COL9A1	Collagen alpha-1(IX) chain	0.097	0.081	0.112	2.79E-10	3.28E-09
COL9A2	Collagen alpha-2(IX) chain	0.056	0.039	0.072	0.000661948	0.00257983
COPB2	Coatomer subunit beta'	0.200	0.186	0.215	2.8E-44	1.64E-41
CORO1A	Coronin-1A	0.045	0.028	0.061	0.007102538	0.022015609
CORO6	Coronin-6	0.089	0.073	0.105	2.13E-08	0.000000187
CPA1	Carboxypeptidase A1	0.055	0.039	0.072	0.00086862	0.003318922
CPA2	Carboxypeptidase A2	0.074	0.059	0.090	0.00000132	0.00000853
CPB2	Carboxypeptidase B2	-0.089	-0.106	-0.072	0.000000124	0.000000941
CPLX2	Complexin-2	0.056	0.040	0.072	0.000485723	0.001955605
CPM	Carboxypeptidase M	-0.058	-0.073	-0.042	0.000174105	0.000762533
CPPED1	Serine/threonine-protein phosphatase CPPED1	0.041	0.025	0.058	0.011679138	0.0335345
CPTP	Ceramide-1-phosphate transfer protein	0.052	0.038	0.066	0.000198149	0.000856789
CPVL	Probable serine carboxypeptidase CPVL	0.182	0.167	0.197	6.71E-33	1.09E-30
CPXM1	Probable carboxypeptidase X1	0.039	0.022	0.056	0.018365069	0.049023832
CPXM2	Inactive carboxypeptidase-like protein X2	0.085	0.069	0.101	0.00000013	0.000000978
CRACR2A	EF-hand calcium-binding domain-containing protein 4B	0.152	0.136	0.167	4.44E-22	1.94E-20
CRADD	Death domain-containing protein CRADD	0.045	0.028	0.061	0.006922121	0.021547774
CREB3	Cyclic AMP-responsive element-binding protein 3	0.063	0.046	0.079	0.000139852	0.00062794
CRELD1	Protein disulfide isomerase CRELD1	-0.076	-0.092	-0.059	0.00000516	0.0000303
CRHBP	Corticotropin-releasing factor-binding protein	0.065	0.049	0.081	0.0000632	0.000304305
CRHR1	Corticotropin-releasing factor receptor 1	0.066	0.050	0.083	0.000079	0.000375297
CRIP2	Cysteine-rich protein 2	0.072	0.055	0.088	0.0000108	0.0000603
CRISP3	Cysteine-rich secretory protein 3	0.071	0.054	0.087	0.0000119	0.0000652
CRLF1	Cytokine receptor-like factor 1	0.076	0.060	0.092	0.00000155	0.0000099
CRNN	Cornulin	-0.097	-0.112	-0.083	1.31E-11	1.83E-10
CRTAC1	Cartilage acidic protein 1	-0.033	-0.047	-0.019	0.017613136	0.047319114
CRTAP	Cartilage-associated protein	0.161	0.146	0.176	3.73E-27	3.9E-25
CRX	Cone-rod homeobox protein	0.054	0.040	0.068	0.000130271	0.000588537
CRYBB1	Beta-crystallin B1	0.205	0.190	0.220	1.3E-40	5.42E-38
CRYBB2	Beta-crystallin B2	0.143	0.128	0.158	1.21E-20	4.33E-19
CRYGD	Gamma-crystallin D	0.184	0.169	0.199	2.26E-34	4.72E-32
CRYM	Ketimine reductase mu-crystallin	0.103	0.087	0.119	1.06E-10	1.29E-09
CSF1	Macrophage colony-stimulating factor 1	0.049	0.032	0.066	0.003615919	0.01213471
CSF2	Granulocyte-macrophage colony-stimulating factor	0.072	0.056	0.088	0.00000385	0.0000232
CSF2RB	Cytokine receptor common subunit beta	0.070	0.054	0.086	0.000008	0.0000451
CSF3	Granulocyte colony-stimulating factor	0.071	0.055	0.086	0.00000541	0.0000317
CSF3R	Granulocyte colony-stimulating factor receptor	0.075	0.059	0.092	0.00000532	0.0000312
CSNK1D	Casein kinase I isoform delta	0.072	0.056	0.089	0.00000766	0.0000435
CSNK2A1	Casein kinase II subunit alpha	0.065	0.048	0.081	0.0000815	0.000386247
CSPG4	Chondroitin sulfate proteoglycan 4	0.075	0.058	0.091	0.00000551	0.0000322
CSPG5	Chondroitin sulfate proteoglycan 5	0.068	0.052	0.083	0.0000137	0.0000743
CSRP3	Cysteine and glycine-rich protein 3	0.058	0.041	0.075	0.000632454	0.002480594
CST1	Cystatin-SN	0.073	0.057	0.090	0.00000953	0.0000531
CST3	Cystatin-C	0.040	0.024	0.056	0.01130158	0.032642806
CST7	Cystatin-F	-0.084	-0.100	-0.069	0.000000072	0.000000568
CSTB	Cystatin-B	0.082	0.066	0.098	0.000000231	0.0000017
CTAG1A_CTAG1B	Cancer/testis antigen 1	0.072	0.056	0.087	0.0000038	0.000023
CTBS	Di-N-acetylchitobiase	0.131	0.116	0.146	3.3E-18	1.01E-16
CTHRC1	Collagen triple helix repeat-containing protein 1	0.133	0.116	0.149	6.87E-16	1.58E-14
CTRC	Chymotrypsin-C	0.045	0.028	0.061	0.005551675	0.017773871
CTRL	Chymotrypsin-like protease CTRL-1	-0.049	-0.065	-0.033	0.002030813	0.007195233
CTSB	Cathepsin B	0.088	0.073	0.103	8.42E-09	7.86E-08
CTSD	Cathepsin D	0.134	0.120	0.149	4.86E-21	1.84E-19
CTSF	Cathepsin F	0.046	0.029	0.063	0.006290259	0.019812961
CTSS	Cathepsin S	0.139	0.123	0.155	6.89E-18	1.97E-16
CX3CL1	Fractalkine	0.043	0.027	0.059	0.008155678	0.02475498
CXCL1	Growth-regulated alpha protein	0.068	0.051	0.084	0.0000357	0.000179475
CXCL11	C-X-C motif chemokine 11	0.050	0.033	0.066	0.002448643	0.008551236
CXCL12	Stromal cell-derived factor 1	0.197	0.182	0.212	1.26E-37	3.69E-35
CXCL14	C-X-C motif chemokine 14	0.062	0.046	0.078	0.000146542	0.000649986
CXCL8	Interleukin-8	0.114	0.100	0.129	5.58E-15	1.14E-13
DAG1	Dystroglycan	0.099	0.084	0.115	3.4E-10	3.93E-09
DAND5	DAN domain family member 5	-0.067	-0.083	-0.051	0.0000255	0.00013129
DAPK2	Death-associated protein kinase 2	0.159	0.144	0.175	3.44E-25	2.14E-23
DBH	Dopamine beta-hydroxylase	-0.067	-0.082	-0.051	0.0000124	0.0000676
DBI	Acyl-CoA-binding protein	0.060	0.044	0.076	0.000173946	0.000762533
DCBLD2	Discoidin, CUB and LCCL domain-containing protein 2	0.072	0.056	0.087	0.00000606	0.0000348
DCLRE1C	Protein artemis	0.047	0.030	0.063	0.004759091	0.015456469
DCTN2	Dynactin subunit 2	0.040	0.025	0.056	0.009990145	0.02928906
DCTN6	Dynactin subunit 6	0.064	0.047	0.081	0.00014129	0.000631931
DCTPP1	dCTP pyrophosphatase 1	0.045	0.028	0.062	0.00793113	0.024173818
DDR1	Epithelial discoidin domain-containing receptor 1	-0.066	-0.082	-0.050	0.0000267	0.000137112
DDT	D-dopachrome decarboxylase	-0.075	-0.090	-0.059	0.00000227	0.0000141
DDX58	Antiviral innate immune response receptor RIG-I	0.066	0.050	0.082	0.0000467	0.000232039
DECR1	2,4-dienoyl-CoA reductase, mitochondrial	0.127	0.111	0.143	1.78E-15	3.84E-14
DEFB104A_DEFB1(	Beta-defensin 104	0.054	0.038	0.071	0.000815892	0.003137965
DEFB118	Beta-defensin 118	0.064	0.048	0.079	0.0000608	0.00029437

DENND2B	DENN domain-containing protein 2B	0.115	0.100	0.130	3.28E-15	6.79E-14
DENR	Density-regulated protein	0.069	0.053	0.085	0.0000199	0.000105367
DGCR6	Protein DGCR6	0.043	0.026	0.060	0.009419561	0.028066643
DGKA	Diacylglycerol kinase alpha	0.092	0.076	0.107	2.23E-09	2.31E-08
DHPS	Deoxyhypusine synthase	0.057	0.041	0.073	0.000346025	0.001442841
DHRS4L2	Dehydrogenase/reductase SDR family member 4-like 2	0.158	0.143	0.174	2.3E-25	1.53E-23
DIABLO	Diablo homolog, mitochondrial	0.071	0.054	0.087	0.0000235	0.000121923
DIPK1C	Divergent protein kinase domain 1C	0.126	0.109	0.142	1.81E-14	3.46E-13
DIPK2B	Divergent protein kinase domain 2B	0.067	0.051	0.082	0.0000215	0.000112443
DKK3	Dickkopf-related protein 3	0.051	0.035	0.068	0.002163213	0.007590724
DKK4	Dickkopf-related protein 4	0.042	0.025	0.058	0.010800463	0.031443977
DLG4	Disks large homolog 4	0.038	0.022	0.053	0.014450352	0.040303797
DLGAP5	Disks large-associated protein 5	0.157	0.141	0.173	3.32E-22	1.47E-20
DLK1	Protein delta homolog 1	0.067	0.050	0.083	0.0000433	0.000216142
DLL1	Delta-like protein 1	0.053	0.037	0.070	0.000968288	0.003666199
DMP1	Dentin matrix acidic phosphoprotein 1	0.188	0.178	0.198	4.85E-81	1.42E-77
DNAJA1	DnaJ homolog subfamily A member 1	-0.050	-0.066	-0.035	0.001291964	0.004786326
DNAJA2	DnaJ homolog subfamily A member 2	0.085	0.070	0.100	3.21E-08	0.000000271
DNAJB1	DnaJ homolog subfamily B member 1	0.101	0.086	0.116	1.88E-11	2.54E-10
DNAJB2	DnaJ homolog subfamily B member 2	0.043	0.026	0.059	0.009654726	0.028592465
DNAJB6	DnaJ homolog subfamily B member 6	0.153	0.139	0.168	1.94E-26	1.62E-24
DNAJB8	DnaJ homolog subfamily B member 8	0.114	0.098	0.130	2.29E-12	3.43E-11
DNLZ	DNL-type zinc finger protein	0.089	0.074	0.105	1.75E-08	0.000000155
DNM1	Dynamin-1	0.159	0.143	0.174	2.95E-24	1.72E-22
DNM3	Dynamin-3	0.086	0.070	0.102	5.63E-08	0.000000456
DNMBP	Dynamin-binding protein	0.086	0.070	0.102	0.000000106	0.000000811
DNPEP	Aspartyl aminopeptidase	0.131	0.115	0.146	2.8E-17	7.59E-16
DOK1	Docking protein 1	0.091	0.074	0.107	2.67E-08	0.000000232
DOK2	Docking protein 2	0.108	0.092	0.125	1.45E-11	2.01E-10
DPEP2	Dipeptidase 2	0.086	0.070	0.101	7.39E-08	0.000000581
DPP10	Inactive dipeptidyl peptidase 10	0.039	0.023	0.056	0.01697339	0.046016924
DPP4	Dipeptidyl peptidase 4	0.055	0.039	0.072	0.000546704	0.002177132
DPP6	Dipeptidyl aminopeptidase-like protein 6	-0.079	-0.095	-0.062	0.00000163	0.0000103
DPT	Dermatopontin	0.050	0.034	0.066	0.001573292	0.005677447
DPY30	Protein dpy-30 homolog	0.041	0.025	0.058	0.010855664	0.031510531
DRG2	Developmentally-regulated GTP-binding protein 2	0.110	0.097	0.124	7.47E-16	1.65E-14
DSC2	Desmocollin-2	0.115	0.099	0.131	8.81E-13	1.37E-11
DSCAM	Down syndrome cell adhesion molecule	0.106	0.090	0.122	4.4E-11	5.66E-10
DSG4	Desmoglein-4	0.082	0.066	0.099	0.000000429	0.00000302
DTNB	Dystrobrevin beta	0.055	0.039	0.072	0.00081535	0.003137965
DTX2	Probable E3 ubiquitin-protein ligase DTX2	0.107	0.091	0.123	6.27E-11	7.9E-10
DUSP3	Dual specificity protein phosphatase 3	0.089	0.073	0.105	1.66E-08	0.000000148
ECHS1	Enoyl-CoA hydratase, mitochondrial	0.136	0.120	0.152	1.67E-17	4.57E-16
ECM1	Extracellular matrix protein 1	0.161	0.145	0.177	1.07E-23	5.69E-22
EDEM2	ER degradation-enhancing alpha-mannosidase-like protein 2	0.037	0.021	0.052	0.018340103	0.049023832
EDF1	Endothelial differentiation-related factor 1	0.065	0.048	0.081	0.000103075	0.000478233
EDN1	Endothelin-1	0.087	0.071	0.103	0.0000001	0.000000769
EFCAB14	EF-hand calcium-binding domain-containing protein 14	0.050	0.034	0.066	0.001550607	0.005602503
EFNA1	Ephrin-A1	0.040	0.024	0.057	0.015645963	0.042955508
EFNA4	Ephrin-A4	-0.061	-0.077	-0.045	0.000120084	0.000549304
EGF	Pro-epidermal growth factor	0.054	0.038	0.071	0.001055293	0.003969911
EGFR	Epidermal growth factor receptor	0.112	0.096	0.127	2.47E-13	4.2E-12
EGLN1	Egl nine homolog 1	0.062	0.047	0.077	0.0000231	0.000119704
EIF2AK2	Interferon-induced, double-stranded RNA-activated protein kinase	0.040	0.024	0.057	0.01579647	0.043233222
EIF2AK3	Eukaryotic translation initiation factor 2-alpha kinase 3	0.040	0.023	0.056	0.01667008	0.045284986
EIF2S2	Eukaryotic translation initiation factor 2 subunit 2	0.058	0.041	0.074	0.00047515	0.00191832
EIF4E	Eukaryotic translation initiation factor 4E	0.037	0.022	0.052	0.015590663	0.042870657
EIF4EBP1	Eukaryotic translation initiation factor 4E-binding protein 1	0.083	0.068	0.099	6.28E-08	0.000000506
EIF4G1	Eukaryotic translation initiation factor 4 gamma 1	0.044	0.027	0.060	0.009668002	0.028602804
EIF4G3	Eukaryotic translation initiation factor 4 gamma 3	0.110	0.094	0.126	1.59E-11	2.17E-10
EIF5	Eukaryotic translation initiation factor 5	0.157	0.142	0.172	4.67E-26	3.48E-24
ELAVL4	ELAV-like protein 4	0.123	0.108	0.139	8.94E-15	1.79E-13
ELN	Elastin	0.072	0.056	0.088	0.0000112	0.0000621
ELOA	Elongin-A	0.065	0.049	0.081	0.0000647	0.000310783
ENAH	Protein enabled homolog	0.039	0.023	0.054	0.014153593	0.039665343
ENG	Endoglin	0.049	0.033	0.066	0.002919521	0.010004408
ENO2	Gamma-enolase	0.082	0.066	0.098	0.000000511	0.00000355
ENPEP	Glutamyl aminopeptidase	0.146	0.131	0.161	2.8E-22	1.28E-20
ENPP7	Ectonucleotide pyrophosphatase/phosphodiesterase family member 7	0.063	0.047	0.079	0.0000826	0.000390445
ENTPD2	Ectonucleoside triphosphate diphosphohydrolase 2	0.076	0.060	0.092	0.00000142	0.00000918
ENTPD6	Ectonucleoside triphosphate diphosphohydrolase 6	0.073	0.057	0.089	0.0000058	0.0000336
ENTR1	Endosome-associated-trafficking regulator 1	0.186	0.171	0.202	1.33E-31	1.95E-29
EPHA1	Ephrin type-A receptor 1	0.074	0.058	0.090	0.00000371	0.0000225
EPHA4	Ephrin type-A receptor 4	-0.074	-0.090	-0.058	0.00000352	0.0000215
EPO	Erythropoietin	0.061	0.045	0.077	0.00014139	0.000631931
ERBB3	Receptor tyrosine-protein kinase erbB-3	0.106	0.090	0.122	3.08E-11	4.02E-10
ERBB4	Receptor tyrosine-protein kinase erbB-4	0.066	0.050	0.082	0.00005	0.000247311
EREG	Proepiregulin	0.114	0.099	0.129	2.38E-14	4.43E-13
ERP44	Endoplasmic reticulum resident protein 44	0.117	0.101	0.133	4.56E-13	7.44E-12
ERVV-1	Endogenous retrovirus group V member 1 Env polyprotein	0.074	0.057	0.090	0.00000857	0.0000482
ESM1	Endothelial cell-specific molecule 1	0.040	0.023	0.056	0.017163577	0.046367038
ESR1	Estrogen receptor	0.135	0.119	0.150	3.33E-18	1.01E-16
EVI2B	Protein EVI2B	0.110	0.094	0.125	2.37E-12	3.54E-11
EVPL	Envoplakin	0.101	0.086	0.116	1.5E-11	2.06E-10
EXOSC10	Exosome component 10	0.106	0.090	0.122	1.12E-11	1.58E-10
EXTL1	Exostosin-like 1	0.119	0.102	0.135	3.63E-13	5.98E-12
EZR	Ezrin	0.158	0.144	0.172	4.17E-29	5.07E-27
F13B	Coagulation factor XIII B chain	0.070	0.054	0.086	0.0000154	0.0000829
F2	Prothrombin	0.075	0.059	0.091	0.00000213	0.0000133
F2R	Proteinase-activated receptor 1	0.088	0.072	0.103	3.92E-08	0.000000328
FABP1	Fatty acid-binding protein, liver	0.071	0.055	0.087	0.0000121	0.0000662
FABP4	Fatty acid-binding protein, adipocyte	0.131	0.116	0.146	4.71E-18	1.39E-16
FABP5	Fatty acid-binding protein 5	0.054	0.038	0.070	0.000859907	0.003289932
FABP6	Gastrotropin	-0.036	-0.051	-0.021	0.016986747	0.046016924
FADD	FAS-associated death domain protein	0.100	0.083	0.116	6.72E-10	7.47E-09
FAM171B	Protein FAM171B	0.055	0.038	0.071	0.000905741	0.00344724
FAM3D	Protein FAM3D	0.107	0.092	0.122	1.06E-12	1.64E-11
FAP	Prolyl endopeptidase FAP	0.052	0.036	0.069	0.001603946	0.00577381
FBLN2	Fibulin-2	0.079	0.063	0.096	0.00000107	0.00000701
FBN2	Fibrillin-2	0.087	0.071	0.103	5.06E-08	0.000000413
FBP1	Fructose-1,6-bisphosphatase 1	0.137	0.121	0.153	9.44E-18	2.63E-16
FCAMR	High affinity immunoglobulin alpha and immunoglobulin mu Fc receptor	0.125	0.110	0.140	4.66E-17	1.18E-15
FCAR	Immunoglobulin alpha Fc receptor	0.091	0.075	0.107	1.55E-08	0.000000138
FCGR2B	Low affinity immunoglobulin gamma Fc region receptor II-b	0.080	0.064	0.096	0.000000492	0.00000344
FCN1	Ficolin-1	0.133	0.116	0.149	4.36E-16	1.03E-14
FCN2	Ficolin-2	0.104	0.089	0.119	6.67E-12	9.51E-11
FCRL3	Fc receptor-like protein 3	0.059	0.043	0.075	0.000299767	0.00125713

FCRL5	Fc receptor-like protein 5	0.056	0.040	0.072	0.000576517	0.002286511
FDX1	Adrenodoxin, mitochondrial	0.070	0.055	0.085	0.00000426	0.0000254
FDX2	Ferredoxin-2, mitochondrial	0.068	0.052	0.084	0.000026	0.000133671
FEN1	Flap endonuclease 1	0.043	0.027	0.060	0.008856547	0.026551474
FES	Tyrosine-protein kinase Fes/Fps	0.050	0.034	0.067	0.001895692	0.006765698
FETUB	Fetuin-B	0.041	0.024	0.057	0.014999426	0.041636583
FGA	Fibrinogen alpha chain	0.056	0.040	0.073	0.000616957	0.002430412
FGD3	FYVE, RhoGEF and PH domain-containing protein 3	0.054	0.038	0.071	0.000949867	0.003605794
FGF16	Fibroblast growth factor 16	0.053	0.037	0.070	0.001070811	0.004023111
FGF3	Fibroblast growth factor 3	0.056	0.040	0.073	0.000694988	0.002704995
FGF7	Fibroblast growth factor 7	0.136	0.122	0.150	6.83E-22	2.77E-20
FGF9	Fibroblast growth factor 9	0.050	0.033	0.066	0.002189144	0.007672504
FGFBP1	Fibroblast growth factor-binding protein 1	0.038	0.023	0.054	0.014074937	0.039482766
FGFBP2	Fibroblast growth factor-binding protein 2	0.117	0.101	0.133	1.05E-13	1.83E-12
FGFBP3	Fibroblast growth factor-binding protein 3	0.106	0.090	0.121	6.03E-12	8.65E-11
FGFR4	Fibroblast growth factor receptor 4	0.064	0.049	0.080	0.0000227	0.000118159
FGL1	Fibrinogen-like protein 1	0.096	0.080	0.112	1.33E-09	1.43E-08
FGR	Tyrosine-protein kinase Fgr	0.088	0.072	0.105	4.88E-08	0.000000399
FHIP2A	FHF complex subunit HOOK interacting protein 2A	0.040	0.024	0.056	0.011279568	0.032611451
FHIT	Bis(5'-adenosyl)-triphosphatase	0.116	0.100	0.132	2.89E-13	4.88E-12
FKBP14	Peptidyl-prolyl cis-trans isomerase FKBP14	0.123	0.107	0.139	1.45E-14	2.83E-13
FKBP4	Peptidyl-prolyl cis-trans isomerase FKBP4	-0.052	-0.067	-0.038	0.000291602	0.001228172
FKBP5	Peptidyl-prolyl cis-trans isomerase FKBP5	0.142	0.128	0.156	3.1E-24	1.78E-22
FKBP7	Peptidyl-prolyl cis-trans isomerase FKBP7	0.094	0.077	0.110	8.25E-09	7.72E-08
FKBPL	FK506-binding protein-like	0.152	0.137	0.167	5.88E-24	3.31E-22
FLI1	Friend leukemia integration 1 transcription factor	0.077	0.061	0.092	0.000000959	0.00000637
FMNL1	Formin-like protein 1	0.119	0.104	0.135	1.45E-14	2.83E-13
FMR1	Synaptic functional regulator FMR1	0.047	0.030	0.063	0.004936994	0.015928073
FN1	Fibronectin	-0.041	-0.058	-0.024	0.013915678	0.039111084
FOLH1	Glutamate carboxypeptidase 2	-0.053	-0.070	-0.036	0.001534353	0.005557514
FOLR3	Folate receptor gamma	0.077	0.061	0.093	0.00000168	0.0000106
FOS	Proto-oncogene c-Fos	0.078	0.062	0.095	0.00000203	0.0000127
FOSB	Protein fosB	-0.121	-0.135	-0.107	7.37E-18	2.09E-16
FOXO1	Forkhead box protein O1	0.045	0.028	0.062	0.006868917	0.021404952
FST	Follistatin	0.043	0.026	0.059	0.009111472	0.027259808
FSTL3	Follistatin-related protein 3	0.045	0.028	0.062	0.007214077	0.022314017
FTCD	Formimidoyltransferase-cyclodeaminase	0.090	0.075	0.105	1.05E-09	1.14E-08
FUOM	Fucose mutarotase	0.064	0.047	0.081	0.000133997	0.000604436
FUT1	Galactoside alpha-(1,2)-fucosyltransferase 1	0.048	0.031	0.065	0.004117187	0.013583194
FZD10	Frizzled-10	0.059	0.043	0.075	0.000275745	0.001169814
GABARAPL1	Gamma-aminobutyric acid receptor-associated protein-like 1	0.085	0.069	0.101	0.000000178	0.00000133
GAD1	Glutamate decarboxylase 1	0.064	0.050	0.078	0.00000277	0.000017
GADD45B	Growth arrest and DNA damage-inducible protein GADD45 beta	0.047	0.031	0.064	0.003906321	0.012989963
GADD45GIP1	Growth arrest and DNA damage-inducible proteins-interacting protein 1	0.082	0.065	0.099	0.000000912	0.00000611
GAGE2A	G antigen 2A	0.041	0.025	0.057	0.011793585	0.033730577
GAL	Galanin peptides	0.057	0.041	0.073	0.000459498	0.001862846
GAMT	Guanidinoacetate N-methyltransferase	0.142	0.130	0.154	3.21E-33	5.51E-31
GAS2	Growth arrest-specific protein 2	0.161	0.146	0.177	5.88E-26	4.19E-24
GASK1A	Golgi-associated kinase 1A	0.048	0.032	0.065	0.004160278	0.013705178
GAST	Gastrin	0.092	0.076	0.108	7.4E-09	6.95E-08
GBA	Lysosomal acid glucosylceramidase	0.056	0.040	0.071	0.000425255	0.001743367
GBP2	Guanylate-binding protein 2	0.051	0.034	0.067	0.00215788	0.007590234
GBP6	Guanylate-binding protein 6	0.074	0.058	0.090	0.00000271	0.0000167
GCHFR	GTP cyclohydrolase 1 feedback regulatory protein	0.042	0.025	0.058	0.011363414	0.032756664
GCLM	Glutamate--cysteine ligase regulatory subunit	0.044	0.028	0.061	0.006696428	0.020914517
GCNT1	Beta-1,3-galactosyl-O-glycosyl-glycoprotein beta-1,6-N-acetylglucosaminyltransferase	0.088	0.072	0.104	4.17E-08	0.000000345
GFRA2	GDNF family receptor alpha-2	0.174	0.160	0.189	2.55E-33	4.66E-31
GFRAL	GDNF family receptor alpha-like	0.050	0.033	0.067	0.002686539	0.009337401
GGACT	Gamma-glutamylaminecyclotransferase	0.058	0.042	0.074	0.000364992	0.001511148
GGCT	Gamma-glutamylcyclotransferase	0.138	0.123	0.152	2.9E-21	1.14E-19
GGT1	Glutathione hydrolase 1 proenzyme	0.065	0.049	0.081	0.0000554	0.00027171
GGT5	Glutathione hydrolase 5 proenzyme	0.070	0.054	0.086	0.0000155	0.0000835
GH1	Somatotropin	0.089	0.073	0.105	1.87E-08	0.000000165
GH2	Growth hormone variant	0.050	0.035	0.065	0.001125373	0.004211864
GHR	Growth hormone receptor	0.139	0.125	0.154	2.38E-22	1.1E-20
GHRHR	Growth hormone-releasing hormone receptor	0.088	0.073	0.102	4.07E-09	3.98E-08
GHRL	Appetite-regulating hormone	0.049	0.034	0.065	0.001511	0.00550705
GID8	Glucose-induced degradation protein 8 homolog	0.067	0.051	0.084	0.0000336	0.0001711
GIGYF2	GRB10-interacting GYF protein 2	-0.047	-0.063	-0.031	0.003727931	0.01246767
GIPC3	PDZ domain-containing protein GIPC3	0.077	0.061	0.094	0.0000032	0.0000196
GIPR	Gastric inhibitory polypeptide receptor	0.065	0.049	0.081	0.0000341	0.000172885
GJA8	Gap junction alpha-8 protein	0.082	0.066	0.098	0.000000294	0.00000214
GKN1	Gastrokine-1	0.048	0.033	0.064	0.001956511	0.006957276
GLI2	Zinc finger protein GLI2	0.070	0.053	0.086	0.0000304	0.000155602
GLO1	Lactoylglutathione lyase	0.066	0.049	0.082	0.000058	0.000282706
GLOD4	Glyoxalase domain-containing protein 4	0.092	0.077	0.108	2.63E-09	2.67E-08
GLP1R	Glucagon-like peptide 1 receptor	0.061	0.045	0.077	0.000165618	0.000731272
GLRX	Glutaredoxin-1	0.073	0.059	0.088	0.000000349	0.0000025
GLYR1	Putative oxidoreductase GLYR1	0.049	0.033	0.066	0.002792371	0.009659292
GM2A	Ganglioside GM2 activator	0.048	0.032	0.065	0.003727001	0.01246767
GMPR2	GMP reductase 2	0.048	0.031	0.064	0.003812842	0.01270804
GNE	Bifunctional UDP-N-acetylglucosamine 2-epimerase/N-acetylmannosamine kinase	0.040	0.024	0.057	0.01429869	0.040033592
GNGT1	Guanine nucleotide-binding protein G(T) subunit gamma-T1	0.064	0.049	0.079	0.0000207	0.000108769
GNPDA1	Glucosamine-6-phosphate isomerase 1	0.127	0.112	0.142	3.62E-17	9.54E-16
GNPDA2	Glucosamine-6-phosphate isomerase 2	0.065	0.049	0.082	0.0000712	0.000339996
GOLM2	Protein GOLM2	0.062	0.046	0.078	0.000117869	0.000540391
GORASP2	Golgi reassembly-stacking protein 2	-0.165	-0.181	-0.149	1.75E-25	1.19E-23
GOT1	Aspartate aminotransferase, cytoplasmic	-0.104	-0.119	-0.088	2.11E-11	2.82E-10
GP1BB	Platelet glycoprotein Ib beta chain	0.095	0.080	0.110	2.91E-10	3.39E-09
GP6	Platelet glycoprotein VI	0.088	0.072	0.104	3.65E-08	0.000000307
GPC1	Glypican-1	0.153	0.137	0.168	1.45E-23	7.43E-22
GPC5	Glypican-5	0.061	0.044	0.077	0.000199375	0.000860818
GPHA2	Glycoprotein hormone alpha-2	0.110	0.094	0.125	3.34E-13	5.6E-12
GPIHBP1	Glycosylphosphatidylinositol-anchored high density lipoprotein-binding protein 1	0.051	0.035	0.068	0.001944723	0.006923781
GPKOW	G-patch domain and KOW motifs-containing protein	0.076	0.060	0.092	0.00000263	0.0000162
GPR101	Probable G-protein coupled receptor 101	0.064	0.049	0.078	0.0000123	0.0000674
GPR158	Probable G-protein coupled receptor 158	0.056	0.040	0.073	0.000533705	0.002131175
GPR15L	Protein GPR15L	0.078	0.062	0.095	0.00000193	0.0000121
GRHPR	Glyoxylate reductase/hydroxypyruvate reductase	0.062	0.046	0.078	0.000123188	0.000561747
GRIN2B	Glutamate receptor ionotropic, NMDA 2B	0.091	0.075	0.107	1.52E-08	0.000000135
GRN	Progranulin	0.094	0.078	0.109	1.59E-09	1.68E-08
GRPEL1	GrpE protein homolog 1, mitochondrial	0.076	0.061	0.092	0.000000442	0.00000311
GSAP	Gamma-secretase-activating protein	0.122	0.106	0.137	5.23E-15	1.08E-13
GSR	Glutathione reductase, mitochondrial	0.040	0.024	0.056	0.013203211	0.037251916
GSTA1	Glutathione S-transferase A1	0.048	0.031	0.064	0.003415591	0.011595553
GSTT2B	Glutathione S-transferase theta-2B	0.090	0.074	0.107	2.63E-08	0.000000229
GTF2IRD1	General transcription factor II-I repeat domain-containing protein 1	0.155	0.139	0.171	2.4E-23	1.19E-21

GTPBP2	GTP-binding protein 2	0.103	0.087	0.119	9.6E-11	1.18E-09
GUK1	Guanylate kinase	0.041	0.024	0.057	0.012915695	0.036562533
GUSB	Beta-glucuronidase	0.106	0.091	0.122	6.97E-12	9.89E-11
GYS1	Glycogen [starch] synthase, muscle	0.057	0.040	0.073	0.000518258	0.002080862
GZMB	Granzyme B	0.044	0.028	0.061	0.005666119	0.018051032
HADH	Hydroxyacyl-coenzyme A dehydrogenase, mitochondrial	0.070	0.055	0.086	0.00000602	0.0000347
HARS1	Histidine--tRNA ligase, cytoplasmic	0.071	0.055	0.088	0.0000187	0.0000992
HAVCR1	Hepatitis A virus cellular receptor 1	0.045	0.028	0.062	0.007045035	0.021861352
HAVCR2	Hepatitis A virus cellular receptor 2	0.063	0.046	0.079	0.000135677	0.00061107
HBEGF	Proheparin-binding EGF-like growth factor	0.081	0.064	0.098	0.000000999	0.00000662
HCG22	Protein PBMUCL2	-0.040	-0.057	-0.023	0.015938778	0.043507695
HCLS1	Hematopoietic lineage cell-specific protein	0.039	0.024	0.054	0.009799785	0.028875779
HDAC8	Histone deacetylase 8	0.050	0.033	0.067	0.002972108	0.010172684
HDGFL2	Hepatoma-derived growth factor-related protein 2	0.114	0.098	0.130	4.89E-13	7.85E-12
HEG1	Protein HEG homolog 1	0.077	0.063	0.091	6.95E-08	0.000000555
HEPACAM2	HEPACAM family member 2	0.130	0.115	0.145	5.54E-18	1.62E-16
HEPH	Hephaestin	0.043	0.027	0.059	0.008258846	0.025016174
HGFAC	Hepatocyte growth factor activator	0.041	0.025	0.058	0.011589368	0.033342248
HGS	Hepatocyte growth factor-regulated tyrosine kinase substrate	0.059	0.044	0.074	0.000097	0.000452954
HIP1	Huntingtin-interacting protein 1	0.060	0.044	0.076	0.000197122	0.000853613
HIP1R	Huntingtin-interacting protein 1-related protein	0.113	0.097	0.129	7.03E-13	1.11E-11
HJV	Hemojuvelin	0.045	0.030	0.061	0.003753981	0.012540442
HK2	Hexokinase-2	0.097	0.081	0.114	1.75E-09	1.84E-08
HLA-A	HLA class I histocompatibility antigen, A alpha chain	0.060	0.045	0.075	0.0000679	0.000324858
HLA-DRA	HLA class II histocompatibility antigen, DR alpha chain	0.043	0.026	0.060	0.009485105	0.028233159
HMBS	Porphobilinogen deaminase	0.113	0.097	0.129	8.59E-13	1.35E-11
HMCN2	Hemicentin-2	0.060	0.044	0.076	0.000174547	0.000762631
HMGCS1	Hydroxymethylglutaryl-CoA synthase, cytoplasmic	0.084	0.068	0.099	8.09E-08	0.000000629
HMMR	Hyaluronan mediated motility receptor	0.095	0.079	0.112	5.38E-09	5.13E-08
HMOX1	Heme oxygenase 1	0.074	0.058	0.090	0.00000557	0.0000325
HMOX2	Heme oxygenase 2	0.057	0.041	0.074	0.000405787	0.001670586
HNF1A	Hepatocyte nuclear factor 1-alpha	0.140	0.124	0.155	1.92E-19	6.29E-18
HNMT	Histamine N-methyltransferase	0.193	0.177	0.209	3.13E-34	6.11E-32
HNRNPK	Heterogeneous nuclear ribonucleoprotein K	0.042	0.026	0.058	0.007926358	0.024173818
HPGDS	Hematopoietic prostaglandin D synthase	0.050	0.034	0.067	0.001963137	0.006968688
HRC	Sarcoplasmic reticulum histidine-rich calcium-binding protein	0.047	0.033	0.061	0.000600145	0.002367374
HRG	Histidine-rich glycoprotein	0.095	0.080	0.111	2.96E-10	3.44E-09
HS3ST3B1	Heparan sulfate glucosamine 3-O-sulfotransferase 3B1	0.176	0.160	0.192	9.81E-29	1.15E-26
HSBP1	Heat shock factor-binding protein 1	0.106	0.091	0.121	3.86E-12	5.58E-11
HSD11B1	Corticosteroid 11-beta-dehydrogenase isozyme 1	0.111	0.095	0.127	3.64E-12	5.29E-11
HTRA2	Serine protease HTRA2, mitochondrial	0.073	0.057	0.088	0.00000493	0.0000292
HYAL1	Hyaluronidase-1	0.039	0.023	0.055	0.015772296	0.043207519
ICA1	Islet cell autoantigen 1	0.070	0.055	0.086	0.00000704	0.0000401
ICAM3	Intercellular adhesion molecule 3	0.086	0.070	0.102	6.93E-08	0.000000555
IFI30	Gamma-interferon-inducible lysosomal thiol reductase	0.041	0.025	0.057	0.011314123	0.032646773
IFIT3	Interferon-induced protein with tetratricopeptide repeats 3	0.095	0.079	0.111	2.89E-09	2.91E-08
IFNGR2	Interferon gamma receptor 2	0.087	0.071	0.103	2.76E-08	0.000000238
IFNL1	Interferon lambda-1	0.101	0.086	0.117	6.34E-11	7.95E-10
IFNL2	Interferon lambda-2	0.082	0.065	0.098	0.000000559	0.00000387
IFNLR1	Interferon lambda receptor 1	0.084	0.068	0.100	9.12E-08	0.000000703
IFNW1	Interferon omega-1	0.129	0.113	0.145	7.43E-16	1.65E-14
IFT20	Intraflagellar transport protein 20 homolog	0.079	0.063	0.095	0.00000105	0.00000693
IGBP1	Immunoglobulin-binding protein 1	0.175	0.160	0.191	1.81E-28	2.03E-26
IGDCC3	Immunoglobulin superfamily DCC subclass member 3	0.106	0.089	0.122	5.26E-11	6.66E-10
IGDCC4	Immunoglobulin superfamily DCC subclass member 4	0.108	0.092	0.125	1.68E-11	2.28E-10
IGF1R	Insulin-like growth factor 1 receptor	0.084	0.068	0.099	2.95E-08	0.000000252
IGFBP2	Insulin-like growth factor-binding protein 2	0.235	0.219	0.251	3.28E-48	2.4E-45
IGFBP3	Insulin-like growth factor-binding protein 3	0.157	0.141	0.173	2.06E-23	1.04E-21
IGFBP4	Insulin-like growth factor-binding protein 4	0.060	0.045	0.076	0.000125857	0.000571781
IGFBP6	Insulin-like growth factor-binding protein 6	0.056	0.040	0.073	0.000702191	0.00272577
IGFBP7	Insulin-like growth factor-binding protein 7	0.167	0.152	0.183	1.28E-27	1.39E-25
IGFL4	Insulin growth factor-like family member 4	0.046	0.029	0.062	0.005833464	0.018513807
IGLC2	Immunoglobulin lambda constant 2	0.086	0.070	0.102	7.75E-08	0.000000607
IGLON5	IgLON family member 5	0.167	0.151	0.183	3.58E-26	2.83E-24
IGSF21	Immunoglobulin superfamily member 21	0.061	0.045	0.078	0.000168748	0.000743967
IGSF3	Immunoglobulin superfamily member 3	0.088	0.072	0.103	1.33E-08	0.00000012
IGSF8	Immunoglobulin superfamily member 8	0.064	0.048	0.080	0.0000582	0.000282924
IKBKG	NF-kappa-B essential modulator	0.054	0.037	0.070	0.001075264	0.004034655
IL10RA	Interleukin-10 receptor subunit alpha	0.079	0.063	0.096	0.00000111	0.00000723
IL10RB	Interleukin-10 receptor subunit beta	0.090	0.074	0.106	4.16E-08	0.000000345
IL11	Interleukin-11	0.102	0.086	0.119	4.07E-10	4.67E-09
IL12B	Interleukin-12 subunit beta	0.127	0.112	0.142	4.36E-17	1.13E-15
IL12RB1	Interleukin-12 receptor subunit beta-1	0.101	0.086	0.117	3.04E-11	3.99E-10
IL13	Interleukin-13	0.122	0.107	0.137	6.13E-16	1.42E-14
IL13RA1	Interleukin-13 receptor subunit alpha-1	0.092	0.077	0.107	9.06E-10	9.95E-09
IL13RA2	Interleukin-13 receptor subunit alpha-2	0.132	0.117	0.147	9.28E-19	2.98E-17
IL15	Interleukin-15	0.112	0.096	0.128	2.54E-12	3.77E-11
IL15RA	Interleukin-15 receptor subunit alpha	0.070	0.054	0.087	0.000021	0.000110211
IL16	Pro-interleukin-16	0.094	0.078	0.110	2.35E-09	2.41E-08
IL17A	Interleukin-17A	0.090	0.075	0.106	4.58E-09	4.42E-08
IL17C	Interleukin-17C	0.151	0.136	0.166	2.24E-24	1.34E-22
IL17D	Interleukin-17D	0.116	0.100	0.131	9.17E-14	1.63E-12
IL17F	Interleukin-17F	0.063	0.047	0.079	0.0000897	0.000421393
IL17RA	Interleukin-17 receptor A	0.113	0.098	0.129	3.39E-13	5.66E-12
IL17RB	Interleukin-17 receptor B	0.152	0.137	0.168	3.16E-22	1.42E-20
IL18	Interleukin-18	0.049	0.032	0.065	0.002719767	0.009430462
IL18BP	Interleukin-18-binding protein	0.101	0.085	0.116	1.35E-10	1.62E-09
IL18R1	Interleukin-18 receptor 1	0.053	0.037	0.069	0.000952545	0.003611268
IL18RAP	Interleukin-18 receptor accessory protein	0.056	0.040	0.073	0.00045386	0.001847677
IL1A	Interleukin-1 alpha	0.078	0.062	0.093	0.000000724	0.00000492
IL1B	Interleukin-1 beta	0.154	0.138	0.169	6.88E-23	3.3E-21
IL1R1	Interleukin-1 receptor type 1	0.092	0.076	0.109	2.18E-08	0.00000019
IL20	Interleukin-20	0.128	0.112	0.144	1.43E-15	3.1E-14
IL22	Interleukin-22	-0.040	-0.057	-0.024	0.015164499	0.041935506
IL22RA1	Interleukin-22 receptor subunit alpha-1	0.098	0.083	0.113	9.69E-11	1.19E-09
IL25	Interleukin-25	0.070	0.053	0.086	0.0000215	0.000112443
IL33	Interleukin-33	0.062	0.045	0.078	0.000182393	0.000795725
IL34	Interleukin-34	0.063	0.047	0.080	0.000123548	0.000562508
IL36A	Interleukin-36 alpha	0.052	0.035	0.068	0.001621206	0.005828763
IL36G	Interleukin-36 gamma	0.071	0.054	0.087	0.0000241	0.000124518
IL3RA	Interleukin-3 receptor subunit alpha	0.039	0.023	0.056	0.017592533	0.047307244
IL4R	Interleukin-4 receptor subunit alpha	0.063	0.047	0.079	0.00006	0.000290861
IL5	Interleukin-5	-0.084	-0.099	-0.068	8.35E-08	0.000000647
IL6R	Interleukin-6 receptor subunit alpha	0.043	0.027	0.060	0.009546364	0.02837537
IL6ST	Interleukin-6 receptor subunit beta	0.058	0.041	0.075	0.000572284	0.002275898
ILKAP	Integrin-linked kinase-associated serine/threonine phosphatase 2C	0.057	0.040	0.073	0.000445704	0.001818384
IMPA1	Inositol monophosphatase 1	0.105	0.089	0.121	6.77E-11	8.46E-10

IMPACT	Protein IMPACT	0.042	0.026	0.059	0.010364347	0.030234516
IMPG1	Interphotoreceptor matrix proteoglycan 1	-0.048	-0.064	-0.032	0.002580495	0.008990211
INHBB	Inhibin beta B chain	0.087	0.071	0.103	0.000000055	0.000000446
INHBC	Inhibin beta C chain	0.043	0.027	0.060	0.008644162	0.026093923
INPP5J	Phosphatidylinositol 4,5-bisphosphate 5-phosphatase A	0.146	0.131	0.161	4.91E-22	2.11E-20
INPPL1	Phosphatidylinositol 3,4,5-trisphosphate 5-phosphatase 2	0.052	0.036	0.069	0.001517494	0.005514073
IPCEF1	Interactor protein for cytohesin exchange factors 1	0.199	0.183	0.214	3.66E-38	1.19E-35
IRAG2	Inositol 1,4,5-triphosphate receptor associated 2	0.064	0.047	0.080	0.0000972	0.000452985
IRAK1	Interleukin-1 receptor-associated kinase 1	0.096	0.080	0.112	4.51E-09	4.36E-08
ISM2	Isthmin-2	0.058	0.043	0.073	0.00011733	0.0005398
IST1	IST1 homolog	0.113	0.098	0.128	2.28E-14	4.28E-13
ITGAM	Integrin alpha-M	0.080	0.063	0.096	0.000001	0.00000664
ITGAV	Integrin alpha-V	0.064	0.052	0.077	0.000000312	0.00000225
ITGB1BP2	Integrin beta-1-binding protein 2	0.040	0.024	0.057	0.015502786	0.042669155
ITGB5	Integrin beta-5	0.054	0.037	0.071	0.001112184	0.004167837
ITGB6	Integrin beta-6	0.066	0.050	0.083	0.0000592	0.00028745
ITIH3	Inter-alpha-trypsin inhibitor heavy chain H3	0.045	0.027	0.063	0.011723668	0.033596354
JCHAIN	Immunoglobulin J chain	0.050	0.032	0.067	0.004533862	0.014790713
JPT2	Jupiter microtubule associated homolog 2	0.138	0.121	0.155	9.57E-16	2.09E-14
JUN	Transcription factor AP-1	0.070	0.053	0.087	0.0000573	0.000280478
KAZN	Kazrin	0.057	0.039	0.075	0.001246679	0.004630295
KCNC4	Potassium voltage-gated channel subfamily C member 4	0.187	0.171	0.203	5.54E-31	7.71E-29
KDM3A	Lysine-specific demethylase 3A	0.091	0.075	0.106	5.71E-09	5.42E-08
KDR	Vascular endothelial growth factor receptor 2	0.076	0.059	0.093	0.0000117	0.0000643
KEL	Kell blood group glycoprotein	0.088	0.071	0.106	0.000000307	0.00000222
KHK	Ketohexokinase	0.071	0.054	0.087	0.0000148	0.0000799
KIAA2013	Uncharacterized protein KIAA2013	0.042	0.026	0.058	0.008009021	0.024385801
KIF22	Kinesin-like protein KIF22	0.039	0.023	0.056	0.017713341	0.047544623
KIR3DL1	Killer cell immunoglobulin-like receptor 3DL1	0.048	0.030	0.066	0.008083877	0.024562549
KIT	Mast/stem cell growth factor receptor Kit	0.066	0.048	0.083	0.000190254	0.00082632
KLF4	Krueppel-like factor 4	0.057	0.039	0.074	0.001464243	0.005343299
KLK12	Kallikrein-12	0.067	0.051	0.083	0.0000348	0.000175563
KLK15	Kallikrein-15	-0.043	-0.060	-0.025	0.016490853	0.044858881
KLKB1	Plasma kallikrein	0.073	0.055	0.091	0.0000345	0.000174572
KLRK1	NKG2-D type II integral membrane protein	0.097	0.083	0.112	3.17E-11	4.12E-10
KYNU	Kynureninase	-0.117	-0.131	-0.103	2.86E-17	7.66E-16
LATS1	Serine/threonine-protein kinase LATS1	0.063	0.046	0.081	0.000299402	0.00125713
LEPR	Leptin receptor	0.094	0.076	0.111	0.000000071	0.000000563
LETM1	Mitochondrial proton/calcium exchanger protein	0.053	0.036	0.070	0.001728217	0.00620587
LGALS7_LGALS7B	Galectin-7	0.106	0.089	0.123	3.73E-10	4.3E-09
LGALS9	Galectin-9	0.068	0.051	0.086	0.000061	0.000294836
LHPP	Phospholysine phosphohistidine inorganic pyrophosphate phosphatase	0.074	0.057	0.092	0.0000171	0.0000916
LIFR	Leukemia inhibitory factor receptor	0.048	0.030	0.065	0.006577771	0.020585465
LILRA3	Leukocyte immunoglobulin-like receptor subfamily A member 3	0.063	0.046	0.080	0.00016399	0.000726277
LPL	Lipoprotein lipase	0.117	0.100	0.134	1.29E-11	1.8E-10
LRCH4	Leucine-rich repeat and calponin homology domain-containing protein 4	0.050	0.032	0.068	0.005643967	0.018010171
LRIG1	Leucine-rich repeats and immunoglobulin-like domains protein 1	0.094	0.078	0.109	3.67E-09	3.61E-08
LRTM2	Leucine-rich repeat and transmembrane domain-containing protein 2	0.042	0.025	0.060	0.017811816	0.047744152
LSM8	U6 snRNA-associated Sm-like protein LSm8	0.077	0.059	0.094	0.0000176	0.0000943
LTA4H	Leukotriene A-4 hydrolase	0.054	0.036	0.071	0.002126468	0.007500475
LTB	Lymphotoxin-beta	0.088	0.072	0.105	0.000000123	0.000000939
LTBP2	Latent-transforming growth factor beta-binding protein 2	0.077	0.060	0.094	0.00000686	0.0000392
LTBR	Tumor necrosis factor receptor superfamily member 3	0.091	0.074	0.109	0.000000136	0.00000103
LTO1	Protein LTO1 homolog	0.077	0.059	0.095	0.0000116	0.0000642
LUZP2	Leucine zipper protein 2	0.090	0.073	0.108	0.00000018	0.00000134
LY6D	Lymphocyte antigen 6D	0.067	0.049	0.084	0.000130198	0.000588537
LY75	Lymphocyte antigen 75	0.097	0.080	0.113	6.79E-09	6.43E-08
LYZL2	Lysozyme-like protein 2	0.058	0.041	0.076	0.000980862	0.003699434
MAGEA3	Melanoma-associated antigen 3	0.062	0.044	0.080	0.000457947	0.001859136
MANSC1	MANSC domain-containing protein 1	0.050	0.033	0.068	0.004115377	0.013583194
MAPK13	Mitogen-activated protein kinase 13	0.041	0.024	0.059	0.018355922	0.049023832
MAPKAPK2	MAP kinase-activated protein kinase 2	0.060	0.042	0.079	0.000880126	0.003358495
MAPT	Microtubule-associated protein tau	0.067	0.051	0.084	0.000058	0.000282706
MARCO	Macrophage receptor MARCO	0.084	0.067	0.101	0.00000106	0.000007
MARS1	Methionine--tRNA ligase, cytoplasmic	0.054	0.036	0.072	0.00215351	0.007583989
MAVS	Mitochondrial antiviral-signaling protein	0.069	0.051	0.087	0.0000935	0.000438176
MAX	Protein max	0.055	0.037	0.073	0.002091934	0.007393861
MB	Myoglobin	0.106	0.090	0.123	2.13E-10	2.52E-09
MBL2	Mannose-binding protein C	0.133	0.116	0.150	2.31E-15	4.89E-14
MCEE	Methylmalonyl-CoA epimerase, mitochondrial	0.047	0.030	0.065	0.007164976	0.02218562
MENT	Protein MENT	0.063	0.045	0.080	0.00032243	0.001346376
MEP1B	Meprin A subunit beta	0.081	0.064	0.098	0.00000131	0.0000085
MESD	LRP chaperone MESD	0.060	0.043	0.078	0.00057365	0.002278233
MGMT	Methylated-DNA--protein-cysteine methyltransferase	0.174	0.158	0.191	1.64E-26	1.45E-24
MIA	Melanoma-derived growth regulatory protein	0.108	0.092	0.125	4.97E-11	6.37E-10
MICALL2	MICAL-like protein 2	0.150	0.135	0.166	5.27E-22	2.23E-20
MIF	Macrophage migration inhibitory factor	0.045	0.028	0.063	0.010683952	0.031135785
MILR1	Allergin-1	0.097	0.079	0.114	4.36E-08	0.000000358
MINDY1	Ubiquitin carboxyl-terminal hydrolase MINDY-1	0.166	0.151	0.182	8.07E-27	8.14E-25
MLLT1	Protein ENL	-0.060	-0.078	-0.043	0.000546424	0.002177132
MLN	Promotilin	0.096	0.080	0.111	1.24E-09	1.35E-08
MME	Neprilysin	0.063	0.046	0.081	0.000279693	0.001184845
MMP3	Stromelysin-1	0.136	0.120	0.153	2.35E-16	5.68E-15
MNAT1	CDK-activating kinase assembly factor MAT1	0.053	0.035	0.071	0.00354289	0.011944485
MOCS2	Molybdopterin synthase catalytic subunit	-0.061	-0.078	-0.043	0.000638205	0.002497285
MORC3	MORC family CW-type zinc finger protein 3	0.061	0.043	0.079	0.000658426	0.00256953
MPHOSPH8	M-phase phosphoprotein 8	0.116	0.098	0.133	2.53E-11	3.35E-10
MRPL28	39S ribosomal protein L28, mitochondrial	0.066	0.049	0.083	0.000108633	0.00050243
MRPL46	39S ribosomal protein L46, mitochondrial	0.121	0.105	0.137	1.05E-13	1.83E-12
MSLN	Mesothelin	0.098	0.080	0.115	2.92E-08	0.000000251
MSMB	Beta-microseminoprotein	0.085	0.067	0.103	0.00000179	0.0000113
MTIF3	Translation initiation factor IF-3, mitochondrial	0.098	0.080	0.116	5.65E-08	0.000000456
MTSS2	Protein MTSS 2	0.044	0.027	0.062	0.011986694	0.034249371
MUC13	Mucin-13	0.124	0.107	0.141	1.2E-13	2.08E-12
MUC16	Mucin-16	0.099	0.082	0.116	8.46E-09	7.88E-08
MVK	Mevalonate kinase	0.100	0.084	0.117	1.47E-09	1.58E-08
MYBPC2	Myosin-binding protein C, fast-type	0.052	0.034	0.069	0.003486904	0.011769308
MYL1	Myosin light chain 1/3, skeletal muscle isoform	0.098	0.081	0.115	8.66E-09	8.01E-08
MYL4	Myosin light chain 4	0.053	0.035	0.071	0.002984303	0.010202476
MYL6B	Myosin light chain 6B	0.089	0.071	0.106	0.000000581	0.00000401
MYOC	Myocilin	0.043	0.027	0.060	0.008440101	0.025538733
MYOM3	Myomesin-3	0.047	0.029	0.066	0.010002432	0.029295701
MZT1	Mitotic-spindle organizing protein 1	0.045	0.027	0.062	0.009552297	0.02837537
NAA10	N-alpha-acetyltransferase 10	0.222	0.207	0.237	3.24E-48	2.4E-45
NCF2	Neutrophil cytosol factor 2	-0.043	-0.061	-0.025	0.015650912	0.042955508
NEXN	Nexilin	0.050	0.032	0.068	0.004544117	0.014800659
NMI	N-myc-interactor	0.079	0.062	0.095	0.00000238	0.0000147

NOP56	Nucleolar protein 56	0.052	0.034	0.069	0.002841641	0.009794949
NOTCH2	Neurogenic locus notch homolog protein 2	0.106	0.089	0.123	7.29E-10	8.07E-09
NOTCH3	Neurogenic locus notch homolog protein 3	0.075	0.057	0.092	0.0000212	0.000110894
NPM1	Nucleophosmin	0.048	0.030	0.066	0.007045294	0.021861352
NPPB	Natriuretic peptides B	0.147	0.130	0.164	6.55E-18	1.9E-16
NPTN	Neuroplastin	0.085	0.069	0.102	0.000000345	0.00000248
NPTX2	Neuronal pentraxin-2	0.042	0.025	0.059	0.012539339	0.035710079
NPY	Pro-neuropeptide Y	0.042	0.024	0.059	0.018364387	0.049023832
NT5E	5'-nucleotidase	0.049	0.036	0.062	0.000117951	0.000540391
NUBP1	Cytosolic Fe-S cluster assembly factor NUBP1	-0.084	-0.101	-0.068	0.000000493	0.00000344
NUDC	Nuclear migration protein nudC	0.056	0.039	0.074	0.001425361	0.005234083
NXPH3	Neurexophilin-3	0.054	0.036	0.071	0.001907456	0.006799382
OBP2B	Odorant-binding protein 2b	0.111	0.094	0.129	1.19E-10	1.44E-09
ORM1	Alpha-1-acid glycoprotein 1	0.061	0.045	0.076	0.00010024	0.000466562
OSTN	Osteocrin	0.156	0.140	0.172	1.13E-22	5.34E-21
OTOA	Otoancorin	0.053	0.035	0.070	0.00270642	0.009395326
OTUD7B	OTU domain-containing protein 7B	0.120	0.104	0.137	3.64E-13	5.98E-12
OXT	Oxytocin-neurophysin 1	0.124	0.108	0.140	1.95E-15	4.15E-14
PACS2	Phosphofurin acidic cluster sorting protein 2	0.071	0.054	0.089	0.0000342	0.00017319
PADI2	Protein-arginine deiminase type-2	0.051	0.033	0.069	0.004646244	0.015106753
PADI4	Protein-arginine deiminase type-4	0.071	0.053	0.089	0.0000961	0.0004494
PAEP	Glycodelin	0.138	0.121	0.154	1.38E-16	3.43E-15
PALM3	Paralemmin-3	0.051	0.034	0.068	0.00309988	0.010560546
PAM	Peptidyl-glycine alpha-amidating monooxygenase	-0.043	-0.061	-0.026	0.014760596	0.041012568
PAMR1	Inactive serine protease PAMR1	0.084	0.068	0.101	0.000000224	0.00000166
PARK7	Parkinson disease protein 7	0.042	0.024	0.060	0.01861456	0.049554061
PBLD	Phenazine biosynthesis-like domain-containing protein	0.063	0.046	0.081	0.000314499	0.001315136
PCDH17	Protocadherin-17	0.139	0.122	0.157	2.83E-15	5.95E-14
PCDH7	Protocadherin-7	0.085	0.069	0.101	8.01E-08	0.000000625
PCDH9	Protocadherin-9	0.098	0.082	0.115	1.67E-09	1.76E-08
PCSK7	Proprotein convertase subtilisin/kexin type 7	0.042	0.024	0.060	0.018458192	0.049182585
PDCD5	Programmed cell death protein 5	0.049	0.031	0.067	0.006055732	0.019148589
PDGFC	Platelet-derived growth factor C	0.078	0.061	0.096	0.00000434	0.0000258
PDGFRB	Platelet-derived growth factor receptor beta	0.049	0.031	0.067	0.00605968	0.019148589
PDIA2	Protein disulfide-isomerase A2	0.047	0.029	0.065	0.00888717	0.026615982
PDIA3	Protein disulfide-isomerase A3	0.094	0.077	0.112	6.54E-08	0.000000525
PEAR1	Platelet endothelial aggregation receptor 1	0.079	0.063	0.095	0.000000571	0.00000395
PECR	Peroxisomal trans-2-enoyl-CoA reductase	0.053	0.035	0.071	0.002881487	0.009908924
PER3	Period circadian protein homolog 3	0.081	0.065	0.098	0.00000149	0.00000955
PFKFB2	6-phosphofructo-2-kinase/fructose-2,6-bisphosphatase 2	0.099	0.082	0.115	2.49E-09	2.54E-08
PGF	Placenta growth factor	0.053	0.035	0.071	0.003037498	0.010372204
PGLYRP2	N-acetylmuramoyl-L-alanine amidase	0.134	0.117	0.151	3.1E-15	6.47E-14
PI16	Peptidase inhibitor 16	0.049	0.031	0.066	0.004833724	0.015646705
PIGR	Polymeric immunoglobulin receptor	0.115	0.099	0.131	4.91E-13	7.85E-12
PINLYP	phospholipase A2 inhibitor and Ly6/PLAUR domain-containing protein	0.078	0.061	0.094	0.00000361	0.000022
PITHD1	PITH domain-containing protein 1	0.075	0.057	0.092	0.0000195	0.000103465
PLA2G10	Group 10 secretory phospholipase A2	0.047	0.029	0.066	0.008594454	0.025978893
PLA2G1B	Phospholipase A2	0.083	0.066	0.101	0.00000152	0.00000973
PLPBP	Pyridoxal phosphate homeostasis protein	0.055	0.038	0.073	0.001547611	0.005598598
PLSCR3	Phospholipid scramblase 3	0.074	0.058	0.090	0.00000599	0.0000345
PLXDC1	Plexin domain-containing protein 1	0.094	0.077	0.111	2.94E-08	0.000000252
PLXNA4	Plexin-A4	0.071	0.054	0.087	0.0000201	0.000105998
PM20D1	N-fatty-acyl-amino acid synthase/hydrolase PM20D1	0.103	0.085	0.120	1.96E-09	2.05E-08
PMCH	Pro-MCH	0.076	0.059	0.093	0.00000772	0.0000437
PMM2	Phosphomannomutase 2	0.052	0.034	0.070	0.003463846	0.011718544
PMS1	PMS1 protein homolog 1	0.062	0.044	0.079	0.000464973	0.001882431
PMVK	Phosphomevalonate kinase	0.047	0.029	0.065	0.008704423	0.026175956
PNLIP	Pancreatic triacylglycerol lipase	0.044	0.025	0.062	0.016497878	0.044858881
PNMA1	Paraneoplastic antigen Ma1	0.058	0.041	0.076	0.000774318	0.002989869
PNMA2	Paraneoplastic antigen Ma2	0.069	0.052	0.087	0.0000875	0.00041194
PNPT1	Polyribonucleotide nucleotidyltransferase 1, mitochondrial	0.045	0.027	0.064	0.011748089	0.033633364
PODXL	Podocalyxin	0.050	0.033	0.068	0.004783731	0.015502047
PON2	Serum paraoxonase/arylesterase 2	0.079	0.061	0.097	0.00000728	0.0000414
PON3	Serum paraoxonase/lactonase 3	0.068	0.050	0.085	0.000117094	0.0005398
PPIB	Peptidyl-prolyl cis-trans isomerase B	0.066	0.049	0.084	0.000145781	0.000648582
PPIE	Peptidyl-prolyl cis-trans isomerase E	0.147	0.131	0.164	6.45E-20	2.19E-18
PPP1R12B	Protein phosphatase 1 regulatory subunit 12B	-0.071	-0.087	-0.054	0.0000183	0.0000978
PPP1R9B	Neurabin-2	0.104	0.087	0.122	3.32E-09	3.28E-08
PPT1	Palmitoyl-protein thioesterase 1	0.136	0.121	0.151	8.87E-21	3.28E-19
PRC1	Protein regulator of cytokinesis 1	0.085	0.068	0.103	0.000000645	0.00000441
PRG2	Bone marrow proteoglycan	0.084	0.067	0.101	0.000000724	0.00000492
PRKAG3	5'-AMP-activated protein kinase subunit gamma-3	0.162	0.145	0.179	8.78E-22	3.51E-20
PRKD2	Serine/threonine-protein kinase D2	0.060	0.042	0.078	0.000737935	0.002856931
PRKG1	cGMP-dependent protein kinase 1	0.116	0.099	0.133	5.13E-12	7.39E-11
PROC	Vitamin K-dependent protein C	-0.041	-0.058	-0.024	0.017440757	0.047028906
PROCR	Endothelial protein C receptor	0.058	0.040	0.075	0.001180074	0.004399688
PROS1	Vitamin K-dependent protein S	0.145	0.128	0.161	1.8E-18	5.66E-17
PRSS8	Prostasin	0.046	0.028	0.064	0.009909797	0.029082666
PSAPL1	Proactivator polypeptide-like 1	0.057	0.040	0.074	0.000975293	0.003687943
PSIP1	PC4 and SFRS1-interacting protein	0.062	0.045	0.079	0.000252389	0.001080136
PSMD9	26S proteasome non-ATPase regulatory subunit 9	0.047	0.029	0.065	0.009159124	0.027346393
PTEN	Phosphatidylinositol 3,4,5-trisphosphate 3-phosphatase and dual-specificity protein ph	-0.046	-0.064	-0.029	0.008241592	0.024989808
PTGES2	Prostaglandin E synthase 2	-0.029	-0.039	-0.018	0.005041522	0.016229482
PTGR1	Prostaglandin reductase 1	0.055	0.038	0.073	0.001748045	0.006269368
PTPN6	Tyrosine-protein phosphatase non-receptor type 6	0.076	0.059	0.094	0.000011	0.000061
PTPRF	Receptor-type tyrosine-protein phosphatase F	0.063	0.045	0.080	0.00039362	0.00162507
PTPRR	Receptor-type tyrosine-protein phosphatase R	0.052	0.036	0.068	0.001193791	0.004445159
PTPRZ1	Receptor-type tyrosine-protein phosphatase zeta	0.061	0.044	0.079	0.000455123	0.001850244
PTS	6-pyruvoyl tetrahydrobiopterin synthase	0.094	0.079	0.110	5.06E-10	5.73E-09
PTTG1	Securin	0.068	0.050	0.086	0.000165086	0.000730024
PVALB	Parvalbumin alpha	0.046	0.029	0.064	0.009144588	0.027330911
PVR	Poliovirus receptor	0.073	0.055	0.090	0.0000377	0.000188905
PXDNL	Peroxidasin-like protein	0.088	0.071	0.105	0.000000169	0.00000126
QPCT	Glutaminyl-peptide cyclotransferase	0.102	0.086	0.119	5.99E-10	6.69E-09
QSOX1	Sulfhydryl oxidase 1	0.065	0.049	0.082	0.000078	0.000372097
RAB11FIP3	Rab11 family-interacting protein 3	0.057	0.039	0.075	0.001314705	0.004864407
RAB39B	Ras-related protein Rab-39B	0.087	0.071	0.104	0.000000231	0.0000017
RARRES1	Retinoic acid receptor responder protein 1	0.050	0.032	0.068	0.005705047	0.01814565
RASGRF1	Ras-specific guanine nucleotide-releasing factor 1	0.107	0.090	0.124	5.35E-10	6.02E-09
RASSF2	Ras association domain-containing protein 2	0.042	0.027	0.058	0.007407716	0.022768404
RBFOX3	RNA binding protein fox-1 homolog 3	0.079	0.062	0.096	0.00000384	0.0000231
RBKS	Ribokinase	0.044	0.027	0.062	0.009851826	0.028970711
RBP5	Retinol-binding protein 5	0.050	0.032	0.068	0.004517517	0.014770361
REEP4	Receptor expression-enhancing protein 4	0.049	0.031	0.067	0.006483533	0.020334086
REG3G	Regenerating islet-derived protein 3-gamma	0.052	0.034	0.070	0.003480262	0.011760469
REN	Renin	-0.047	-0.063	-0.031	0.003334352	0.011332919
RET	Proto-oncogene tyrosine-protein kinase receptor Ret	0.099	0.082	0.116	7.21E-09	0.000000068

RHOC	Rho-related GTP-binding protein RhoC	0.065	0.047	0.083	0.000263877	0.001126004
RLN1	Prorelaxin H1	0.161	0.145	0.178	5.03E-23	2.45E-21
RNASE1	Ribonuclease pancreatic	0.050	0.032	0.068	0.006093793	0.019214841
RNASEH2A	Ribonuclease H2 subunit A	-0.044	-0.062	-0.026	0.014737549	0.040987493
RNF149	E3 ubiquitin-protein ligase RNF149	0.050	0.033	0.068	0.004438579	0.014544805
RNF4	E3 ubiquitin-protein ligase RNF4	0.051	0.033	0.068	0.004322574	0.014196499
ROBO1	Roundabout homolog 1	0.051	0.034	0.069	0.003181331	0.010825415
ROR1	Inactive tyrosine-protein kinase transmembrane receptor ROR1	-0.053	-0.067	-0.038	0.000430051	0.00176056
RPGR	X-linked retinitis pigmentosa GTPase regulator	0.048	0.030	0.066	0.006353718	0.019969804
RRM2	Ribonucleoside-diphosphate reductase subunit M2	0.054	0.036	0.072	0.002838164	0.009794949
RTBDN	Retbindin	0.045	0.027	0.063	0.01147923	0.03305792
S100A13	Protein S100-A13	0.080	0.063	0.097	0.00000359	0.0000219
SAG	S-arrestin	0.052	0.034	0.070	0.003812847	0.01270804
SAMD9L	Sterile alpha motif domain-containing protein 9-like	0.056	0.038	0.074	0.002068652	0.007320424
SCARA5	Scavenger receptor class A member 5	0.049	0.031	0.067	0.006551152	0.020524135
SCARF1	Scavenger receptor class F member 1	0.052	0.034	0.069	0.003615478	0.01213471
SCGB2A2	Mammaglobin-A	0.099	0.082	0.115	2.14E-09	2.22E-08
SCGB3A2	Secretoglobin family 3A member 2	0.060	0.042	0.077	0.000640114	0.002501407
SCN2A	Sodium channel protein type 2 subunit alpha	0.049	0.031	0.067	0.005925126	0.018784319
SCN3A	Sodium channel protein type 3 subunit alpha	0.045	0.027	0.063	0.011631405	0.033430282
SCN3B	Sodium channel subunit beta-3	0.044	0.026	0.062	0.015117153	0.041868479
SCN4B	Sodium channel subunit beta-4	0.059	0.040	0.077	0.001255506	0.004657163
SCPEP1	Retinoid-inducible serine carboxypeptidase	0.165	0.150	0.181	2.54E-25	1.65E-23
SCRN1	Secernin-1	0.091	0.076	0.107	2.95E-09	2.95E-08
SEC31A	Protein transport protein Sec31A	0.124	0.108	0.140	9.78E-15	1.93E-13
SELL	L-selectin	0.043	0.025	0.060	0.01594141	0.043507695
SEMA4C	Semaphorin-4C	0.125	0.109	0.141	1.72E-14	3.31E-13
SEPTIN3	Neuronal-specific septin-3	0.049	0.030	0.067	0.007374444	0.02271391
SERPINA1	Alpha-1-antitrypsin	0.139	0.122	0.155	2.45E-16	5.86E-15
SERPINA6	Corticosteroid-binding globulin	0.067	0.049	0.085	0.000189613	0.00082476
SERPINB9	Serpin B9	0.053	0.037	0.069	0.001149781	0.004292224
SERPINC1	Antithrombin-III	0.168	0.152	0.184	1.4E-26	1.32E-24
SERPINE2	Glia-derived nexin	0.048	0.030	0.065	0.006074047	0.019173262
SERPINH1	Serpin H1	0.069	0.053	0.085	0.0000201	0.000106055
SH2B3	SH2B adapter protein 3	0.049	0.031	0.067	0.006318886	0.019881706
SHC1	SHC-transforming protein 1	0.117	0.099	0.134	1.23E-11	1.73E-10
SHH	Sonic hedgehog protein	0.092	0.075	0.110	0.000000113	0.000000864
SIGLEC15	Sialic acid-binding Ig-like lectin 15	0.056	0.039	0.074	0.001439832	0.005279059
SIL1	Nucleotide exchange factor SIL1	0.048	0.030	0.065	0.00574097	0.018240061
SKAP2	Src kinase-associated phosphoprotein 2	0.056	0.040	0.073	0.000522812	0.002093395
SLC4A1	Band 3 anion transport protein	0.072	0.054	0.090	0.0000533	0.000262212
SLC9A3R2	Na(+)/H(+) exchange regulatory cofactor NHE-RF2	-0.045	-0.062	-0.028	0.008050896	0.024487793
SLIRP	SRA stem-loop-interacting RNA-binding protein, mitochondrial	0.055	0.037	0.072	0.001780753	0.006363255
SLIT2	Slit homolog 2 protein	0.057	0.039	0.075	0.001361915	0.005020024
SMNDC1	Survival of motor neuron-related-splicing factor 30	0.151	0.136	0.165	4.03E-26	3.1E-24
SMPD1	Sphingomyelin phosphodiesterase	0.099	0.082	0.116	8.55E-09	7.93E-08
SMPD3	Sphingomyelin phosphodiesterase 3	0.056	0.038	0.073	0.001337416	0.004942184
SNAPIN	SNARE-associated protein Snapin	0.046	0.028	0.063	0.009591675	0.028434551
SNED1	Sushi, nidogen and EGF-like domain-containing protein 1	0.044	0.026	0.062	0.015069169	0.041790494
SNRPB2	U2 small nuclear ribonucleoprotein B''	0.042	0.025	0.059	0.015125937	0.041868479
SNX15	Sorting nexin-15	0.113	0.096	0.130	2.15E-11	2.87E-10
SORCS2	VPS10 domain-containing receptor SorCS2	0.045	0.029	0.062	0.005985203	0.018954224
SORD	Sorbitol dehydrogenase	0.044	0.027	0.060	0.008650363	0.026093923
SORT1	Sortilin	0.047	0.030	0.065	0.00682764	0.02129903
SOX2	Transcription factor SOX-2	0.068	0.051	0.085	0.0000854	0.000403455
SPACA5_SPACA5B	Sperm acrosome-associated protein 5	0.078	0.060	0.095	0.0000111	0.0000613
SPAG1	Sperm-associated antigen 1	0.042	0.025	0.060	0.015894665	0.043461277
SPINK2	Serine protease inhibitor Kazal-type 2	0.083	0.066	0.100	0.000000947	0.00000632
SPINK4	Serine protease inhibitor Kazal-type 4	0.070	0.057	0.083	9.27E-08	0.000000713
SPINK5	Serine protease inhibitor Kazal-type 5	0.086	0.068	0.104	0.00000156	0.00000996
SPRR3	Small proline-rich protein 3	0.068	0.051	0.086	0.000117452	0.0005398
SRPK2	SRSF protein kinase 2	0.090	0.074	0.106	1.46E-08	0.000000131
SRPX	Sushi repeat-containing protein SRPX	0.052	0.034	0.069	0.003580586	0.012057663
SSC5D	Soluble scavenger receptor cysteine-rich domain-containing protein SSC5D	0.063	0.046	0.080	0.000266782	0.001136739
ST13	Hsc70-interacting protein	0.055	0.037	0.073	0.001964488	0.006968688
STIP1	Stress-induced-phosphoprotein 1	0.071	0.054	0.087	0.0000147	0.0000797
STX16	Syntaxin-16	0.136	0.120	0.152	3.13E-17	8.31E-16
STX1B	Syntaxin-1B	0.052	0.034	0.070	0.003598014	0.012102411
STX3	Syntaxin-3	0.121	0.105	0.138	1.49E-13	2.56E-12
STX4	Syntaxin-4	0.173	0.156	0.189	3.22E-26	2.62E-24
SYAP1	Synapse-associated protein 1	0.044	0.026	0.062	0.012557669	0.035710079
SYTL4	Synaptotagmin-like protein 4	0.053	0.035	0.071	0.002855194	0.009830072
TACSTD2	Tumor-associated calcium signal transducer 2	0.044	0.026	0.062	0.015345098	0.042354789
TAFA5	Chemokine-like protein TAFA-5	0.050	0.032	0.068	0.005619479	0.017951626
TAP1	Antigen peptide transporter 1	0.080	0.062	0.097	0.00000639	0.0000366
TBC1D23	TBC1 domain family member 23	-0.055	-0.073	-0.036	0.002615157	0.009100123
TBCA	Tubulin-specific chaperone A	0.043	0.026	0.061	0.012565535	0.035710079
TBCB	Tubulin-folding cofactor B	0.075	0.058	0.093	0.0000151	0.0000813
TBCC	Tubulin-specific chaperone C	0.085	0.069	0.100	4.12E-08	0.000000343
TCOF1	Treacle protein	0.097	0.080	0.115	3.16E-08	0.000000268
TCP11	T-complex protein 11 homolog	-0.071	-0.089	-0.054	0.0000533	0.000262212
TDGF1	Teratocarcinoma-derived growth factor 1	0.060	0.043	0.078	0.000696358	0.002706723
TDP1	Tyrosyl-DNA phosphodiesterase 1	0.097	0.079	0.114	3.91E-08	0.000000328
TEF	Thyrotroph embryonic factor	0.114	0.097	0.131	1.98E-11	2.66E-10
TEK	Angiopoietin-1 receptor	0.045	0.027	0.063	0.012861562	0.036463963
TERF1	Telomeric repeat-binding factor 1	0.134	0.117	0.152	1.83E-14	3.48E-13
TET2	Methylcytosine dioxygenase TET2	-0.047	-0.064	-0.029	0.00736627	0.022712667
TFAP2A	Transcription factor AP-2-alpha	-0.080	-0.098	-0.063	0.00000394	0.0000236
TFF1	Trefoil factor 1	0.073	0.057	0.089	0.00000778	0.000044
TFF3	Trefoil factor 3	0.088	0.073	0.103	9.38E-09	8.62E-08
TGFB2	Transforming growth factor beta-2 proprotein	0.112	0.095	0.129	6.83E-11	8.49E-10
TGFBI	Transforming growth factor-beta-induced protein ig-h3	0.076	0.059	0.094	0.0000124	0.0000676
TGFBR2	TGF-beta receptor type-2	0.043	0.025	0.061	0.015384304	0.042413907
TGOLN2	Trans-Golgi network integral membrane protein 2	-0.068	-0.082	-0.053	0.00000566	0.0000329
THBS4	Thrombospondin-4	0.150	0.134	0.166	8.97E-21	3.28E-19
THPO	Thrombopoietin	0.101	0.084	0.118	2.7E-09	2.73E-08
THRAP3	Thyroid hormone receptor-associated protein 3	0.062	0.044	0.080	0.000622243	0.002447935
TIE1	Tyrosine-protein kinase receptor Tie-1	0.051	0.033	0.069	0.004490334	0.014697924
TIMM8A	Mitochondrial import inner membrane translocase subunit Tim8 A	-0.062	-0.079	-0.044	0.000468722	0.001894983
TIMP3	Metalloproteinase inhibitor 3	0.049	0.032	0.067	0.005210217	0.016698975
TK1	Thymidine kinase, cytosolic	0.067	0.049	0.084	0.000145085	0.000646468
TLR4	Toll-like receptor 4	0.044	0.027	0.061	0.01091406	0.03164861
TMEM25	Transmembrane protein 25	0.048	0.030	0.065	0.007900373	0.024130398
TMOD4	Tropomodulin-4	0.045	0.027	0.062	0.010813264	0.031444529
TNC	Tenascin	0.050	0.033	0.068	0.003971108	0.013175423
TNF	Tumor necrosis factor	0.065	0.048	0.082	0.000174264	0.000762533
TNFRSF10B	Tumor necrosis factor receptor superfamily member 10B	0.044	0.026	0.061	0.014422859	0.040265535

TNFRSF11B	Tumor necrosis factor receptor superfamily member 11B	0.088	0.072	0.104	2.71E-08	0.000000234
TNFSF11	Tumor necrosis factor ligand superfamily member 11	0.087	0.071	0.104	0.000000127	0.000000965
TOP1	DNA topoisomerase 1	0.067	0.050	0.084	0.000081	0.000384326
TP53	Cellular tumor antigen p53	0.050	0.033	0.068	0.004332606	0.014213477
TPM3	Tropomyosin alpha-3 chain	0.048	0.031	0.066	0.004985375	0.01606643
TPSAB1	Tryptase alpha/beta-1	0.073	0.055	0.091	0.000046	0.000229033
TPSD1	Tryptase delta	0.065	0.048	0.083	0.000170217	0.000749313
TPT1	Translationally-controlled tumor protein	0.065	0.049	0.082	0.0000868	0.000409336
TRIM24	Transcription intermediary factor 1-alpha	0.048	0.030	0.066	0.007253753	0.022413023
TSLP	Thymic stromal lymphopoietin	0.065	0.047	0.083	0.000235712	0.001011726
TSPAN1	Tetraspanin-1	0.055	0.037	0.073	0.002160543	0.007590464
TSPAN15	Tetraspanin-15	0.046	0.028	0.064	0.00977458	0.028830572
TWF2	Twinfilin-2	-0.043	-0.061	-0.025	0.01672824	0.045400784
TXNDC15	Thioredoxin domain-containing protein 15	0.054	0.037	0.072	0.001762242	0.006312543
TXNL1	Thioredoxin-like protein 1	0.096	0.079	0.114	4.18E-08	0.000000345
TXNRD1	Thioredoxin reductase 1, cytoplasmic	0.068	0.050	0.085	0.000113347	0.000523404
TYRO3	Tyrosine-protein kinase receptor TYRO3	0.049	0.031	0.066	0.005150089	0.016542538
UBAC1	Ubiquitin-associated domain-containing protein 1	0.047	0.029	0.064	0.007309049	0.022560032
UBE2L6	Ubiquitin/ISG15-conjugating enzyme E2 L6	0.046	0.027	0.064	0.012338241	0.035185052
UNG	Uracil-DNA glycosylase	0.051	0.033	0.069	0.004547038	0.014800659
USP28	Ubiquitin carboxyl-terminal hydrolase 28	0.042	0.024	0.060	0.018756966	0.049887727
USP47	Ubiquitin carboxyl-terminal hydrolase 47	0.044	0.027	0.062	0.012756469	0.036201125
VEGFC	Vascular endothelial growth factor C	-0.116	-0.132	-0.100	4.71E-13	7.61E-12
VPS28	Vacuolar protein sorting-associated protein 28 homolog	0.046	0.028	0.064	0.008821146	0.026472496
VSTM1	V-set and transmembrane domain-containing protein 1	0.067	0.051	0.082	0.0000251	0.000129456
WAS	Wiskott-Aldrich syndrome protein	0.124	0.107	0.141	5.28E-13	8.39E-12
WASHC3	WASH complex subunit 3	0.093	0.077	0.109	4.5E-09	4.36E-08
WASL	Neural Wiskott-Aldrich syndrome protein	0.093	0.076	0.110	4.31E-08	0.000000355
WFIKKN1	WAP, Kazal, immunoglobulin, Kunitz and NTR domain-containing protein 1	0.058	0.041	0.074	0.000584787	0.002313509
WFIKKN2	WAP, Kazal, immunoglobulin, Kunitz and NTR domain-containing protein 2	0.078	0.062	0.094	0.00000076	0.00000513
XPNPEP2	Xaa-Pro aminopeptidase 2	0.042	0.024	0.059	0.017820346	0.047744152
YARS1	Tyrosine--tRNA ligase, cytoplasmic	0.126	0.111	0.141	1.9E-16	4.67E-15

Models were adjusted for age, sex, ethnicity, Townsend deprivation index, smoking status, alcohol intake, physical activity, body mass index, household income, education status, hypertension, high density lipoprotein, triglyceride, glycosylated hemoglobin, glucose-lowering drugs, lipid-lowering drugs, coronary heart disease at baseline and stroke at baseline.

Supplementary Table 14 Associations of plasma proteins with the risk of HF

	Protein's description used in the UK Biobank	Association of plasma proteins with the risk of HF				
		HR	Lower	Upper	*P*	*FDR*
ABL1	Tyrosine-protein kinase ABL1	1.194	1.127	1.265	2.22E-09	1.98E-08
ACAA1	3-ketoacyl-CoA thiolase, peroxisomal	1.094	1.027	1.165	5.35E-03	1.87E-02
ACADM	Medium-chain specific acyl-CoA dehydrogenase, mitochondrial	1.310	1.250	1.374	4.40E-29	2.30E-27
ACADSB	Short/branched chain specific acyl-CoA dehydrogenase, mitochondrial	1.080	1.022	1.140	6.03E-03	0.020817925
ACAN	Aggrecan core protein	1.453	1.380	1.530	1.90E-45	5.04E-43
ACHE	Acetylcholinesterase	1.235	1.165	1.309	1.11E-12	1.33E-11
ACOX1	Peroxisomal acyl-coenzyme A oxidase 1	1.090	1.027	1.157	0.004518703	1.61E-02
ACP1	Low molecular weight phosphotyrosine protein phosphatase	1.101	1.034	1.172	2.65E-03	9.95E-03
ACP5	Tartrate-resistant acid phosphatase type 5	1.194	1.129	1.263	5.18E-10	4.90E-09
ACP6	Lysophosphatidic acid phosphatase type 6	0.927	0.876	0.982	9.78E-03	0.032033281
ACRBP	Acrosin-binding protein	1.236	1.151	1.327	4.85E-09	4.13E-08
ACRV1	Acrosomal protein SP-10	1.062	1.013	1.114	0.013220452	4.17E-02
ACTN2	Alpha-actinin-2	1.107	1.044	1.174	0.000697428	2.96E-03
ACTN4	Alpha-actinin-4	1.124	1.057	1.195	1.89E-04	8.93E-04
ACVRL1	Serine/threonine-protein kinase receptor R3	1.097	1.035	1.163	1.86E-03	7.22E-03
ACY1	Aminoacylase-1	1.081	1.032	1.132	9.74E-04	0.004019413
ACY3	N-acyl-aromatic-L-amino acid amidohydrolase	0.899	0.841	0.962	2.14E-03	0.008184361
ADA	Adenosine deaminase	1.757	1.629	1.895	2.47E-48	8.03E-46
ADAM12	Disintegrin and metalloproteinase domain-containing protein 12	1.124	1.059	1.194	0.000133804	6.45E-04
ADAM15	Disintegrin and metalloproteinase domain-containing protein 15	1.162	1.092	1.236	2.03E-06	1.28E-05
ADAM23	Disintegrin and metalloproteinase domain-containing protein 23	1.289	1.225	1.356	2.52E-22	7.84E-21
ADAM8	Disintegrin and metalloproteinase domain-containing protein 8	1.177	1.099	1.261	3.45E-06	0.0000211
ADAMTS1	A disintegrin and metalloproteinase with thrombospondin motifs 1	1.079	1.016	1.147	1.31E-02	0.041439095
ADCYAP1R1	Pituitary adenylate cyclase-activating polypeptide type I receptor	1.136	1.066	1.210	0.0000786	3.93E-04
ADGRD1	Adhesion G-protein coupled receptor D1	1.104	1.030	1.183	0.005020373	1.78E-02
ADGRE2	Adhesion G protein-coupled receptor E2	1.376	1.295	1.461	4.01E-25	1.56E-23
ADGRE5	Adhesion G protein-coupled receptor E5	1.103	1.041	1.170	9.58E-04	3.97E-03
ADGRG2	Adhesion G-protein coupled receptor G2	0.921	0.866	0.979	8.37E-03	0.027936401
ADGRV1	Adhesion G-protein coupled receptor V1	0.917	0.861	0.976	6.45E-03	0.022103192
ADH1B	All-trans-retinol dehydrogenase	1.178	1.109	1.251	0.000000117	8.79E-07
ADIPOQ	Adiponectin	1.520	1.434	1.611	4.81E-45	1.08E-42
ADM	Pro-adrenomedullin	1.198	1.127	1.274	7.79E-09	6.43E-08
ADRA2A	Alpha-2A adrenergic receptor	1.149	1.079	1.223	1.47E-05	8.31E-05
AFAP1	Actin filament-associated protein 1	1.214	1.139	1.295	3.16E-09	2.77E-08
AFM	Afamin	1.405	1.320	1.495	8.87E-27	3.76E-25
AGR2	Anterior gradient protein 2 homolog	1.078	1.019	1.142	0.009559012	3.15E-02
AGT	Angiotensinogen	1.145	1.081	1.213	0.00000372	2.26E-05
AHCY	Adenosylhomocysteinase	1.196	1.124	1.273	1.90E-08	1.51E-07
AHNAK	Neuroblast differentiation-associated protein AHNAK	1.082	1.017	1.152	1.24E-02	3.95E-02
AHSA1	Activator of 90 kDa heat shock protein ATPase homolog 1	0.868	0.815	0.925	1.31E-05	0.0000749
AIF1L	Allograft inflammatory factor 1-like	1.269	1.198	1.345	9.48E-16	1.55E-14
AIFM1	Apoptosis-inducing factor 1, mitochondrial	0.898	0.845	0.954	0.000463953	2.05E-03
ALCAM	CD166 antigen	1.338	1.268	1.412	2.26E-26	9.42E-25
ALPI	Intestinal-type alkaline phosphatase	1.078	1.018	1.141	1.06E-02	3.45E-02
AMFR	E3 ubiquitin-protein ligase AMFR	1.096	1.033	1.163	2.37E-03	9.00E-03
AMIGO2	Amphoterin-induced protein 2	1.320	1.248	1.397	4.77E-22	1.41E-20
AMN	Protein amnionless	0.914	0.857	0.973	5.20E-03	0.018355022
AMOTL2	Angiomotin-like protein 2	1.175	1.103	1.251	0.000000498	3.42E-06
AMY1A AMY1B AMY	Alpha-amylase 1A Alpha-amylase 1B Alpha-amylase 1C	1.110	1.044	1.180	0.000897065	3.74E-03
AMY2A	Pancreatic alpha-amylase	1.206	1.145	1.271	2.28E-12	2.61E-11
ANGPT1	Angiopoietin-1	1.176	1.111	1.245	2.27E-08	1.78E-07
ANGPT2	Angiopoietin-2	0.811	0.762	0.863	3.75E-11	3.87E-10
ANGPTL1	Angiopoietin-related protein 1	1.153	1.078	1.234	3.88E-05	0.000204869
ANKMY2	Ankyrin repeat and MYND domain-containing protein 2	1.088	1.025	1.155	0.005495209	1.92E-02
ANP32C	Acidic leucine-rich nuclear phosphoprotein 32 family member C	1.124	1.057	1.196	0.000213521	9.99E-04
ANXA10	Annexin A10	1.156	1.091	1.225	9.15E-07	6.05E-06
ANXA11	Annexin A11	1.242	1.173	1.315	9.39E-14	1.25E-12
ANXA2	Annexin A2	1.091	1.019	1.169	1.29E-02	0.040834237
ANXA3	Annexin A3	1.245	1.183	1.311	9.15E-17	1.65E-15
ANXA5	Annexin A5	1.248	1.183	1.317	3.56E-16	6.09E-15
AOC1	Amiloride-sensitive amine oxidase [copper-containing]	1.285	1.221	1.353	6.85E-22	2.00E-20
AP1G2	AP-1 complex subunit gamma-like 2	1.174	1.104	1.248	3.27E-07	2.35E-06
AP2B1	AP-2 complex subunit beta	1.141	1.079	1.207	3.49E-06	2.13E-05
APLP1	Amyloid-like protein 1	1.158	1.094	1.226	4.91E-07	0.00000338
APOC1	Apolipoprotein C-I	1.117	1.050	1.188	4.62E-04	0.002040144
APOD	Apolipoprotein D	0.876	0.819	0.936	0.0000983	4.85E-04
APOE	Apolipoprotein E	1.074	1.016	1.136	0.011741345	3.78E-02
APOF	Apolipoprotein F	1.094	1.029	1.162	4.13E-03	1.48E-02
APOH	Beta-2-glycoprotein 1	1.142	1.093	1.192	1.86E-09	1.68E-08
APOL1	Apolipoprotein L1	1.260	1.181	1.345	2.93E-12	3.31E-11
APPL2	DCC-interacting protein 13-beta	1.170	1.102	1.243	2.92E-07	0.00000211
APRT	Adenine phosphoribosyltransferase	1.199	1.129	1.273	3.71E-09	3.25E-08
ARHGAP25	Rho GTPase-activating protein 25	0.903	0.851	0.959	0.000788137	3.31E-03
ARHGAP45	Rho GTPase-activating protein 45	1.261	1.188	1.337	1.44E-14	2.12E-13
ARHGEF10	Rho guanine nucleotide exchange factor 10	1.259	1.201	1.319	3.48E-22	1.06E-20
ARHGEF12	Rho guanine nucleotide exchange factor 12	1.240	1.172	1.311	5.68E-14	7.69E-13
ARHGEF5	Rho guanine nucleotide exchange factor 5	1.276	1.189	1.370	1.42E-11	1.53E-10
ARID3A	AT-rich interactive domain-containing protein 3A	1.116	1.051	1.186	0.000345545	1.57E-03
ARID4B	AT-rich interactive domain-containing protein 4B	1.188	1.128	1.252	1.12E-10	1.11E-09
ARL13B	ADP-ribosylation factor-like protein 13B	1.115	1.060	1.173	2.88E-05	1.55E-04
ARMCX2	Armadillo repeat-containing X-linked protein 2	1.166	1.107	1.229	6.84E-09	5.66E-08
ARNT	Aryl hydrocarbon receptor nuclear translocator	1.214	1.162	1.269	8.24E-18	1.62E-16
ARNTL	Aryl hydrocarbon receptor nuclear translocator-like protein 1	1.104	1.040	1.173	1.18E-03	0.004769935
ARSA	Arylsulfatase A	1.218	1.144	1.296	5.3E-10	5.00E-09
ARSB	Arylsulfatase B	1.098	1.033	1.167	0.002588015	9.74E-03
ART3	Ecto-ADP-ribosyltransferase 3	1.139	1.070	1.213	4.63E-05	2.41E-04
ARTN	Artemin	1.099	1.029	1.175	5.29E-03	1.86E-02
ASAH2	Neutral ceramidase	1.144	1.092	1.199	1.98E-08	0.000000156
ASGR1	Asialoglycoprotein receptor 1	1.096	1.040	1.155	6.08E-04	0.002619946
ASPN	Asporin	1.201	1.138	1.267	2.93E-11	3.04E-10
ASPSCR1	Tether containing UBX domain for GLUT4	1.120	1.053	1.191	0.000341998	1.56E-03
ATF2	Cyclic AMP-dependent transcription factor ATF-2	1.349	1.278	1.423	1.19E-27	5.36E-26
ATF4	Cyclic AMP-dependent transcription factor ATF-4	1.194	1.125	1.267	4.55E-09	3.91E-08
ATG16L1	Autophagy-related protein 16-1	1.247	1.167	1.332	6.13E-11	6.22E-10
ATP1B2	Sodium/potassium-transporting ATPase subunit beta-2	1.191	1.126	1.260	9.94E-10	9.02E-09
ATP1B3	Sodium/potassium-transporting ATPase subunit beta-3	1.237	1.170	1.308	6.16E-14	8.26E-13
ATP1B4	Protein ATP1B4	1.136	1.069	1.208	0.0000445	2.32E-04
ATP6V1D	V-type proton ATPase subunit D	1.137	1.071	1.209	3.12E-05	1.66E-04
ATP6V1F	V-type proton ATPase subunit F	1.087	1.023	1.155	6.87E-03	2.34E-02
ATRAID	All-trans retinoic acid-induced differentiation factor	1.354	1.286	1.426	8.43E-31	5.36E-29
ATRN	Attractin, Isoform 2	1.269	1.205	1.335	7.68E-20	1.87E-18
ATXN10	Ataxin-10	1.259	1.184	1.339	1.71E-13	2.20E-12
ATXN2	Ataxin-2	1.107	1.047	1.170	0.000344125	1.56E-03
ATXN3	Ataxin-3	1.320	1.246	1.399	4.14E-21	1.13E-19
AXIN1	Axin-1	1.447	1.367	1.531	1.33E-37	1.30E-35
AXL	Tyrosine-protein kinase receptor UFO	1.252	1.169	1.340	9.75E-11	9.79E-10
AZI2	5-azacytidine-induced protein 2	1.228	1.157	1.304	1.77E-11	1.9E-10
AZU1	Azurocidin	1.370	1.301	1.443	5.25E-33	3.74E-31
B3GNT7	UDP-GlcNAc:betaGal beta-1,3-N-acetylglucosaminyltransferase 7	1.303	1.214	1.397	1.46E-13	1.90E-12
B4GALT1	Beta-1,4-galactosyltransferase 1	1.197	1.148	1.248	2.65E-17	5.04E-16
B4GAT1	Beta-1,4-glucuronyltransferase 1	1.120	1.059	1.184	6.96E-05	3.52E-04
BACH1	Transcription regulator protein BACH1	1.138	1.077	1.203	4.59E-06	0.0000276
BAG3	BAG family molecular chaperone regulator 3	1.145	1.079	1.215	7.04E-06	0.0000413
BAG4	BAG family molecular chaperone regulator 4	1.092	1.031	1.157	0.002660347	9.97E-03

BAG6	Large proline-rich protein BAG6	1.125	1.067	1.187	0.0000158	8.84E-05
BAMBI	BMP and activin membrane-bound inhibitor homolog	1.326	1.267	1.389	1.43E-33	1.05E-31
BAX	Apoptosis regulator BAX	1.134	1.068	1.204	4.15E-05	2.18E-04
BCAM	Basal cell adhesion molecule	1.447	1.366	1.533	5.18E-36	4.58E-34
BCAN	Brevican core protein	1.331	1.265	1.400	1.86E-28	9.07E-27
BCAT1	Branched-chain-amino-acid aminotransferase, cytosolic	1.298	1.227	1.373	1.53E-19	3.61E-18
BCL2	Apoptosis regulator Bcl-2	1.255	1.189	1.324	1.15E-16	2.05E-15
BCL2L1	Bcl-2-like protein 1	1.268	1.189	1.353	6.63E-13	7.98E-12
BCL2L11	Bcl-2-like protein 11, Isoform BimL	1.260	1.186	1.339	6.57E-14	8.77E-13
BCL7B	B-cell CLL/lymphoma 7 protein family member B	1.265	1.191	1.343	1.40E-14	2.08E-13
BCR	Breakpoint cluster region protein	1.090	1.030	1.153	2.61E-03	0.009814267
BDNF	Brain-derived neurotrophic factor	1.083	1.023	1.146	0.005738191	2.00E-02
BEX3	Protein BEX3	1.269	1.187	1.357	3.08E-12	3.46E-11
BGLAP	Osteocalcin	1.160	1.099	1.225	7.33E-08	5.58E-07
BGN	Biglycan	1.142	1.077	1.210	8.69E-06	5.05E-05
BHLHE40	Class E basic helix-loop-helix protein 40	1.166	1.099	1.237	3.73E-07	0.00000265
BID	BH3-interacting domain death agonist	1.133	1.042	1.233	3.60E-03	0.013072678
BIN2	Bridging integrator 2	0.904	0.849	0.962	0.001515517	5.96E-03
BLNK	B-cell linker protein	1.146	1.087	1.208	0.000000453	3.17E-06
BLOC1S3	Biogenesis of lysosome-related organelles complex 1 subunit 3	1.136	1.072	1.204	1.75E-05	9.77E-05
BLVRB	Flavin reductase	1.141	1.066	1.221	1.34E-04	6.45E-04
BMPER	BMP-binding endothelial regulator protein	1.190	1.122	1.263	9.09E-09	7.46E-08
BNIP2	BCL2/adenovirus E1B 19 kDa protein-interacting protein 2	1.107	1.044	1.173	6.92E-04	0.002939976
BRDT	Bromodomain testis-specific protein	1.247	1.125	1.382	0.0000267	1.45E-04
BRK1	Protein BRICK1	1.164	1.097	1.234	0.000000385	2.73E-06
BSG	Basigin	1.154	1.088	1.224	1.92E-06	1.23E-05
BSND	Barttin	1.278	1.208	1.351	7.62E-18	1.51E-16
BST1	ADP-ribosyl cyclase/cyclic ADP-ribose hydrolase 2	1.123	1.049	1.202	8.15E-04	0.003417598
BTD	Biotinidase	1.368	1.284	1.456	1.77E-22	5.68E-21
BTN2A1	Butyrophilin subfamily 2 member A1	1.318	1.266	1.371	5.34E-42	9.17E-40
BTNL10	Butyrophilin-like protein 10	1.119	1.055	1.186	0.000163604	7.81E-04
C19orf12	Protein C19orf12	1.160	1.091	1.232	1.94E-06	1.24E-05
C1GALT1C1	C1GALT1-specific chaperone 1	1.121	1.056	1.190	1.96E-04	9.22E-04
C1QA	Complement C1q subcomponent subunit A	1.369	1.296	1.446	2.14E-29	1.2E-27
C1QBP	Complement component 1 Q subcomponent-binding protein, mitochondrial	1.274	1.205	1.348	2.09E-17	4.02E-16
C1QTNF6	Complement C1q tumor necrosis factor-related protein 6	1.266	1.191	1.344	2.07E-14	2.98E-13
C1QTNF9	Complement C1q and tumor necrosis factor-related protein 9A	1.124	1.056	1.196	0.000245671	1.14E-03
C1R	Complement C1r subcomponent	1.269	1.198	1.345	7.66E-16	1.26E-14
C1RL	Complement C1r subcomponent-like protein	1.212	1.141	1.286	2.87E-10	2.78E-09
C1S	Complement C1s subcomponent	1.157	1.078	1.241	5.04E-05	0.000259155
C2CD2L	Phospholipid transfer protein C2CD2L	1.339	1.260	1.422	3.23E-21	8.99E-20
C7	Complement component C7	1.257	1.197	1.321	9.78E-20	2.36E-18
CA12	Carbonic anhydrase 12	0.894	0.840	0.952	0.000460694	2.04E-03
CA14	Carbonic anhydrase 14	0.860	0.800	0.924	4.23E-05	2.22E-04
CA3	Carbonic anhydrase 3	0.889	0.833	0.949	4.33E-04	1.93E-03
CA4	Carbonic anhydrase 4	1.110	1.046	1.178	6.09E-04	0.002619946
CA5A	Carbonic anhydrase 5A, mitochondrial	1.511	1.422	1.607	4.01E-40	5.59E-38
CA7	Carbonic anhydrase 7	1.267	1.201	1.336	3.52E-18	7.19E-17
CA8	Carbonic anhydrase-related protein	1.465	1.389	1.545	3.07E-45	7.47E-43
CA9	Carbonic anhydrase 9	1.137	1.075	1.202	6.93E-06	4.08E-05
CABP2	Calcium-binding protein 2	1.435	1.355	1.520	9.95E-35	8.08E-33
CALCB	Calcitonin gene-related peptide 2	1.166	1.086	1.251	2.12E-05	0.000117036
CAPG	Macrophage-capping protein	1.150	1.085	1.218	2.14E-06	0.0000134
CAPS	Calcyphosin	1.158	1.086	1.236	0.00000886	5.14E-05
CASC3	Protein CASC3	1.379	1.305	1.456	2.12E-30	1.26E-28
CASP1	Caspase-1	1.172	1.105	1.243	1.37E-07	1.02E-06
CASP10	Caspase-10	0.915	0.855	0.980	1.07E-02	3.48E-02
CASP3	Caspase-3	1.155	1.082	1.233	1.52E-05	0.0000854
CASP8	Caspase-8	1.159	1.091	1.231	1.72E-06	0.000011
CASQ2	Calsequestrin-2	1.373	1.295	1.456	2.79E-26	1.15E-24
CAT	Catalase	1.085	1.023	1.151	0.006445542	2.21E-02
CBLN1	Cerebellin-1	1.418	1.340	1.500	7.14E-34	5.49E-32
CBLN4	Cerebellin-4	1.162	1.093	1.235	1.35E-06	8.80E-06
CBX2	Chromobox protein homolog 2	1.083	1.026	1.144	3.95E-03	0.014261586
CC2D1A	Coiled-coil and C2 domain-containing protein 1A	1.269	1.195	1.347	8.22E-15	1.25E-13
CCDC50	Coiled-coil domain-containing protein 50	1.178	1.107	1.254	0.000000243	1.77E-06
CCER2	Coiled-coil domain-containing glutamate-rich protein 2	1.096	1.034	1.161	0.002120072	8.10E-03
CCL13	C-C motif chemokine 13	1.081	1.016	1.151	1.45E-02	4.53E-02
CCL14	C-C motif chemokine 14	1.254	1.183	1.330	4.11E-14	5.59E-13
CCL15	C-C motif chemokine 15	1.110	1.044	1.181	9.26E-04	0.003851628
CCL16	C-C motif chemokine 16	1.170	1.091	1.255	1.00E-05	0.0000577
CCL17	C-C motif chemokine 17	0.825	0.772	0.882	1.63E-08	1.31E-07
CCL18	C-C motif chemokine 18	1.169	1.099	1.243	0.000000676	4.57E-06
CCL19	C-C motif chemokine 19	1.242	1.175	1.312	2.13E-14	3.05E-13
CCL2	C-C motif chemokine 2	1.192	1.124	1.264	4.37E-09	3.78E-08
CCL21	C-C motif chemokine 21	1.267	1.199	1.338	2.43E-17	4.64E-16
CCL22	C-C motif chemokine 22	1.186	1.126	1.248	7.82E-11	7.89E-10
CCL23	C-C motif chemokine 23	1.102	1.038	1.170	0.001561289	6.13E-03
CCL24	C-C motif chemokine 24	1.148	1.096	1.203	5.24E-09	4.42E-08
CCL25	C-C motif chemokine 25	1.090	1.027	1.157	4.72E-03	1.69E-02
CCL26	C-C motif chemokine 26	1.241	1.169	1.318	1.92E-12	2.22E-11
CCL27	C-C motif chemokine 27	1.245	1.165	1.331	1.04E-10	1.04E-09
CCL7	C-C motif chemokine 7	1.321	1.252	1.395	5.45E-24	2.02E-22
CCNE1	G1/S-specific cyclin-E1	1.192	1.124	1.265	5.74E-09	4.81E-08
CD109	CD109 antigen	1.197	1.127	1.271	5.19E-09	4.40E-08
CD160	CD160 antigen	1.139	1.072	1.209	2.30E-05	1.26E-04
CD164	Sialomucin core protein 24	1.152	1.087	1.221	1.95E-06	1.24E-05
CD1C	T-cell surface glycoprotein CD1c	1.266	1.209	1.325	1.23E-23	4.34E-22
CD2	T-cell surface antigen CD2	1.116	1.045	1.192	1.02E-03	0.004155542
CD22	B-cell receptor CD22	1.127	1.061	1.198	0.000114563	5.58E-04
CD226	CD226 antigen	1.106	1.044	1.172	0.000682641	2.90E-03
CD27	CD27 antigen	1.353	1.279	1.430	3.34E-26	1.36E-24
CD2AP	CD2-associated protein	0.840	0.788	0.895	6.67E-08	5.11E-07
CD300A	CMRF35-like molecule 8	1.099	1.034	1.168	2.41E-03	0.009160395
CD33	Myeloid cell surface antigen CD33	0.861	0.809	0.918	3.80E-06	0.000023
CD34	Hematopoietic progenitor cell antigen CD34	0.905	0.851	0.963	0.001711342	6.69E-03
CD38	ADP-ribosyl cyclase/cyclic ADP-ribose hydrolase 1	1.333	1.235	1.438	1.25E-13	1.65E-12
CD3D	T-cell surface glycoprotein CD3 delta chain	1.174	1.106	1.246	1.40E-07	1.04E-06
CD3E	T-cell surface glycoprotein CD3 epsilon chain	1.155	1.087	1.226	2.76E-06	1.71E-05
CD4	T-cell surface glycoprotein CD4	1.317	1.242	1.396	2.94E-20	7.28E-19
CD40	Tumor necrosis factor receptor superfamily member 5	1.080	1.016	1.148	1.39E-02	0.043884778
CD46	Membrane cofactor protein	0.899	0.845	0.957	0.000788349	3.31E-03
CD48	CD48 antigen	1.256	1.188	1.328	1.52E-15	2.38E-14
CD59	CD59 glycoprotein	1.204	1.134	1.278	1.07E-09	9.66E-09
CD6	T-cell differentiation antigen CD6	1.640	1.543	1.742	2.43E-57	1.21E-54
CD63	CD63 antigen	0.880	0.827	0.937	6.02E-05	0.00030784
CD7	T-cell antigen CD7	1.412	1.331	1.498	2.01E-30	1.22E-28
CD70	CD70 antigen	1.395	1.327	1.467	6.69E-39	8.14E-37
CD72	B-cell differentiation antigen CD72	1.387	1.325	1.453	7.41E-44	1.55E-41
CD79B	B-cell antigen receptor complex-associated protein beta chain	1.155	1.089	1.226	1.93E-06	1.23E-05
CD80	T-lymphocyte activation antigen CD80	0.820	0.771	0.872	2.10E-10	2.04E-09
CD82	CD82 antigen	1.115	1.059	1.175	4.07E-05	0.000214314
CD83	CD83 antigen	0.926	0.871	0.985	1.43E-02	0.044770403
CD99	CD99 antigen	1.191	1.127	1.259	6.26E-10	5.83E-09
CD99L2	CD99 antigen-like protein 2	1.094	1.030	1.163	0.003604714	1.31E-02
CDC123	Cell division cycle protein 123 homolog	1.229	1.147	1.316	4.59E-09	3.93E-08
CDC25A	M-phase inducer phosphatase 1	0.884	0.836	0.934	1.12E-05	6.40E-05

CDC26	Anaphase-promoting complex subunit CDC26	1.116	1.047	1.190	7.22E-04	0.003048549
CDC42BPB	Serine/threonine-protein kinase MRCK beta	1.088	1.020	1.161	1.03E-02	0.033604525
CDH1	Cadherin-1	1.139	1.062	1.221	0.000255145	1.18E-03
CDH17	Cadherin-17	1.314	1.246	1.385	7.45E-24	2.69E-22
CDH2	Cadherin-2	1.279	1.216	1.345	1.93E-21	5.48E-20
CDH22	Cadherin-22	1.159	1.096	1.226	2.45E-07	1.78E-06
CDH3	Cadherin-3	1.198	1.136	1.264	3.76E-11	3.87E-10
CDH4	Cadherin-4	1.141	1.074	1.212	1.93E-05	0.000107099
CDK5RAP3	CDK5 regulatory subunit-associated protein 3	1.221	1.154	1.293	5.56E-12	6.15E-11
CDKL5	Cyclin-dependent kinase-like 5	1.239	1.165	1.318	1.18E-11	1.28E-10
CDON	Cell adhesion molecule-related/down-regulated by oncogenes	1.117	1.050	1.190	4.98E-04	2.17E-03
CDSN	Corneodesmosin	0.891	0.838	0.947	1.95E-04	9.20E-04
CEACAM1	Carcinoembryonic antigen-related cell adhesion molecule 1	1.117	1.046	1.193	9.46E-04	0.003929644
CEACAM16	Carcinoembryonic antigen-related cell adhesion molecule 16	1.261	1.185	1.342	2.88E-13	3.55E-12
CEACAM18	Carcinoembryonic antigen-related cell adhesion molecule 18	1.169	1.104	1.238	7.71E-08	5.84E-07
CEACAM19	Carcinoembryonic antigen-related cell adhesion molecule 19	1.391	1.298	1.490	5.28E-21	1.39E-19
CEACAM20	Carcinoembryonic antigen-related cell adhesion molecule 20	1.098	1.031	1.169	3.42E-03	1.25E-02
CEACAM21	Carcinoembryonic antigen-related cell adhesion molecule 21	1.092	1.033	1.155	1.93E-03	7.48E-03
CEACAM6	Carcinoembryonic antigen-related cell adhesion molecule 6	1.120	1.050	1.195	5.84E-04	0.002529202
CEACAM8	Carcinoembryonic antigen-related cell adhesion molecule 8	1.318	1.243	1.398	3.28E-20	8.06E-19
CEBPB	CCAAT/enhancer-binding protein beta	1.149	1.097	1.204	4.92E-09	4.18E-08
CELSR2	Cadherin EGF LAG seven-pass G-type receptor 2	1.110	1.046	1.178	0.000617562	2.65E-03
CEMIP2	Cell surface hyaluronidase	1.168	1.109	1.230	4.25E-09	3.70E-08
CEND1	Cell cycle exit and neuronal differentiation protein 1	1.096	1.027	1.169	5.51E-03	1.93E-02
CENPF	Centromere protein F	0.917	0.862	0.977	6.96E-03	0.023620845
CEP164	Centrosomal protein of 164 kDa	1.093	1.033	1.156	1.94E-03	0.007480123
CEP20	Centrosomal protein 20	1.094	1.028	1.164	0.004819027	1.72E-02
CEP350	Centrosome-associated protein 350	1.115	1.057	1.177	0.000071	3.58E-04
CERT	Ceramide transfer protein	1.131	1.055	1.213	5.25E-04	2.28E-03
CES1	Liver carboxylesterase 1	1.094	1.030	1.162	3.51E-03	1.28E-02
CES3	Carboxylesterase 3	1.106	1.038	1.180	1.97E-03	0.007594038
CETN2	Centrin-2	1.342	1.281	1.406	6.06E-35	5.06E-33
CETN3	Centrin-3	1.138	1.069	1.210	0.000044	2.30E-04
CFB	Complement factor B	0.901	0.846	0.960	0.001172978	4.74E-03
CFC1	Cryptic protein	1.194	1.123	1.270	1.77E-08	1.41E-07
CGB3_CGB5_CGB8	Choriogonadotropin subunit beta 3	1.273	1.202	1.348	1.31E-16	2.34E-15
CGN	Cingulin	1.132	1.070	1.197	1.39E-05	0.0000787
CHAC2	Glutathione-specific gamma-glutamylcyclotransferase 2	1.091	1.024	1.162	6.76E-03	0.023098939
CHEK2	Serine/threonine-protein kinase Chk2	1.211	1.145	1.281	2.35E-11	2.47E-10
CHGA	Chromogranin-A	1.220	1.146	1.298	4.07E-10	3.89E-09
CHMP1A	Charged multivesicular body protein 1a	1.216	1.143	1.294	6.59E-10	6.09E-09
CHMP6	Charged multivesicular body protein 6	1.459	1.382	1.539	3.94E-43	7.20E-41
CHRDL1	Chordin-like protein 1	1.151	1.072	1.236	1.04E-04	0.000508854
CHRDL2	Chordin-like protein 2	1.081	1.017	1.149	1.31E-02	0.041336348
CHRM1	Muscarinic acetylcholine receptor M1	1.149	1.080	1.222	0.00000963	5.58E-05
CIT	Citron Rho-interacting kinase	0.921	0.865	0.980	0.009810438	3.21E-02
CKMT1A CKMT1B	Creatine kinase U-type, mitochondrial	1.097	1.034	1.163	2.01E-03	7.70E-03
CLASP1	CLIP-associating protein 1	0.926	0.871	0.983	1.22E-02	3.93E-02
CLC	Galectin-10	1.135	1.067	1.208	6.14E-05	0.000312486
CLEC12A	C-type lectin domain family 12 member A	1.100	1.029	1.175	4.88E-03	0.017338644
CLEC14A	C-type lectin domain family 14 member A	1.143	1.074	1.217	0.0000296	1.59E-04
CLEC1A	C-type lectin domain family 1 member A	1.480	1.405	1.559	4.73E-49	1.73E-46
CLEC2L	C-type lectin domain family 2 member L	1.185	1.117	1.258	1.99E-08	1.57E-07
CLEC4D	C-type lectin domain family 4 member D	1.420	1.339	1.505	6.35E-32	4.32E-30
CLEC4G	C-type lectin domain family 4 member G	1.096	1.031	1.164	3.25E-03	0.011927577
CLEC7A	C-type lectin domain family 7 member A	1.166	1.094	1.244	2.83E-06	0.0000174
CLGN	Calmegin	1.225	1.161	1.294	2.01E-13	2.55E-12
CLIC5	Chloride intracellular channel protein 5	1.100	1.028	1.176	0.005800593	2.01E-02
CLNS1A	Methylosome subunit pICln	1.095	1.029	1.165	4.08E-03	1.47E-02
CLSTN2	Calsyntenin-2	1.087	1.029	1.148	3.04E-03	1.12E-02
CLSTN3	Calsyntenin-3	0.920	0.865	0.977	6.88E-03	0.023422599
CLU	Clusterin	1.087	1.025	1.153	5.65E-03	0.01972663
CLUL1	Clusterin-like protein 1	1.198	1.132	1.268	5.44E-10	5.11E-09
CNPY4	Protein canopy homolog 4	0.900	0.848	0.956	0.000632902	2.71E-03
CNST	Consortin	1.132	1.079	1.187	3.43E-07	2.46E-06
CNTF	Ciliary neurotrophic factor	1.140	1.073	1.213	2.74E-05	1.48E-04
CNTN1	Contactin-1	1.350	1.265	1.440	1.17E-19	2.79E-18
CNTN4	Contactin-4	1.165	1.098	1.236	4.72E-07	0.00000327
CNTN5	Contactin-5	1.128	1.063	1.197	0.0000652	3.30E-04
COCH	Cochlin	0.903	0.844	0.965	0.002700186	1.01E-02
COL18A1	Collagen alpha-1(XVIII) chain	1.270	1.204	1.340	1.42E-18	3.05E-17
COL6A3	Collagen alpha-3(VI) chain	1.430	1.349	1.515	1.28E-33	9.63E-32
COL9A1	Collagen alpha-1(IX) chain	1.320	1.246	1.399	5.05E-21	1.34E-19
COPB2	Coatomer subunit beta'	1.289	1.210	1.373	3.95E-15	6.08E-14
CORO1A	Coronin-1A	1.077	1.016	1.142	0.012442499	3.97E-02
CORO6	Coronin-6	1.324	1.258	1.393	5.25E-27	2.29E-25
CPA1	Carboxypeptidase A1	1.094	1.032	1.160	2.50E-03	9.44E-03
CPA2	Carboxypeptidase A2	1.233	1.160	1.311	1.85E-11	1.98E-10
CPB2	Carboxypeptidase B2	0.924	0.868	0.984	1.41E-02	0.044285684
CPLX2	Complexin-2	1.082	1.021	1.146	7.55E-03	0.025429057
CPTP	Ceramide-1-phosphate transfer protein	1.356	1.277	1.439	1.89E-23	6.51E-22
CPVL	Probable serine carboxypeptidase CPVL	1.351	1.289	1.417	1.47E-35	1.27E-33
CPXM2	Inactive carboxypeptidase-like protein X2	1.076	1.015	1.142	1.46E-02	4.56E-02
CR1	Complement receptor type 1	1.224	1.157	1.295	2.67E-12	3.03E-11
CRACR2A	EF-hand calcium-binding domain-containing protein 4B	1.183	1.116	1.254	1.73E-08	0.000000138
CREB3	Cyclic AMP-responsive element-binding protein 3	1.159	1.095	1.227	4.13E-07	0.0000029
CREG1	Protein CREG1	1.105	1.038	1.176	0.001743591	6.80E-03
CRHR1	Corticotropin-releasing factor receptor 1	1.177	1.104	1.254	0.00000059	4.03E-06
CRIM1	Cysteine-rich motor neuron 1 protein	1.176	1.119	1.236	1.66E-10	1.63E-09
CRISP2	Cysteine-rich secretory protein 2	1.109	1.048	1.174	3.31E-04	1.52E-03
CRISP3	Cysteine-rich secretory protein 3	1.125	1.060	1.194	9.94E-05	0.000488968
CRKL	Crk-like protein	1.118	1.055	1.185	1.65E-04	0.000783513
CRLF1	Cytokine receptor-like factor 1	1.161	1.089	1.237	0.00000404	2.44E-05
CRNN	Cornulin	1.234	1.151	1.322	2.48E-09	2.20E-08
CRTAC1	Cartilage acidic protein 1	1.403	1.307	1.506	6.28E-21	1.64E-19
CRTAP	Cartilage-associated protein	1.480	1.412	1.552	2.45E-59	1.79E-56
CRX	Cone-rod homeobox protein	1.226	1.159	1.297	1.62E-12	1.9E-11
CRYBB1	Beta-crystallin B1	1.316	1.268	1.367	1.77E-46	5.17E-44
CRYBB2	Beta-crystallin B2	1.308	1.238	1.382	1.77E-21	5.06E-20
CRYGD	Gamma-crystallin D	1.145	1.082	1.212	0.00000319	1.96E-05
CRYM	Ketimine reductase mu-crystallin	1.157	1.099	1.219	3.02E-08	2.35E-07
CSF1	Macrophage colony-stimulating factor 1	1.121	1.061	1.184	4.69E-05	2.43E-04
CSF1R	Macrophage colony-stimulating factor 1 receptor	1.091	1.034	1.151	1.36E-03	0.005397499
CSF2	Granulocyte-macrophage colony-stimulating factor	1.248	1.185	1.315	5.13E-17	9.49E-16
CSF2RB	Cytokine receptor common subunit beta	1.259	1.187	1.335	1.15E-14	1.73E-13
CSF3	Granulocyte colony-stimulating factor	1.294	1.218	1.375	6.64E-17	1.21E-15
CSF3R	Granulocyte colony-stimulating factor receptor	1.188	1.121	1.259	5.95E-09	4.97E-08
CSNK2A1	Casein kinase II subunit alpha	1.217	1.151	1.287	5.95E-12	6.56E-11
CSPG4	Chondroitin sulfate proteoglycan 4	1.264	1.190	1.343	3.70E-14	5.05E-13
CSPG5	Chondroitin sulfate proteoglycan 5	1.157	1.092	1.226	6.70E-07	0.00000454
CSRP3	Cysteine and glycine-rich protein 3	1.077	1.019	1.139	0.008586048	2.86E-02
CST1	Cystatin-SN	1.149	1.090	1.211	0.000000228	1.67E-06
CST3	Cystatin-C	1.110	1.056	1.167	3.95E-05	2.08E-04
CST7	Cystatin-F	1.101	1.032	1.174	3.48E-03	1.27E-02
CSTB	Cystatin-B	1.150	1.082	1.223	8.31E-06	0.0000484
CTAG1A CTAG1B	Cancer/testis antigen 1	1.307	1.239	1.380	2.13E-22	6.7E-21
CTBS	Di-N-acetylchitobiase	1.169	1.097	1.246	0.0000016	1.03E-05

CTHRC1	Collagen triple helix repeat-containing protein 1	1.174	1.110	1.241	1.84E-08	1.46E-07
CTRB1	Chymotrypsinogen B	1.193	1.126	1.263	2.16E-09	1.94E-08
CTSB	Cathepsin B	1.247	1.165	1.335	2.01E-10	1.96E-09
CTSC	Dipeptidyl peptidase 1	0.868	0.814	0.927	1.97E-05	0.000109319
CTSD	Cathepsin D	1.171	1.100	1.246	6.49E-07	0.00000442
CTSF	Cathepsin F	1.070	1.013	1.129	0.014701751	4.57E-02
CTSS	Cathepsin S	1.335	1.264	1.411	6.71E-25	2.58E-23
CTSZ	Cathepsin Z	1.076	1.017	1.138	1.10E-02	3.56E-02
CUZD1	CUB and zona pellucida-like domain-containing protein 1	1.117	1.052	1.187	2.86E-04	1.31E-03
CXCL1	Growth-regulated alpha protein	1.223	1.157	1.293	1.24E-12	1.46E-11
CXCL12	Stromal cell-derived factor 1	1.221	1.164	1.281	3.86E-16	6.56E-15
CXCL14	C-X-C motif chemokine 14	1.122	1.059	1.189	0.0000911	4.51E-04
DAG1	Dystroglycan	1.094	1.025	1.168	0.00724819	2.45E-02
DAND5	DAN domain family member 5	0.860	0.807	0.917	3.55E-06	2.16E-05
DAPK2	Death-associated protein kinase 2	1.230	1.155	1.311	1.53E-10	1.51E-09
DBH	Dopamine beta-hydroxylase	0.893	0.838	0.952	4.87E-04	0.002134031
DCN	Decorin	1.179	1.101	1.263	2.50E-06	0.0000156
DCTD	Deoxycytidylate deaminase	1.095	1.032	1.163	0.002866721	1.07E-02
DCTN2	Dynactin subunit 2	1.300	1.234	1.369	4.92E-23	1.65E-21
DCTPP1	dCTP pyrophosphatase 1	1.123	1.064	1.185	2.67E-05	1.45E-04
DDC	Aromatic-L-amino-acid decarboxylase	1.111	1.048	1.177	4.17E-04	1.87E-03
DDR1	Epithelial discoidin domain-containing receptor 1	0.878	0.825	0.935	4.44E-05	0.00023161
DDT	D-dopachrome decarboxylase	0.928	0.874	0.986	1.58E-02	0.04897297
DDX39A	ATP-dependent RNA helicase DDX39A	1.153	1.091	1.219	0.000000422	2.95E-06
DDX4	Probable ATP-dependent RNA helicase DDX4	1.216	1.166	1.269	1.89E-19	4.29E-18
DECR1	2,4-dienoyl-CoA reductase, mitochondrial	1.084	1.020	1.152	9.93E-03	3.25E-02
DEFA1 DEFA1B	Neutrophil defensin 1	1.279	1.211	1.351	1.01E-18	2.18E-17
DEFB104A DEFB104B	Beta-defensin 104	1.173	1.106	1.243	9.25E-08	0.000000697
DEFB116	Beta-defensin 116	1.193	1.125	1.266	3.92E-09	3.42E-08
DEFB118	Beta-defensin 118	1.145	1.077	1.218	0.0000156	8.77E-05
DENND2B	DENN domain-containing protein 2B	1.103	1.036	1.175	0.002072265	7.94E-03
DENR	Density-regulated protein	1.214	1.147	1.286	2.30E-11	2.42E-10
DHPS	Deoxyhypusine synthase	1.225	1.159	1.295	5.64E-13	6.84E-12
DHRS4L2	Dehydrogenase/reductase SDR family member 4-like 2	1.327	1.263	1.394	2.56E-29	1.39E-27
DIABLO	Diablo homolog, mitochondrial	1.150	1.079	1.226	1.74E-05	0.0000973
DIPK1C	Divergent protein kinase domain 1C	1.226	1.160	1.296	5.27E-13	6.41E-12
DKK1	Dickkopf-related protein 1	1.308	1.227	1.395	1.63E-16	2.87E-15
DLG4	Disks large homolog 4	1.398	1.328	1.471	7.77E-38	8.11E-36
DLGAP5	Disks large-associated protein 5	1.160	1.091	1.234	2.38E-06	1.49E-05
DLK1	Protein delta homolog 1	1.100	1.039	1.165	1.16E-03	0.004684132
DLL1	Delta-like protein 1	1.256	1.191	1.324	4.40E-17	8.19E-16
DMP1	Dentin matrix acidic phosphoprotein 1	1.265	1.139	1.405	0.0000109	6.28E-05
DNAJA2	DnaJ homolog subfamily A member 2	1.269	1.197	1.344	6.49E-16	1.08E-14
DNAJA4	DnaJ homolog subfamily A member 4	1.125	1.057	1.199	2.42E-04	1.13E-03
DNAJB1	DnaJ homolog subfamily B member 1	1.154	1.085	1.227	5.34E-06	3.18E-05
DNAJB14	DnaJ homolog subfamily B member 14	1.145	1.085	1.208	7.44E-07	0.00000499
DNAJB6	DnaJ homolog subfamily B member 6	1.511	1.420	1.608	4.79E-39	6.08E-37
DNAJB8	DnaJ homolog subfamily B member 8	1.100	1.037	1.167	0.001621838	6.35E-03
DNER	Delta and Notch-like epidermal growth factor-related receptor	1.150	1.083	1.222	0.00000568	3.37E-05
DNLZ	DNL-type zinc finger protein	1.090	1.022	1.162	8.77E-03	2.91E-02
DNM1	Dynamin-1	1.327	1.245	1.414	2.70E-18	5.71E-17
DNM3	Dynamin-3	1.081	1.015	1.150	1.47E-02	0.045716188
DNMBP	Dynamin-binding protein	1.147	1.076	1.222	2.52E-05	0.000137419
DNPEP	Aspartyl aminopeptidase	1.416	1.336	1.501	1.23E-31	7.98E-30
DOCK9	Dedicator of cytokinesis protein 9	1.113	1.034	1.199	0.004459844	1.60E-02
DOK2	Docking protein 2	1.163	1.095	1.236	9.77E-07	6.40E-06
DPEP2	Dipeptidase 2	1.287	1.214	1.363	1.19E-17	2.32E-16
DPP4	Dipeptidyl peptidase 4	1.127	1.061	1.198	1.07E-04	0.000521508
DPP6	Dipeptidyl aminopeptidase-like protein 6	0.843	0.793	0.895	3.53E-08	0.000000274
DPY30	Protein dpy-30 homolog	1.104	1.040	1.172	0.001150854	4.67E-03
DRAXIN	Draxin	1.102	1.039	1.170	0.001285363	5.15E-03
DRG2	Developmentally-regulated GTP-binding protein 2	1.567	1.470	1.670	1.73E-43	3.36E-41
DSC2	Desmocollin-2	1.162	1.098	1.229	1.88E-07	1.38E-06
DSCAM	Down syndrome cell adhesion molecule	1.338	1.273	1.406	2.22E-30	1.3E-28
DSG4	Desmoglein-4	1.239	1.180	1.302	1.57E-17	3.04E-16
DTNB	Dystrobrevin beta	1.149	1.084	1.217	0.00000253	1.57E-05
DTX2	Probable E3 ubiquitin-protein ligase DTX2	1.206	1.143	1.271	4.68E-12	5.21E-11
DUSP29	Dual specificity phosphatase 29	1.079	1.016	1.145	1.27E-02	4.03E-02
DUSP3	Dual specificity protein phosphatase 3	1.194	1.137	1.254	1.16E-12	1.38E-11
DYNLT1	Dynein light chain Tctex-type 1	1.161	1.095	1.232	6.63E-07	0.0000045
ECE1	Endothelin-converting enzyme 1	1.081	1.015	1.151	1.52E-02	0.047203012
ECHS1	Enoyl-CoA hydratase, mitochondrial	1.127	1.059	1.201	0.000185166	8.77E-04
ECM1	Extracellular matrix protein 1	1.322	1.244	1.405	1.87E-19	4.29E-18
ECSCR	Endothelial cell-specific chemotaxis regulator	1.102	1.039	1.168	1.21E-03	4.84E-03
EDDM3B	Epididymal secretory protein E3-beta	1.200	1.131	1.274	2.25E-09	2.00E-08
EDEM2	ER degradation-enhancing alpha-mannosidase-like protein 2	1.089	1.024	1.157	6.27E-03	0.021574248
EDF1	Endothelial differentiation-related factor 1	1.117	1.052	1.186	2.85E-04	0.001310716
EDN1	Endothelin-1	1.146	1.084	1.212	0.00000163	1.05E-05
EFHD1	EF-hand domain-containing protein D1	1.145	1.076	1.218	0.0000178	9.87E-05
EGFR	Epidermal growth factor receptor	1.272	1.193	1.356	2.04E-13	2.57E-12
EIF1AX	Eukaryotic translation initiation factor 1A, X-chromosomal	1.140	1.076	1.207	7.87E-06	4.59E-05
EIF2AK2	Interferon-induced, double-stranded RNA-activated protein kinase	1.128	1.055	1.205	3.85E-04	0.001733934
EIF2S2	Eukaryotic translation initiation factor 2 subunit 2	1.189	1.121	1.261	7.84E-09	6.45E-08
EIF4E	Eukaryotic translation initiation factor 4E	1.107	1.035	1.184	0.002930943	1.09E-02
EIF4EBP1	Eukaryotic translation initiation factor 4E-binding protein 1	1.088	1.021	1.160	0.009408763	3.11E-02
EIF4G3	Eukaryotic translation initiation factor 4 gamma 3	1.152	1.087	1.221	1.98E-06	1.25E-05
EIF5	Eukaryotic translation initiation factor 5	1.310	1.233	1.391	2.04E-18	4.36E-17
EIF5A	Eukaryotic translation initiation factor 5A-1	1.126	1.060	1.196	1.23E-04	0.000595866
ELAC1	Zinc phosphodiesterase ELAC protein 1	1.259	1.174	1.351	1.31E-10	1.3E-09
ELAVL4	ELAV-like protein 4	1.346	1.261	1.438	7.14E-19	1.56E-17
ELN	Elastin	1.088	1.024	1.156	0.006213927	2.14E-02
ELOA	Elongin-A	1.084	1.018	1.155	1.18E-02	3.78E-02
ENO2	Gamma-enolase	1.142	1.078	1.211	6.78E-06	4.00E-05
ENOPH1	Enolase-phosphatase E1	1.159	1.095	1.227	3.61E-07	0.00000257
ENOX2	Ecto-NOX disulfide-thiol exchanger 2	1.118	1.049	1.192	5.99E-04	0.002586205
ENPEP	Glutamyl aminopeptidase	1.321	1.237	1.411	8.96E-17	1.63E-15
ENPP5	Ectonucleotide pyrophosphatase/phosphodiesterase family member 5	0.919	0.859	0.983	0.014382193	4.50E-02
ENTR1	Endosome-associated-trafficking regulator 1	1.330	1.257	1.408	8.58E-23	2.85E-21
EPB41L5	Band 4.1-like protein 5	0.917	0.861	0.977	7.12E-03	2.41E-02
EPCAM	Epithelial cell adhesion molecule	1.084	1.021	1.150	8.08E-03	0.027022034
EPHA1	Ephrin type-A receptor 1	1.313	1.242	1.389	7.80E-22	2.26E-20
EPHB4	Ephrin type-B receptor 4	0.852	0.800	0.908	0.000000893	5.93E-06
EPO	Erythropoietin	1.255	1.184	1.330	1.69E-14	2.47E-13
EPPK1	Epiplakin	1.176	1.101	1.255	1.43E-06	9.28E-06
ERBB2	Receptor tyrosine-protein kinase erbB-2	1.113	1.053	1.177	1.64E-04	7.82E-04
ERBB3	Receptor tyrosine-protein kinase erbB-3	1.303	1.239	1.371	1.81E-24	6.87E-23
ERBB4	Receptor tyrosine-protein kinase erbB-4	1.208	1.151	1.268	1.85E-14	2.68E-13
ERBIN	Erbin	1.290	1.218	1.366	4.11E-18	8.35E-17
ERC2	ERC protein 2	0.865	0.816	0.916	0.000000755	5.04E-06
EREG	Proepiregulin	1.199	1.128	1.275	5.36E-09	4.52E-08
ERP44	Endoplasmic reticulum resident protein 44	1.121	1.054	1.192	2.85E-04	1.31E-03
ESM1	Endothelial cell-specific molecule 1	1.140	1.085	1.199	3.00E-07	0.00000217
ESR1	Estrogen receptor	1.087	1.018	1.160	1.23E-02	0.039250384
ESYT2	Extended synaptotagmin-2	1.258	1.189	1.331	1.21E-15	1.96E-14
EVI2B	Protein EVI2B	1.144	1.086	1.205	0.000000375	2.66E-06
EVPL	Envoplakin	1.277	1.199	1.361	3.29E-14	4.54E-13
EXOSC10	Exosome component 10	1.310	1.250	1.374	4.82E-29	2.47E-27

EXTL1	Exostosin-like 1	1.719	1.617	1.827	3.10E-68	3.02E-65
EZR	Ezrin	1.162	1.095	1.234	9.35E-07	0.00000617
F13B	Coagulation factor XIII B chain	1.098	1.037	1.164	0.00149935	5.91E-03
F2	Prothrombin	1.179	1.112	1.250	0.000000039	3.02E-07
F2R	Proteinase-activated receptor 1	1.237	1.169	1.310	2.31E-13	2.90E-12
FABP1	Fatty acid-binding protein, liver	1.125	1.059	1.195	1.28E-04	6.17E-04
FABP2	Fatty acid-binding protein, intestinal	1.093	1.032	1.158	2.49E-03	0.009435964
FABP4	Fatty acid-binding protein, adipocyte	1.798	1.710	1.891	2.10E-116	3.06E-113
FADD	FAS-associated death domain protein	1.237	1.165	1.314	3.83E-12	4.29E-11
FAM3C	Protein FAM3C	1.097	1.030	1.168	0.003901156	1.41E-02
FAM3D	Protein FAM3D	1.358	1.282	1.438	2.53E-25	9.98E-24
FAS	Tumor necrosis factor receptor superfamily member 6	0.859	0.810	0.912	5.16E-07	3.54E-06
FBLN2	Fibulin-2	1.199	1.128	1.274	4.83E-09	4.13E-08
FBP1	Fructose-1,6-bisphosphatase 1	1.158	1.091	1.228	1.40E-06	0.00000909
FCAMR	High affinity immunoglobulin alpha and immunoglobulin mu Fc receptor	1.120	1.048	1.196	0.000852467	3.56E-03
FCAR	Immunoglobulin alpha Fc receptor	1.095	1.028	1.166	0.004811939	1.72E-02
FCER1A	High affinity immunoglobulin epsilon receptor subunit alpha	1.067	1.012	1.125	1.61E-02	4.97E-02
FCN1	Ficolin-1	1.187	1.114	1.265	1.25E-07	9.37E-07
FCRL6	Fc receptor-like protein 6	1.089	1.029	1.153	3.24E-03	0.011926621
FCRLB	Fc receptor-like B	1.108	1.046	1.174	4.61E-04	0.002040144
FDX2	Ferredoxin-2, mitochondrial	1.195	1.134	1.259	2.85E-11	2.98E-10
FEN1	Flap endonuclease 1	1.080	1.018	1.145	0.010738426	3.48E-02
FETUB	Fetuin-B	1.088	1.020	1.160	1.05E-02	3.42E-02
FGF16	Fibroblast growth factor 16	1.138	1.072	1.208	2.29E-05	1.26E-04
FGF19	Fibroblast growth factor 19	1.090	1.025	1.159	5.75E-03	0.020003537
FGF7	Fibroblast growth factor 7	1.406	1.348	1.466	2.48E-57	1.21E-54
FGF9	Fibroblast growth factor 9	1.248	1.181	1.318	2.46E-15	3.83E-14
FGFBP1	Fibroblast growth factor-binding protein 1	1.169	1.097	1.247	0.00000163	1.05E-05
FGFBP2	Fibroblast growth factor-binding protein 2	1.251	1.185	1.320	3.97E-16	6.71E-15
FGFBP3	Fibroblast growth factor-binding protein 3	1.285	1.207	1.368	3.28E-15	5.07E-14
FGFR4	Fibroblast growth factor receptor 4	1.336	1.271	1.405	1.44E-29	8.28E-28
FGL1	Fibrinogen-like protein 1	1.313	1.233	1.399	3.32E-17	6.26E-16
FGR	Tyrosine-protein kinase Fgr	1.208	1.132	1.290	1.53E-08	1.23E-07
FHIT	Bis(5'-adenosyl)-triphosphatase	1.140	1.072	1.211	0.0000279	1.51E-04
FKBP14	Peptidyl-prolyl cis-trans isomerase FKBP14	1.217	1.160	1.277	1.27E-15	2.04E-14
FKBP5	Peptidyl-prolyl cis-trans isomerase FKBP5	1.119	1.044	1.200	1.56E-03	6.13E-03
FKBP7	Peptidyl-prolyl cis-trans isomerase FKBP7	1.094	1.036	1.155	1.18E-03	0.004759225
FKBPL	FK506-binding protein-like	1.471	1.387	1.560	6.77E-38	7.33E-36
FLI1	Friend leukemia integration 1 transcription factor	1.119	1.055	1.187	0.000172221	8.19E-04
FLRT2	Leucine-rich repeat transmembrane protein FLRT2	1.110	1.046	1.178	0.000545945	2.37E-03
FMNL1	Formin-like protein 1	1.208	1.138	1.281	3.55E-10	3.42E-09
FOLR1	Folate receptor alpha	0.926	0.872	0.983	1.14E-02	3.68E-02
FOLR3	Folate receptor gamma	1.169	1.112	1.229	7.12E-10	6.52E-09
FOSB	Protein fosB	0.921	0.869	0.975	4.95E-03	0.017569033
FRMD4B	FERM domain-containing protein 4B	0.927	0.872	0.986	0.016225353	5.00E-02
FST	Follistatin	1.134	1.066	1.206	0.0000669	3.38E-04
FSTL1	Follistatin-related protein 1	1.096	1.031	1.165	3.25E-03	1.19E-02
FUT1	Galactoside alpha-(1,2)-fucosyltransferase 1	1.080	1.023	1.141	5.72E-03	2.00E-02
FUT3_FUT5	3-galactosyl-N-acetylglucosaminide 4-alpha-L-fucosyltransferase FUT3_4-galactosyl	1.196	1.125	1.271	1.03E-08	8.43E-08
FUT8	Alpha-(1,6)-fucosyltransferase	1.077	1.015	1.143	1.48E-02	0.045921744
FZD10	Frizzled-10	1.144	1.080	1.211	0.00000494	2.96E-05
FZD8	Frizzled-8	0.900	0.845	0.958	0.00101146	4.15E-03
GABARAPL1	Gamma-aminobutyric acid receptor-associated protein-like 1	1.144	1.085	1.207	7.22E-07	4.85E-06
GAD1	Glutamate decarboxylase 1	1.261	1.177	1.351	5.26E-11	5.36E-10
GADD45B	Growth arrest and DNA damage-inducible protein GADD45 beta	1.121	1.057	1.190	1.60E-04	0.000766821
GADD45GIP1	Growth arrest and DNA damage-inducible proteins-interacting protein 1	1.177	1.118	1.240	6.56E-10	6.08E-09
GAGE2A	G antigen 2A	1.096	1.028	1.169	0.005115361	1.81E-02
GAMT	Guanidinoacetate N-methyltransferase	1.163	1.067	1.268	0.000586018	2.53E-03
GAS2	Growth arrest-specific protein 2	1.250	1.188	1.315	7.84E-18	1.55E-16
GAS6	Growth arrest-specific protein 6	1.093	1.042	1.148	3.08E-04	1.41E-03
GASK1A	Golgi-associated kinase 1A	1.178	1.110	1.251	7.23E-08	0.000000552
GAST	Gastrin	1.175	1.104	1.250	4.04E-07	0.00000284
GBA	Lysosomal acid glucosylceramidase	1.112	1.045	1.183	0.000771279	3.25E-03
GBP2	Guanylate-binding protein 2	1.154	1.091	1.220	0.000000459	3.20E-06
GBP4	Guanylate-binding protein 4	1.077	1.016	1.142	1.28E-02	4.06E-02
GC	Vitamin D-binding protein	1.170	1.093	1.252	5.89E-06	3.49E-05
GCG	Pro-glucagon	1.136	1.072	1.204	1.54E-05	0.0000863
GCNT1	Beta-1,3-galactosyl-O-glycosyl-glycoprotein beta-1,6-N-acetylglucosaminyltransferas	1.223	1.153	1.298	3.03E-11	3.14E-10
GDF2	Growth/differentiation factor 2	0.886	0.829	0.946	0.000299184	1.37E-03
GFRA1	GDNF family receptor alpha-1	1.098	1.040	1.159	0.000656289	2.80E-03
GFRA2	GDNF family receptor alpha-2	1.246	1.174	1.322	4.12E-13	5.04E-12
GFRA3	GDNF family receptor alpha-3	1.132	1.073	1.195	6.40E-06	3.79E-05
GGA1	ADP-ribosylation factor-binding protein GGA1	0.903	0.849	0.960	1.10E-03	0.004474762
GGACT	Gamma-glutamylaminecyclotransferase	1.216	1.147	1.290	7.73E-11	7.82E-10
GGCT	Gamma-glutamylcyclotransferase	1.256	1.179	1.338	1.63E-12	1.90E-11
GGH	Gamma-glutamyl hydrolase	1.090	1.030	1.153	0.002766732	1.03E-02
GGT1	Glutathione hydrolase 1 proenzyme	1.244	1.181	1.311	2.58E-16	4.46E-15
GGT5	Glutathione hydrolase 5 proenzyme	1.228	1.165	1.295	3.06E-14	4.23E-13
GH1	Somatotropin	1.240	1.173	1.310	1.79E-14	2.61E-13
GH2	Growth hormone variant	1.190	1.122	1.262	5.98E-09	4.98E-08
GHR	Growth hormone receptor	1.444	1.369	1.523	2.59E-41	3.98E-39
GHRHR	Growth hormone-releasing hormone receptor	1.189	1.128	1.253	1.07E-10	1.07E-09
GHRL	Appetite-regulating hormone	0.838	0.786	0.894	7.88E-08	5.95E-07
GID8	Glucose-induced degradation protein 8 homolog	1.267	1.201	1.337	3.31E-18	6.81E-17
GIMAP7	GTPase IMAP family member 7	1.172	1.109	1.238	2.06E-08	0.000000162
GIPC3	PDZ domain-containing protein GIPC3	1.083	1.023	1.147	6.33E-03	0.021750505
GIPR	Gastric inhibitory polypeptide receptor	1.272	1.207	1.340	1.76E-19	4.08E-18
GJA8	Gap junction alpha-8 protein	1.119	1.057	1.185	0.000123088	5.97E-04
GKN1	Gastrokine-1	1.148	1.079	1.221	1.10E-05	6.33E-05
GLA	Alpha-galactosidase A	1.165	1.099	1.236	3.87E-07	2.73E-06
GLI2	Zinc finger protein GLI2	1.078	1.022	1.136	5.76E-03	0.020015067
GLO1	Lactoylglutathione lyase	1.221	1.155	1.290	1.65E-12	1.93E-11
GLOD4	Glyoxalase domain-containing protein 4	1.305	1.237	1.376	1.71E-22	5.56E-21
GLYR1	Putative oxidoreductase GLYR1	1.097	1.037	1.161	0.001348009	5.37E-03
GMPR	GMP reductase 1	0.903	0.849	0.960	1.06E-03	4.33E-03
GMPR2	GMP reductase 2	1.112	1.052	1.176	1.94E-04	9.15E-04
GNGT1	Guanine nucleotide-binding protein G(T) subunit gamma-T1	1.244	1.170	1.323	2.91E-12	3.3E-11
GNPDA1	Glucosamine-6-phosphate isomerase 1	1.381	1.315	1.451	3.83E-38	4.31E-36
GNPDA2	Glucosamine-6-phosphate isomerase 2	1.149	1.083	1.220	0.00000511	3.05E-05
GOLGA3	Golgin subfamily A member 3	1.373	1.288	1.464	3.53E-22	1.06E-20
GOLM2	Protein GOLM2	1.160	1.094	1.228	5.18E-07	3.54E-06
GORASP2	Golgi reassembly-stacking protein 2	1.100	1.033	1.172	3.00E-03	1.11E-02
GOT1	Aspartate aminotransferase, cytoplasmic	1.091	1.031	1.154	2.43E-03	0.009201835
GP1BA	Platelet glycoprotein Ib alpha chain	1.076	1.015	1.141	1.45E-02	0.045411918
GP6	Platelet glycoprotein VI	1.230	1.163	1.301	5.97E-13	7.21E-12
GPA33	Cell surface A33 antigen	0.909	0.857	0.964	0.001415874	5.60E-03
GPD1	Glycerol-3-phosphate dehydrogenase	0.896	0.845	0.950	2.17E-04	1.01E-03
GPHA2	Glycoprotein hormone alpha-2	1.302	1.223	1.387	1.78E-16	3.11E-15
GPKOW	G-patch domain and KOW motifs-containing protein	1.106	1.048	1.168	2.66E-04	0.001227632
GPR101	Probable G-protein coupled receptor 101	1.230	1.149	1.316	2.61E-09	0.000000023
GPR15L	Protein GPR15L	1.117	1.051	1.188	0.000347935	1.58E-03
GRHPR	Glyoxylate reductase/hydroxypyruvate reductase	1.139	1.081	1.199	0.000000911	6.04E-06
GRN	Progranulin	1.127	1.058	1.201	2.11E-04	9.90E-04
GRPEL1	GrpE protein homolog 1, mitochondrial	1.143	1.072	1.219	4.67E-05	2.42E-04
GSAP	Gamma-secretase-activating protein	1.229	1.164	1.298	1.28E-13	1.68E-12
GSR	Glutathione reductase, mitochondrial	1.138	1.075	1.206	1.08E-05	0.0000623
GSTA1	Glutathione S-transferase A1	1.158	1.089	1.231	0.00000261	1.62E-05

GSTT2B	Glutathione S-transferase theta-2B	1.115	1.047	1.186	0.000633259	2.71E-03
GTF2IRD1	General transcription factor II-I repeat domain-containing protein 1	1.274	1.198	1.355	1.32E-14	1.97E-13
GTPBP2	GTP-binding protein 2	1.231	1.170	1.295	1.39E-15	2.21E-14
GUCY2C	Heat-stable enterotoxin receptor	1.193	1.112	1.280	8.53E-07	0.00000568
GUK1	Guanylate kinase	1.101	1.038	1.168	1.44E-03	0.00567823
GUSB	Beta-glucuronidase	1.203	1.128	1.284	1.75E-08	1.40E-07
GZMB	Granzyme B	1.328	1.252	1.408	2.81E-21	7.89E-20
HARS1	Histidine--tRNA ligase, cytoplasmic	1.191	1.120	1.267	2.61E-08	2.04E-07
HAVCR2	Hepatitis A virus cellular receptor 2	1.162	1.096	1.232	4.83E-07	3.35E-06
HBEGF	Proheparin-binding EGF-like growth factor	1.213	1.139	1.292	2.14E-09	1.93E-08
HCG22	Protein PBMUCL2	1.118	1.058	1.181	7.25E-05	0.000364671
HCLS1	Hematopoietic lineage cell-specific protein	1.188	1.121	1.260	6.16E-09	5.12E-08
HEG1	Protein HEG homolog 1	1.283	1.204	1.366	9.52E-15	1.44E-13
HEPACAM2	HEPACAM family member 2	1.327	1.250	1.409	2.47E-20	6.23E-19
HEPH	Hephaestin	1.125	1.059	1.194	1.19E-04	5.77E-04
HGS	Hepatocyte growth factor-regulated tyrosine kinase substrate	1.260	1.179	1.347	1.15E-11	1.25E-10
HIP1R	Huntingtin-interacting protein 1-related protein	1.312	1.236	1.393	5.39E-19	1.19E-17
HK2	Hexokinase-2	1.083	1.020	1.150	0.009596441	3.16E-02
HLA-E	HLA class I histocompatibility antigen, alpha chain E	1.088	1.027	1.152	0.004206875	1.51E-02
HMBS	Porphobilinogen deaminase	1.301	1.235	1.371	3.52E-23	1.19E-21
HMCN2	Hemicentin-2	1.199	1.132	1.270	5.97E-10	5.58E-09
HMGCS1	Hydroxymethylglutaryl-CoA synthase, cytoplasmic	1.114	1.043	1.190	1.33E-03	0.005325244
HMMR	Hyaluronan mediated motility receptor	1.151	1.087	1.218	1.21E-06	0.00000792
HMOX1	Heme oxygenase 1	1.194	1.124	1.269	1.08E-08	8.81E-08
HNF1A	Hepatocyte nuclear factor 1-alpha	1.300	1.241	1.362	3.03E-28	1.45E-26
HNMT	Histamine N-methyltransferase	1.360	1.284	1.439	5.58E-26	2.23E-24
HNRNPK	Heterogeneous nuclear ribonucleoprotein K	1.306	1.233	1.384	1.88E-19	4.29E-18
HS1BP3	HCLS1-binding protein 3	1.119	1.058	1.183	7.80E-05	0.000390369
HS3ST3B1	Heparan sulfate glucosamine 3-O-sulfotransferase 3B1	1.191	1.131	1.253	1.94E-11	2.06E-10
HSBP1	Heat shock factor-binding protein 1	1.259	1.190	1.333	1.58E-15	2.46E-14
HSD11B1	Corticosteroid 11-beta-dehydrogenase isozyme 1	1.204	1.126	1.287	5.32E-08	4.09E-07
HTRA2	Serine protease HTRA2, mitochondrial	1.093	1.035	1.154	1.50E-03	5.91E-03
ICA1	Islet cell autoantigen 1	1.113	1.048	1.182	4.92E-04	2.15E-03
ICAM3	Intercellular adhesion molecule 3	1.246	1.181	1.316	1.49E-15	2.36E-14
IFI30	Gamma-interferon-inducible lysosomal thiol reductase	0.928	0.874	0.986	1.51E-02	0.046774479
IFIT3	Interferon-induced protein with tetratricopeptide repeats 3	1.106	1.044	1.173	0.000641244	2.74E-03
IFNGR2	Interferon gamma receptor 2	1.207	1.142	1.275	2.43E-11	2.54E-10
IFNL1	Interferon lambda-1	1.309	1.237	1.386	1.48E-20	3.79E-19
IFNL2	Interferon lambda-2	1.300	1.239	1.364	6.28E-27	2.70E-25
IFNW1	Interferon omega-1	1.183	1.137	1.232	2.31E-16	4.02E-15
IFT20	Intraflagellar transport protein 20 homolog	1.165	1.097	1.238	7.50E-07	0.00000502
IGBP1	Immunoglobulin-binding protein 1	1.249	1.179	1.322	3.35E-14	4.59E-13
IGDCC3	Immunoglobulin superfamily DCC subclass member 3	1.159	1.093	1.229	0.000000954	6.28E-06
IGF1R	Insulin-like growth factor 1 receptor	1.281	1.219	1.347	1.98E-22	6.30E-21
IGF2BP3	Insulin-like growth factor 2 mRNA-binding protein 3	1.187	1.115	1.263	7.49E-08	5.69E-07
IGFBP1	Insulin-like growth factor-binding protein 1	1.154	1.091	1.220	4.60E-07	0.0000032
IGFBP2	Insulin-like growth factor-binding protein 2	1.288	1.226	1.352	6.02E-24	2.2E-22
IGFBP3	Insulin-like growth factor-binding protein 3	1.136	1.069	1.208	0.0000385	2.04E-04
IGFBP7	Insulin-like growth factor-binding protein 7	1.269	1.198	1.343	4.56E-16	7.66E-15
IGLC2	Immunoglobulin lambda constant 2	1.152	1.084	1.223	4.50E-06	2.71E-05
IGLON5	IgLON family member 5	1.315	1.253	1.381	1.48E-28	7.31E-27
IGSF3	Immunoglobulin superfamily member 3	1.268	1.197	1.344	7.68E-16	1.26E-14
IGSF8	Immunoglobulin superfamily member 8	1.279	1.205	1.358	1.02E-15	1.65E-14
IL10RA	Interleukin-10 receptor subunit alpha	1.138	1.069	1.213	0.000059	3.02E-04
IL10RB	Interleukin-10 receptor subunit beta	1.243	1.173	1.318	2.43E-13	3.04E-12
IL11	Interleukin-11	1.206	1.158	1.256	1.59E-19	3.73E-18
IL12B	Interleukin-12 subunit beta	1.289	1.227	1.353	3.15E-24	1.18E-22
IL12RB1	Interleukin-12 receptor subunit beta-1	1.189	1.155	1.224	1.07E-31	7.08E-30
IL13	Interleukin-13	1.308	1.236	1.383	9.89E-21	2.56E-19
IL13RA1	Interleukin-13 receptor subunit alpha-1	1.324	1.249	1.403	4.73E-21	1.28E-19
IL13RA2	Interleukin-13 receptor subunit alpha-2	1.429	1.353	1.509	9.72E-38	9.80E-36
IL15	Interleukin-15	1.225	1.173	1.279	2.60E-20	6.48E-19
IL15RA	Interleukin-15 receptor subunit alpha	1.128	1.064	1.196	4.92E-05	2.54E-04
IL16	Pro-interleukin-16	1.280	1.216	1.348	4.14E-21	1.13E-19
IL17A	Interleukin-17A	1.322	1.259	1.388	2.83E-29	1.51E-27
IL17C	Interleukin-17C	1.426	1.354	1.501	2.06E-41	3.34E-39
IL17D	Interleukin-17D	1.250	1.209	1.292	1.72E-39	2.29E-37
IL17F	Interleukin-17F	1.233	1.166	1.304	2.76E-13	3.43E-12
IL17RA	Interleukin-17 receptor A	1.380	1.313	1.451	8.47E-37	7.74E-35
IL17RB	Interleukin-17 receptor B	1.246	1.182	1.314	5.06E-16	8.45E-15
IL18	Interleukin-18	1.236	1.170	1.305	2.38E-14	3.38E-13
IL18BP	Interleukin-18-binding protein	1.318	1.266	1.373	4.98E-41	7.28E-39
IL19	Interleukin-19	0.917	0.862	0.975	0.005997587	2.07E-02
IL1A	Interleukin-1 alpha	1.253	1.181	1.331	1.49E-13	1.94E-12
IL1B	Interleukin-1 beta	1.257	1.196	1.321	1.25E-19	2.96E-18
IL1R2	Interleukin-1 receptor type 2	1.250	1.170	1.336	4.64E-11	4.76E-10
IL2	Interleukin-2	0.897	0.841	0.957	9.79E-04	0.004036797
IL20	Interleukin-20	1.124	1.061	1.191	0.0000813	4.05E-04
IL22RA1	Interleukin-22 receptor subunit alpha-1	1.266	1.191	1.347	5.88E-14	7.92E-13
IL24	Interleukin-24	1.102	1.039	1.169	1.19E-03	4.78E-03
IL25	Interleukin-25	1.103	1.037	1.173	1.75E-03	6.80E-03
IL2RA	Interleukin-2 receptor subunit alpha	0.908	0.854	0.964	1.74E-03	0.006787625
IL33	Interleukin-33	1.197	1.131	1.268	6.81E-10	6.26E-09
IL34	Interleukin-34	1.135	1.069	1.205	0.000031	1.66E-04
IL4R	Interleukin-4 receptor subunit alpha	1.377	1.304	1.455	1.72E-30	1.07E-28
IL5	Interleukin-5	0.781	0.738	0.828	5.85E-17	1.08E-15
IL6R	Interleukin-6 receptor subunit alpha	1.079	1.016	1.145	1.27E-02	4.04E-02
IL6ST	Interleukin-6 receptor subunit beta	1.108	1.050	1.170	2.00E-04	0.000938535
ILKAP	Integrin-linked kinase-associated serine/threonine phosphatase 2C	1.257	1.190	1.328	2.94E-16	5.06E-15
IMMT	MICOS complex subunit MIC60	1.152	1.084	1.224	0.00000549	3.27E-05
IMPA1	Inositol monophosphatase 1	1.178	1.111	1.249	4.21E-08	3.25E-07
IMPG1	Interphotoreceptor matrix proteoglycan 1	1.198	1.127	1.273	5.64E-09	4.74E-08
INHBB	Inhibin beta B chain	1.181	1.103	1.265	1.97E-06	1.25E-05
INPP5J	Phosphatidylinositol 4,5-bisphosphate 5-phosphatase A	1.444	1.366	1.527	1.77E-38	2.07E-36
INSL3	Insulin-like 3	1.121	1.059	1.188	9.13E-05	0.000450898
INSL4	Early placenta insulin-like peptide	1.081	1.022	1.143	0.006211465	2.14E-02
IPCEF1	Interactor protein for cytohesin exchange factors 1	1.246	1.168	1.329	1.92E-11	2.05E-10
IQGAP2	Ras GTPase-activating-like protein IQGAP2	1.263	1.187	1.344	1.65E-13	2.12E-12
IRAG2	Inositol 1,4,5-triphosphate receptor associated 2	1.114	1.049	1.184	4.86E-04	2.13E-03
IRAK1	Interleukin-1 receptor-associated kinase 1	1.217	1.145	1.292	1.99E-10	1.94E-09
ISM2	Isthmin-2	1.201	1.127	1.279	1.47E-08	0.000000119
IST1	IST1 homolog	1.445	1.377	1.516	6.3E-51	2.63E-48
ITGA11	Integrin alpha-11	0.889	0.832	0.950	0.000488967	2.14E-03
ITGA5	Integrin alpha-5	1.135	1.067	1.208	6.02E-05	3.08E-04
ITGA6	Integrin alpha-6	1.261	1.189	1.338	1.45E-14	2.14E-13
ITGAM	Integrin alpha-M	1.156	1.097	1.219	5.81E-08	0.000000446
ITGAV	Integrin alpha-V	1.271	1.186	1.361	8.49E-12	9.29E-11
ITGB5	Integrin beta-5	1.138	1.080	1.200	0.00000151	9.82E-06
JAM3	Junctional adhesion molecule C	1.132	1.065	1.202	0.0000612	3.12E-04
JPT2	Jupiter microtubule associated homolog 2	1.266	1.197	1.338	1.34E-16	2.38E-15
JUN	Transcription factor AP-1	1.199	1.128	1.273	4.52E-09	3.90E-08
KAZALD1	Kazal-type serine protease inhibitor domain-containing protein 1	1.112	1.044	1.185	9.62E-04	0.003979077
KAZN	Kazrin	1.179	1.109	1.254	1.67E-07	0.00000124
KCNC4	Potassium voltage-gated channel subfamily C member 4	1.228	1.156	1.304	2.22E-11	2.36E-10
KCNIP4	Kv channel-interacting protein 4	1.094	1.025	1.168	0.006943124	2.36E-02
KDM3A	Lysine-specific demethylase 3A	1.165	1.082	1.254	4.77E-05	2.46E-04
KDR	Vascular endothelial growth factor receptor 2	1.146	1.074	1.222	3.64E-05	1.93E-04

KIAA0319	Dyslexia-associated protein KIAA0319	1.297	1.195	1.407	4.68E-10	4.44E-09
KIAA2013	Uncharacterized protein KIAA2013	0.911	0.851	0.976	7.63E-03	0.02563866
KIF22	Kinesin-like protein KIF22	1.168	1.098	1.241	0.000000692	4.67E-06
KIFBP	KIF-binding protein	1.084	1.020	1.151	0.009564816	3.15E-02
KRT17	Keratin, type I cytoskeletal 17	1.096	1.025	1.172	7.10E-03	2.41E-02
LAYN	Layilin	1.149	1.079	1.223	1.43E-05	8.13E-05
LEPR	Leptin receptor	1.157	1.118	1.196	3.55E-17	6.66E-16
LGALS7 LGALS7B	Galectin-7	1.195	1.158	1.234	9.00E-28	4.18E-26
LGALS9	Galectin-9	1.126	1.049	1.208	0.001011624	4.15E-03
LIFR	Leukemia inhibitory factor receptor	1.115	1.049	1.186	0.000471343	2.07E-03
LILRA3	Leukocyte immunoglobulin-like receptor subfamily A member 3	0.872	0.818	0.929	2.59E-05	1.41E-04
LILRA6	Leukocyte immunoglobulin-like receptor subfamily A member 6	1.084	1.018	1.155	1.21E-02	3.88E-02
LMNB1	Lamin-B1	1.115	1.041	1.195	1.94E-03	0.007480123
LPCAT2	Lysophosphatidylcholine acyltransferase 2	1.138	1.059	1.223	4.33E-04	0.001928124
LPL	Lipoprotein lipase	1.244	1.176	1.315	2.4E-14	3.38E-13
LPP	Lipoma-preferred partner	1.114	1.044	1.189	0.001078582	4.40E-03
LRG1	Leucine-rich alpha-2-glycoprotein	0.893	0.832	0.960	2.00E-03	7.67E-03
LSM8	U6 snRNA-associated Sm-like protein LSm8	1.187	1.108	1.271	9.69E-07	6.36E-06
LUZP2	Leucine zipper protein 2	1.286	1.203	1.374	1.51E-13	1.96E-12
LY75	Lymphocyte antigen 75	1.186	1.109	1.269	6.94E-07	0.00000468
LYAR	Cell growth-regulating nucleolar protein	1.075	1.017	1.135	0.010312439	3.36E-02
LYZL2	Lysozyme-like protein 2	1.114	1.050	1.181	0.000338219	1.54E-03
MAPT	Microtubule-associated protein tau	1.129	1.055	1.209	4.81E-04	2.11E-03
MARCO	Macrophage receptor MARCO	1.273	1.195	1.357	1.10E-13	1.45E-12
MAVS	Mitochondrial antiviral-signaling protein	1.097	1.028	1.171	5.21E-03	0.018355022
MAX	Protein max	1.189	1.119	1.264	2.53E-08	0.000000198
MB	Myoglobin	1.227	1.150	1.309	5.83E-10	5.46E-09
MBL2	Mannose-binding protein C	1.331	1.254	1.412	5.02E-21	1.34E-19
MEGF9	Multiple epidermal growth factor-like domains protein 9	0.840	0.781	0.903	2.12E-06	1.34E-05
MENT	Protein MENT	1.104	1.035	1.176	2.53E-03	9.55E-03
MEP1B	Meprin A subunit beta	1.155	1.087	1.228	3.67E-06	0.0000223
MERTK	Tyrosine-protein kinase Mer	0.925	0.871	0.983	1.16E-02	0.037314103
MGMT	Methylated-DNA--protein-cysteine methyltransferase	1.106	1.027	1.192	0.007604109	2.56E-02
MIA	Melanoma-derived growth regulatory protein	1.260	1.196	1.327	3.1E-18	6.46E-17
MICB MICA	MHC class I polypeptide-related sequence A MHC class I polypeptide-related sequer	1.092	1.019	1.170	1.23E-02	3.93E-02
MILR1	Allergin-1	1.176	1.099	1.258	2.66E-06	1.64E-05
MLN	Promotilin	1.345	1.278	1.417	2.44E-29	1.34E-27
MME	Neprilysin	1.174	1.117	1.235	3.88E-10	3.72E-09
MMP1	Interstitial collagenase	1.111	1.055	1.170	0.0000629	3.19E-04
MMP3	Stromelysin-1	1.213	1.127	1.305	0.00000023	1.68E-06
MMUT	Methylmalonyl-CoA mutase, mitochondrial	1.350	1.252	1.456	7.02E-15	1.07E-13
MOCS2	Molybdopterin synthase catalytic subunit	0.868	0.809	0.931	7.95E-05	3.97E-04
MORF4L1	Mortality factor 4-like protein 1	1.078	1.020	1.139	7.75E-03	0.026003757
MORN4	MORN repeat-containing protein 4	0.898	0.836	0.965	3.19E-03	0.011730385
MPHOSPH8	M-phase phosphoprotein 8	1.148	1.079	1.222	0.0000129	7.36E-05
MRPL28	39S ribosomal protein L28, mitochondrial	1.087	1.017	1.161	0.01353376	4.27E-02
MRPL46	39S ribosomal protein L46, mitochondrial	1.239	1.162	1.321	4.86E-11	4.97E-10
MSLN	Mesothelin	1.113	1.044	1.188	1.13E-03	4.59E-03
MSMB	Beta-microseminoprotein	1.195	1.127	1.267	2.91E-09	2.56E-08
MTSS2	Protein MTSS 2	0.910	0.853	0.971	4.27E-03	0.015310638
MUC13	Mucin-13	1.188	1.134	1.244	2.82E-13	3.49E-12
MUC16	Mucin-16	1.116	1.045	1.191	0.001002701	4.13E-03
MVK	Mevalonate kinase	1.230	1.148	1.318	4.33E-09	3.75E-08
MYL1	Myosin light chain 1/3, skeletal muscle isoform	1.114	1.044	1.189	1.08E-03	4.41E-03
MYL3	Myosin light chain 3	1.082	1.016	1.152	1.44E-02	0.045009798
MYL6B	Myosin light chain 6B	1.101	1.034	1.173	2.72E-03	0.010157783
MYOC	Myocilin	1.197	1.117	1.282	0.000000357	2.55E-06
MYOM2	Myomesin-2	1.117	1.051	1.187	0.000341435	1.56E-03
NAA10	N-alpha-acetyltransferase 10	1.380	1.296	1.470	8.50E-24	3.03E-22
NAMPT	Nicotinamide phosphoribosyltransferase	1.097	1.027	1.172	5.90E-03	2.04E-02
NDUFA5	NADH dehydrogenase [ubiquinone] 1 alpha subcomplex subunit 5	0.913	0.854	0.977	7.96E-03	0.026653712
NFKB1	Nuclear factor NF-kappa-B p105 subunit	1.117	1.051	1.187	3.50E-04	0.00158514
NMI	N-myc-interactor	1.261	1.189	1.338	1.11E-14	1.68E-13
NOTCH3	Neurogenic locus notch homolog protein 3	1.116	1.043	1.195	0.001580742	6.19E-03
NPPB	Natriuretic peptides B	1.408	1.324	1.497	1.02E-27	4.64E-26
NPTN	Neuroplastin	1.197	1.138	1.260	4.06E-12	4.53E-11
NPTX2	Neuronal pentraxin-2	1.230	1.164	1.299	1.90E-13	2.42E-12
NPY	Pro-neuropeptide Y	1.113	1.046	1.184	6.79E-04	0.002893696
NT5E	5'-nucleotidase	1.446	1.330	1.572	6.05E-18	1.21E-16
NTF3	Neurotrophin-3	1.074	1.013	1.139	0.015940761	4.92E-02
NUBP1	Cytosolic Fe-S cluster assembly factor NUBP1	0.847	0.789	0.909	4.60E-06	2.76E-05
NUDT5	ADP-sugar pyrophosphatase	0.907	0.841	0.978	1.14E-02	3.69E-02
NXPH3	Neurexophilin-3	1.155	1.077	1.237	4.64E-05	0.000240742
OMG	Oligodendrocyte-myelin glycoprotein	0.847	0.796	0.902	1.73E-07	0.00000127
ORM1	Alpha-1-acid glycoprotein 1	1.341	1.264	1.424	4.54E-22	1.35E-20
OSTN	Osteocrin	1.147	1.068	1.231	0.000154337	7.40E-04
OTOA	Otoancorin	1.169	1.094	1.248	3.29E-06	2.01E-05
OTUD7B	OTU domain-containing protein 7B	1.284	1.204	1.369	2.40E-14	3.38E-13
PACS2	Phosphofurin acidic cluster sorting protein 2	1.119	1.040	1.205	2.67E-03	0.009973193
PADI4	Protein-arginine deiminase type-4	1.094	1.029	1.162	3.92E-03	0.014195507
PAEP	Glycodelin	1.144	1.065	1.229	0.000222934	1.04E-03
PAK4	Serine/threonine-protein kinase PAK 4	1.078	1.019	1.140	0.008569688	2.85E-02
PAMR1	Inactive serine protease PAMR1	1.298	1.211	1.392	1.91E-13	2.42E-12
PCDH17	Protocadherin-17	1.235	1.165	1.310	1.97E-12	2.28E-11
PDCL2	Phosducin-like protein 2	1.145	1.077	1.218	1.46E-05	0.0000826
PDGFC	Platelet-derived growth factor C	1.111	1.052	1.173	1.47E-04	0.000707767
PDIA3	Protein disulfide-isomerase A3	1.101	1.029	1.179	0.005313509	1.87E-02
PDLIM7	PDZ and LIM domain protein 7	1.146	1.076	1.221	0.0000226	1.24E-04
PEAR1	Platelet endothelial aggregation receptor 1	0.870	0.805	0.939	3.89E-04	1.75E-03
PENK	Proenkephalin-A	1.083	1.020	1.150	9.63E-03	3.17E-02
PER3	Period circadian protein homolog 3	1.236	1.165	1.311	2.12E-12	2.44E-11
PGLYRP2	N-acetylmuramoyl-L-alanine amidase	1.222	1.174	1.272	1.12E-22	3.68E-21
PLA2G1B	Phospholipase A2	1.167	1.094	1.245	0.0000028	1.73E-05
PLA2G4A	Cytosolic phospholipase A2	0.919	0.861	0.981	0.010888486	3.53E-02
PLXDC1	Plexin domain-containing protein 1	1.151	1.072	1.236	1.11E-04	5.43E-04
PLXNB2	Plexin-B2	1.100	1.040	1.164	8.39E-04	3.51E-03
PM20D1	N-fatty-acyl-amino acid synthase/hydrolase PM20D1	1.159	1.097	1.224	1.55E-07	0.00000115
PMM2	Phosphomannomutase 2	1.153	1.079	1.232	2.82E-05	0.000151905
PMS1	PMS1 protein homolog 1	1.108	1.036	1.185	0.002915598	1.08E-02
PODXL	Podocalyxin	1.223	1.172	1.276	1.62E-20	4.11E-19
POLR2A	DNA-directed RNA polymerase II subunit RPB1	1.096	1.028	1.169	4.99E-03	1.77E-02
PON2	Serum paraoxonase/arylesterase 2	1.110	1.040	1.186	1.86E-03	7.22E-03
PON3	Serum paraoxonase/lactonase 3	1.097	1.034	1.165	2.30E-03	0.008741822
PPCDC	Phosphopantothenoylcysteine decarboxylase	1.085	1.022	1.151	6.99E-03	0.02370564
PPIB	Peptidyl-prolyl cis-trans isomerase B	1.115	1.045	1.191	0.001053807	4.31E-03
PPIE	Peptidyl-prolyl cis-trans isomerase E	1.269	1.185	1.360	1.1E-11	1.19E-10
PPP1R12B	Protein phosphatase 1 regulatory subunit 12B	0.879	0.822	0.939	1.39E-04	6.71E-04
PPP1R9B	Neurabin-2	1.149	1.104	1.195	6.11E-12	6.72E-11
PRC1	Protein regulator of cytokinesis 1	1.097	1.030	1.169	4.04E-03	0.014576952
PREB	Prolactin regulatory element-binding protein	1.094	1.031	1.161	2.97E-03	0.010985982
PRG2	Bone marrow proteoglycan	1.136	1.070	1.206	0.0000297	1.59E-04
PRKAG3	5'-AMP-activated protein kinase subunit gamma-3	1.320	1.240	1.405	4.25E-18	8.56E-17
PRKD2	Serine/threonine-protein kinase D2	1.120	1.062	1.180	2.58E-05	1.41E-04
PRKG1	cGMP-dependent protein kinase 1	1.128	1.055	1.207	4.08E-04	1.83E-03
PRKRA	Interferon-inducible double-stranded RNA-dependent protein kinase activator A	1.105	1.038	1.177	1.76E-03	0.006837151
PROS1	Vitamin K-dependent protein S	1.221	1.155	1.290	1.20E-12	1.43E-11
PRSS8	Prostasin	1.113	1.044	1.185	0.000954374	3.96E-03

PSAPL1	Proactivator polypeptide-like 1	1.121	1.052	1.194	0.000399178	1.79E-03
PSMD9	26S proteasome non-ATPase regulatory subunit 9	1.122	1.060	1.188	7.54E-05	3.78E-04
PSMG3	Proteasome assembly chaperone 3	1.092	1.026	1.163	6.03E-03	2.08E-02
PTGES2	Prostaglandin E synthase 2	0.882	0.799	0.975	1.36E-02	0.042686541
PTH	Parathyroid hormone	0.894	0.833	0.960	1.91E-03	0.007403533
PTPRS	Receptor-type tyrosine-protein phosphatase S	1.143	1.081	1.208	0.00000243	1.52E-05
PTPRZ1	Receptor-type tyrosine-protein phosphatase zeta	1.110	1.041	1.183	0.001385034	5.49E-03
PTS	6-pyruvoyl tetrahydrobiopterin synthase	1.172	1.089	1.262	2.34E-05	1.28E-04
PVR	Poliovirus receptor	1.110	1.041	1.183	1.37E-03	5.44E-03
PXDNL	Peroxidasin-like protein	1.093	1.027	1.163	5.21E-03	0.018355022
QDPR	Dihydropteridine reductase	1.127	1.061	1.196	1.05E-04	0.000515456
QPCT	Glutaminyl-peptide cyclotransferase	1.285	1.216	1.357	3.91E-19	8.71E-18
QSOX1	Sulfhydryl oxidase 1	1.245	1.185	1.308	2.9E-18	6.10E-17
RAB11FIP3	Rab11 family-interacting protein 3	1.087	1.018	1.162	1.30E-02	4.12E-02
RAB39B	Ras-related protein Rab-39B	1.197	1.118	1.281	2.11E-07	1.54E-06
RAB3GAP1	Rab3 GTPase-activating protein catalytic subunit	1.128	1.069	1.191	1.35E-05	0.0000767
RAD23B	UV excision repair protein RAD23 homolog B	0.817	0.758	0.880	1.26E-07	0.000000942
RASGRF1	Ras-specific guanine nucleotide-releasing factor 1	1.278	1.204	1.358	1.31E-15	2.10E-14
RBFOX3	RNA binding protein fox-1 homolog 3	1.116	1.039	1.198	0.002530144	9.54E-03
RET	Proto-oncogene tyrosine-protein kinase receptor Ret	1.271	1.189	1.359	2.14E-12	2.46E-11
RLN1	Prorelaxin H1	1.168	1.091	1.250	7.82E-06	4.57E-05
ROBO1	Roundabout homolog 1	1.117	1.049	1.188	5.11E-04	0.002226161
RP2	Protein XRP2	1.195	1.125	1.270	9.16E-09	0.000000075
RPA2	Replication protein A 32 kDa subunit	1.108	1.045	1.175	0.000561167	2.43E-03
RRM2B	Ribonucleoside-diphosphate reductase subunit M2 B	1.138	1.063	1.218	0.000211217	9.90E-04
RSPO3	R-spondin-3	1.101	1.043	1.162	4.48E-04	1.99E-03
S100A4	Protein S100-A4	1.217	1.141	1.298	2.22E-09	1.98E-08
S100P	Protein S100-P	1.090	1.021	1.165	1.03E-02	0.033525424
SAP18	Histone deacetylase complex subunit SAP18	0.922	0.865	0.983	1.25E-02	0.039845482
SAT2	Thialysine N-epsilon-acetyltransferase	1.101	1.040	1.166	0.001006941	4.14E-03
SCGB2A2	Mammaglobin-A	1.109	1.028	1.196	0.007450064	2.51E-02
SCPEP1	Retinoid-inducible serine carboxypeptidase	1.342	1.279	1.407	6.36E-34	5.02E-32
SCRN1	Secernin-1	1.243	1.160	1.333	9.30E-10	8.47E-09
SEC31A	Protein transport protein Sec31A	1.234	1.179	1.292	3.18E-19	7.16E-18
SEMA3G	Semaphorin-3G	1.098	1.026	1.175	6.81E-03	0.023246674
SEMA4C	Semaphorin-4C	1.339	1.265	1.418	1.64E-23	5.69E-22
SERPINA1	Alpha-1-antitrypsin	1.193	1.115	1.276	0.000000319	2.30E-06
SERPINA6	Corticosteroid-binding globulin	1.098	1.028	1.172	5.17E-03	1.83E-02
SERPINB9	Serpin B9	1.166	1.098	1.238	4.87E-07	3.36E-06
SERPINC1	Antithrombin-III	1.345	1.267	1.428	2.94E-22	9.03E-21
SERPINF2	Alpha-2-antiplasmin	1.112	1.046	1.182	6.48E-04	0.00277075
SEZ6	Seizure protein 6 homolog	1.098	1.033	1.167	0.00266657	9.97E-03
SH2B3	SH2B adapter protein 3	1.154	1.098	1.212	1.42E-08	1.15E-07
SHC1	SHC-transforming protein 1	1.121	1.053	1.195	3.83E-04	1.73E-03
SHH	Sonic hedgehog protein	1.194	1.137	1.254	9.56E-13	1.15E-11
SIGLEC10	Sialic acid-binding Ig-like lectin 10	1.079	1.019	1.142	9.04E-03	0.029935386
SIGLEC15	Sialic acid-binding Ig-like lectin 15	1.092	1.021	1.167	9.74E-03	0.031953555
SIL1	Nucleotide exchange factor SIL1	1.144	1.070	1.224	0.0000851	4.22E-04
SKAP2	Src kinase-associated phosphoprotein 2	1.136	1.058	1.220	0.000434096	1.93E-03
SKIV2L	Helicase SKI2W	1.109	1.039	1.185	1.94E-03	7.48E-03
SLAMF1	Signaling lymphocytic activation molecule	0.870	0.812	0.933	8.43E-05	4.19E-04
SLC4A1	Band 3 anion transport protein	1.156	1.090	1.226	1.53E-06	0.00000992
SMAD3	Mothers against decapentaplegic homolog 3	1.107	1.048	1.169	2.47E-04	0.001145334
SMNDC1	Survival of motor neuron-related-splicing factor 30	1.266	1.160	1.382	0.000000131	9.78E-07
SMOC2	SPARC-related modular calcium-binding protein 2	0.893	0.834	0.956	0.001161114	4.69E-03
SMPD1	Sphingomyelin phosphodiesterase	1.222	1.146	1.303	7.98E-10	7.29E-09
SNAP29	Synaptosomal-associated protein 29	0.857	0.801	0.917	7.34E-06	4.30E-05
SNAPIN	SNARE-associated protein Snapin	1.091	1.023	1.164	8.28E-03	0.027654901
SNED1	Sushi, nidogen and EGF-like domain-containing protein 1	1.108	1.046	1.174	4.62E-04	0.002040144
SNX15	Sorting nexin-15	1.237	1.171	1.306	2.45E-14	3.42E-13
SNX18	Sorting nexin-18	1.143	1.071	1.219	0.0000565	2.90E-04
SPESP1	Sperm equatorial segment protein 1	1.079	1.019	1.143	8.98E-03	2.98E-02
SPINK5	Serine protease inhibitor Kazal-type 5	1.095	1.036	1.157	1.21E-03	4.84E-03
SPRR1B	Cornifin-B	1.084	1.015	1.157	1.62E-02	0.049984541
SPRY2	Protein sprouty homolog 2	0.916	0.855	0.980	1.15E-02	0.03698835
SPTLC1	Serine palmitoyltransferase 1	1.086	1.024	1.151	0.005791312	2.01E-02
SRPK2	SRSF protein kinase 2	1.299	1.240	1.361	7.72E-28	3.64E-26
SSC5D	Soluble scavenger receptor cysteine-rich domain-containing protein SSC5D	1.147	1.077	1.221	1.67E-05	9.36E-05
ST13	Hsc70-interacting protein	1.084	1.022	1.150	7.54E-03	2.54E-02
ST8SIA1	Alpha-N-acetylneuraminide alpha-2,8-sialyltransferase	1.081	1.016	1.151	1.43E-02	0.04491649
STX16	Syntaxin-16	1.272	1.222	1.324	6.29E-32	4.32E-30
STX1B	Syntaxin-1B	1.132	1.067	1.200	0.0000367	1.95E-04
STX3	Syntaxin-3	1.267	1.201	1.336	3.12E-18	6.47E-17
STX4	Syntaxin-4	1.220	1.177	1.265	3.93E-27	1.74E-25
STX6	Syntaxin-6	1.251	1.177	1.329	3.86E-13	4.73E-12
SWAP70	Switch-associated protein 70	1.118	1.048	1.193	7.11E-04	0.003006724
SYT1	Synaptotagmin-1	1.122	1.058	1.189	1.05E-04	0.000515114
TALDO1	Transaldolase	1.079	1.014	1.147	0.015620137	4.83E-02
TARM1	T-cell-interacting, activating receptor on myeloid cells protein 1	1.132	1.057	1.213	0.000409476	1.83E-03
TBCA	Tubulin-specific chaperone A	1.158	1.085	1.237	1.21E-05	6.94E-05
TBCB	Tubulin-folding cofactor B	1.099	1.027	1.176	6.59E-03	2.26E-02
TBCC	Tubulin-specific chaperone C	1.264	1.190	1.342	2.62E-14	3.65E-13
TCOF1	Treacle protein	1.146	1.099	1.195	1.65E-10	1.62E-09
TDGF1	Teratocarcinoma-derived growth factor 1	1.088	1.021	1.158	0.00900294	2.98E-02
TDO2	Tryptophan 2,3-dioxygenase	1.093	1.029	1.160	0.003536469	1.29E-02
TEF	Thyrotroph embryonic factor	1.272	1.206	1.341	6.05E-19	1.33E-17
TERF1	Telomeric repeat-binding factor 1	1.131	1.069	1.197	2.02E-05	1.12E-04
TET2	Methylcytosine dioxygenase TET2	0.894	0.835	0.957	1.34E-03	0.005350386
TF	Serotransferrin	0.902	0.841	0.968	4.32E-03	0.015468987
TFF3	Trefoil factor 3	1.130	1.049	1.217	0.001316124	5.26E-03
TFPI	Tissue factor pathway inhibitor	0.889	0.829	0.953	0.000877253	3.66E-03
TFPI2	Tissue factor pathway inhibitor 2	1.106	1.034	1.184	3.41E-03	1.25E-02
THBS4	Thrombospondin-4	1.325	1.269	1.384	6.71E-37	6.33E-35
THPO	Thrombopoietin	1.245	1.162	1.334	4.48E-10	4.27E-09
THRAP3	Thyroid hormone receptor-associated protein 3	1.088	1.029	1.150	3.17E-03	0.011682497
TIMM8A	Mitochondrial import inner membrane translocase subunit Tim8 A	0.914	0.854	0.978	0.009695561	3.18E-02
TK1	Thymidine kinase, cytosolic	1.122	1.063	1.184	0.0000295	1.59E-04
TLR4	Toll-like receptor 4	1.088	1.022	1.158	8.49E-03	2.83E-02
TMEM25	Transmembrane protein 25	1.109	1.044	1.178	7.69E-04	3.24E-03
TMOD4	Tropomodulin-4	1.100	1.044	1.159	3.51E-04	0.001585445
TNF	Tumor necrosis factor	1.105	1.052	1.161	7.51E-05	0.000377309
TNFAIP8L2	Tumor necrosis factor alpha-induced protein 8-like protein 2	1.093	1.022	1.169	0.009397094	3.11E-02
TNFRSF11B	Tumor necrosis factor receptor superfamily member 11B	1.154	1.074	1.240	0.0000897	4.44E-04
TNFRSF1B	Tumor necrosis factor receptor superfamily member 1B	1.112	1.056	1.172	6.25E-05	3.18E-04
TNFRSF21	Tumor necrosis factor receptor superfamily member 21	1.247	1.163	1.336	3.78E-10	3.63E-09
TNFSF11	Tumor necrosis factor ligand superfamily member 11	1.131	1.053	1.215	7.08E-04	0.002998824
TOMM20	Mitochondrial import receptor subunit TOM20 homolog	1.082	1.017	1.151	1.27E-02	0.040329027
TOP1MT	DNA topoisomerase I, mitochondrial	1.085	1.016	1.159	0.015161094	4.70E-02
TOP2B	DNA topoisomerase 2-beta	1.147	1.066	1.234	0.000251344	1.16E-03
TP53INP1	Tumor protein p53-inducible nuclear protein 1	1.076	1.019	1.135	7.90E-03	2.65E-02
TPSD1	Tryptase delta	1.091	1.028	1.159	4.10E-03	1.47E-02
TRIM25	E3 ubiquitin/ISG15 ligase TRIM25	1.129	1.071	1.190	6.64E-06	0.0000392
TRIM26	Tripartite motif-containing protein 26	1.139	1.071	1.211	3.31E-05	0.000176073
TSC1	Hamartin	1.074	1.014	1.138	0.014677409	4.57E-02
TSLP	Thymic stromal lymphopoietin	1.124	1.080	1.171	1.22E-08	9.87E-08
TXNDC15	Thioredoxin domain-containing protein 15	1.098	1.032	1.167	3.01E-03	1.11E-02
TXNDC5	Thioredoxin domain-containing protein 5	1.110	1.041	1.183	1.39E-03	5.51E-03

TXNL1	Thioredoxin-like protein 1	1.279	1.201	1.362	2.28E-14	3.25E-13
TXNRD1	Thioredoxin reductase 1, cytoplasmic	1.091	1.024	1.162	6.87E-03	0.023421446
TYRO3	Tyrosine-protein kinase receptor TYRO3	1.136	1.063	1.215	0.000178478	8.47E-04
UBAC1	Ubiquitin-associated domain-containing protein 1	1.102	1.032	1.176	0.003508665	1.28E-02
VAT1	Synaptic vesicle membrane protein VAT-1 homolog	0.886	0.826	0.951	8.24E-04	3.45E-03
VEGFC	Vascular endothelial growth factor C	0.903	0.840	0.970	5.33E-03	1.87E-02
VNN1	Pantetheinase	1.139	1.065	1.217	1.44E-04	0.000692804
VPS28	Vacuolar protein sorting-associated protein 28 homolog	1.109	1.038	1.186	2.29E-03	0.008734047
VWA1	von Willebrand factor A domain-containing protein 1	1.093	1.032	1.159	0.002504138	9.46E-03
WAS	Wiskott-Aldrich syndrome protein	1.230	1.152	1.313	6.72E-10	6.19E-09
WASHC3	WASH complex subunit 3	1.114	1.037	1.198	3.35E-03	1.23E-02
WFIKKN1	WAP, Kazal, immunoglobulin, Kunitz and NTR domain-containing protein 1	1.142	1.081	1.206	2.13E-06	1.34E-05
YARS1	Tyrosine--tRNA ligase, cytoplasmic	1.350	1.281	1.423	7.34E-29	3.7E-27
YOD1	Ubiquitin thioesterase OTU1	0.913	0.851	0.980	1.12E-02	0.036331437
YY1	Transcriptional repressor protein YY1	1.132	1.067	1.201	0.0000422	2.21E-04
ZP3	Zona pellucida sperm-binding protein 3	1.175	1.060	1.302	0.002117979	8.10E-03

Models were adjusted for age, sex, ethnicity, Townsend deprivation index, smoking status, alcohol intake, physical activity, body mass index, household income, education status, hypertension, high density lipoprotein, triglyceride, glycosylated hemoglobin, glucose-lowering drugs, lipid-lowering drugs, coronary heart disease at baseline and stroke at baseline.

Supplementary Table 15 Mediation effects of plasma proteins associated with both PRISm and the risk of HF

	Protein's description used in the UK Biobank	Mediation effects of plasma proteins				
		Proportion	Lower	Upper	*P*	*FDR* *
ABL1	Tyrosine-protein kinase ABL1	2.35%	1.38%	3.81%	0	0
ACADM	Medium-chain specific acyl-CoA dehydrogenase, mitochondrial	4.76%	3.14%	6.94%	0	0
ACAN	Aggrecan core protein	6.80%	4.66%	9.67%	0	0
ACHE	Acetylcholinesterase	4.45%	2.89%	6.75%	0	0
ACP5	Tartrate-resistant acid phosphatase type 5	3.08%	1.94%	4.67%	0	0
ACRBP	Acrosin-binding protein	5.48%	3.32%	8.42%	0	0
ACRV1	Acrosomal protein SP-10	0.55%	0.14%	1.17%	0.002	0.002417112
ACY1	Aminoacylase-1	0.99%	0.39%	1.88%	0	0
ACY3	N-acyl-aromatic-L-amino acid amidohydrolase	2.02%	0.81%	3.75%	0.004	0.004581081
ADA	Adenosine deaminase	18.50%	14.37%	25.12%	0	0
ADAM12	Disintegrin and metalloproteinase domain-containing protein 12	-2.36%	-4.18%	-1.10%	0	0
ADAM23	Disintegrin and metalloproteinase domain-containing protein 23	4.78%	3.27%	7.03%	0	0
ADAMTS1	A disintegrin and metalloproteinase with thrombospondin motifs 1	0.63%	0.10%	1.48%	0.02	0.020861538
ADCYAP1R1	Pituitary adenylate cyclase-activating polypeptide type I receptor	1.39%	0.59%	2.47%	0	0
ADGRD1	Adhesion G-protein coupled receptor D1	0.67%	0.09%	1.56%	0.024	0.024691958
ADGRE2	Adhesion G protein-coupled receptor E2	2.51%	1.18%	4.11%	0	0
ADH1B	All-trans-retinol dehydrogenase	1.98%	1.03%	3.38%	0	0
ADIPOQ	Adiponectin	11.84%	8.64%	16.61%	0	0
ADM	Pro-adrenomedullin	1.63%	0.53%	3.01%	0.002	0.002417112
ADRA2A	Alpha-2A adrenergic receptor	2.29%	1.04%	3.70%	0	0
AFAP1	Actin filament-associated protein 1	2.89%	1.69%	4.48%	0	0
AFM	Afamin	4.49%	2.55%	6.87%	0	0
AGR2	Anterior gradient protein 2 homolog	0.80%	0.24%	1.72%	0.002	0.002417112
AGT	Angiotensinogen	0.86%	0.05%	1.92%	0.028	0.028633484
AHNAK	Neuroblast differentiation-associated protein AHNAK	0.93%	0.31%	1.90%	0.004	0.004581081
AIF1L	Allograft inflammatory factor 1-like	3.66%	2.31%	5.39%	0	0
ALCAM	CD166 antigen	5.35%	3.59%	7.75%	0	0
ALPI	Intestinal-type alkaline phosphatase	0.48%	0.05%	1.32%	0.008	0.008805195
AMIGO2	Amphoterin-induced protein 2	4.68%	3.06%	6.81%	0	0
AMY1A_AMY1B_AMY1C	Alpha-amylase 1A_Alpha-amylase 1B_Alpha-amylase 1C	1.83%	0.82%	3.17%	0	0
ANGPT1	Angiopoietin-1	1.22%	0.37%	2.43%	0.004	0.004581081
ANGPT2	Angiopoietin-2	1.38%	0.41%	2.69%	0.002	0.002417112
ANGPTL1	Angiopoietin-related protein 1	0.72%	0.09%	1.63%	0.022	0.022772519
ANKMY2	Ankyrin repeat and MYND domain-containing protein 2	0.48%	0.05%	1.20%	0.024	0.024691958
ANP32C	Acidic leucine-rich nuclear phosphoprotein 32 family member C	1.38%	0.52%	2.49%	0	0
ANXA11	Annexin A11	2.13%	1.07%	3.59%	0	0
ANXA3	Annexin A3	4.09%	2.72%	6.11%	0	0
ANXA5	Annexin A5	2.75%	1.56%	4.41%	0	0
AOC1	Amiloride-sensitive amine oxidase [copper-containing]	2.21%	0.94%	4.04%	0	0
AP2B1	AP-2 complex subunit beta	2.57%	1.23%	4.45%	0	0
APOC1	Apolipoprotein C-I	1.49%	0.56%	2.68%	0.002	0.002417112
APOE	Apolipoprotein E	0.64%	0.16%	1.48%	0.002	0.002417112
APOH	Beta-2-glycoprotein 1	2.23%	1.30%	3.58%	0	0
APOL1	Apolipoprotein L1	4.74%	3.09%	7.36%	0	0
APPL2	DCC-interacting protein 13-beta	1.78%	0.88%	3.07%	0	0
APRT	Adenine phosphoribosyltransferase	1.70%	0.73%	2.96%	0	0
ARHGAP45	Rho GTPase-activating protein 45	6.21%	4.26%	8.95%	0	0
ARHGEF10	Rho guanine nucleotide exchange factor 10	3.70%	2.29%	5.47%	0	0
ARHGEF12	Rho guanine nucleotide exchange factor 12	3.91%	2.44%	5.74%	0	0
ARHGEF5	Rho guanine nucleotide exchange factor 5	3.91%	2.42%	6.00%	0	0
ARID3A	AT-rich interactive domain-containing protein 3A	1.55%	0.68%	2.91%	0	0
ARID4B	AT-rich interactive domain-containing protein 4B	2.15%	1.21%	3.45%	0	0
ARL13B	ADP-ribosylation factor-like protein 13B	1.56%	0.71%	2.75%	0	0
ARMCX2	Armadillo repeat-containing X-linked protein 2	3.09%	1.79%	4.95%	0	0
ARNT	Aryl hydrocarbon receptor nuclear translocator	3.92%	2.59%	5.72%	0	0
ARNTL	Aryl hydrocarbon receptor nuclear translocator-like protein 1	1.69%	0.46%	3.18%	0.002	0.002417112
ARSA	Arylsulfatase A	1.14%	0.10%	2.41%	0.032	0.032527736
ART3	Ecto-ADP-ribosyltransferase 3	1.08%	0.38%	2.07%	0	0
ARTN	Artemin	0.84%	0.26%	1.85%	0.004	0.004581081
ASAH2	Neutral ceramidase	3.52%	2.09%	5.43%	0	0
ASGR1	Asialoglycoprotein receptor 1	0.96%	0.37%	1.84%	0	0
ASPN	Asporin	4.67%	2.99%	6.96%	0	0
ASPSCR1	Tether containing UBX domain for GLUT4	1.98%	0.96%	3.28%	0	0
ATF2	Cyclic AMP-dependent transcription factor ATF-2	5.60%	3.92%	7.96%	0	0
ATF4	Cyclic AMP-dependent transcription factor ATF-4	4.40%	2.73%	6.81%	0	0
ATG16L1	Autophagy-related protein 16-1	3.88%	2.49%	5.87%	0	0
ATP1B2	Sodium/potassium-transporting ATPase subunit beta-2	2.06%	1.12%	3.42%	0	0
ATP1B3	Sodium/potassium-transporting ATPase subunit beta-3	2.10%	1.03%	3.67%	0	0
ATP1B4	Protein ATP1B4	3.05%	1.63%	4.86%	0	0
ATRAID	All-trans retinoic acid-induced differentiation factor	4.95%	3.20%	7.50%	0	0
ATRN	Attractin, Isoform 2	3.55%	2.16%	5.47%	0	0
ATXN10	Ataxin-10	1.75%	0.63%	3.26%	0.002	0.002417112
ATXN2	Ataxin-2	1.60%	0.74%	2.99%	0	0
ATXN3	Ataxin-3	3.65%	2.22%	5.49%	0	0
AXIN1	Axin-1	10.32%	7.62%	14.41%	0	0
AXL	Tyrosine-protein kinase receptor UFO	2.52%	1.39%	4.06%	0	0
AZU1	Azurocidin	5.54%	3.84%	8.15%	0	0
B3GNT7	UDP-GlcNAc:betaGal beta-1,3-N-acetylglucosaminyltransferase 7	1.37%	0.28%	2.77%	0.01	0.010848
B4GALT1	Beta-1,4-galactosyltransferase 1	2.82%	1.78%	4.48%	0	0
B4GAT1	Beta-1,4-glucuronyltransferase 1	1.32%	0.59%	2.37%	0	0
BACH1	Transcription regulator protein BACH1	1.25%	0.52%	2.33%	0	0
BAG3	BAG family molecular chaperone regulator 3	0.95%	0.16%	1.97%	0.01	0.010848
BAG4	BAG family molecular chaperone regulator 4	1.31%	0.51%	2.37%	0.002	0.002417112
BAMBI	BMP and activin membrane-bound inhibitor homolog	5.27%	3.63%	7.67%	0	0
BCAM	Basal cell adhesion molecule	6.95%	4.85%	9.90%	0	0
BCAN	Brevican core protein	4.89%	3.15%	7.31%	0	0
BCAT1	Branched-chain-amino-acid aminotransferase, cytosolic	3.61%	2.22%	5.41%	0	0
BCL2	Apoptosis regulator Bcl-2	1.99%	0.85%	3.35%	0	0
BCL2L1	Bcl-2-like protein 1	2.26%	1.20%	3.89%	0	0
BCL2L11	Bcl-2-like protein 11, Isoform BimL	4.09%	2.56%	6.22%	0	0
BCL7B	B-cell CLL/lymphoma 7 protein family member B	4.11%	2.56%	6.54%	0	0
BCR	Breakpoint cluster region protein	0.94%	0.31%	1.91%	0	0
BDNF	Brain-derived neurotrophic factor	0.53%	0.11%	1.27%	0.006	0.006690789
BEX3	Protein BEX3	4.99%	3.23%	7.57%	0	0
BID	BH3-interacting domain death agonist	2.16%	0.50%	4.17%	0.014	0.01485446
BIN2	Bridging integrator 2	-1.06%	-2.04%	-0.36%	0	0
BLOC1S3	Biogenesis of lysosome-related organelles complex 1 subunit 3	1.46%	0.60%	2.50%	0	0
BMPER	BMP-binding endothelial regulator protein	2.10%	1.05%	3.62%	0	0
BNIP2	BCL2/adenovirus E1B 19 kDa protein-interacting protein 2	0.65%	0.14%	1.59%	0.008	0.008805195
BRK1	Protein BRICK1	2.06%	1.08%	3.52%	0	0
BSND	Barttin	5.35%	3.63%	7.86%	0	0
BTD	Biotinidase	5.68%	3.96%	8.18%	0	0
BTN2A1	Butyrophilin subfamily 2 member A1	5.52%	3.77%	8.01%	0	0
BTNL10	Butyrophilin-like protein 10	0.89%	0.28%	1.74%	0	0
C19orf12	Protein C19orf12	0.91%	0.26%	1.97%	0.004	0.004581081
C1GALT1C1	C1GALT1-specific chaperone 1	1.71%	0.79%	3.10%	0	0
C1QA	Complement C1q subcomponent subunit A	2.56%	1.18%	4.28%	0	0
C1QBP	Complement component 1 Q subcomponent-binding protein, mitochondrial	3.21%	1.76%	4.92%	0	0
C1QTNF6	Complement C1q tumor necrosis factor-related protein 6	2.95%	1.65%	4.92%	0	0
C1R	Complement C1r subcomponent	2.84%	1.58%	4.60%	0	0
C1RL	Complement C1r subcomponent-like protein	2.98%	1.71%	4.58%	0	0
C1S	Complement C1s subcomponent	1.29%	0.40%	2.53%	0.002	0.002417112
C2CD2L	Phospholipid transfer protein C2CD2L	6.31%	4.46%	8.97%	0	0
C7	Complement component C7	4.22%	2.72%	6.24%	0	0
CA5A	Carbonic anhydrase 5A, mitochondrial	9.36%	6.88%	13.10%	0	0
CA7	Carbonic anhydrase 7	2.48%	1.19%	4.22%	0	0
CA8	Carbonic anhydrase-related protein	11.65%	8.74%	15.78%	0	0
CABP2	Calcium-binding protein 2	8.21%	5.91%	11.35%	0	0
CALCB	Calcitonin gene-related peptide 2	2.19%	0.80%	3.99%	0.002	0.002417112
CAPG	Macrophage-capping protein	1.59%	0.71%	2.85%	0	0
CAPS	Calcyphosin	2.55%	1.28%	4.36%	0	0
CASC3	Protein CASC3	5.70%	3.88%	8.27%	0	0
CASP1	Caspase-1	1.40%	0.62%	2.62%	0.002	0.002417112
CASP10	Caspase-10	0.64%	0.08%	1.44%	0.03	0.030586466
CASP3	Caspase-3	2.74%	1.41%	4.70%	0	0
CASQ2	Calsequestrin-2	8.83%	6.50%	12.07%	0	0
CAT	Catalase	0.92%	0.13%	1.97%	0.022	0.022772519

CBLN1	Cerebellin-1	8.30%	5.94%	11.38%	0	0
CBLN4	Cerebellin-4	0.93%	0.24%	1.99%	0.004	0.004581081
CBX2	Chromobox protein homolog 2	1.07%	0.41%	2.09%	0	0
CC2D1A	Coiled-coil and C2 domain-containing protein 1A	3.18%	1.93%	5.07%	0	0
CCER2	Coiled-coil domain-containing glutamate-rich protein 2	2.26%	0.96%	4.00%	0	0
CCL13	C-C motif chemokine 13	0.54%	0.07%	1.36%	0.012	0.012873418
CCL14	C-C motif chemokine 14	4.37%	2.79%	6.47%	0	0
CCL15	C-C motif chemokine 15	1.51%	0.66%	2.76%	0	0
CCL16	C-C motif chemokine 16	3.71%	2.03%	6.33%	0	0
CCL18	C-C motif chemokine 18	2.51%	1.44%	4.14%	0	0
CCL2	C-C motif chemokine 2	2.53%	1.42%	4.13%	0	0
CCL21	C-C motif chemokine 21	4.10%	2.77%	5.99%	0	0
CCL22	C-C motif chemokine 22	2.63%	1.51%	4.13%	0	0
CCL24	C-C motif chemokine 24	4.17%	2.69%	6.04%	0	0
CCL26	C-C motif chemokine 26	5.67%	3.79%	8.33%	0	0
CCL27	C-C motif chemokine 27	2.25%	1.21%	3.70%	0	0
CCL7	C-C motif chemokine 7	4.51%	2.90%	6.61%	0	0
CD109	CD109 antigen	2.32%	1.23%	3.79%	0	0
CD160	CD160 antigen	0.95%	0.27%	1.90%	0.002	0.002417112
CD164	Sialomucin core protein 24	1.48%	0.67%	2.67%	0	0
CD1C	T-cell surface glycoprotein CD1c	3.35%	1.88%	5.19%	0	0
CD2	T-cell surface antigen CD2	1.96%	0.98%	3.52%	0	0
CD226	CD226 antigen	1.34%	0.51%	2.44%	0	0
CD27	CD27 antigen	3.62%	2.19%	5.68%	0	0
CD2AP	CD2-associated protein	1.57%	0.68%	2.86%	0	0
CD34	Hematopoietic progenitor cell antigen CD34	0.55%	0.07%	1.30%	0.03	0.030586466
CD38	ADP-ribosyl cyclase/cyclic ADP-ribose hydrolase 1	5.69%	3.72%	8.45%	0	0
CD3D	T-cell surface glycoprotein CD3 delta chain	2.37%	1.31%	3.81%	0	0
CD3E	T-cell surface glycoprotein CD3 epsilon chain	2.44%	1.31%	4.10%	0	0
CD4	T-cell surface glycoprotein CD4	3.98%	2.51%	6.02%	0	0
CD40	Tumor necrosis factor receptor superfamily member 5	-0.71%	-1.57%	-0.18%	0	0
CD48	CD48 antigen	2.19%	1.05%	3.75%	0	0
CD59	CD59 glycoprotein	1.73%	0.75%	3.07%	0	0
CD6	T-cell differentiation antigen CD6	8.19%	5.78%	11.64%	0	0
CD7	T-cell antigen CD7	7.36%	5.36%	10.34%	0	0
CD70	CD70 antigen	5.87%	3.95%	8.41%	0	0
CD72	B-cell differentiation antigen CD72	7.09%	4.97%	9.99%	0	0
CD79B	B-cell antigen receptor complex-associated protein beta chain	2.80%	1.60%	4.35%	0	0
CD80	T-lymphocyte activation antigen CD80	1.64%	0.68%	2.95%	0.002	0.002417112
CD82	CD82 antigen	1.01%	0.38%	1.89%	0	0
CD99	CD99 antigen	2.90%	1.70%	4.58%	0	0
CD99L2	CD99 antigen-like protein 2	0.75%	0.19%	1.65%	0.004	0.004581081
CDC123	Cell division cycle protein 123 homolog	2.54%	1.39%	4.04%	0	0
CDC25A	M-phase inducer phosphatase 1	1.23%	0.54%	2.29%	0	0
CDC26	Anaphase-promoting complex subunit CDC26	1.59%	0.47%	3.07%	0.006	0.006690789
CDH1	Cadherin-1	0.88%	0.26%	1.75%	0	0
CDH17	Cadherin-17	2.80%	1.57%	4.42%	0	0
CDH2	Cadherin-2	1.84%	0.65%	3.40%	0	0
CDH3	Cadherin-3	3.52%	2.14%	5.51%	0	0
CDH4	Cadherin-4	1.64%	0.80%	2.86%	0	0
CEACAM1	Carcinoembryonic antigen-related cell adhesion molecule 1	2.00%	0.69%	3.64%	0.006	0.006690789
CEACAM16	Carcinoembryonic antigen-related cell adhesion molecule 16	3.74%	2.22%	5.76%	0	0
CEACAM18	Carcinoembryonic antigen-related cell adhesion molecule 18	0.88%	0.18%	1.88%	0.008	0.008805195
CEACAM19	Carcinoembryonic antigen-related cell adhesion molecule 19	9.45%	6.74%	12.97%	0	0
CEACAM20	Carcinoembryonic antigen-related cell adhesion molecule 20	0.78%	0.23%	1.71%	0.004	0.004581081
CEACAM8	Carcinoembryonic antigen-related cell adhesion molecule 8	4.21%	2.66%	6.33%	0	0
CELSR2	Cadherin EGF LAG seven-pass G-type receptor 2	1.49%	0.50%	2.77%	0.006	0.006690789
CEMIP2	Cell surface hyaluronidase	2.34%	1.29%	3.85%	0	0
CEND1	Cell cycle exit and neuronal differentiation protein 1	1.58%	0.44%	3.00%	0.004	0.004581081
CEP20	Centrosomal protein 20	1.42%	0.30%	2.87%	0.014	0.01485446
CEP350	Centrosome-associated protein 350	1.43%	0.61%	2.55%	0	0
CERT	Ceramide transfer protein	0.79%	0.18%	1.71%	0.004	0.004581081
CES3	Carboxylesterase 3	2.12%	0.59%	3.87%	0.006	0.006690789
CETN2	Centrin-2	5.94%	4.14%	8.07%	0	0
CFC1	Cryptic protein	2.80%	1.62%	4.45%	0	0
CGB3 CGB5 CGB8	Choriogonadotropin subunit beta 3	3.29%	1.81%	5.17%	0	0
CGN	Cingulin	1.00%	0.28%	1.89%	0.002	0.002417112
CHAC2	Glutathione-specific gamma-glutamylcyclotransferase 2	1.34%	0.46%	2.62%	0	0
CHEK2	Serine/threonine-protein kinase Chk2	-2.92%	-4.38%	-1.70%	0	0
CHGA	Chromogranin-A	2.97%	1.78%	4.85%	0	0
CHMP6	Charged multivesicular body protein 6	12.16%	8.79%	16.74%	0	0
CHRDL1	Chordin-like protein 1	4.04%	1.95%	6.65%	0	0
CHRDL2	Chordin-like protein 2	0.46%	0.03%	1.14%	0.026	0.026668684
CHRM1	Muscarinic acetylcholine receptor M1	1.53%	0.72%	2.81%	0	0
CIT	Citron Rho-interacting kinase	0.63%	0.06%	1.41%	0.026	0.026668684
CLC	Galectin-10	2.34%	1.26%	4.09%	0	0
CLEC12A	C-type lectin domain family 12 member A	1.08%	0.38%	2.20%	0	0
CLEC14A	C-type lectin domain family 14 member A	1.67%	0.80%	2.94%	0	0
CLEC1A	C-type lectin domain family 1 member A	11.13%	8.53%	14.83%	0	0
CLEC2L	C-type lectin domain family 2 member L	2.11%	1.06%	3.45%	0	0
CLEC4D	C-type lectin domain family 4 member D	9.44%	6.79%	12.73%	0	0
CLEC7A	C-type lectin domain family 7 member A	2.75%	1.46%	4.68%	0	0
CLGN	Calmegin	1.75%	0.63%	3.28%	0	0
CLIC5	Chloride intracellular channel protein 5	-0.45%	-1.23%	-0.04%	0.04	0.040477612
CLU	Clusterin	0.56%	0.10%	1.35%	0.018	0.018862442
CLUL1	Clusterin-like protein 1	4.22%	2.56%	6.47%	0	0
CNST	Consortin	1.35%	0.56%	2.39%	0	0
CNTF	Ciliary neurotrophic factor	1.64%	0.74%	2.86%	0	0
CNTN1	Contactin-1	4.55%	2.98%	6.71%	0	0
CNTN4	Contactin-4	3.36%	2.01%	5.36%	0	0
CNTN5	Contactin-5	2.05%	0.99%	3.47%	0	0
COCH	Cochlin	-1.19%	-2.31%	-0.43%	0	0
COL6A3	Collagen alpha-3(VI) chain	6.94%	4.95%	9.89%	0	0
COL9A1	Collagen alpha-1(IX) chain	5.15%	3.38%	7.39%	0	0
COPB2	Coatomer subunit beta'	7.94%	5.29%	11.20%	0	0
CORO1A	Coronin-1A	0.56%	0.11%	1.43%	0.014	0.01485446
CORO6	Coronin-6	3.74%	2.26%	5.81%	0	0
CPA1	Carboxypeptidase A1	0.99%	0.34%	1.95%	0	0
CPA2	Carboxypeptidase A2	2.94%	1.69%	4.59%	0	0
CPB2	Carboxypeptidase B2	0.79%	0.06%	1.75%	0.034	0.034508982
CPLX2	Complexin-2	0.98%	0.39%	1.89%	0	0
CPTP	Ceramide-1-phosphate transfer protein	2.93%	1.61%	4.70%	0	0
CPVL	Probable serine carboxypeptidase CPVL	9.08%	6.67%	12.18%	0	0
CRACR2A	EF-hand calcium-binding domain-containing protein 4B	4.22%	2.64%	6.36%	0	0
CREB3	Cyclic AMP-responsive element-binding protein 3	1.68%	0.75%	2.85%	0	0
CRHR1	Corticotropin-releasing factor receptor 1	1.62%	0.64%	3.08%	0	0
CRISP3	Cysteine-rich secretory protein 3	1.49%	0.70%	2.67%	0	0
CRLF1	Cytokine receptor-like factor 1	1.82%	0.84%	3.15%	0	0
CRNN	Cornulin	-3.13%	-5.19%	-1.83%	0	0
CRTAC1	Cartilage acidic protein 1	-2.10%	-3.93%	-0.61%	0.006	0.006690789
CRTAP	Cartilage-associated protein	10.62%	7.94%	14.45%	0	0
CRX	Cone-rod homeobox protein	1.70%	0.84%	2.91%	0	0
CRYBB1	Beta-crystallin B1	9.62%	7.25%	12.99%	0	0
CRYBB2	Beta-crystallin B2	6.64%	4.80%	9.40%	0	0
CRYGD	Gamma-crystallin D	5.01%	3.08%	7.55%	0	0
CRYM	Ketimine reductase mu-crystallin	2.20%	1.24%	3.57%	0	0
CSF1	Macrophage colony-stimulating factor 1	1.38%	0.60%	2.48%	0	0
CSF2	Granulocyte-macrophage colony-stimulating factor	2.56%	1.52%	4.14%	0	0
CSF2RB	Cytokine receptor common subunit beta	2.46%	1.34%	4.11%	0	0
CSF3	Granulocyte colony-stimulating factor	2.74%	1.45%	4.34%	0	0
CSF3R	Granulocyte colony-stimulating factor receptor	2.01%	0.99%	3.42%	0	0
CSNK2A1	Casein kinase II subunit alpha	2.32%	1.28%	3.92%	0	0
CSPG4	Chondroitin sulfate proteoglycan 4	2.63%	1.37%	4.55%	0	0
CSPG5	Chondroitin sulfate proteoglycan 5	1.63%	0.76%	2.85%	0	0
CSRP3	Cysteine and glycine-rich protein 3	0.77%	0.22%	1.59%	0.004	0.004581081
CST1	Cystatin-SN	1.80%	0.91%	3.04%	0	0
CST3	Cystatin-C	0.63%	0.10%	1.43%	0.022	0.022772519
CST7	Cystatin-F	-1.40%	-2.67%	-0.49%	0	0
CSTB	Cystatin-B	1.95%	0.96%	3.42%	0	0
CTAG1A CTAG1B	Cancer/testis antigen 1	3.08%	1.57%	4.99%	0	0
CTBS	Di-N-acetylchitobiase	3.24%	1.78%	5.27%	0	0

CTHRC1	Collagen triple helix repeat-containing protein 1	4.01%	2.65%	6.22%	0	0
CTSB	Cathepsin B	2.76%	1.60%	4.50%	0	0
CTSD	Cathepsin D	3.40%	1.74%	5.34%	0	0
CTSS	Cathepsin S	6.27%	4.44%	9.30%	0	0
CXCL1	Growth-regulated alpha protein	2.83%	1.51%	4.49%	0	0
CXCL12	Stromal cell-derived factor 1	6.98%	4.82%	10.02%	0	0
CXCL14	C-X-C motif chemokine 14	1.63%	0.77%	2.85%	0	0
DAG1	Dystroglycan	1.74%	0.66%	3.16%	0	0
DAND5	DAN domain family member 5	1.47%	0.67%	2.65%	0	0
DAPK2	Death-associated protein kinase 2	4.92%	3.08%	7.45%	0	0
DBH	Dopamine beta-hydroxylase	1.09%	0.43%	2.14%	0.002	0.002417112
DCTN2	Dynactin subunit 2	1.60%	0.26%	2.97%	0.014	0.01485446
DCTPP1	dCTP pyrophosphatase 1	0.70%	0.12%	1.64%	0.024	0.024691958
DDR1	Epithelial discoidin domain-containing receptor 1	1.26%	0.53%	2.37%	0	0
DDT	D-dopachrome decarboxylase	0.88%	0.05%	1.92%	0.036	0.036484305
DECR1	2,4-dienoyl-CoA reductase, mitochondrial	1.46%	0.36%	2.92%	0.018	0.018862442
DEFB104A DEFB104B	Beta-defensin 104	1.76%	0.83%	3.04%	0	0
DEFB118	Beta-defensin 118	1.19%	0.45%	2.33%	0.004	0.004581081
DENND2B	DENN domain-containing protein 2B	1.58%	0.45%	2.98%	0.002	0.002417112
DENR	Density-regulated protein	2.07%	0.99%	3.66%	0	0
DHPS	Deoxyhypusine synthase	1.99%	0.93%	3.40%	0	0
DHRS4L2	Dehydrogenase/reductase SDR family member 4-like 2	7.26%	5.39%	10.15%	0	0
DIABLO	Diablo homolog, mitochondrial	1.46%	0.66%	2.82%	0	0
DIPK1C	Divergent protein kinase domain 1C	4.08%	2.62%	6.03%	0	0
DLG4	Disks large homolog 4	2.42%	0.93%	4.46%	0.004	0.004581081
DLGAP5	Disks large-associated protein 5	2.68%	1.13%	4.42%	0.002	0.002417112
DLK1	Protein delta homolog 1	0.88%	0.28%	1.82%	0	0
DLL1	Delta-like protein 1	2.36%	1.03%	3.90%	0	0
DMP1	Dentin matrix acidic phosphoprotein 1	6.49%	3.27%	10.79%	0	0
DNAJA2	DnaJ homolog subfamily A member 2	3.52%	2.10%	5.52%	0	0
DNAJB1	DnaJ homolog subfamily B member 1	2.19%	1.20%	3.64%	0	0
DNAJB6	DnaJ homolog subfamily B member 6	9.48%	6.90%	12.96%	0	0
DNAJB8	DnaJ homolog subfamily B member 8	1.67%	0.62%	3.03%	0.002	0.002417112
DNLZ	DNL-type zinc finger protein	1.55%	0.61%	2.75%	0	0
DNM1	Dynamin-1	7.31%	5.07%	10.46%	0	0
DNM3	Dynamin-3	1.03%	0.17%	2.27%	0.01	0.010848
DNMBP	Dynamin-binding protein	2.01%	0.90%	3.39%	0	0
DNPEP	Aspartyl aminopeptidase	7.52%	5.29%	10.70%	0	0
DOK2	Docking protein 2	2.58%	1.36%	4.05%	0	0
DPEP2	Dipeptidase 2	3.82%	2.32%	5.91%	0	0
DPP4	Dipeptidyl peptidase 4	1.55%	0.74%	2.75%	0	0
DPP6	Dipeptidyl aminopeptidase-like protein 6	2.22%	1.19%	3.68%	0	0
DPY30	Protein dpy-30 homolog	0.75%	0.19%	1.62%	0.004	0.004581081
DRG2	Developmentally-regulated GTP-binding protein 2	8.35%	5.87%	11.82%	0	0
DSC2	Desmocollin-2	2.94%	1.69%	4.64%	0	0
DSCAM	Down syndrome cell adhesion molecule	5.10%	3.36%	7.43%	0	0
DSG4	Desmoglein-4	3.06%	1.78%	4.70%	0	0
DTNB	Dystrobrevin beta	1.45%	0.60%	2.64%	0	0
DTX2	Probable E3 ubiquitin-protein ligase DTX2	3.34%	2.07%	5.24%	0	0
DUSP3	Dual specificity protein phosphatase 3	2.89%	1.71%	4.49%	0	0
ECHS1	Enoyl-CoA hydratase, mitochondrial	2.65%	1.26%	4.47%	0	0
ECM1	Extracellular matrix protein 1	7.46%	5.05%	10.38%	0	0
EDEM2	ER degradation-enhancing alpha-mannosidase-like protein 2	0.78%	0.24%	1.63%	0	0
EDF1	Endothelial differentiation-related factor 1	0.98%	0.30%	1.98%	0.002	0.002417112
EDN1	Endothelin-1	1.69%	0.81%	2.92%	0	0
EGFR	Epidermal growth factor receptor	4.30%	2.70%	6.38%	0	0
EIF2AK2	Interferon-induced, double-stranded RNA-activated protein kinase	0.62%	0.09%	1.49%	0.014	0.01485446
EIF2S2	Eukaryotic translation initiation factor 2 subunit 2	1.97%	0.96%	3.45%	0	0
EIF4E	Eukaryotic translation initiation factor 4E	0.34%	0.01%	0.94%	0.048	0.048428571
EIF4EBP1	Eukaryotic translation initiation factor 4E-binding protein 1	0.99%	0.20%	2.10%	0.018	0.018862442
EIF4G3	Eukaryotic translation initiation factor 4 gamma 3	1.86%	0.84%	3.25%	0	0
EIF5	Eukaryotic translation initiation factor 5	6.47%	4.39%	9.22%	0	0
ELAVL4	ELAV-like protein 4	5.76%	3.87%	8.63%	0	0
ELN	Elastin	0.94%	0.21%	1.98%	0.012	0.012873418
ELOA	Elongin-A	0.89%	0.25%	1.89%	0.004	0.004581081
ENO2	Gamma-enolase	1.92%	1.02%	3.20%	0	0
ENPEP	Glutamyl aminopeptidase	7.46%	5.32%	10.84%	0	0
ENTR1	Endosome-associated-trafficking regulator 1	8.23%	5.84%	11.36%	0	0
EPHA1	Ephrin type-A receptor 1	3.34%	1.89%	5.31%	0	0
EPO	Erythropoietin	1.96%	0.90%	3.35%	0	0
ERBB3	Receptor tyrosine-protein kinase erbB-3	4.75%	3.17%	6.85%	0	0
ERBB4	Receptor tyrosine-protein kinase erbB-4	2.28%	1.19%	3.88%	0	0
EREG	Proepiregulin	3.38%	1.99%	5.20%	0	0
ERP44	Endoplasmic reticulum resident protein 44	1.91%	0.82%	3.31%	0.002	0.002417112
ESM1	Endothelial cell-specific molecule 1	0.65%	0.10%	1.46%	0.02	0.020861538
ESR1	Estrogen receptor	1.68%	0.27%	3.40%	0.016	0.016923557
EVI2B	Protein EVI2B	3.54%	2.29%	5.30%	0	0
EVPL	Envoplakin	4.62%	2.99%	7.03%	0	0
EXOSC10	Exosome component 10	4.71%	3.12%	6.80%	0	0
EXTL1	Exostosin-like 1	10.77%	7.54%	15.08%	0	0
EZR	Ezrin	3.10%	1.57%	5.30%	0	0
F13B	Coagulation factor XIII B chain	1.48%	0.63%	2.68%	0	0
F2	Prothrombin	1.72%	0.88%	3.03%	0	0
F2R	Proteinase-activated receptor 1	3.14%	1.88%	4.81%	0	0
FABP1	Fatty acid-binding protein, liver	1.32%	0.53%	2.55%	0	0
FABP4	Fatty acid-binding protein, adipocyte	14.45%	10.30%	19.85%	0	0
FADD	FAS-associated death domain protein	2.97%	1.74%	4.61%	0	0
FAM3D	Protein FAM3D	5.61%	3.83%	8.09%	0	0
FBLN2	Fibulin-2	2.52%	1.41%	4.13%	0	0
FBP1	Fructose-1,6-bisphosphatase 1	3.25%	1.86%	5.27%	0	0
FCAMR	High affinity immunoglobulin alpha and immunoglobulin mu Fc receptor	1.96%	0.62%	3.55%	0.006	0.006690789
FCAR	Immunoglobulin alpha Fc receptor	1.30%	0.39%	2.52%	0.004	0.004581081
FCN1	Ficolin-1	3.77%	2.32%	6.00%	0	0
FDX2	Ferredoxin-2, mitochondrial	2.25%	1.28%	3.67%	0	0
FEN1	Flap endonuclease 1	0.72%	0.19%	1.60%	0	0
FETUB	Fetuin-B	0.49%	0.05%	1.26%	0.016	0.016923557
FGF16	Fibroblast growth factor 16	1.30%	0.55%	2.47%	0.002	0.002417112
FGF7	Fibroblast growth factor 7	8.00%	5.76%	10.91%	0	0
FGF9	Fibroblast growth factor 9	1.78%	0.69%	3.35%	0.002	0.002417112
FGFBP1	Fibroblast growth factor-binding protein 1	1.56%	0.58%	2.86%	0.002	0.002417112
FGFBP2	Fibroblast growth factor-binding protein 2	4.58%	3.03%	6.60%	0	0
FGFBP3	Fibroblast growth factor-binding protein 3	4.25%	2.66%	6.37%	0	0
FGFR4	Fibroblast growth factor receptor 4	3.28%	1.83%	5.10%	0	0
FGL1	Fibrinogen-like protein 1	3.93%	2.36%	6.16%	0	0
FGR	Tyrosine-protein kinase Fgr	2.44%	1.25%	4.00%	0	0
FHIT	Bis(5'-adenosyl)-triphosphatase	2.47%	1.22%	4.09%	0	0
FKBP14	Peptidyl-prolyl cis-trans isomerase FKBP14	4.16%	2.75%	6.11%	0	0
FKBP5	Peptidyl-prolyl cis-trans isomerase FKBP5	2.50%	0.74%	4.42%	0.004	0.004581081
FKBP7	Peptidyl-prolyl cis-trans isomerase FKBP7	1.37%	0.46%	2.52%	0	0
FKBPL	FK506-binding protein-like	9.64%	7.06%	13.10%	0	0
FLI1	Friend leukemia integration 1 transcription factor	1.70%	0.80%	3.06%	0	0
FMNL1	Formin-like protein 1	3.28%	1.99%	5.02%	0	0
FOLR3	Folate receptor gamma	1.89%	0.97%	3.26%	0	0
FOSB	Protein fosB	1.76%	0.69%	3.24%	0.002	0.002417112
FST	Follistatin	0.92%	0.16%	1.89%	0.014	0.01485446
FUT1	Galactoside alpha-(1,2)-fucosyltransferase 1	0.53%	0.08%	1.26%	0.01	0.010848
FZD10	Frizzled-10	1.21%	0.45%	2.26%	0	0
GABARAPL1	Gamma-aminobutyric acid receptor-associated protein-like 1	1.79%	0.92%	3.05%	0	0
GAD1	Glutamate decarboxylase 1	2.25%	1.21%	3.80%	0	0
GADD45B	Growth arrest and DNA damage-inducible protein GADD45 beta	0.98%	0.33%	1.94%	0	0
GADD45GIP1	Growth arrest and DNA damage-inducible proteins-interacting protein 1	2.28%	1.28%	3.69%	0	0
GAGE2A	G antigen 2A	0.58%	0.01%	1.44%	0.044	0.044459016
GAMT	Guanidinoacetate N-methyltransferase	3.10%	1.35%	5.45%	0	0
GAS2	Growth arrest-specific protein 2	5.62%	3.86%	7.90%	0	0
GASK1A	Golgi-associated kinase 1A	1.49%	0.52%	2.70%	0	0
GAST	Gastrin	2.23%	1.25%	3.86%	0	0
GBA	Lysosomal acid glucosylceramidase	0.73%	0.21%	1.65%	0	0
GBP2	Guanylate-binding protein 2	1.10%	0.28%	2.18%	0.006	0.006690789
GCNT1	Beta-1,3-galactosyl-O-glycosyl-glycoprotein beta-1,6-N-acetylglucosaminyltransferase	2.98%	1.69%	4.66%	0	0
GFRA2	GDNF family receptor alpha-2	5.73%	3.86%	8.37%	0	0
GGACT	Gamma-glutamylaminecyclotransferase	1.80%	0.85%	3.13%	0	0

GGCT	Gamma-glutamylcyclotransferase	4.92%	3.26%	7.31%	0	0
GGT1	Glutathione hydrolase 1 proenzyme	2.13%	1.02%	3.42%	0	0
GGT5	Glutathione hydrolase 5 proenzyme	2.13%	1.07%	3.69%	0	0
GH1	Somatotropin	2.73%	1.58%	4.35%	0	0
GH2	Growth hormone variant	1.18%	0.37%	2.32%	0.004	0.004581081
GHR	Growth hormone receptor	8.41%	6.14%	11.76%	0	0
GHRHR	Growth hormone-releasing hormone receptor	2.51%	1.39%	4.03%	0	0
GHRL	Appetite-regulating hormone	-1.38%	-2.64%	-0.58%	0	0
GID8	Glucose-induced degradation protein 8 homolog	2.92%	1.61%	4.63%	0	0
GIPR	Gastric inhibitory polypeptide receptor	2.55%	1.34%	4.11%	0	0
GJA8	Gap junction alpha-8 protein	1.43%	0.60%	2.56%	0	0
GKN1	Gastrokine-1	1.05%	0.36%	2.10%	0	0
GLI2	Zinc finger protein GLI2	1.29%	0.60%	2.31%	0	0
GLO1	Lactoylglutathione lyase	2.20%	0.95%	3.72%	0	0
GLOD4	Glyoxalase domain-containing protein 4	3.71%	2.22%	5.51%	0	0
GLYR1	Putative oxidoreductase GLYR1	0.75%	0.21%	1.54%	0.002	0.002417112
GMPR2	GMP reductase 2	0.79%	0.23%	1.68%	0.002	0.002417112
GNGT1	Guanine nucleotide-binding protein G(T) subunit gamma-T1	2.29%	1.23%	3.67%	0	0
GNPDA1	Glucosamine-6-phosphate isomerase 1	7.28%	5.19%	10.13%	0	0
GNPDA2	Glucosamine-6-phosphate isomerase 2	1.22%	0.49%	2.34%	0	0
GOLM2	Protein GOLM2	1.37%	0.59%	2.59%	0	0
GORASP2	Golgi reassembly-stacking protein 2	-3.84%	-6.08%	-2.05%	0	0
GOT1	Aspartate aminotransferase, cytoplasmic	-1.65%	-2.96%	-0.65%	0	0
GP6	Platelet glycoprotein VI	3.45%	2.09%	5.24%	0	0
GPHA2	Glycoprotein hormone alpha-2	4.82%	3.14%	7.15%	0	0
GPKOW	G-patch domain and KOW motifs-containing protein	1.64%	0.67%	2.85%	0	0
GPR101	Probable G-protein coupled receptor 101	1.86%	0.88%	3.20%	0	0
GPR15L	Protein GPR15L	1.61%	0.72%	2.89%	0	0
GRHPR	Glyoxylate reductase/hydroxypyruvate reductase	1.24%	0.53%	2.25%	0	0
GRN	Progranulin	2.49%	1.29%	4.08%	0	0
GRPEL1	GrpE protein homolog 1, mitochondrial	1.91%	0.98%	3.17%	0	0
GSAP	Gamma-secretase-activating protein	4.36%	2.82%	6.51%	0	0
GSR	Glutathione reductase, mitochondrial	0.83%	0.22%	1.79%	0.006	0.006690789
GSTA1	Glutathione S-transferase A1	1.26%	0.42%	2.35%	0.002	0.002417112
GSTT2B	Glutathione S-transferase theta-2B	1.38%	0.55%	2.58%	0.004	0.004581081
GTF2IRD1	General transcription factor II-I repeat domain-containing protein 1	5.94%	3.94%	8.38%	0	0
GTPBP2	GTP-binding protein 2	4.06%	2.64%	5.88%	0	0
GUK1	Guanylate kinase	0.65%	0.15%	1.51%	0.012	0.012873418
GUSB	Beta-glucuronidase	2.64%	1.49%	4.31%	0	0
GZMB	Granzyme B	1.66%	0.35%	3.24%	0.01	0.010848
HARS1	Histidine--tRNA ligase, cytoplasmic	2.14%	1.06%	3.58%	0	0
HAVCR2	Hepatitis A virus cellular receptor 2	1.51%	0.65%	2.79%	0	0
HBEGF	Proheparin-binding EGF-like growth factor	3.37%	2.03%	5.19%	0	0
HCG22	Protein PBMUCL2	-1.11%	-2.16%	-0.33%	0	0
HCLS1	Hematopoietic lineage cell-specific protein	0.92%	0.21%	1.89%	0.018	0.018862442
HEG1	Protein HEG homolog 1	3.06%	1.79%	4.80%	0	0
HEPACAM2	HEPACAM family member 2	5.65%	3.94%	8.22%	0	0
HEPH	Hephaestin	0.50%	0.05%	1.23%	0.012	0.012873418
HGS	Hepatocyte growth factor-regulated tyrosine kinase substrate	2.89%	1.60%	4.65%	0	0
HIP1R	Huntingtin-interacting protein 1-related protein	4.12%	2.66%	6.08%	0	0
HK2	Hexokinase-2	1.30%	0.47%	2.49%	0	0
HMBS	Porphobilinogen deaminase	4.68%	3.21%	6.82%	0	0
HMCN2	Hemicentin-2	1.61%	0.71%	2.92%	0	0
HMGCS1	Hydroxymethylglutaryl-CoA synthase, cytoplasmic	1.29%	0.40%	2.54%	0.012	0.012873418
HMMR	Hyaluronan mediated motility receptor	2.32%	1.29%	3.73%	0	0
HMOX1	Heme oxygenase 1	2.09%	1.01%	3.50%	0	0
HNF1A	Hepatocyte nuclear factor 1-alpha	5.79%	4.13%	8.47%	0	0
HNMT	Histamine N-methyltransferase	10.17%	7.29%	13.88%	0	0
HNRNPK	Heterogeneous nuclear ribonucleoprotein K	2.00%	0.74%	3.62%	0	0
HS3ST3B1	Heparan sulfate glucosamine 3-O-sulfotransferase 3B1	4.68%	3.05%	6.92%	0	0
HSBP1	Heat shock factor-binding protein 1	4.40%	2.86%	6.73%	0	0
HSD11B1	Corticosteroid 11-beta-dehydrogenase isozyme 1	3.25%	1.84%	5.05%	0	0
HTRA2	Serine protease HTRA2, mitochondrial	0.84%	0.22%	1.77%	0.004	0.004581081
ICA1	Islet cell autoantigen 1	1.10%	0.40%	2.21%	0	0
ICAM3	Intercellular adhesion molecule 3	3.07%	1.85%	4.70%	0	0
IFI30	Gamma-interferon-inducible lysosomal thiol reductase	-0.69%	-1.55%	-0.14%	0.012	0.012873418
IFIT3	Interferon-induced protein with tetratricopeptide repeats 3	1.19%	0.34%	2.26%	0.002	0.002417112
IFNGR2	Interferon gamma receptor 2	2.76%	1.51%	4.43%	0	0
IFNL1	Interferon lambda-1	4.18%	2.66%	6.41%	0	0
IFNL2	Interferon lambda-2	3.37%	1.88%	5.29%	0	0
IFNW1	Interferon omega-1	3.79%	2.57%	5.61%	0	0
IFT20	Intraflagellar transport protein 20 homolog	1.94%	0.95%	3.24%	0	0
IGBP1	Immunoglobulin-binding protein 1	6.05%	4.18%	8.89%	0	0
IGDCC3	Immunoglobulin superfamily DCC subclass member 3	2.57%	1.49%	4.13%	0	0
IGF1R	Insulin-like growth factor 1 receptor	3.70%	2.37%	5.75%	0	0
IGFBP2	Insulin-like growth factor-binding protein 2	9.34%	6.86%	12.96%	0	0
IGFBP3	Insulin-like growth factor-binding protein 3	3.42%	1.69%	5.61%	0	0
IGFBP7	Insulin-like growth factor-binding protein 7	6.56%	4.43%	9.26%	0	0
IGLC2	Immunoglobulin lambda constant 2	2.10%	1.04%	3.44%	0	0
IGLON5	IgLON family member 5	7.06%	5.18%	9.78%	0	0
IGSF3	Immunoglobulin superfamily member 3	4.24%	2.71%	6.35%	0	0
IGSF8	Immunoglobulin superfamily member 8	2.44%	1.14%	3.93%	0	0
IL10RA	Interleukin-10 receptor subunit alpha	1.72%	0.79%	3.08%	0	0
IL10RB	Interleukin-10 receptor subunit beta	2.90%	1.68%	4.64%	0	0
IL11	Interleukin-11	3.10%	1.93%	4.74%	0	0
IL12B	Interleukin-12 subunit beta	6.74%	4.71%	9.71%	0	0
IL12RB1	Interleukin-12 receptor subunit beta-1	3.02%	2.04%	4.44%	0	0
IL13	Interleukin-13	5.68%	3.84%	8.17%	0	0
IL13RA1	Interleukin-13 receptor subunit alpha-1	4.08%	2.55%	5.97%	0	0
IL13RA2	Interleukin-13 receptor subunit alpha-2	8.19%	5.89%	11.29%	0	0
IL15	Interleukin-15	4.06%	2.74%	5.93%	0	0
IL15RA	Interleukin-15 receptor subunit alpha	1.55%	0.74%	2.76%	0	0
IL16	Pro-interleukin-16	3.61%	2.29%	5.39%	0	0
IL17A	Interleukin-17A	3.94%	2.51%	5.84%	0	0
IL17C	Interleukin-17C	8.97%	6.56%	12.18%	0	0
IL17D	Interleukin-17D	4.32%	3.00%	6.17%	0	0
IL17F	Interleukin-17F	2.35%	1.20%	3.81%	0	0
IL17RA	Interleukin-17 receptor A	5.96%	4.15%	8.48%	0	0
IL17RB	Interleukin-17 receptor B	6.08%	4.07%	8.90%	0	0
IL18	Interleukin-18	1.67%	0.55%	3.14%	0.004	0.004581081
IL18BP	Interleukin-18-binding protein	5.10%	3.55%	7.39%	0	0
IL1A	Interleukin-1 alpha	2.08%	0.99%	3.64%	0	0
IL1B	Interleukin-1 beta	4.91%	3.31%	7.10%	0	0
IL20	Interleukin-20	2.06%	0.94%	3.75%	0	0
IL22RA1	Interleukin-22 receptor subunit alpha-1	3.58%	2.17%	5.65%	0	0
IL25	Interleukin-25	1.02%	0.33%	1.95%	0	0
IL33	Interleukin-33	1.72%	0.75%	3.00%	0	0
IL34	Interleukin-34	1.04%	0.36%	2.07%	0.002	0.002417112
IL4R	Interleukin-4 receptor subunit alpha	3.23%	1.72%	5.20%	0	0
IL5	Interleukin-5	3.89%	2.49%	5.97%	0	0
IL6R	Interleukin-6 receptor subunit alpha	0.46%	0.07%	1.14%	0.022	0.022772519
IL6ST	Interleukin-6 receptor subunit beta	0.92%	0.32%	1.78%	0.002	0.002417112
ILKAP	Integrin-linked kinase-associated serine/threonine phosphatase 2C	2.43%	1.25%	3.83%	0	0
IMPA1	Inositol monophosphatase 1	3.17%	1.98%	4.79%	0	0
IMPG1	Interphotoreceptor matrix proteoglycan 1	-1.51%	-2.80%	-0.52%	0.002	0.002417112
INHBB	Inhibin beta B chain	1.88%	0.92%	3.38%	0	0
INPP5J	Phosphatidylinositol 4,5-bisphosphate 5-phosphatase A	8.94%	6.40%	12.51%	0	0
IPCEF1	Interactor protein for cytohesin exchange factors 1	7.46%	4.94%	10.73%	0	0
IRAG2	Inositol 1,4,5-triphosphate receptor associated 2	0.93%	0.31%	1.97%	0.004	0.004581081
IRAK1	Interleukin-1 receptor-associated kinase 1	2.85%	1.58%	4.52%	0	0
ISM2	Isthmin-2	1.43%	0.63%	2.60%	0	0
IST1	IST1 homolog	7.62%	5.40%	10.68%	0	0
ITGAM	Integrin alpha-M	2.10%	1.10%	3.55%	0	0
ITGAV	Integrin alpha-V	2.60%	1.53%	4.13%	0	0
ITGB5	Integrin beta-5	1.15%	0.45%	2.17%	0	0
JPT2	Jupiter microtubule associated homolog 2	6.07%	4.01%	9.22%	0	0
JUN	Transcription factor AP-1	2.77%	1.39%	4.64%	0	0
KAZN	Kazrin	1.26%	0.29%	2.69%	0.004	0.004581081
KCNC4	Potassium voltage-gated channel subfamily C member 4	6.15%	3.83%	9.15%	0	0
KDM3A	Lysine-specific demethylase 3A	2.69%	1.35%	4.63%	0	0
KDR	Vascular endothelial growth factor receptor 2	1.97%	0.91%	3.67%	0	0

KIAA2013	Uncharacterized protein KIAA2013	-0.81%	-1.91%	-0.27%	0	0
KIF22	Kinesin-like protein KIF22	1.09%	0.30%	2.36%	0.008	0.008805195
LEPR	Leptin receptor	2.27%	1.29%	3.70%	0	0
LGALS7 LGALS7B	Galectin-7	2.54%	1.60%	3.94%	0	0
LGALS9	Galectin-9	1.56%	0.61%	3.10%	0	0
LIFR	Leukemia inhibitory factor receptor	0.78%	0.16%	1.79%	0.01	0.010848
LILRA3	Leukocyte immunoglobulin-like receptor subfamily A member 3	-1.23%	-2.44%	-0.45%	0	0
LPL	Lipoprotein lipase	4.42%	2.91%	6.78%	0	0
LSM8	U6 snRNA-associated Sm-like protein LSm8	1.95%	0.85%	3.53%	0	0
LUZP2	Leucine zipper protein 2	4.25%	2.52%	6.85%	0	0
LY75	Lymphocyte antigen 75	2.14%	1.10%	3.72%	0	0
LYZL2	Lysozyme-like protein 2	0.70%	0.06%	1.68%	0.032	0.032527736
MAPT	Microtubule-associated protein tau	1.64%	0.62%	3.00%	0	0
MARCO	Macrophage receptor MARCO	3.64%	2.13%	6.09%	0	0
MAVS	Mitochondrial antiviral-signaling protein	0.90%	0.18%	2.05%	0.004	0.004581081
MAX	Protein max	1.66%	0.60%	3.09%	0.002	0.002417112
MB	Myoglobin	3.78%	2.18%	5.91%	0	0
MBL2	Mannose-binding protein C	6.40%	4.33%	9.37%	0	0
MENT	Protein MENT	1.26%	0.48%	2.62%	0	0
MEP1B	Meprin A subunit beta	2.24%	1.17%	3.98%	0	0
MIA	Melanoma-derived growth regulatory protein	3.98%	2.50%	6.04%	0	0
MILR1	Allergin-1	2.63%	1.44%	4.33%	0	0
MLN	Promotilin	5.23%	3.35%	8.13%	0	0
MME	Neprilysin	1.70%	0.76%	3.03%	0	0
MMP3	Stromelysin-1	4.66%	2.83%	7.75%	0	0
MOCS2	Molybdopterin synthase catalytic subunit	1.40%	0.54%	2.77%	0.002	0.002417112
MPHOSPH8	M-phase phosphoprotein 8	3.15%	1.70%	5.25%	0	0
MRPL28	39S ribosomal protein L28, mitochondrial	0.85%	0.17%	1.80%	0.012	0.012873418
MRPL46	39S ribosomal protein L46, mitochondrial	4.03%	2.38%	6.31%	0	0
MSLN	Mesothelin	1.54%	0.54%	3.14%	0	0
MSMB	Beta-microseminoprotein	2.53%	1.31%	4.26%	0	0
MTSS2	Protein MTSS 2	-0.66%	-1.64%	-0.06%	0.02	0.020861538
MUC13	Mucin-13	3.74%	2.43%	5.95%	0	0
MUC16	Mucin-16	2.31%	1.08%	3.97%	0	0
MVK	Mevalonate kinase	3.69%	2.22%	5.91%	0	0
MYL1	Myosin light chain 1/3, skeletal muscle isoform	2.24%	1.00%	3.91%	0	0
MYL6B	Myosin light chain 6B	1.69%	0.75%	3.41%	0	0
MYOC	Myocilin	1.79%	0.64%	3.34%	0.002	0.002417112
NAA10	N-alpha-acetyltransferase 10	12.64%	8.81%	18.12%	0	0
NMI	N-myc-interactor	3.08%	1.63%	5.14%	0	0
NOTCH3	Neurogenic locus notch homolog protein 3	1.21%	0.45%	2.40%	0	0
NPPB	Natriuretic peptides B	8.57%	5.83%	13.01%	0	0
NPTN	Neuroplastin	1.93%	0.99%	3.40%	0	0
NPTX2	Neuronal pentraxin-2	1.39%	0.33%	2.87%	0.01	0.010848
NT5E	5'-nucleotidase	3.00%	1.50%	5.00%	0	0
NUBP1	Cytosolic Fe-S cluster assembly factor NUBP1	2.13%	0.89%	3.76%	0	0
NXPH3	Neurexophilin-3	0.95%	0.17%	2.19%	0.01	0.010848
ORM1	Alpha-1-acid glycoprotein 1	2.92%	1.50%	4.92%	0	0
OSTN	Osteocrin	3.23%	1.23%	5.57%	0	0
OTOA	Otoancorin	1.00%	0.23%	2.11%	0.006	0.006690789
OTUD7B	OTU domain-containing protein 7B	4.63%	2.89%	7.12%	0	0
PACS2	Phosphofurin acidic cluster sorting protein 2	1.06%	0.34%	2.32%	0.004	0.004581081
PADI4	Protein-arginine deiminase type-4	0.98%	0.24%	2.16%	0.004	0.004581081
PAEP	Glycodelin	3.05%	1.52%	5.29%	0	0
PAMR1	Inactive serine protease PAMR1	3.79%	2.14%	6.09%	0	0
PCDH17	Protocadherin-17	5.57%	3.57%	8.32%	0	0
PDGFC	Platelet-derived growth factor C	1.33%	0.56%	2.49%	0	0
PDIA3	Protein disulfide-isomerase A3	1.37%	0.36%	2.97%	0.006	0.006690789
PEAR1	Platelet endothelial aggregation receptor 1	-2.05%	-3.83%	-0.95%	0	0
PER3	Period circadian protein homolog 3	2.96%	1.59%	4.72%	0	0
PGLYRP2	N-acetylmuramoyl-L-alanine amidase	4.30%	2.81%	6.47%	0	0
PLA2G1B	Phospholipase A2	2.53%	1.39%	4.33%	0	0
PLXDC1	Plexin domain-containing protein 1	2.28%	1.04%	4.06%	0	0
PM20D1	N-fatty-acyl-amino acid synthase/hydrolase PM20D1	2.37%	1.27%	4.09%	0	0
PMM2	Phosphomannomutase 2	1.37%	0.47%	2.81%	0	0
PMS1	PMS1 protein homolog 1	1.26%	0.42%	2.57%	0	0
PODXL	Podocalyxin	1.56%	0.39%	3.05%	0.004	0.004581081
PON2	Serum paraoxonase/arylesterase 2	0.93%	0.19%	2.04%	0.01	0.010848
PON3	Serum paraoxonase/lactonase 3	1.07%	0.36%	2.23%	0.002	0.002417112
PPIB	Peptidyl-prolyl cis-trans isomerase B	0.86%	0.22%	1.89%	0.004	0.004581081
PPIE	Peptidyl-prolyl cis-trans isomerase E	6.99%	4.56%	10.98%	0	0
PPP1R12B	Protein phosphatase 1 regulatory subunit 12B	1.15%	0.36%	2.38%	0.002	0.002417112
PPP1R9B	Neurabin-2	2.18%	1.25%	3.60%	0	0
PRC1	Protein regulator of cytokinesis 1	1.35%	0.50%	2.66%	0.002	0.002417112
PRG2	Bone marrow proteoglycan	1.82%	0.88%	3.36%	0	0
PRKAG3	5'-AMP-activated protein kinase subunit gamma-3	7.98%	5.36%	11.49%	0	0
PRKD2	Serine/threonine-protein kinase D2	1.16%	0.41%	2.24%	0	0
PRKG1	cGMP-dependent protein kinase 1	2.29%	0.94%	4.27%	0.002	0.002417112
PROS1	Vitamin K-dependent protein S	4.69%	2.91%	7.19%	0	0
PRSS8	Prostasin	0.78%	0.12%	1.70%	0.024	0.024691958
PSAPL1	Proactivator polypeptide-like 1	1.03%	0.29%	2.20%	0.006	0.006690789
PSMD9	26S proteasome non-ATPase regulatory subunit 9	1.02%	0.26%	2.16%	0.008	0.008805195
PTGES2	Prostaglandin E synthase 2	0.67%	0.12%	1.57%	0.002	0.002417112
PTPRZ1	Receptor-type tyrosine-protein phosphatase zeta	1.30%	0.52%	2.48%	0	0
PTS	6-pyruvoyl tetrahydrobiopterin synthase	2.54%	1.28%	4.44%	0	0
PVR	Poliovirus receptor	1.25%	0.44%	2.54%	0.002	0.002417112
PXDNL	Peroxidasin-like protein	1.11%	0.26%	2.32%	0.008	0.008805195
QPCT	Glutaminyl-peptide cyclotransferase	4.43%	2.63%	6.98%	0	0
QSOX1	Sulfhydryl oxidase 1	2.65%	1.36%	4.45%	0	0
RAB11FIP3	Rab11 family-interacting protein 3	0.66%	0.12%	1.58%	0.004	0.004581081
RAB39B	Ras-related protein Rab-39B	2.45%	1.27%	4.07%	0	0
RASGRF1	Ras-specific guanine nucleotide-releasing factor 1	4.89%	3.08%	7.59%	0	0
RBFOX3	RNA binding protein fox-1 homolog 3	1.44%	0.47%	2.88%	0.004	0.004581081
RET	Proto-oncogene tyrosine-protein kinase receptor Ret	4.51%	2.85%	6.90%	0	0
RLN1	Prorelaxin H1	4.46%	2.52%	7.21%	0	0
ROBO1	Roundabout homolog 1	0.99%	0.33%	2.02%	0.002	0.002417112
SCGB2A2	Mammaglobin-A	2.12%	0.93%	3.88%	0	0
SCPEP1	Retinoid-inducible serine carboxypeptidase	7.91%	5.86%	11.09%	0	0
SCRN1	Secernin-1	3.38%	1.97%	5.41%	0	0
SEC31A	Protein transport protein Sec31A	4.99%	3.34%	7.74%	0	0
SEMA4C	Semaphorin-4C	5.55%	3.57%	8.33%	0	0
SERPINA1	Alpha-1-antitrypsin	3.58%	2.05%	6.00%	0	0
SERPINA6	Corticosteroid-binding globulin	0.91%	0.24%	1.93%	0.002	0.002417112
SERPINB9	Serpin B9	1.61%	0.65%	3.03%	0	0
SERPINC1	Antithrombin-III	9.00%	6.30%	13.12%	0	0
SH2B3	SH2B adapter protein 3	0.99%	0.21%	2.06%	0.018	0.018862442
SHC1	SHC-transforming protein 1	1.90%	0.63%	3.54%	0.002	0.002417112
SHH	Sonic hedgehog protein	2.38%	1.34%	3.94%	0	0
SIGLEC15	Sialic acid-binding Ig-like lectin 15	0.88%	0.20%	1.98%	0.002	0.002417112
SIL1	Nucleotide exchange factor SIL1	1.04%	0.24%	2.23%	0.006	0.006690789
SKAP2	Src kinase-associated phosphoprotein 2	1.29%	0.49%	2.59%	0	0
SLC4A1	Band 3 anion transport protein	1.29%	0.46%	2.57%	0	0
SMNDC1	Survival of motor neuron-related-splicing factor 30	5.81%	3.33%	9.00%	0	0
SMPD1	Sphingomyelin phosphodiesterase	2.92%	1.55%	4.82%	0	0
SNAPIN	SNARE-associated protein Snapin	0.69%	0.09%	1.69%	0.014	0.01485446
SNED1	Sushi, nidogen and EGF-like domain-containing protein 1	0.76%	0.21%	1.84%	0.006	0.006690789
SNX15	Sorting nexin-15	3.77%	2.31%	5.77%	0	0
SPINK5	Serine protease inhibitor Kazal-type 5	1.09%	0.27%	2.15%	0	0
SRPK2	SRSF protein kinase 2	4.39%	2.75%	6.68%	0	0
SSC5D	Soluble scavenger receptor cysteine-rich domain-containing protein SSC5D	1.58%	0.63%	3.14%	0	0
ST13	Hsc70-interacting protein	0.77%	0.19%	1.83%	0.002	0.002417112
STX16	Syntaxin-16	6.07%	4.36%	9.04%	0	0
STX1B	Syntaxin-1B	1.18%	0.38%	2.36%	0	0
STX3	Syntaxin-3	5.43%	3.77%	7.66%	0	0
STX4	Syntaxin-4	5.78%	4.12%	8.49%	0	0
TBCA	Tubulin-specific chaperone A	0.95%	0.21%	2.03%	0.008	0.008805195
TBCB	Tubulin-folding cofactor B	1.13%	0.35%	2.35%	0.002	0.002417112
TBCC	Tubulin-specific chaperone C	3.03%	1.77%	4.81%	0	0
TCOF1	Treacle protein	2.55%	1.60%	4.14%	0	0
TDGF1	Teratocarcinoma-derived growth factor 1	0.79%	0.17%	1.87%	0.006	0.006690789
TEF	Thyrotroph embryonic factor	4.80%	2.96%	7.34%	0	0

TERF1	Telomeric repeat-binding factor 1	2.35%	1.10%	4.14%	0.002	0.002417112
TET2	Methylcytosine dioxygenase TET2	1.07%	0.36%	2.18%	0	0
TFF3	Trefoil factor 3	1.65%	0.62%	3.05%	0.002	0.002417112
THBS4	Thrombospondin-4	6.72%	4.73%	9.59%	0	0
THPO	Thrombopoietin	3.99%	2.37%	6.39%	0	0
THRAP3	Thyroid hormone receptor-associated protein 3	1.05%	0.34%	2.09%	0	0
TIMM8A	Mitochondrial import inner membrane translocase subunit Tim8 A	0.75%	0.16%	1.69%	0.008	0.008805195
TK1	Thymidine kinase, cytosolic	1.15%	0.45%	2.31%	0	0
TLR4	Toll-like receptor 4	0.57%	0.07%	1.49%	0.018	0.018862442
TMEM25	Transmembrane protein 25	0.61%	0.06%	1.55%	0.028	0.028633484
TMOD4	Tropomodulin-4	0.69%	0.12%	1.52%	0.022	0.022772519
TNF	Tumor necrosis factor	1.08%	0.45%	2.13%	0	0
TNFRSF11B	Tumor necrosis factor receptor superfamily member 11B	1.79%	0.70%	3.53%	0.004	0.004581081
TNFSF11	Tumor necrosis factor ligand superfamily member 11	1.74%	0.57%	3.34%	0.002	0.002417112
TPSD1	Tryptase delta	1.09%	0.39%	2.16%	0	0
TSLP	Thymic stromal lymphopoietin	1.17%	0.52%	2.17%	0	0
TXNDC15	Thioredoxin domain-containing protein 15	0.95%	0.29%	1.98%	0.002	0.002417112
TXNL1	Thioredoxin-like protein 1	3.87%	2.25%	6.41%	0	0
TXNRD1	Thioredoxin reductase 1, cytoplasmic	1.30%	0.50%	2.63%	0	0
TYRO3	Tyrosine-protein kinase receptor TYRO3	1.04%	0.30%	2.13%	0.002	0.002417112
UBAC1	Ubiquitin-associated domain-containing protein 1	0.78%	0.18%	1.92%	0.006	0.006690789

Models were adjusted for age, sex, ethnicity, Townsend deprivation index, smoking status, alcohol intake, physical activity, body mass index, household income, education status, hypertension, high density lipoprotein, triglyceride, lipid-lowering drug, coronary heart disease at baseline and stroke at baseline;

*0 indicates that the *FDR* value is smaller than the smallest value displayed (0.002).

Supplementary Table 16 KEGG pathways enriched by the significant mediators between PRISm and HF

ID	Description	GeneRatio	BgRatio	*P* value	*P*.adjust	qvalue	geneID	Count
hsa04060	Cytokine-cytokine receptor interaction	72/397	295/8390	6.75E-33	2.11E-30	1.73E-30	IFNL1/IFNL2/IL34/IL17F/I	72
hsa04630	JAK-STAT signaling pathway	37/397	166/8390	9.18E-16	1.43E-13	1.17E-13	IFNL1/IFNL2/TSLP/IL22R	37
hsa04061	Viral protein interaction with cytokine and cytokine receptor	25/397	100/8390	3.27E-12	3.41E-10	2.79E-10	IL34/IL22RA1/IL20/CCL27	25
hsa04640	Hematopoietic cell lineage	24/397	99/8390	1.83E-11	1.43E-09	1.17E-09	TNF/THPO/MME/ITGAM/	24
hsa04657	IL-17 signaling pathway	22/397	94/8390	2.71E-10	1.69E-08	1.39E-08	IL17F/IL25/IL17RB/IL17D	22
hsa05323	Rheumatoid arthritis	19/397	93/8390	4.95E-08	2.57E-06	2.11E-06	TNFSF11/TNF/TLR4/CXC	19
hsa04151	PI3K-Akt signaling pathway	40/397	354/8390	2.11E-07	9.4E-06	7.7E-06	PDGFC/CREB3/BCL2L11/	40
hsa05321	Inflammatory bowel disease	14/397	65/8390	1.5E-06	5.85E-05	4.8E-05	IL17F/TNF/TLR4/JUN/IL1	14
hsa04610	Complement and coagulation cascades	15/397	86/8390	1.02E-05	0.000354	0.00029	PROS1/SERPINA1/MBL2/	15
hsa04668	TNF signaling pathway	17/397	114/8390	2.3E-05	0.000718	0.000589	CREB3/BAG4/FADD/TNF/	17
hsa04514	Cell adhesion molecules	20/397	157/8390	4.74E-05	0.001182	0.000969	CD99L2/CD226/PVR/CD9	20
hsa04066	HIF-1 signaling pathway	16/397	109/8390	4.87E-05	0.001182	0.000969	TLR4/IL6R/IGF1R/IFNGR	16
hsa05133	Pertussis	13/397	76/8390	4.93E-05	0.001182	0.000969	TNF/TLR4/JUN/ITGAM/IR	13
hsa04210	Apoptosis	18/397	136/8390	6.82E-05	0.001519	0.001245	DIABLO/HTRA2/BCL2L11	18
hsa01521	EGFR tyrosine kinase inhibitor resistance	13/397	79/8390	7.48E-05	0.001556	0.001276	PDGFC/BCL2L11/SHC1/K	13
hsa04010	MAPK signaling pathway	30/397	302/8390	8.89E-05	0.001659	0.00136	PDGFC/ARTN/FGF16/TNF	30
hsa04215	Apoptosis - multiple species	8/397	32/8390	9.04E-05	0.001659	0.00136	DIABLO/HTRA2/BCL2L11	8
hsa05152	Tuberculosis	21/397	180/8390	0.000113	0.001954	0.001602	CLEC7A/ARHGEF12/COR	21
hsa05171	Coronavirus disease - COVID-19	24/397	232/8390	0.000246	0.00404	0.003312	MAVS/TNF/TLR4/CCL2/E	24
hsa04064	NF-kappa B signaling pathway	14/397	104/8390	0.000363	0.005661	0.004641	TNFSF11/TNF/TLR4/CXC	14
hsa04940	Type I diabetes mellitus	8/397	43/8390	0.000789	0.011726	0.009613	TNF/IL12B/IL1B/IL1A/ICA	8
hsa05162	Measles	16/397	139/8390	0.000842	0.011945	0.009793	MAVS/FADD/TLR4/EIF2A	16
hsa04936	Alcoholic liver disease	16/397	142/8390	0.001062	0.014406	0.01181	PRKAG3/IL17RA/ADIPOQ	16
hsa04658	Th1 and Th2 cell differentiation	12/397	92/8390	0.00125	0.01617	0.013257	DLL1/NOTCH3/JUN/IL13/	12
hsa05134	Legionellosis	9/397	57/8390	0.001296	0.01617	0.013257	TNF/TLR4/ITGAM/IL18/IL	9
hsa05144	Malaria	8/397	50/8390	0.002189	0.026263	0.021531	TNF/TLR4/THBS4/CCL2/I	8
hsa05310	Asthma	6/397	31/8390	0.002904	0.032737	0.026839	EPX/TNF/PRG2/IL13/IL5/	6
hsa05164	Influenza A	17/397	171/8390	0.002952	0.032737	0.026839	IL33/MAVS/TPSD1/FADD	17
hsa05142	Chagas disease	12/397	102/8390	0.003043	0.032737	0.026839	FADD/TNF/TLR4/CCL2/JU	12
hsa04974	Protein digestion and absorption	12/397	103/8390	0.003301	0.034333	0.028147	ATP1B4/MME/MEP1B/EL	12
hsa05205	Proteoglycans in cancer	19/397	205/8390	0.003803	0.038272	0.031377	ARHGEF12/FZD10/EZR/T	19
hsa04062	Chemokine signaling pathway	18/397	192/8390	0.004245	0.041387	0.033931	CCL27/CCL26/CXCL14/SI	18
hsa04659	Th17 cell differentiation	12/397	108/8390	0.004872	0.046059	0.037761	IL17F/JUN/IL17A/IL12RB1	12
hsa05030	Cocaine addiction	7/397	49/8390	0.007751	0.071124	0.05831	CREB3/JUN/FOSB/DLG4/	7
hsa05330	Allograft rejection	6/397	38/8390	0.008242	0.073475	0.060238	TNF/IL12B/IL5/GZMB/CD	6
hsa05224	Breast cancer	14/397	147/8390	0.009653	0.083436	0.068404	DLL1/FZD10/FGF16/TNFS	14
hsa05140	Leishmaniasis	9/397	77/8390	0.010136	0.083436	0.068404	TNF/TLR4/JUN/ITGAM/IR	9
hsa05163	Human cytomegalovirus infection	19/397	225/8390	0.010162	0.083436	0.068404	ARHGEF12/CREB3/FADD	19
hsa04961	Endocrine and other factor-regulated calcium reabsorption	7/397	53/8390	0.011838	0.094707	0.077644	DNM3/ATP1B4/ESR1/DN	7
hsa04621	NOD-like receptor signaling pathway	16/397	186/8390	0.014916	0.116341	0.095381	MAVS/ATG16L1/GABAR	16
hsa05145	Toxoplasmosis	11/397	112/8390	0.016654	0.126736	0.103903	TNF/TLR4/IRAK1/IL12B/I	11
hsa00480	Glutathione metabolism	7/397	57/8390	0.017293	0.128464	0.10532	GSTT2B/CHAC2/GGCT/G	7
hsa04012	ErbB signaling pathway	9/397	85/8390	0.018616	0.135072	0.110737	SHC1/JUN/EREG/ERBB4/	9
hsa05218	Melanoma	8/397	72/8390	0.019847	0.140734	0.115379	PDGFC/FGF16/GADD45B/	8
hsa04115	p53 signaling pathway	8/397	73/8390	0.021404	0.144264	0.118273	CHEK2/GADD45B/CD82/I	8
hsa05146	Amoebiasis	10/397	102/8390	0.022131	0.144264	0.118273	TNF/TLR4/SERPINB9/ITG	10
hsa05416	Viral myocarditis	7/397	60/8390	0.022404	0.144264	0.118273	EIF4G3/DAG1/CD40/CD8	7
hsa05207	Chemical carcinogenesis - receptor activation	17/397	212/8390	0.023071	0.144264	0.118273	GSTT2B/DLL1/CREB3/FG	17
hsa04810	Regulation of actin cytoskeleton	18/397	229/8390	0.023624	0.144264	0.118273	PDGFC/BRK1/ARHGEF12	18
hsa05130	Pathogenic Escherichia coli infection	16/397	197/8390	0.024305	0.144264	0.118273	BRK1/ARHGEF12/FADD/	16
hsa04211	Longevity regulating pathway	9/397	89/8390	0.024396	0.144264	0.118273	PRKAG3/CREB3/ADIPOQ	9
hsa04512	ECM-receptor interaction	9/397	89/8390	0.024396	0.144264	0.118273	GP6/THBS4/ITGB5/ITGA	9
hsa04918	Thyroid hormone synthesis	8/397	75/8390	0.024775	0.144264	0.118273	ATP1B4/CREB3/GSR/ATF	8
hsa04625	C-type lectin receptor signaling pathway	10/397	104/8390	0.024969	0.144264	0.118273	CLEC4D/CLEC7A/IL17D/	10
hsa05417	Lipid and atherosclerosis	17/397	215/8390	0.026018	0.147594	0.121003	TNF/TLR4/CCL2/MMP3/J	17
hsa04672	Intestinal immune network for IgA production	6/397	49/8390	0.027066	0.150798	0.12363	CXCL12/IL15RA/IL15/IL5/	6
hsa04145	Phagosome	13/397	152/8390	0.027765	0.151979	0.124598	CLEC7A/CORO1A/HGS/M	13
hsa05143	African trypanosomiasis	5/397	37/8390	0.02897	0.155837	0.127761	APOL1/TNF/IL18/IL12B/IL	5
hsa04014	Ras signaling pathway	18/397	236/8390	0.030738	0.162548	0.133263	PDGFC/FGF16/SHC1/RAS	18
hsa05418	Fluid shear stress and atherosclerosis	12/397	139/8390	0.031636	0.164235	0.134646	GSTT2B/TNF/CCL2/KDR/	12
hsa05340	Primary immunodeficiency	5/397	38/8390	0.03211	0.164235	0.134646	CD40/CD4/CD3E/CD3D/A	5
hsa00430	Taurine and hypotaurine metabolism	3/397	16/8390	0.037206	0.187233	0.1535	GGT5/GGT1/GAD1	3
hsa04217	Necroptosis	13/397	159/8390	0.038068	0.188527	0.154561	IL33/CHMP6/FADD/TNF/	13
hsa04380	Osteoclast differentiation	11/397	128/8390	0.03973	0.193684	0.15879	LILRA3/TNFSF11/TNF/TN	11
hsa01522	Endocrine resistance	9/397	98/8390	0.041846	0.20086	0.164673	DLL1/SHC1/NOTCH3/JUN	9
hsa05161	Hepatitis B	13/397	162/8390	0.043225	0.203212	0.166601	MAVS/CREB3/FADD/TNF	13
hsa00910	Nitrogen metabolism	3/397	17/8390	0.043638	0.203212	0.166601	CA8/CA7/CA5A	3
hsa05332	Graft-versus-host disease	5/397	42/8390	0.046743	0.214467	0.175828	TNF/IL1B/IL1A/GZMB/CD	5
hsa05226	Gastric cancer	12/397	149/8390	0.049671	0.22301	0.182832	FZD10/FGF16/AXIN1/SHH	12
hsa05210	Colorectal cancer	8/397	86/8390	0.050034	0.22301	0.182832	BCL2L11/AXIN1/GADD45	8
hsa05169	Epstein-Barr virus infection	15/397	202/8390	0.055623	0.241414	0.19792	MAVS/BCL2L11/FADD/T	15
hsa01524	Platinum drug resistance	7/397	73/8390	0.056418	0.241414	0.19792	GSTT2B/FADD/GSTA1/C	7
hsa04620	Toll-like receptor signaling pathway	9/397	104/8390	0.057283	0.241414	0.19792	FADD/TNF/TLR4/JUN/IRA	9
hsa04935	Growth hormone synthesis, secretion and action	10/397	120/8390	0.057573	0.241414	0.19792	GHRL/CREB3/SHC1/IGFB	10
hsa01210	2-Oxocarboxylic acid metabolism	3/397	19/8390	0.058032	0.241414	0.19792	GOT1/BCAT1/ACY1	3
hsa05235	PD-L1 expression and PD-1 checkpoint pathway in cancer	8/397	89/8390	0.059063	0.241623	0.198092	TLR4/JUN/IFNGR2/EGFR/	8
hsa05135	Yersinia infection	11/397	137/8390	0.059631	0.241623	0.198092	ARHGEF12/SKAP2/TNF/T	11
hsa04915	Estrogen signaling pathway	11/397	138/8390	0.062186	0.24773	0.203099	CREB3/SHC1/JUN/FKBP5/	11
hsa04932	Non-alcoholic fatty liver disease	12/397	155/8390	0.063343	0.24773	0.203099	PRKAG3/BCL2L11/ADIPC	12
hsa04623	Cytosolic DNA-sensing pathway	7/397	75/8390	0.063521	0.24773	0.203099	IL33/MAVS/FADD/IL18/IL	7
hsa04130	SNARE interactions in vesicular transport	4/397	33/8390	0.06853	0.263968	0.216411	STX1B/STX16/STX4/STX	4
hsa04015	Rap1 signaling pathway	15/397	210/8390	0.072666	0.276485	0.226673	PDGFC/PRKD2/FGF16/KD	15
hsa04964	Proximal tubule bicarbonate reclamation	3/397	23/8390	0.092478	0.347627	0.284998	ATP1B4/ATP1B3/ATP1B2	3
hsa04142	Lysosome	10/397	132/8390	0.095037	0.352993	0.289397	PSAPL1/CD164/SMPD1/G	10
hsa04933	AGE-RAGE signaling pathway in diabetic complications	8/397	100/8390	0.100496	0.364821	0.299095	TNF/CCL2/JUN/IL1B/IL1A	8
hsa04510	Focal adhesion	14/397	203/8390	0.10056	0.364821	0.299095	PDGFC/THBS4/SHC1/RAS	14
hsa05166	Human T-cell leukemia virus 1 infection	15/397	222/8390	0.104148	0.369843	0.303211	CDC26/CHEK2/CREB3/TN	15
hsa00600	Sphingolipid metabolism	5/397	53/8390	0.104315	0.369843	0.303211	PSAPL1/ASAH2/SMPD1/G	5
hsa01232	Nucleotide metabolism	7/397	85/8390	0.106819	0.374466	0.307001	DCTPP1/GMPR2/TK1/NT	7
hsa04972	Pancreatic secretion	8/397	102/8390	0.109433	0.378183	0.310049	ATP1B4/PLA2G1B/CPB2/	8
hsa04722	Neurotrophin signaling pathway	9/397	119/8390	0.11035	0.378183	0.310049	BEX3/SH2B3/SHC1/JUN/I	9
hsa04911	Insulin secretion	7/397	86/8390	0.11185	0.378183	0.310049	ATP1B4/CREB3/ATF2/AT	7
hsa05120	Epithelial cell signaling in Helicobacter pylori infection	6/397	70/8390	0.112728	0.378183	0.310049	PTPRZ1/JUN/CXCL1/EGF	6
hsa04622	RIG-I-like receptor signaling pathway	6/397	71/8390	0.118533	0.39343	0.322549	MAVS/FADD/TNF/IL12B/	6
hsa00790	Folate biosynthesis	3/397	26/8390	0.122631	0.402746	0.330186	PTS/MOCS2/ALPI	3
hsa05219	Bladder cancer	4/397	41/8390	0.127172	0.41331	0.338847	DAPK2/EGFR/HBEGF/CD	4
hsa05170	Human immunodeficiency virus 1 infection	14/397	212/8390	0.129681	0.416464	0.341433	FADD/TNF/TLR4/JUN/IRA	14
hsa04140	Autophagy - animal	10/397	141/8390	0.130812	0.416464	0.341433	RAB39B/ATG16L1/GABA	10
hsa00650	Butanoate metabolism	3/397	27/8390	0.133386	0.420368	0.344633	HMGCS1/GAD1/ECHS1	3
hsa00533	Glycosaminoglycan biosynthesis - keratan sulfate	2/397	14/8390	0.139824	0.436251	0.357655	B3GNT7/B4GALT1	2
hsa03320	PPAR signaling pathway	6/397	75/8390	0.143191	0.437921	0.359024	ADIPOQ/LPL/HMGCS1/F	6
hsa00860	Porphyrin metabolism	4/397	43/8390	0.144327	0.437921	0.359024	HEPH/HMOX1/HMBS/GU	4
hsa05222	Small cell lung cancer	7/397	92/8390	0.14457	0.437921	0.359024	GADD45B/MAX/ITGAV/F	7
hsa04970	Salivary secretion	7/397	93/8390	0.150428	0.451283	0.369979	ATP1B4/PRKG1/CST3/CS	7
hsa04978	Mineral absorption	5/397	60/8390	0.15324	0.455342	0.373306	ATP1B4/HEPH/HMOX1/A	5
hsa05100	Bacterial invasion of epithelial cells	6/397	77/8390	0.156337	0.458119	0.375583	DNM3/CD2AP/SHC1/HCL	6
hsa04926	Relaxin signaling pathway	9/397	129/8390	0.157111	0.458119	0.375583	CREB3/SHC1/RLN1/JUN/	9
hsa04612	Antigen processing and presentation	6/397	78/8390	0.163103	0.466862	0.382752	IFI30/TNF/PDIA3/CTSS/C	6
hsa04721	Synaptic vesicle cycle	6/397	78/8390	0.163103	0.466862	0.382752	STX1B/DNM3/CPLX2/ST	6
hsa01523	Antifolate resistance	3/397	30/8390	0.167436	0.474909	0.389349	TNF/IL1B/FOLR3	3
hsa05225	Hepatocellular carcinoma	11/397	168/8390	0.172105	0.483755	0.396601	GSTT2B/FZD10/AXIN1/T	11
hsa05215	Prostate cancer	7/397	97/8390	0.174929	0.487301	0.399508	PDGFC/CREB3/MMP3/IG	7
hsa05217	Basal cell carcinoma	5/397	63/8390	0.17668	0.487658	0.399801	FZD10/AXIN1/SHH/GAD	5
hsa04024	cAMP signaling pathway	14/397	225/8390	0.179268	0.487658	0.399801	GPHA2/GHRL/ATP1B4/C	14
hsa04973	Carbohydrate digestion and absorption	4/397	47/8390	0.181094	0.487658	0.399801	ATP1B4/HK2/ATP1B3/AT	4

hsa04925	Aldosterone synthesis and secretion	7/397	98/8390	0.181309	0.487658	0.399801	PRKD2/ATP1B4/CREB3/A	7
hsa04270	Vascular smooth muscle contraction	9/397	134/8390	0.183606	0.489615	0.401405	MYL6B/ARHGEF12/PRKC	9
hsa00280	Valine, leucine and isoleucine degradation	4/397	48/8390	0.190736	0.5018	0.411395	HMGCS1/ECHS1/BCAT1/	4
hsa04136	Autophagy - other	3/397	32/8390	0.191392	0.5018	0.411395	ATG16L1/GABARAPL1/IC	3
hsa00520	Amino sugar and nucleotide sugar metabolism	4/397	49/8390	0.200536	0.520881	0.427038	GNPDA2/GNPDA1/PMM2/	4
hsa05202	Transcriptional misregulation in cancer	12/397	193/8390	0.203188	0.520881	0.427038	ASPSCR1/GADD45B/MMI	12
hsa00051	Fructose and mannose metabolism	3/397	33/8390	0.203678	0.520881	0.427038	PMM2/HK2/FBP1	3
hsa04710	Circadian rhythm	3/397	34/8390	0.21614	0.548258	0.449483	PRKAG3/PER3/ARNTL	3
hsa04979	Cholesterol metabolism	4/397	51/8390	0.220567	0.552892	0.453282	LPL/APOH/APOE/APOC1	4
hsa04660	T cell receptor signaling pathway	7/397	104/8390	0.221511	0.552892	0.453282	TNF/JUN/IL5/CSF2/CD4/C	7
hsa05031	Amphetamine addiction	5/397	69/8390	0.227151	0.562469	0.461134	CREB3/JUN/FOSB/ATF2/	5
hsa04928	Parathyroid hormone synthesis, secretion and action	7/397	106/8390	0.235569	0.578721	0.474458	CREB3/TNFSF11/EGFR/H	7
hsa05320	Autoimmune thyroid disease	4/397	53/8390	0.241099	0.58411	0.478876	IL5/GZMB/CD40/CD80	4
hsa00350	Tyrosine metabolism	3/397	36/8390	0.241507	0.58411	0.478876	GOT1/DBH/ADH1B	3
hsa04020	Calcium signaling pathway	14/397	240/8390	0.246288	0.591091	0.484599	PDGFC/FGF16/RET/KDR/	14
hsa05410	Hypertrophic cardiomyopathy	6/397	90/8390	0.252731	0.592267	0.485563	PRKAG3/TNF/ITGB5/ITG	6
hsa04137	Mitophagy - animal	5/397	72/8390	0.253831	0.592267	0.485563	GABARAPL1/JUN/CSNK2	5
hsa04960	Aldosterone-regulated sodium reabsorption	3/397	37/8390	0.254371	0.592267	0.485563	ATP1B4/ATP1B3/ATP1B2	3
hsa05216	Thyroid cancer	3/397	37/8390	0.254371	0.592267	0.485563	RET/GADD45B/CDH1	3
hsa04022	cGMP-PKG signaling pathway	10/397	167/8390	0.266544	0.616013	0.505031	ATP1B4/CREB3/GTF2IRD	10
hsa00220	Arginine biosynthesis	2/397	22/8390	0.279618	0.635627	0.521111	GOT1/ACY1	2
hsa00340	Histidine metabolism	2/397	22/8390	0.279618	0.635627	0.521111	HNMT/AOC1	2
hsa04650	Natural killer cell mediated cytotoxicity	8/397	132/8390	0.286929	0.635627	0.521111	TNF/SHC1/IFNGR2/GZMI	8
hsa04971	Gastric acid secretion	5/397	76/8390	0.290495	0.635627	0.521111	ATP1B4/EZR/GAST/ATP1	5
hsa05220	Chronic myeloid leukemia	5/397	76/8390	0.290495	0.635627	0.521111	SHC1/GADD45B/BCR/BCI	5
hsa05132	Salmonella infection	14/397	249/8390	0.29056	0.635627	0.521111	AHNAK/BRK1/DCTN2/FA	14
hsa04141	Protein processing in endoplasmic reticulum	10/397	171/8390	0.290692	0.635627	0.521111	SIL1/EDEM2/SEC31A/DN	10
hsa05213	Endometrial cancer	4/397	58/8390	0.294057	0.635627	0.521111	AXIN1/GADD45B/EGFR/C	4
hsa00515	Mannose type O-glycan biosynthesis	2/397	23/8390	0.297441	0.635627	0.521111	B4GAT1/B4GALT1	2
hsa00900	Terpenoid backbone biosynthesis	2/397	23/8390	0.297441	0.635627	0.521111	MVK/HMGCS1	2
hsa04614	Renin-angiotensin system	2/397	23/8390	0.297441	0.635627	0.521111	MME/ENPEP	2
hsa05412	Arrhythmogenic right ventricular cardiomyopathy	5/397	77/8390	0.299811	0.636334	0.521691	ITGB5/ITGAV/DSC2/DAG	5
hsa05150	Staphylococcus aureus infection	6/397	96/8390	0.30188	0.636397	0.521742	MBL2/ITGAM/FCAR/C1S/	6
hsa00534	Glycosaminoglycan biosynthesis - heparan sulfate / heparin	2/397	24/8390	0.315194	0.660003	0.541096	HS3ST3B1/EXTL1	2
hsa00380	Tryptophan metabolism	3/397	42/8390	0.319724	0.665027	0.545214	ECHS1/CAT/AOC1	3
hsa00590	Arachidonic acid metabolism	4/397	61/8390	0.326534	0.674693	0.553139	PTGES2/PLA2G1B/GGT5/	4
hsa00071	Fatty acid degradation	3/397	43/8390	0.332879	0.683278	0.560178	ECHS1/ADH1B/ACADM	3
hsa04962	Vasopressin-regulated water reabsorption	3/397	44/8390	0.346025	0.705461	0.578364	DCTN2/CREB3/STX4	3
hsa04152	AMPK signaling pathway	7/397	121/8390	0.348208	0.705461	0.578364	PRKAG3/CREB3/ADIPOQ	7
hsa00601	Glycosphingolipid biosynthesis - lacto and neolacto series	2/397	27/8390	0.36772	0.740186	0.606832	B4GALT1/FUT1	2
hsa04930	Type II diabetes mellitus	3/397	46/8390	0.372229	0.744458	0.610335	ADIPOQ/TNF/HK2	3
hsa04080	Neuroactive ligand-receptor interaction	19/397	367/8390	0.375687	0.746589	0.612082	GPHA2/GHRL/RLN1/MLN	19
hsa00620	Pyruvate metabolism	3/397	47/8390	0.385261	0.759044	0.622293	GRHPR/GLO1/ADH1B	3
hsa05131	Shigellosis	13/397	247/8390	0.386821	0.759044	0.622293	ATG16L1/TNF/TLR4/JUN/	13
hsa00010	Glycolysis / Gluconeogenesis	4/397	67/8390	0.39187	0.764146	0.626476	HK2/FBP1/ENO2/ADH1B	4
hsa04664	Fc epsilon RI signaling pathway	4/397	68/8390	0.402709	0.776353	0.636484	TNF/IL13/IL5/CSF2	4
hsa05022	Pathways of neurodegeneration - multiple diseases	24/397	476/8390	0.403106	0.776353	0.636484	RAB39B/HTRA2/FZD10/D	24
hsa04920	Adipocytokine signaling pathway	4/397	69/8390	0.413512	0.791508	0.648908	PRKAG3/ADIPOQ/TNF/LI	4
hsa00630	Glyoxylate and dicarboxylate metabolism	2/397	30/8390	0.418609	0.796378	0.652901	GRHPR/CAT	2
hsa00330	Arginine and proline metabolism	3/397	50/8390	0.423929	0.801611	0.657191	GOT1/GAMT/AOC1	3
hsa04068	FoxO signaling pathway	7/397	131/8390	0.42652	0.801652	0.657225	PRKAG3/GABARAPL1/BC	7
hsa00052	Galactose metabolism	2/397	31/8390	0.435113	0.806803	0.661448	HK2/B4GALT1	2
hsa00410	beta-Alanine metabolism	2/397	31/8390	0.435113	0.806803	0.661448	GAD1/ECHS1	2
hsa05167	Kaposi sarcoma-associated herpesvirus infection	10/397	194/8390	0.437018	0.806803	0.661448	FADD/EIF2AK2/JUN/IL6S	10
hsa05223	Non-small cell lung cancer	4/397	72/8390	0.445641	0.814646	0.667878	RET/GADD45B/FHIT/EGF	4
hsa04725	Cholinergic synapse	6/397	113/8390	0.446489	0.814646	0.667878	CREB3/GNGT1/CHRM1/B	6
hsa04520	Adherens junction	5/397	93/8390	0.451166	0.816179	0.669135	HEG1/IGF1R/EGFR/CSNK	5
hsa04934	Cushing syndrome	8/397	155/8390	0.452561	0.816179	0.669135	FZD10/CREB3/AXIN1/EGI	8
hsa01200	Carbon metabolism	6/397	115/8390	0.463359	0.829181	0.679794	HK2/GOT1/FBP1/ENO2/E	6
hsa05322	Systemic lupus erythematosus	7/397	136/8390	0.465433	0.829181	0.679794	TNF/CD40/CD80/C7/C1S/	7
hsa03450	Non-homologous end-joining	1/397	13/8390	0.467743	0.829181	0.679794	FEN1	1
hsa01230	Biosynthesis of amino acids	4/397	75/8390	0.477214	0.836465	0.685766	GOT1/ENO2/BCAT1/ACY	4
hsa05214	Glioma	4/397	75/8390	0.477214	0.836465	0.685766	SHC1/GADD45B/IGF1R/E	4
hsa04340	Hedgehog signaling pathway	3/397	56/8390	0.498425	0.858612	0.703923	SHH/GLI2/BCL2	3
hsa05165	Human papillomavirus infection	16/397	331/8390	0.502338	0.858612	0.703923	FZD10/CREB3/FADD/AXI	16
hsa00980	Metabolism of xenobiotics by cytochrome P450	4/397	78/8390	0.508076	0.858612	0.703923	GSTT2B/HSD11B1/GSTA1	4
hsa04071	Sphingolipid signaling pathway	6/397	121/8390	0.513076	0.858612	0.703923	ASAH2/TNF/SMPD1/CTSI	6
hsa04919	Thyroid hormone signaling pathway	6/397	121/8390	0.513076	0.858612	0.703923	ATP1B4/NOTCH3/ITGAV/	6
hsa00512	Mucin type O-glycan biosynthesis	2/397	36/8390	0.513566	0.858612	0.703923	C1GALT1C1/GCNT1	2
hsa00760	Nicotinate and nicotinamide metabolism	2/397	36/8390	0.513566	0.858612	0.703923	NT5E/CD38	2
hsa00603	Glycosphingolipid biosynthesis - globo and isoglobo series	1/397	15/8390	0.517	0.858612	0.703923	FUT1	1
hsa00730	Thiamine metabolism	1/397	15/8390	0.517	0.858612	0.703923	ALPI	1
hsa04550	Signaling pathways regulating pluripotency of stem cells	7/397	143/8390	0.518781	0.858612	0.703923	FZD10/AXIN1/LIFR/INHB	7
hsa00240	Pyrimidine metabolism	3/397	58/8390	0.522175	0.858612	0.703923	DCTPP1/TK1/NT5E	3
hsa00983	Drug metabolism - other enzymes	4/397	80/8390	0.528192	0.858612	0.703923	GSTT2B/TK1/GUSB/GST	4
hsa00250	Alanine, aspartate and glutamate metabolism	2/397	37/8390	0.528377	0.858612	0.703923	GOT1/GAD1	2
hsa01250	Biosynthesis of nucleotide sugars	2/397	37/8390	0.528377	0.858612	0.703923	PMM2/HK2	2
hsa04914	Progesterone-mediated oocyte maturation	5/397	102/8390	0.533069	0.861749	0.706494	CDC26/FZR1/KIF22/IGF1F	5
hsa00360	Phenylalanine metabolism	1/397	16/8390	0.539895	0.868285	0.711853	GOT1	1
hsa04730	Long-term depression	3/397	60/8390	0.545299	0.872478	0.715291	PRKG1/IGF1R/CRHR1	3
hsa04530	Tight junction	8/397	169/8390	0.551358	0.873209	0.71589	MYL6B/CGN/PRKAG3/EZ	8
hsa05010	Alzheimer disease	18/397	384/8390	0.552351	0.873209	0.71589	FZD10/FADD/AXIN1/TNF	18
hsa04072	Phospholipase D signaling pathway	7/397	148/8390	0.555682	0.873209	0.71589	PDGFC/DNM3/SHC1/F2R/	7
hsa00450	Selenocompound metabolism	1/397	17/8390	0.561708	0.873209	0.71589	TXNRD1	1
hsa04662	B cell receptor signaling pathway	4/397	84/8390	0.567172	0.873209	0.71589	LILRA3/JUN/CD79B/CD72	4
hsa04213	Longevity regulating pathway - multiple species	3/397	62/8390	0.56776	0.873209	0.71589	PRKAG3/IGF1R/CAT	3
hsa04330	Notch signaling pathway	3/397	62/8390	0.56776	0.873209	0.71589	DTX2/DLL1/NOTCH3	3
hsa00230	Purine metabolism	6/397	128/8390	0.568741	0.873209	0.71589	GMPR2/NT5E/GUK1/FHIT	6
hsa00260	Glycine, serine and threonine metabolism	2/397	40/8390	0.570944	0.873209	0.71589	GRHPR/GAMT	2
hsa04922	Glucagon signaling pathway	5/397	107/8390	0.576131	0.876526	0.718609	PRKAG3/CREB3/FBP1/AT	5
hsa03250	Viral life cycle - HIV-1	3/397	63/8390	0.578732	0.876526	0.718609	CHMP6/HGS/CD4	3
hsa00511	Other glycan degradation	1/397	18/8390	0.582489	0.877955	0.719781	GBA	1
hsa01240	Biosynthesis of cofactors	7/397	153/8390	0.591299	0.886386	0.726693	PTS/PMM2/MOCS2/HMBS	7
hsa04260	Cardiac muscle contraction	4/397	87/8390	0.59521	0.886386	0.726693	ATP1B4/CASQ2/ATP1B3/	4
hsa04261	Adrenergic signaling in cardiomyocytes	7/397	154/8390	0.598251	0.886386	0.726693	ATP1B4/CREB3/ATF2/BC	7
hsa04927	Cortisol synthesis and secretion	3/397	65/8390	0.600144	0.886386	0.726693	CREB3/ATF2/ATF4	3
hsa00531	Glycosaminoglycan degradation	1/397	19/8390	0.602288	0.886386	0.726693	GUSB	1
hsa04975	Fat digestion and absorption	2/397	43/8390	0.610661	0.894489	0.733336	PLA2G1B/FABP1	2
hsa05160	Hepatitis C	7/397	157/8390	0.618735	0.900928	0.738615	MAVS/FADD/TNF/EIF2AK	7
hsa05221	Acute myeloid leukemia	3/397	67/8390	0.620832	0.900928	0.738615	ITGAM/EIF4EBP1/CSF2	3
hsa04670	Leukocyte transendothelial migration	5/397	114/8390	0.632712	0.906018	0.742788	CD99L2/EZR/CXCL12/CD	5
hsa04910	Insulin signaling pathway	6/397	137/8390	0.635448	0.906018	0.742788	PRKAG3/SHC1/HK2/FBP1	6
hsa00770	Pantothenate and CoA biosynthesis	1/397	21/8390	0.639119	0.906018	0.742788	BCAT1	1
hsa04924	Renin secretion	3/397	69/8390	0.64078	0.906018	0.742788	EDN1/CTSB/ADCYAP1R1	3
hsa05204	Chemical carcinogenesis - DNA adducts	3/397	69/8390	0.64078	0.906018	0.742788	GSTT2B/HSD11B1/GSTA1	3
hsa04144	Endocytosis	11/397	251/8390	0.647661	0.906018	0.742788	CHMP6/DNM3/IST1/RAB1	11
hsa04912	GnRH signaling pathway	4/397	93/8390	0.647924	0.906018	0.742788	JUN/EGFR/HBEGF/ATF4	4
hsa04917	Prolactin signaling pathway	3/397	70/8390	0.650475	0.906018	0.742788	TNFSF11/SHC1/ESR1	3
hsa05230	Central carbon metabolism in cancer	3/397	70/8390	0.650475	0.906018	0.742788	RET/HK2/EGFR	3
hsa00514	Other types of O-glycan biosynthesis	2/397	47/8390	0.659172	0.914051	0.749374	C1GALT1C1/B4GALT1	2
hsa00982	Drug metabolism - cytochrome P450	3/397	72/8390	0.669301	0.92399	0.757522	GSTT2B/GSTA1/ADH1B	3
hsa05414	Dilated cardiomyopathy	4/397	96/8390	0.672516	0.924339	0.757808	TNF/ITGB5/ITGAV/DAG1	4
hsa04713	Circadian entrainment	4/397	97/8390	0.680445	0.929571	0.762098	PER3/PRKG1/GNGT1/AD	4
hsa04977	Vitamin digestion and absorption	1/397	24/8390	0.688084	0.929571	0.762098	BTD	1
hsa04350	TGF-beta signaling pathway	4/397	98/8390	0.68824	0.929571	0.762098	BAMBI/FST/TNF/INHBB	4
hsa05231	Choline metabolism in cancer	4/397	98/8390	0.68824	0.929571	0.762098	PDGFC/JUN/EIF4EBP1/E	4
hsa05212	Pancreatic cancer	3/397	76/8390	0.704703	0.943078	0.773171	GADD45B/EGFR/BCL2L1	3

hsa04611	Platelet activation	5/397	124/8390	0.705069	0.943078	0.773171	GP6/ARHGEF12/PRKG1/F	5
hsa00270	Cysteine and methionine metabolism	2/397	52/8390	0.712864	0.943078	0.773171	GOT1/BCAT1	2
hsa00592	alpha-Linolenic acid metabolism	1/397	26/8390	0.716987	0.943078	0.773171	PLA2G1B	1
hsa04950	Maturity onset diabetes of the young	1/397	26/8390	0.716987	0.943078	0.773171	HNF1A	1
hsa03018	RNA degradation	3/397	79/8390	0.729305	0.943078	0.773171	LSM8/EXOSC10/ENO2	3
hsa00062	Fatty acid elongation	1/397	27/8390	0.73042	0.943078	0.773171	ECHS1	1
hsa04966	Collecting duct acid secretion	1/397	27/8390	0.73042	0.943078	0.773171	SLC4A1	1
hsa05208	Chemical carcinogenesis - reactive oxygen species	9/397	223/8390	0.735384	0.943078	0.773171	GSTT2B/PRKD2/JUN/HM	9
hsa04921	Oxytocin signaling pathway	6/397	154/8390	0.743396	0.943078	0.773171	MYL6B/PRKAG3/PPP1R1	6
hsa05020	Prion disease	11/397	273/8390	0.751607	0.943078	0.773171	CREB3/TNF/PSMD9/IL1B/	11
hsa04218	Cellular senescence	6/397	156/8390	0.754443	0.943078	0.773171	CHEK2/GADD45B/IL1A/I	6
hsa04728	Dopaminergic synapse	5/397	132/8390	0.755375	0.943078	0.773171	CREB3/GNGT1/ATF2/AT	5
hsa04744	Phototransduction	1/397	29/8390	0.755408	0.943078	0.773171	GNGT1	1
hsa05203	Viral carcinogenesis	8/397	204/8390	0.75651	0.943078	0.773171	CREB3/EIF2AK2/JUN/IL6	8
hsa01212	Fatty acid metabolism	2/397	57/8390	0.759294	0.943078	0.773171	ECHS1/ACADM	2
hsa04110	Cell cycle	6/397	157/8390	0.759836	0.943078	0.773171	CDC26/FZR1/CHEK2/GA	6
hsa04390	Hippo signaling pathway	6/397	157/8390	0.759836	0.943078	0.773171	FZD10/AXIN1/LLGL1/GL	6
hsa04360	Axon guidance	7/397	182/8390	0.765006	0.943078	0.773171	SEMA4C/ARHGEF12/SHH	7
hsa00030	Pentose phosphate pathway	1/397	30/8390	0.767022	0.943078	0.773171	FBP1	1
hsa00053	Ascorbate and aldarate metabolism	1/397	30/8390	0.767022	0.943078	0.773171	GUSB	1
hsa00591	Linoleic acid metabolism	1/397	30/8390	0.767022	0.943078	0.773171	PLA2G1B	1
hsa04923	Regulation of lipolysis in adipocytes	2/397	58/8390	0.767762	0.943078	0.773171	PRKG1/FABP4	2
hsa00640	Propanoate metabolism	1/397	32/8390	0.788625	0.961241	0.788062	ECHS1	1
hsa05034	Alcoholism	7/397	187/8390	0.788711	0.961241	0.788062	CREB3/SHC1/GNGT1/FO	7
hsa04540	Gap junction	3/397	88/8390	0.793502	0.963317	0.789764	PDGFC/PRKG1/EGFR	3
hsa04727	GABAergic synapse	3/397	89/8390	0.799788	0.963452	0.789874	GABARAPL1/GNGT1/GA	3
hsa04976	Bile secretion	3/397	89/8390	0.799788	0.963452	0.789874	ATP1B4/ATP1B3/ATP1B2	3
hsa05206	MicroRNAs in cancer	12/397	310/8390	0.803669	0.964403	0.790654	BCL2L11/EZR/SHC1/NOT	12
hsa00040	Pentose and glucuronate interconversions	1/397	35/8390	0.817341	0.976206	0.800331	GUSB	1
hsa05014	Amyotrophic lateral sclerosis	14/397	364/8390	0.825638	0.976206	0.800331	RAB39B/DCTN2/TNF/AT	14
hsa00500	Starch and sucrose metabolism	1/397	36/8390	0.82602	0.976206	0.800331	HK2	1
hsa03030	DNA replication	1/397	36/8390	0.82602	0.976206	0.800331	FEN1	1
hsa00830	Retinol metabolism	2/397	68/8390	0.83908	0.987898	0.809916	DHRS4L2/ADH1B	2
hsa05211	Renal cell carcinoma	2/397	69/8390	0.844999	0.991127	0.812564	JUN/ARNT	2
hsa00513	Various types of N-glycan biosynthesis	1/397	39/8390	0.849667	0.992869	0.813992	B4GALT1	1
hsa04916	Melanogenesis	3/397	101/8390	0.863444	0.995315	0.815997	FZD10/CREB3/EDN1	3
hsa03440	Homologous recombination	1/397	41/8390	0.863621	0.995315	0.815997	BRIP1	1
hsa04216	Ferroptosis	1/397	41/8390	0.863621	0.995315	0.815997	HMOX1	1
hsa00562	Inositol phosphate metabolism	2/397	73/8390	0.86675	0.995315	0.815997	INPP5J/IMPA1	2
hsa04150	mTOR signaling pathway	5/397	156/8390	0.867711	0.995315	0.815997	FZD10/TNF/IGF1R/EIF4E	5
hsa03410	Base excision repair	1/397	44/8390	0.882168	1	0.819838	FEN1	1
hsa03022	Basal transcription factors	1/397	45/8390	0.887773	1	0.819838	GTF2IRD1	1
hsa04931	Insulin resistance	3/397	108/8390	0.891741	1	0.819838	PRKAG3/CREB3/TNF	3
hsa04613	Neutrophil extracellular trap formation	6/397	190/8390	0.892269	1	0.819838	CLEC7A/PADI4/TLR4/IT	6
hsa03050	Proteasome	1/397	46/8390	0.893112	1	0.819838	PSMD9	1
hsa04371	Apelin signaling pathway	4/397	139/8390	0.900909	1	0.819838	PRKAG3/NOTCH3/GNGT	4
hsa04146	Peroxisome	2/397	82/8390	0.905826	1	0.819838	MVK/CAT	2
hsa00510	N-Glycan biosynthesis	1/397	50/8390	0.912051	1	0.819838	B4GALT1	1
hsa00565	Ether lipid metabolism	1/397	50/8390	0.912051	1	0.819838	PLA2G1B	1
hsa05017	Spinocerebellar ataxia	4/397	143/8390	0.912424	1	0.819838	ATXN10/ATXN2/PSMD9/	4
hsa04310	Wnt signaling pathway	5/397	171/8390	0.913047	1	0.819838	BAMBI/FZD10/AXIN1/JU	5
hsa04913	Ovarian steroidogenesis	1/397	51/8390	0.916238	1	0.819838	IGF1R	1
hsa03460	Fanconi anemia pathway	1/397	54/8390	0.927642	1	0.819838	BRIP1	1
hsa04370	VEGF signaling pathway	1/397	59/8390	0.94331	1	0.819838	KDR	1
hsa03040	Spliceosome	4/397	157/8390	0.943909	1	0.819838	LSM8/PPIE/SMNDC1/HN	4
hsa00140	Steroid hormone biosynthesis	1/397	62/8390	0.951036	1	0.819838	HSD11B1	1
hsa00561	Glycerolipid metabolism	1/397	62/8390	0.951036	1	0.819838	LPL	1
hsa00564	Glycerophospholipid metabolism	2/397	99/8390	0.952155	1	0.819838	PLA2G1B/ACHE	2
hsa00310	Lysine degradation	1/397	63/8390	0.95337	1	0.819838	ECHS1	1
hsa03420	Nucleotide excision repair	1/397	63/8390	0.95337	1	0.819838	CETN2	1
hsa00970	Aminoacyl-tRNA biosynthesis	1/397	66/8390	0.959727	1	0.819838	HARS1	1
hsa04720	Long-term potentiation	1/397	67/8390	0.961648	1	0.819838	ATF4	1
hsa04714	Thermogenesis	6/397	232/8390	0.966944	1	0.819838	KDM3A/PRKAG3/CREB3/	6
hsa04724	Glutamatergic synapse	2/397	115/8390	0.975217	1	0.819838	GNGT1/DLG4	2
hsa04726	Serotonergic synapse	2/397	115/8390	0.975217	1	0.819838	GNGT1/CASP3	2
hsa04814	Motor proteins	4/397	193/8390	0.983527	1	0.819838	MYL6B/DCTN2/MYL1/KI	4
hsa04114	Oocyte meiosis	2/397	131/8390	0.987362	1	0.819838	CDC26/IGF1R	2
hsa05032	Morphine addiction	1/397	91/8390	0.988152	1	0.819838	GNGT1	1
hsa05012	Parkinson disease	6/397	266/8390	0.98854	1	0.819838	HTRA2/PSMD9/MAPT/CA	6
hsa03015	mRNA surveillance pathway	1/397	97/8390	0.991172	1	0.819838	CASC3	1
hsa04070	Phosphatidylinositol signaling system	1/397	97/8390	0.991172	1	0.819838	IMPA1	1
hsa04750	Inflammatory mediator regulation of TRP channels	1/397	98/8390	0.991594	1	0.819838	IL1B	1
hsa05016	Huntington disease	7/397	306/8390	0.991613	1	0.819838	DCTN2/CREB3/PSMD9/D	7
hsa04120	Ubiquitin mediated proteolysis	2/397	142/8390	0.992106	1	0.819838	CDC26/FZR1	2
hsa03013	Nucleocytoplasmic transport	1/397	108/8390	0.994855	1	0.819838	CASC3	1
hsa03008	Ribosome biogenesis in eukaryotes	2/397	164/8390	0.996967	1	0.819838	TCOF1/CSNK2A1	2
hsa03010	Ribosome	2/397	167/8390	0.997342	1	0.819838	MRPL30/MRPL28	2
hsa04723	Retrograde endocannabinoid signaling	1/397	148/8390	0.999282	1	0.819838	GNGT1	1
hsa05415	Diabetic cardiomyopathy	2/397	203/8390	0.999466	1	0.819838	GSR/CTSD	2
hsa04740	Olfactory transduction	1/397	439/8390	1	1	0.819838	PRKG1	1

Supplementary Table 17 Baseline characteristics of the included and excluded population

	Spirometric transition analysis			Proteomics analysis		
	Included population	Excluded population	*P*	Included population	Excluded population	*P*
No. of participants	32,202	470,006		40,047	462,161	
Male sex	14,851 (46.12)	214,156 (45.56)	<0.0001	17,522 (43.75)	211,455 (45.75)	<0.0001
Age, y	55 (49-61)	58 (50-63)	<0.0001	57 (49-63)	58 (50-63)	<0.0001
Ethnicity			<0.0001			<0.0001
White	31,247 (97.03)	441,192 (93.87)		37,327 (93.21)	435,112 (94.15)	
Mixed	152 (0.47)	2799 (0.60)		277 (0.69)	2674 (0.58)	
Asian or Asian British	289 (0.90)	9585 (2.04)		742 (1.85)	9132 (1.98)	
Black or Black British	200 (0.62)	7855 (1.67)		920 (2.30)	7135 (1.54)	
Other	235 (0.73)	5891 (1.25)		590 (1.47)	5536 (1.20)	
Townsend score	-2.64 (-3.89, -0.58)	-2.09 (-3.62, 0.62)	<0.0001	-2.15 (-3.66, 0.55)	-2.13 (-3.64, 0.55)	<0.0001
Smoking status			<0.0001			<0.0001
Never	20,063 (62.30)	253,302 (53.89)		22,915 (57.22)	250,450 (54.19)	
Previous	10,349 (32.14)	162,615 (34.60)		13,499 (33.71)	159,465 (34.50)	
Current	1727 (5.36)	51,216 (10.90)		3467 (8.66)	49,476 (10.71)	
Alcohol intake			<0.0001			<0.0001
Never	1442 (4.48)	39,162 (8.33)		3259 (8.14)	37,345 (8.08)	
Special occasions only	2607 (8.10)	55,364 (11.78)		4613 (11.52)	53,358 (11.55)	
One to three times a month	3566 (11.07)	52,245 (11.12)		4525 (11.30)	51,286 (11.10)	
Once or twice a week	8362 (25.97)	120,867 (25.72)		10,551 (26.35)	118,678 (25.68)	
Three or four times a week	9054 (28.12)	106,235 (22.62)		9152 (22.85)	106,227 (22.98)	
Daily or almost daily	7161 (22.24)	94,562 (20.12)		7864 (19.64)	93,859 (20.31)	
Body mass index			<0.0001			<0.0001
less than 18.5	132 (0.41)	2494 (0.53)		169 (0.42)	2457 (0.53)	
18.5 to 25	12,557 (38.99)	149,748 (31.86)		12,767 (31.88)	149,538 (32.36)	
25 to 30	13,881 (43.11)	198,130 (42.15)		17,238 (43.04)	194,773 (42.14)	
greater than 30	5620 (17.45)	116,573 (24.80)		9826 (24.54)	112,367 (24.31)	
Physical activities, min			<0.0001			<0.0001
0	348 (1.08)	8495 (1.81)		667 (1.67)	8176 (1.77)	
0-600	4530 (14.07)	62,222 (13.24)		5480 (13.68)	61,272 (13.26)	
600-3000	15,152 (47.05)	187,878 (39.97)		16,360 (40.85)	186,670 (40.39)	
greater than 3000	7757 (24.09)	115,781 (24.63)		9897 (24.71)	113,641 (24.59)	
Education status			<0.0001			<0.0001
College or University degree	15,139 (47.01)	145,918 (31.05)		13,472 (33.64)	147,585 (31.93)	
NVQ or HND or HNC or equivalent	1691 (5.25)	31,026 (6.60)		2579 (6.44)	30,138 (6.52)	
Other professional qualifications	1499 (4.65)	24,291 (5.17)		2100 (5.24)	23,690 (5.13)	
A levels/AS levels or equivalent	4311 (13.39)	50,973 (10.85)		4619 (11.53)	50,665 (10.96)	
Ordinary levels/GCSEs or equivalent / CSEs or equivalent	7532 (23.39)	124,479 (26.48)		10,664 (26.63)	121,347 (26.26)	
None of the above	1946 (6.04)	83,286 (17.72)		6193 (15.46)	79,541 (17.14)	
Income per year			<0.0001			<0.0001
greater than £100,000	2405 (7.47)	20,515 (4.36)		1913 (4.78)	21,007 (4.55)	
£52,000 to £100,000	8504 (26.41)	77,726 (16.54)		7087 (17.70)	79,143 (17.12)	
£31,000 to £51,999	8990 (27.92)	101,738 (21.65)		9052 (22.60)	101,676 (22.00)	
£18,000 to £30,999	6381 (19.82)	101,726 (21.64)		8865 (22.14)	99,242 (21.47)	
less than £18,000	3193 (9.92)	93,946 (19.99)		7392 (18.46)	89,747 (19.42)	
High density lipoprotein cholesterol			<0.0001			<0.0001
Low	4423 (13.74)	82,357 (17.52)		7093 (17.71)	79,687 (17.24)	
High	23,376 (72.59)	319,496 (67.98)		27,876 (69.61)	314,996 (68.16)	
Triglycerides			<0.0001			<0.0001
Low	19,815 (61.53)	261,049 (55.54)		23,059 (57.58)	257,805 (55.78)	
High	10,715 (33.27)	177,393 (37.74)		15,191 (37.93)	172,917 (37.41)	
Hypertension	11,897 (36.94)	209,650 (44.61)	<0.0001	17,353 (43.33)	204,194 (44.18)	<0.0001
Glycosylated hemoglobin	472 (1.47)	17,128 (3.64)	<0.0001	1368 (3.42)	16,232 (3.51)	0.3152
Glucose-lowering drugs	507 (1.57)	18,082 (3.85)	<0.0001	1433 (3.58)	17,156 (3.71)	0.1736
Lipid-lowering drugs	3553 (11.03)	86,850 (18.48)	<0.0001	6882 (17.18)	83,521 (18.07)	<0.0001

Data were presented as frequency (%) or median (P_{25}-P_{75}).

Abbreviations: A levels, Advanced Levels; AS levels, Advanced Subsidiary Levels; CSE, Certificate of Secondary Education; FEV1, forced expiratory volume in one second; FVC, forced vital capacity; GCSE, General Certificate of Secondary Education; HNC, Higher National Certificate; HND, Higher National Diploma; NVQ, National Vocational Qualification; PRISm, preserved ratio impaired spirometry; y, year.

Supplementary Table 18 SNPs excluded due to potential associations with confounders

SNPs	Traits	*P* value
rs9690193	Systolic Blood Pressure	2.80×10^{-11}
rs10278486	Whole body fat-free mass	7.50×10^{-09}
rs6480400	Low density lipoprotein cholesterol levels	1.90×10^{-08}
rs10858246	Trunk predicted mass	1.30×10^{-08}
rs4809327	Trunk predicted mass	2.50×10^{-13}
	C-reactive protein	9.40×10^{-22}

Abbreviations: SNP, single nucleotide polymorphism.
